# Reactivity of Stabilized Vinyldiazo Compounds toward Alkenyl- and Alkynylsilanes under Gold Catalysis: Regio- and Stereoselective Synthesis of Skipped Dienes and Enynes

**DOI:** 10.1021/acs.orglett.1c01381

**Published:** 2021-05-13

**Authors:** Olaya Bernardo, Kota Yamamoto, Israel Fernández, Luis A. López

**Affiliations:** † Departamento de Química Orgánica e Inorgánica, Instituto Universitario de Química Organometálica “Enrique Moles and Centro de Innovación en Química Avanzada (ORFEO−CINQA), Universidad de Oviedo, Julián Clavería 8, 33006-Oviedo, Spain; ‡ Departamento de Química Orgánica I and Centro de Innovación en Química Avanzada (ORFEO−CINQA), Facultad de Ciencias Químicas, Universidad Complutense de Madrid, 28040-Madrid, Spain

## Abstract

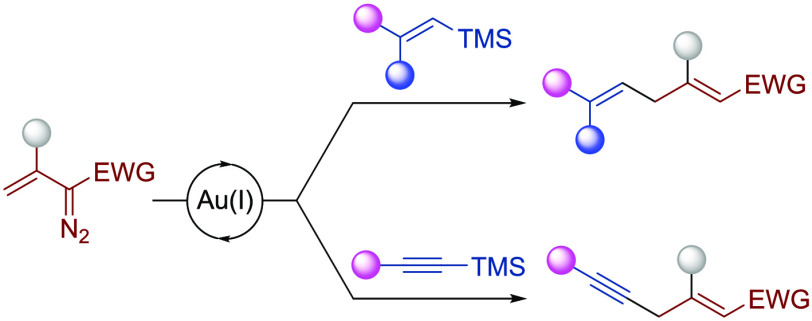

We report the gold-catalyzed reaction of vinyldiazo compounds and alkenylsilanes to produce skipped dienes, which are common structural motifs in an array of bioactive compounds. This carbon–carbon bond-forming transformation proceeds with complete regio- and stereoselectivity with the silyl group serving as a regio- and stereocontrolling element. Likewise, the use of alkynylsilanes as reaction partners yielded skipped enynes resulting from a C(sp)–C(sp^3^) coupling. Mechanistic experiments and DFT studies have provided support for a stepwise mechanism.

Since the seminal work by Nolan, Díaz-Requejo, Pérez and co-workers in 2005,^1^ the marriage of diazo reagents with gold catalysts has proven to be an extremely fertile field of research leading to reactivity modes previously unattainable by using more traditional catalysts.^2^ In this realm, initial developments focused on the use of ethyl diazoacetate and aryl substituted derivatives thereof.^3^ The implementation of these gold-based methodologies to vinyldiazo compounds is more recent. Liu and co-workers in 2011 reported the synthesis of quinoline oxide derivatives through a gold-catalyzed formal [3 + 3] cycloaddition of vinyldiazo compounds with nitroso derivatives.^4^ Since this pioneering study, the use of gold catalysts in transformations of vinyldiazo compounds has gained increasing interest.^5^ In this regard, in the past decade, our group and others have explored the reactivity of a number of unsaturated substrates toward vinyldiazo compounds under gold catalysis (Scheme 1a). In 2013, we reported the gold-catalyzed reaction of stabilized vinyldiazo compounds and unbiased alkenes to yield 2,6-diene derivatives.^6^ Despite a broad scope of alkenes, this methodology generally yields an inseparable mixture of positional isomers and *E* and *Z* stereoisomers. Concurrently, Davies and co-workers reported the highly enantioselective Au(I)-catalyzed [3 + 2] cycloaddition between vinyldiazoacetates and enol ethers.^7,8^ Our group also reported the regioselective synthesis of functionalized cyclopentene derivatives through the gold-catalyzed [3 + 2] cycloaddition of vinyldiazo compounds and styrenes.^9^

**Scheme 1:**
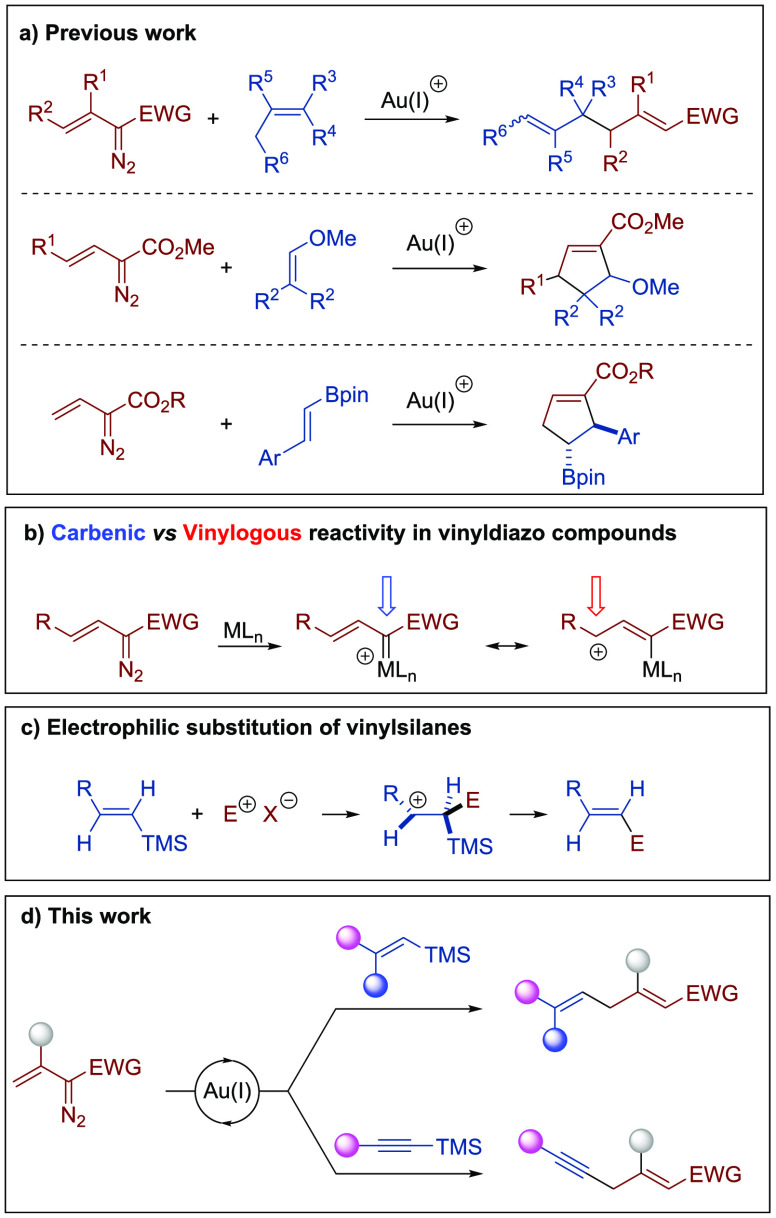
Background of the Present Study

Although a detailed mechanistic study of these transformations has not yet been undertaken, most of them have been rationalized on the basis of the initial generation of a highly electrophilic gold carbene complex, which can undergo attack of the unsaturated reagent to the vinylogous position. This vinylogous reactivity of vinyldiazo compounds had been much less common than that involving the carbenic position (Scheme 1b).^10^

On the other hand, owing to their availability, nontoxic nature, and right balance between reactivity and stability, organosilicon compounds have reached an indisputable position as valuable reagents in organic synthesis.^11^ In particular, vinylsilanes undergo a facile electrophilic substitution as a result of the ability of the carbon–silicon σ bond to stabilize an adjacent carbocation (the so-called β-silicon effect) (Scheme 1c).^12^

We surmised that merging the electrophilic character of the postulated gold carbene intermediate generated from vinyldiazo compounds with the innate ability of vinylsilanes to undergo electrophilic-induced desilylation could enable new synthetic opportunities. Herein, we report the gold-catalyzed reaction of vinyldiazo reagents with vinylsilanes leading to functionalized skipped dienes, structural motifs found in numerous natural products and bioactive compounds.^13,14^ Extension of this carbon–carbon bond formation to alkynylsilanes was also accomplished providing skipped enynes.

We began our investigation by studying the gold-catalyzed reaction of ethyl 2-diazobut-3-enoate (**1a**) and (*E*)-trimethyl(styryl)silane (**2a**). Initial experiments were conducted in dichloromethane as the solvent at room temperature in the presence of 5 mol % of the corresponding gold complex. In order to overcome potential competitive side reactions,^15^ slow addition of a solution of vinyldiazo reagent **1a** to a solution of vinylsilane **2a** (2 equiv) and the catalytic system was performed. Under these controlled addition conditions, a catalytic system composed of JohnPhosAuCl (5 mol %) and NaBAr^F^_4_ (5 mol %, BAr_4_^F–^ = 3,5-bis(trifluoromethyl)phenylborate) as a halide scavenger outperformed other gold(I) catalysts tested delivering (2*E*,5*E*)-6-phenylhexa-2,5-dienoate (**3aa**) in 85% yield after chromatographic purification (eq 1; see the Supporting Information for details on the screening study). Under these conditions, the reaction could be scaled up to 1 mmol with minimal erosion of yield (75% yield, 162.2 mg).
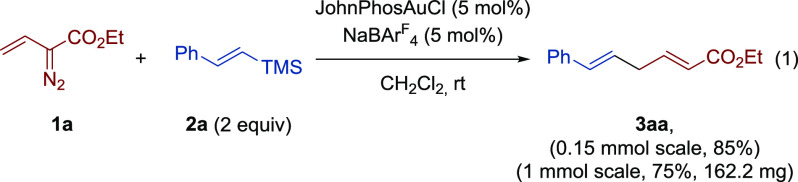
1

Next, we investigated the substrate scope of this gold-catalyzed C(sp^2^)–C(sp^3^) bond formation (Scheme 2). Keeping diazo compound **1a** as the reaction partner, we first studied the variation of the vinylsilane component **2**. In this regard, we found that *para*-substituted substrates bearing electron-donating groups at the aryl moiety, such as methyl (**2b**; R^3^ = *p*-Me-C_6_H_4_, R^4^ = H) and methoxy (**2c**; R^3^ = *p*-MeO-C_6_H_4_, R^4^ = H), engaged in this gold-catalyzed coupling reaction providing the corresponding skipped dienes **3ab** and **3ac** in 74% and 45% yield, respectively. F-, Cl-, and Br-substituted 2-arylvinylsilanes (**2d**–**f**; R^3^ = *p*-X-C_6_H_4_, R^4^ = H) also performed well to provide the corresponding dienes in good yields (**3ad**, 92%; **3ae**, 61%; **3af**, 70%). Even a strong electron-withdrawing trifluoromethyl substituent at the *para* position of the aryl ring (**2g**; R^3^ = *p*-CF_3_–C_6_H_4_, R^4^ = H) was tolerated as exemplified by product **3ag** (51% yield). However, the reaction with a substrate featuring a nitro group (**2h**; R^3^ = *p*-NO_2_–C_6_H_4_, R^4^ = H) proceeded sluggishly to produce diene **3ah** with a significantly lower yield (19%). On the other hand, *ortho*- and *meta*-substitution on the aryl group of the vinylsilane were well accommodated, as exemplified by products **3ai** (60% yield) and **3aj** (55% yield). A 2,2-disubstituted vinylsilane, namely (2,2-diphenylvinyl)trimethylsilane (**2k**; R^3^ = R^4^ = C_6_H_5_), was also transformed into the corresponding skipped diene **3ak** in good yield (75%).

**Scheme 2:**
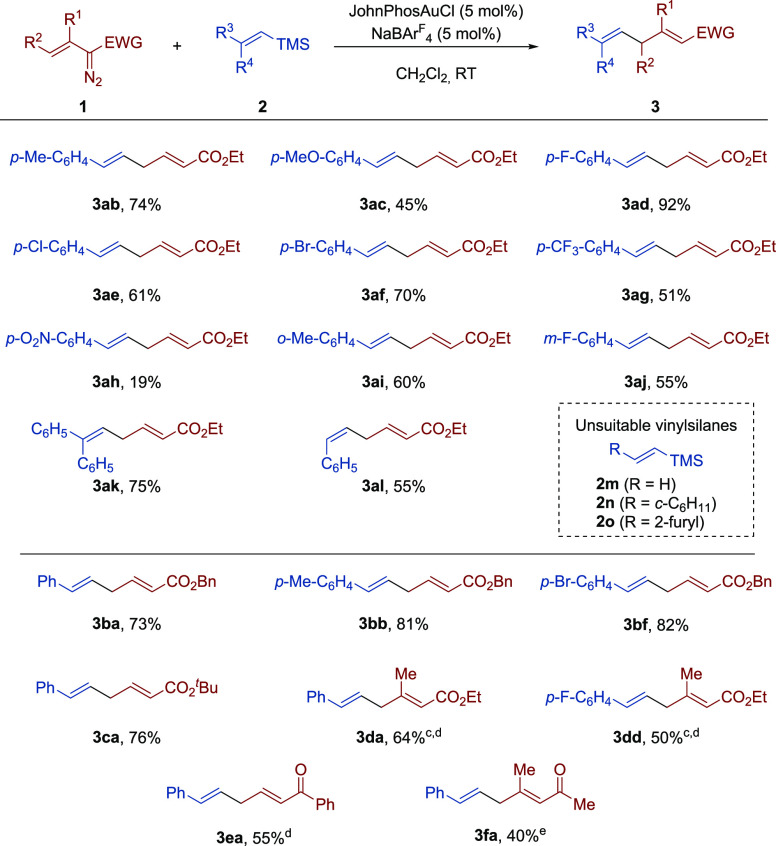
Scope of the Gold-Catalyzed Reaction of Vinyldiazo Compounds **1** and Vinylsilanes **2**^,^ Reaction conditions: **1** (0.15 mmol), **2** (0.30 mmol, 2 equiv), JohnPhosAuCl (5 mol %), NaBArF_4_ (5 mol %), CH_2_Cl_2_ (1.8 mL), rt. Yield of isolated products. Isolated as a 4:1 mixture of (2*E*,5*E*) and (2*Z*,5*E*) isomers. Four equivalents of the vinylsilane were used. Isolated as a 10:1 mixture of (3*E*,6*E*) and (3*Z*,6*E*) isomers.

We next evaluated *Z*-alkenyl silanes under our reaction conditions. Pleasingly, treatment of ethyl 2-diazobut-3-enoate (**1a**) with (*Z*)-trimethyl(styryl)silane (**2l**, R^3^ = H, R^4^ = C_6_H_5_) led to ethyl (2*E*,5*Z*)-6-phenylhexa-2,5-dienoate (**3al**) in moderate yield (55%), albeit with complete regio- and stereoselectivity.

Our studies indicate that an aryl group on the β-position of the vinylsilane is paramount to the success of the present reaction. Indeed, the parent vinyltrimethylsilane **2m** (R^3^ = R^4^ = H) proved unreactive under our optimized conditions, as did β-alkyl-substituted alkenylsilanes such as **2n** (R^3^ = cyclohexyl, R^4^ = H). Furyl-substituted alkenylsilane **2o** (R^3^ = 2-furyl, R^4^ = H) also failed to undergo the present transformation. Instead of the desired product, a mixture of products was observed.^16^

Thereupon, the structural variation of the vinyldiazo component was addressed. Benzyl 2-diazobut-3-enoate (**1b**; EWG = COOBn, R^1^ = R^2^ = H) reacted well with vinylsilanes **2a** (R^3^ = C_6_H_5_, R^4^ = H), **2b** (R^3^ = *p*-Me-C_6_H_4_, R^4^ = H), and **2f** (R^3^ = *p*-Br–C_6_H_4_, R^4^ = H), thereby providing the desired products **3ba** (73%), **3bb** (81%), and **3bf** (82%), respectively. Likewise, reaction of *tert*-butyl 2-diazobut-3-enoate (**1c**; EWG = COO^*t*^Bu, R^1^ = R^2^ = H) with **2a** furnished the expected diene **3ca** in 68% yield. Next, variation of the vinyl moiety of the diazo component was studied. Substitution at the C3 atom was tolerated as illustrated by the formation of diene **3da** in 64% yield when ethyl 2-diazo-3-metylbut-3-enoate (**1d**; EWG = COOEt; R^1^ = Me, R^2^ = H) was reacted with (*E*)-trimethyl(styryl)silane (**2a**). The reaction of diazoacetate **1d** with vinylsilane **2d** (R^3^ = *p*-F–C_6_H_4_, R^4^ = H) proceeded similarly delivering the expected product **3dd** in 50% yield. Noteworthy, substitution at the C3 atom of the vinyl moiety had a noticeable effect on the stereochemical outcome, as dienes **3da** and **3dd** were isolated as 4:1 mixtures of (2*E*,5*E*) and (2*Z*,5*E*) isomers. Unfortunately, probably because of steric hindrance, C4-substituted vinyldiazo compounds did not perform well in this transformation.

To further explore the scope of our transformation we next evaluated the reactivity of vinyldiazo ketones toward (*E*)-trimethyl(styryl)silane (**2a**).^17^ In this regard, we found that 2-diazo-1-phenylbut-3-en-1-one (**1e**; EWG = COPh, R^1^ = R^2^ = H) was also amenable to the present transformation delivering (2*E*,5*E*)-1,6-diphenylhexa-2,5-dien-1-one (**3ea**) in 55% yield with complete regio- and stereoselectivity. On the other hand, 3-diazo-4-methylpent-4-en-2-one (**1f**; EWG = COMe, R^1^ = Me, R^2^ = H) reacted with vinylsilane **2a** to give the expected product **3fa** in 40% yield as a 10:1 mixture of (3*E*,6*E*) and (3*Z*,6*E*) isomers.

Next, we briefly investigated the reactivity of silyl group protected enoldiazoacetates.^18^ Gratifyingly, reaction of TMS-protected enoldiazoacetate **1g** with silanes **2a** (Ar = Ph) and **2d** (Ar = *p*-F–C_6_H_4_) resulted in the formation of a reaction mixture from which compounds **4ga** and **4gd** were obtained in 55% and 44% yield, after column chromatography (eq 2). It should be mentioned here that, along with compounds **4**, diethyl 2-diazo-3,6-dioxooctanedioate (**5**) was also produced as a minor byproduct (8–10%).^19^
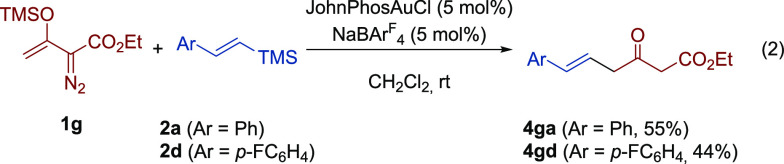
2

Several control experiments were performed to gain insight into this coupling reaction between vinyldiazo compounds and alkenylsilanes. First, we found that conducting the model reaction in deuterated dichloromethane gave the skipped diene **3aa** without incorporation of deuterium in its structure (eq 3), thus demonstrating that the *extra* hydrogen incorporated in the final product does not come from the solvent. Next, we conducted the model reaction in CH_2_Cl_2_ as solvent in the presence of D_2_O (2 equiv). Following conventional workup, diene **3aa**-D with the deuterium label incorporated exclusively in the 2-position was isolated (eq 4). Although compound **3aa**-D was isolated in low yield (20%) because of partial decomposition of the starting diazo compound, this result would confirm that external water participates in the present transformation. Finally, reaction of deuterated vinyldiazo compound **1c**-D with (*E*)-trimethyl(styryl)silane (**2a**) led to skipped diene **3ca**-D without positional scrambling of the deuterium label (eq 5).
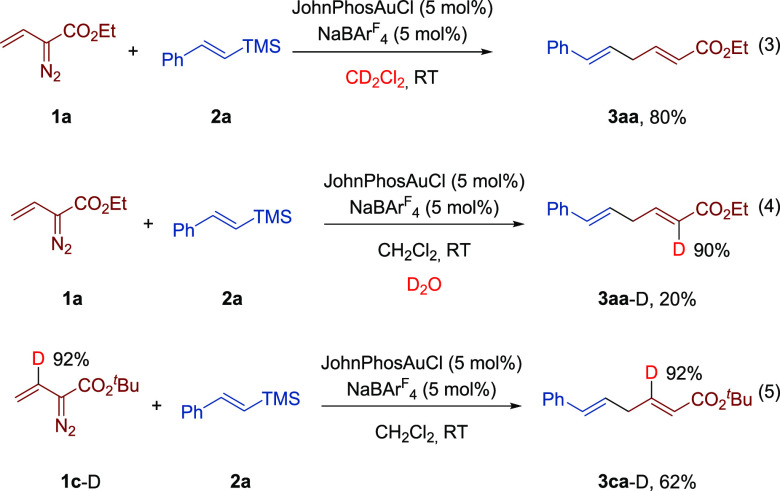
3

Based on these control experiments and previous gold-catalyzed transformations of vinyldiazo compounds, a likely mechanism is proposed in Scheme 3. The process is suggested to begin with the decomposition of the diazo derivative **1** leading to the corresponding gold carbene intermediate **I**, which would undergo attack of the vinylsilane **2** to the vinylogous position with generation of a carbocationic species **II**, stabilized by π-conjugation from the adjacent phenyl group and additionally by hyperconjugation from the TMS group placed in the β-position. Intermediate **II** would evolve through an intramolecular 1,4-migration of the TMS group delivering diene **III**,^20^ which in the presence of trace amounts of water present in the reaction medium would lead to the final diene **3**.

**Scheme 3:**
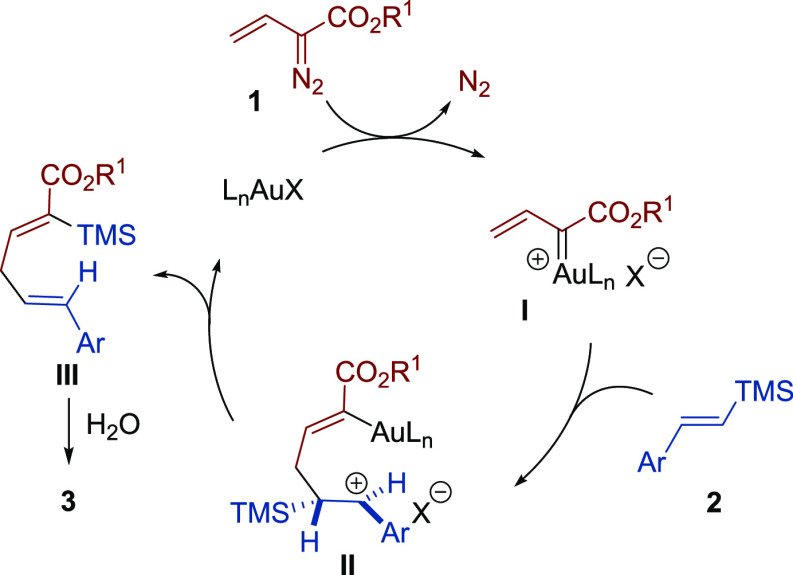
Proposed Mechanism for the Formation of Skipped Dienes **3**

Density Functional Theory (DFT) calculations provided further support for the proposed mechanism (see the Supporting Information for details). In particular, our calculations suggest that (a) the generation of the carbene intermediate **I** occurs stepwise involving the migration of the transition metal fragment from the initially formed gold(I)-alkene complex followed by N_2_ release; (b) the formation of intermediate **II** can be considered as a barrierless reaction; (c) the intramolecular 1,4-migration of the TMS group is a highly exergonic concerted process which occurs with a rather low barrier of 10.3 kcal/mol; and (d) the desilylation of intermediate **III** to provide the final product **3** is promoted by the gold catalyst in the presence of a water molecule. According to our calculations, the high exergonicity of the process (Δ*G* = −27.5 kcal/mol) would be the driving force for the key intramolecular 1,4-migration of the TMS group (transformation of intermediate **II** into **III**).

The proposed mechanism would account well for the observed stereochemical outcome. Thus, the E-configuration of the fragment arising from the vinyldiazo compound would be ascribed to the preferred conformation of the carbene intermediate.^21^ On the other hand, the retention in the configuration of the carbon–carbon double bond of the vinylsilane is well-established and has been interpreted as a consequence of the β-silicon effect.^22^

Encouraged by the results obtained with vinylsilanes, we next wondered whether alkynylsilanes could serve as suitable coupling partners, thus providing a convenient access to skipped enynes. These motifs are highly attractive because they are valuable building blocks and also structural motifs in natural products and biologically active compounds and new synthetic procedures for their synthesis would be highly desirable.^23^

We first studied the reaction of ethyl 2-diazo-3-butenoate (**1a**) with 1-phenyl-2-trimethylsilylacetylene (**6a**). Gratifyingly, after a slight reoptimization of the reaction conditions, we found that using [JohnPhosAu(MeCN)][SbF_6_] (5 mol %) as the catalyst provided ethyl (*E*)-6-phenylhex-2-en-5-ynoate (**7aa**) in 52% yield (Scheme 4).

**Scheme 4:**
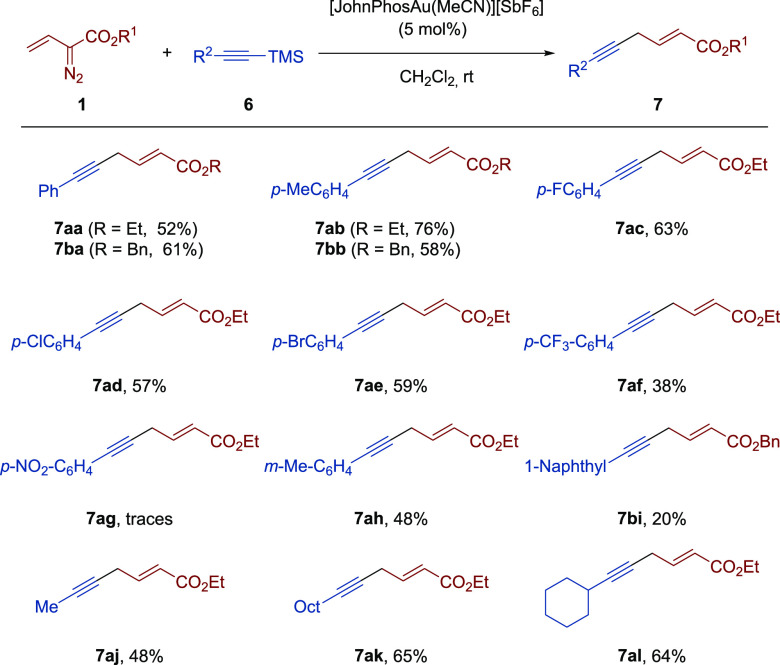
Scope of the Gold-Catalyzed Reaction of Vinyldiazoacetates **1** and Alkynylsilanes **6**^,^ Reaction conditions: **1** (0.15 mmol), **6** (0.60 mmol, 4 equiv), [JohnPhosAu(MeCN)][SbF_6_] (5 mol %), CH_2_Cl_2_ (1.8 mL), rt. Yield of isolated product.

Concerning the reaction scope of this C(sp)–C(sp^3^) coupling, a variety of aryl-substituted trimethylsilylacetylenes **6** were subjected to the previously developed reaction conditions providing the corresponding skipped enynes **7** in moderate yields and complete regio- and stereoselectivity. As seen in Scheme 4, both alkyl groups and halogens could be accommodated in the aryl moiety. In line with the results displayed by vinylsilanes, substrates bearing strong electron-withdrawing groups performed poorly. In fact, the use of a *p*-trifluoromethylphenyl substituted alkynylsilane led to the corresponding skipped enyne **7af** with a low yield (38%), while only traces of product **7ag** were observed in the reaction of **1a** with a substrate featuring a *p*-nitrophenyl group. In contrast, a substrate having a 3-methylphenyl group was suitable for the current reaction as illustrated by the formation of the corresponding product **7ah** in moderate yield. On the other hand, a 1-naphthyl-substituted alkynylsilane could also provide the desired skipped enyne **7bi** in 20% yield. Notably, alkyl-substituted alkynylsilanes also engaged in this transformation providing the corresponding skipped enynes **7aj**, **7ak**, and **7al** in moderate yields.

According to DFT calculations, this C(sp)–C(sp^3^) bond-forming transformation is suggested to occur through the generation of a vinylic carbocation followed by a concerted intramolecular 1,4-silyl migration similar to that commented above (see Supporting Information).

In conclusion, we have devised a simple and efficient route to skipped dienes based on the gold-catalyzed reaction of vinyldiazo compounds and alkenylsilanes. In most cases this transformation proceeds with complete regio- and stereoselectivity with the silyl group serving as a regio- and stereocontrolling group. Alkynylsilanes are also well suited for the current transformation providing differently substituted skipped enynes in moderate yields. The results reported herein expand the range of carbon–carbon bond-forming reactions available from vinyldiazo compounds under gold catalysis.

## Supporting Information Available

Experimental procedures, characterization data, computational details, and copies of NMR spectra (PDF)
