# Supplementary material for: Reactivity of Stabilized Vinyldiazo Compounds toward Alkenyl- and Alkynylsilanes under Gold Catalysis: Regio- and Stereoselective Synthesis of Skipped Dienes and Enynes
Source: Org Lett. 2021 May 13;23(11):4452–6. doi: 10.1021/acs.orglett.1c01381 (PMC8900156; doi:10.1021/acs.orglett.1c01381)
Supplement: Supplementary file 1 — ol1c01381_si_001.pdf [file ol1c01381_si_001.pdf]

Supporting Information

# Reactivity of Stabilized Vinyldiazo Compounds toward Alkenyl- and Alkynylsilanes under Gold-Catalysis: Regio- and Stereoselective Synthesis of Skipped Dienes and Enynes


Olaya Bernardo,[a] Kota Yamamoto, [a] Israel Fernández, [b]* and Luis A. López [a]*

[a] Departamento de Química Orgánica e Inorgánica, Instituto Universitario de Química Organometálica "Enrique Moles and Centro de Innovación en Química Avanzada (ORFEO-CINQA), Universidad de Oviedo, Julián Clavería 8, 33006-Oviedo, Spain

[b] Departamento de Química Orgánica I and Centro de Innovación en Química Avanzada (ORFEO-CINQA), Facultad de Ciencias Químicas, Universidad Complutense de Madrid, 28040-Madrid, Spain

lalg@uniovi.es

israel@quim.ucm.es

## TABLE OF CONTENTS


| | | |
|---|---|---|
| 1. | General Considerations | S-3 |
| 2. | Summary of Catalyst Screening | S-5 |
| 3. | General Procedure for the Synthesis of Skipped Dienes **3** | S-6 |
| 4. | Characterization Data of Skipped Dienes **3** | S-7 |
| 5. | Experimental Procedure for the Synthesis of **3aa** (1 mmol Scale) | S-18 |
| 6. | Synthesis of compounds **4** from TMS-protected enoldiazoacetate **1e** | S-19 |
| 7. | Synthesis of diethyl 2-diazo-3,6-dioxooctanedioate (**5**) | S-21 |
| 8. | Mechanistic experiments | S-22 |
| 9. | General Procedure for the Synthesis of Skipped Enynes **7** | S-25 |
| 10. | Characterization Data of Skipped Enynes | S-26 |
| 11. | Computational details | S-33 |
| 12. | References | S-55 |
| 13. | $^{1}$H- and $^{13}$C-NMR spectra for new compounds | S-57 |
| 14. | HRMS Analysis Reports | S-100 |

## 1. General Considerations

All reactions were carried out using oven-dried glassware under an atmosphere of nitrogen (99.99%). Dichloromethane (DCM) was distilled from $CaH_2$ prior use. The solvents used in column chromatography were obtained from commercial suppliers and used without further distillation. TLC was performed on aluminum-backed plates coated with silica gel 60 with F254 indicator (Merck), using UV light as a visualizing agent and phosphomolybdic acid in ethanol, potassium permanganate solution or *p*-anisaldehyde in ethanol, and heat as developing agent. Flash chromatography was performed on silica gel (40-60 um). $^1$H-NMR (300, 400 MHz) and $^{13}$C NMR (75.5, 100 MHz) spectra were measured in $CDCl_3$ at room temperature on a Bruker DPX-300, Bruker AV-300 MHz and Bruker AV-400 instruments, with $CDCl_3$ (δ = 7.26, $^1$H NMR; δ = 77.16, $^{13}$C NMR) as internal standard. Data are reported as follows: chemical shift, multiplicity, coupling constants (*J* in Hz) and integration.

High-resolution mass spectra (HRMS) were determined by Universidad de Oviedo with a Bruker Impact II, Q – TOF mass Spectrometer.

This study was carried out using vinyldiazoacetates **1a**-**g**, vinylsilanes **2a**-**n** and alkynylsilanes **6a**-**l** depicted in Figure S1.

Vinyldiazo compounds **1a**-**e**,[1] **1f**,[2] and **1g**[3] were prepared according to well-known procedures previously described in the literature. Vinylsilanes **2**[4] and alkynylsilanes **6**[5] were prepared according to literature procedures. All other reagents used in this work were of the best commercial grade available and used without further purification.

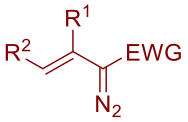


**1a** ($R^1 = R^2$ = H; EWG = COOEt)
**1b** ($R^1 = R^2$ = H; EWG = COOBn)
**1c** ($R^1 = R^2$ = H; EWG = COO$^t$Bu)
**1c**-D ($R^1$ D; $R^2$ = H; EWG = COO$^t$Bu)
**1d** ($R^1$ = Me; $R^2$ = H; EWG = COOEt)
**1e** ($R^1 = R^2$ = H; EWG = COPh)
**1f** ($R^1$ = Me; $R^2$ = H; EWG = COMe)
**1g** ($R^1$ = OTMS; $R^2$ = H; EWG = COOEt)

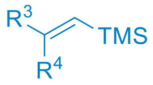


**2a** ($R^3$ = Ph; $R^4$ = H)
**2b** ($R^3$ = *p*-Me-$C_6H_4$; $R^4$ = H)
**2c** ($R^3$ = *p*-MeO-$C_6H_4$; $R^4$ = H)
**2d** ($R^3$ = *p*-F-$C_6H_4$; $R^4$ = H)
**2e** ($R^3$ = *p*-Cl-$C_6H_4$; $R^4$ = H)
**2f** ($R^3$ = *p*-Br-$C_6H_4$; $R^4$ = H)
**2g** ($R^3$ = *p*-$F_3C$-$C_6H_4$; $R^4$ = H)
**2h** ($R^3$ = *p*-$O_2N$-$C_6H_4$; $R^4$ = H)
**2i** ($R^3$ = *o*-Me-$C_6H_4$; $R^4$ = H)
**2j** ($R^3$ = *m*-F-$C_6H_4$; $R^4$ = H)
**2k** ($R^3 = R^4$ = Ph)
**2l** ($R^3$ = H; $R^4$ = Ph)
**2m** ($R^3 = R^4$ = H)
**2n** ($R^3$ = *c*-$C_6H_{11}$; $R^4$ = H)
**2o** ($R^3$ = 2-Furyl; $R^4$ = H)

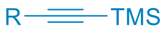


**6a** (R = Ph)
**6b** (R = *p*-Me-$C_6H_4$)
**6c** (R = *p*-F-$C_6H_4$)
**6d** (R = *p*-Cl-$C_6H_4$)
**6e** (R = *p*-Br-$C_6H_4$)
**6f** (R = *p*-$F_3C$-$C_6H_4$)
**6g** (R = *p*-$O_2N$-$C_6H_4$)
**6h** (R = *m*-Me-$C_6H_4$)
**6i** (R = 1-Naphthyl)
**6j** (R = Me)
**6k** (R = n-$C_8H_{17}$)
**6l** (R = *c*-$C_6H_{11}$)

**Figure S1**. Starting materials used in this work

## 2. Summary of Catalyst Screening

**Table S1.** Optimization of reaction conditions[a,b]

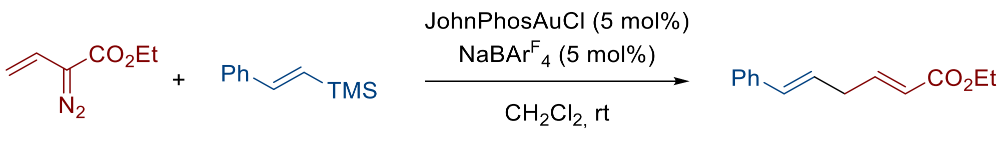


| Entry | Variation from optimal conditions | Yield (%)[c] |
|---|---|---|
| 1 | No changes | 85 |
| 2 | [JohnPhosAu($CH_3CN$)][BARF] used instead | 82 |
| 3 | [JohnPhosAu($CH_3CN$)][$SbF_6$] used instead | 62 |
| 4 | [JohnPhosAu][$NTf_2$] used instead | 50 |
| 5 | [IPrAu($CH_3CN$)][$SbF_6$] used instead | 44 |
| 6 | [IPrAu][$NTf_2$] used instead | 40 |
| 7 | $Rh_2(OAc)_4$ used instead | n.d. |
| 8 | $Cu(MeCN)_4BF_4$ used instead | n.d. |
| 9 | 1,2-dichloroethane instead of dichloromethane | 72 |
| 10 | Toluene instead of dichloromethane | n.d. |
| 11 | Hexane instead of dichloromethane | n.d. |
| 12 | THF instead of DCM | n.d. |
| 13 | TBDMS instead of TMS | 58 |
| 14 | Triphenylsilyl instead of TMS | n.d. |

[a] These experiments were performed on a 0.15 mmol scale. [b] Slow addition of **1a** over 30 minutes via syringe pump. [c] Yield of the isolated product after chromatographic purification.

## 3. General Procedure for the Synthesis of Skipped Dienes 3

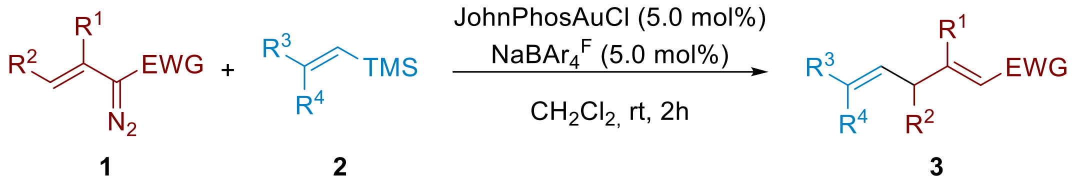


To a solution of the corresponding vinylsilane **2** (0.30 mmol), JohnphosAuCl (4.0 mg, 5.0 mol%) and $NaBAr_4^F$ (6.6 mg, 5.0 mol%) in $CH_2Cl_2$ (1.5 mL) was slowly added over 30 min (via a syringe pump) a solution of the corresponding vinyldiazo compound **1** (0.15 mmol) in $CH_2Cl_2$ (0.3 mL). After the addition was complete, the resulting mixture was stirred at room temperature for 2 hours. Then, the solvent was removed under reduced pressure and the resulting mixture was purified by flash chromatography (silica gel, hexanes/ethyl acetate 40:1) to yield skipped dienes **3**.

All skipped dienes **3**, except **3aa** and **3ak**, are new compounds.

## 4. Characterization Data of Skipped Dienes 3

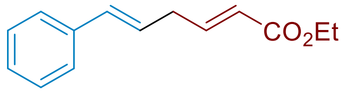

**3aa**

**Ethyl (2*E*,5*E*)-6-phenylhexa-2,5-dienoate** (**3aa**). The general procedure was followed using ethyl 2-diazobut-3-enoate (**1a**, 21.0 mg, 0.15 mmol) and (*E*)-trimethyl(styryl)silane (**2a**, 52.9 mg, 0.30 mmol). Final chromatographic purification (silica gel, hexanes/ethyl acetate 40:1) afforded compound **3aa** (27.6 mg, 85%) as a yellowish oil.

**$^1$H NMR** ($CDCl_3$, 300 MHz): δ 7.40 -7.23 (m, 5H), 7.08 (dt, 1H, *J* = 15.7 and 6.5 Hz), 6.48 (dt, 1H, *J* = 15.8 and 1.5 Hz), 6.22 (dt, 1H, *J* = 15.9 and 6.7 Hz), 5.94 (dt, 1H, *J* = 15.7 and 1.7 Hz), 4.23 (q, 2H, *J* = 7.1 Hz), 3.16-3.10 (m, 2H), 1.32 (t, 3H, *J* = 7.1 Hz).

**$^{13}$C{H} NMR** ($CDCl_3$, 75 MHz): δ 166.5, 146.5, 137.1, 132.5, 128.6, 127.4, 126.2, 125.4, 122.3, 60.3, 35.3, 15.3.

**HRMS** (EI) m/z: $[M + H]^+$ Calcd for $C_{14}H_{17}O_2$ 217.1223; Found 217.1219.

The spectroscopic data of compound **3aa** were consistent with those reported in the literature.[6]

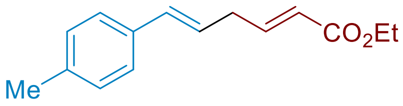

**3ab**

**Ethyl (2*E*,5*E*)-6-(*p*-tolyl)hexa-2,5-dienoate** (**3ab**). The general procedure was followed using ethyl 2-diazobut-3-enoate (**1a**, 21.0 mg, 0.15 mmol) and (*E*)-trimethyl(4-methylstyryl)silane (**2b**, 57.1 mg, 0.30 mmol). Final chromatographic purification (silica gel, hexanes/ethyl acetate 40:1) afforded compound **3ab** (25.6 mg, 74%) as a yellowish oil.

**$^1$H NMR** ($CDCl_3$, 300 MHz): δ 7.31-7.24 (m, 2H), 7.14 (d, 2H, *J* = 7.9 Hz), 7.10-7.00 (m, 1H), 6.44 (dt, 1H, *J* = 15.8 and 1.6 Hz), 6.16 (dt, 1H, *J* = 15.8 and 6.7 Hz), 5.92 (dt, 1H, *J* = 15.7 and 1.7 Hz), 4.22 (q, 2H, *J* = 7.1 Hz), 3.14-3.09 (m, 2H), 2.36 (s, 3H), 1.31 (t, 3H, *J* = 7.1 Hz).

**$^{13}$C{H} NMR** ($CDCl_3$, 75 MHz): δ 166.6, 146.7, 137.2, 134.3, 132.3, 129.3, 126.0, 124.3, 122.2, 60.3, 35.3, 21.2, 14.3.

**HRMS** (EI) m/z: $[M + H]^+$ Calcd for $C_{15}H_{19}O_2$ 231.1380; Found 231.1378.

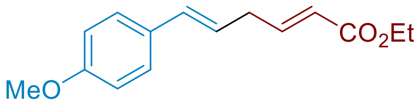


**3ac**

**Ethyl (2*E*,5*E*)-6-(4-methoxyphenyl)hexa-2,5-dienoate** (**3ac**). The general procedure was followed using ethyl-2-diazobut-3-enoate (**1a**, 21.0 mg, 0.15 mmol) and (*E*)-(4-methoxystyryl)trimethylsilane (**2c**, 61.9 mg, 0.30 mmol). Final chromatographic purification (silica gel, hexanes/ethyl acetate 40:1) afforded compound **3ac** (16.6 mg, 45%) as a yellow oil.

**$^{1}$H NMR** ($CDCl_3$, 300 MHz): δ 7.31 (d, 2H, *J* = 8.7 Hz), 7.10-7.00 (m, 1H), 6.90- 6.83 (m, 2H), 6.45-6.36 (m, 1H), 6.06 (dt, 1H, *J* = 15.8 and 6.8 Hz), 5.91 (dt, 1H, *J* = 15.7 and 1.7 Hz), 4.22 (q, 2H, *J* = 7.1 Hz), 3.83 (s, 3H), 3.12-3.08 (m, 2H), 1.31 (t, *J* = 7.1 Hz, 3H).

**$^{13}$C{H} NMR** ($CDCl_3$, 75 MHz): δ 166.6, 159.1, 146.8, 131.8, 129.9, 127.3, 123.2, 122.1, 114.0, 60.3, 55.3, 35.3, 14.3.

**HRMS** (EI) m/z: $[M + Na]^+$ Calcd for $C_{15}H_{18}O_3Na$ 269.1148; Found 269.1143.

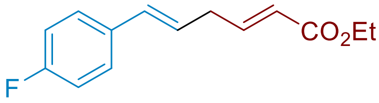


**3ad**

**Ethyl (2*E*,5*E*)-6-(4-fluorphenyl)hexa-2,5-dienoate** (**3ad**). The general procedure was followed using ethyl 2-diazobut-3-enoate (**1a**, 21.0 mg, 0.15 mmol) and (*E*)-(4-fluorostyryl)trimethylsilane (**2d**, 58.3 mg, 0.30 mmol). Final chromatographic purification (silica gel, hexanes/ethyl acetate 40:1) afforded compound **3ad** (32.3 mg, 92%) as a yellow oil.

$^{1}$**H NMR** ($CDCl_3$, 300 MHz): δ 7.36-7.28 (m, 2H), 7.11-6.97 (m, 3H), 6.43 (dt, 1H, *J* = 15.8 and 1.6 Hz), 6.12 (dt, 1H, *J* = 15.8 and 6.1 Hz), 5.91 (dt, 1H, *J* = 15.7 and 1.7 Hz), 4.22 (q, 2H, *J* = 7.1 Hz), 3.14-3.08 (m, 2H), 1.31 (t, 3H, *J* = 7.1 Hz).

$^{13}$**C{H} NMR** ($CDCl_3$, 75 MHz): δ 166.5, 162.2 ($J_{C\text{-}F}$ = 245.0 Hz), 146.3, 133.2 ($J_{C\text{-}F}$ = 3.8 Hz), 131.3, 127.6 ($J_{C\text{-}F}$ = 8.3 Hz), 125.2 ($J_{C\text{-}F}$= 2.3 Hz), 122.3, 115.5 ($J_{C\text{-}F}$= 21.0 Hz), 60.3, 35.2, 14.3.

$^{19}$**F NMR** ($CDCl_3$, 282 MHz) = δ - 114.8.

**HRMS** (EI) m/z: $[M + H]^+$ Calcd for $C_{14}H_{16}FO_2$ 235.1129; Found 235.1131.

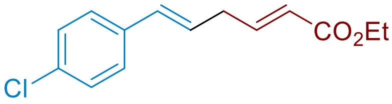


**3ae**

**Benzyl (2*E*,5*E*)-6-(4-chlorophenyl)hexa-2,5-dienoate** (**3ae**). The general procedure was followed using ethyl 2-diazobut-3-enoate (**1a**, 21.0 mg, 0.15 mmol) and (*E*)-(4-chlorostyryl)trimethylsilane (**2e**, 63.2 mg, 0.30 mmol). Final chromatographic purification (silica gel, hexanes/ethyl acetate 40:1) afforded compound **3ae** (22.9 mg, 61%) as a yellow oil.

$^{1}$**H NMR** ($CDCl_3$, 300 MHz): δ 7.29 (app s, 4H), 7.05 (dt, 1H, *J* = 15.7 and 6.5 Hz), 6.42 (d, 1H, *J* = 15.9 Hz), 6.19 (dt, 1H, *J* = 15.9 and 6.7 Hz), 5.91 (d, 1H, *J* = 15.7 Hz), 4.22 (q, 2H, *J* = 7.2 Hz), 3.15-3.09 (m, 2H), 1.30 (d, 3H, *J* = 7.2 Hz).

$^{13}$**C{H} NMR** ($CDCl_3$, 75 MHz): δ 166.5, 146.1, 135.6, 133.0, 131.3, 128.7, 127.3, 126.2, 122.5, 60.3, 35.2, 14.3.

**HRMS** (EI) m/z: $[M + K]^+$ Calcd for $C_{14}H_{15}ClO_2K$ 289.0392; Found 289.0392.

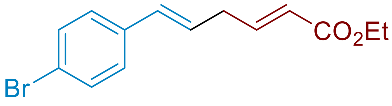


**3af**

**Ethyl (2*E*,5*E*)-6-(4-bromophenyl)hexa-2,5-dienoate** (**3af**). The general procedure was followed using ethyl 2-diazobut-3-enoate (**1a**, 21.0 mg, 0.15 mmol) and (*E*)-(4-

bromostyryl)trimethylsilane (**2f**, 76.6 mg, 0.30 mmol). Final chromatographic purification (silica gel, hexanes/ethyl acetate 40:1) afforded compound **3af** (31.0 mg, 70%) as a yellow oil.

**$^{1}$H NMR** ($CDCl_3$, 300 MHz): δ 7.44 (d, 2H, *J* = 8.5 Hz), 7.23 (d, 2H, *J* = 8.5 Hz), 7.04 (dt, 1H, *J* = 15.7 and 6.5 Hz), 6.40 (d, 1H, *J* = 15.8 Hz), 6.20 (dt, 1H, *J* = 15.8 and 6.6 Hz), 5.91 (dt, 1H, *J* = 15.6 and 1.7 Hz), 4.22 (q, 2H, *J* = 7.1 Hz), 3.14-3.09 (m, 2H), 1.31 (t, 3H, *J* = 7.1 Hz).

**$^{13}$C{H} NMR** ($CDCl_3$, 75 MHz): δ 166.4, 146.0, 136.0, 131.7, 131.3, 127.7, 126.3, 122.5, 121.2, 60.3, 35.2, 14.3.

**HRMS** (EI) m/z: $[M + H]^+$ Calcd for $C_{14}H_{16}BrO_2$ 295.0328; Found 295.0324.

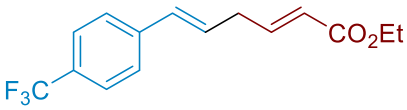


**3ag**

**Ethyl (2*E*,5*E*)-6-(4-(trifluoromethyl)phenyl)hexa-2,5-dienoate** (**3ag**). The general procedure was followed using ethyl-2-diazobut-3-enoate (**1a**, 21.0 mg, 0.15 mmol) and (*E*)-trimethyl(4-(trifluoromethyl)styryl)silane (**2g**, 73.3 mg, 0.30 mmol). Final chromatographic purification (silica gel, hexanes/ethyl acetate 40:1) afforded compound **3ag** (21.7 mg, 51%) as a yellow oil.

**$^{1}$H NMR** ($CDCl_3$, 300 MHz): δ 7.58 (d, 2H, *J* = 8.2 Hz), 7.46 (d, 2H, *J* = 8.2 Hz), 7.06 (dt, 1H, *J* = 15.6 and 6.6 Hz), 6.50 (d, 1H, *J* = 15.9 Hz), 6.31 (dt, 1H, *J* = 15.9 and 6.6 Hz), 5.93 (d, 1H, *J* = 15.8 Hz), 4.23 (q, 2H, *J* = 7.1 Hz), 3.18-3.14 (m, 2H), 1.32 (t, 3H, *J* = 7.1 Hz).

**$^{13}$C{H} NMR** ($CDCl_3$, 75 MHz): δ 165.8, 146.2, 140.9, 131.6, 128,7, 128.1 ($J_{C\text{-}F}$= 258.8 Hz), 126.7, 125.9 ($J_{C\text{-}F}$= 3.8 Hz), 123.1, 122.8, 60.8, 35.6, 14.7.

**$^{19}$F NMR** ($CDCl_3$, 282 MHz): δ - 62.46.

**HRMS** (EI) m/z: $[M + H]^+$ Calcd for $C_{15}H_{16}F_3O_2$ 285.1097; Found 285.1096

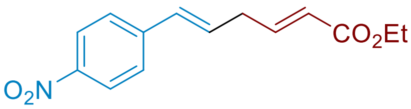


**3ah**

**Ethyl (2*E*,5*E*)-6-(4-nitrophenyl)hexa-2,5-dienoate** (**3ah**). The general procedure was followed using ethyl-2-diazobut-3-enoate (**1a**, 21.0 mg, 0.15 mmol) and (*E*)-(4-nitrostyryl)trimethylsilane (**2h**, 66.4 mg, 0.30 mmol). Final chromatographic purification (silica gel, hexanes/ethyl acetate 40:1) afforded compound **3ah** (7.5 mg, 19%) as a yellow oil.

**$^{1}$H NMR** ($CDCl_3$, 300 MHz): δ 8.20 (d, 2H, *J* = 8.8 Hz), 7.50 (d, 2H, *J* = 8.8 Hz), 7.05 (dt, 1H, *J* = 15.6 and 6.5 Hz), 6.54 (d, 1H, *J* = 15.8 Hz), 6.44 (dt, 1H, *J* = 15.8 and 6.6 Hz), 5.93 (d, 1H, *J* = 15.7 Hz), 4.23 (d, 2H, *J* = 7.2 Hz), 3.21-3.17 (m, 2H), 1.30 (d, 3H, *J* = 7.2 Hz).

**$^{13}$C{H} NMR** ($CDCl_3$, 75 MHz): δ 166.3, 146.9, 145.1, 143.4, 130.7, 130.6, 126.6, 124.0, 123.0, 60.4, 35,2, 14.2.

**HRMS** (EI) m/z: $[M + K]^+$ Calcd for $C_{14}H_{15}NO_4K$ 300.0633; Found 300.0628.

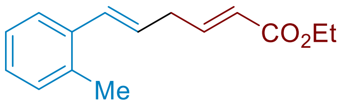

**3ai**

**Ethyl (2*E*,5*E*)-6-(*o*-tolyl)hexa-2,5-dienoate** (**3ai**). The general procedure was followed using ethyl 2-diazobut-3-enoate (**1a**, 21.0 mg, 0.15 mmol) and (*E*)-trimethyl(2-methylstyryl)silane (**2i**, 57.1 mg, 0.30 mmol). Final chromatographic purification (silica gel, hexanes/ethyl acetate 40:1) afforded compound **3ai** (20.7 mg, 60%) as a yellow oil.

**$^{1}$H NMR** ($CDCl_3$, 300 MHz): δ 7.45-7.42 (m, 1H), 7.20-7.15 (m, 3H), 7.08 (dt, 1H, *J* = 15.6 and 6.5 Hz), 6.65 (dt, 1H, *J* = 15.7 and 1.6 Hz), 6.08 (dt, 1H, *J* = 15.7 and 6.8 Hz), 5.93 (dt, 1H, *J* = 15.6 and 1.7 Hz), 4.22 (q, 2H, *J* = 7.1 Hz), 3.18-3.13 (m, 2H), 2.35 (s, 3H), 1.31 (t, 3H, *J* = 7.1 Hz).

**$^{13}$C{H} NMR** ($CDCl_3$, 75 MHz): δ 166.6, 146.7, 136.2, 135.2, 130.5, 130.2, 127.4, 126.8, 126.1, 125.6, 122.2, 60.3, 35.6, 19.8, 14.3.

**HRMS** (EI) m/z: $[M + H]^+$ Calcd for $C_{15}H_{19}O_2$ 231.1380; Found 231.1388.

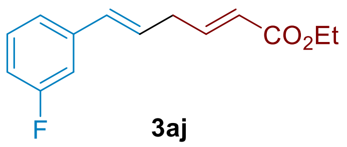

**3aj**

**Ethyl (2*E*,5*E*)-6-(3-fluorophenyl)hexa-2,5-dienoate** (**3aj**). The general procedure was followed using ethyl 2-diazobut-3-enoate (**1a**, 21.0 mg, 0.15 mmol) and (*E*)-(3-fluorostyryl)trimethylsilane (**2j**, 58.3 mg, 0.30 mmol). Final chromatographic purification(silica gel, hexanes/ethyl acetate 40:1) afforded compound **3aj** (19.3 mg, 55%) as a yellow oil.

**$^{1}$H NMR** ($CDCl_3$, 300 MHz): δ 7.32-7.24 (m, 1H), 7.14-7.00 (m, 3H), 6.97-6.91 (m, 1H), 6.43 (d, 1H, *J* = 15.9 Hz), 6.22 (dt, 1H, *J* = 15.9 and 6.6 Hz), 5.92 (dt, 1H, *J* = 15.7 and 1.7 Hz), 4.22 (q, 2H, *J* = 7.1 Hz), 3.15-3.11 (m, 2H), 1.31 (t, 3H, *J* = 7.1 Hz).

**$^{13}$C{H} NMR** ($CDCl_3$, 75 MHz): δ 166.4, 163.1 ($J_{C-F}$ = 243.7 Hz), 146.0, 139.4 ($J_{C-F}$ = 7.7 Hz), 131.4 ($J_{C-F}$ = 2.6 Hz), 130.0 ($J_{C-F}$ = 8.5 Hz), 126.9, 122.5, 122.0 ($J_{C-F}$ = 2.7 Hz), 114.2 ($J_{C-F}$ = 21.3 Hz), 112.6 ($J_{C-F}$ = 21.6 Hz), 60.3, 35.1, 14.3.

**$^{19}$F NMR** ($CDCl_3$, 282 MHz) = δ - 113.6.

**HRMS** (EI) m/z: $[M + H]^+$ Calcd for $C_{14}H_{16}FO_2$ 235.1129 ; Found 235.1129.

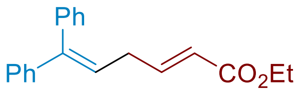

**3ak**

**Ethyl (*E*)-6,6-diphenylhexa-2,5-dienoate** (**3ak**). The general procedure was followed using ethyl 2-diazobut-3-enoate (**1a**, 21.0 mg, 0.15 mmol) and (2,2-diphenylvinyl)trimethylsilane (**2k**, 75.7 mg, 0.30 mmol). Final chromatographic purification (silica gel, hexanes/ethyl acetate 40:1) afforded compound **3ak** (32.9 mg, 75%) as a yellow oil.

**$^{1}$H NMR** ($CDCl_3$, 300 MHz): δ 7.40-7.17 (m, 10H), 7.04 (dt, 1H, *J* = 15.7 and 6.1 Hz), 6.10 (t, 1H, *J* = 7.6 Hz), 5.91 (dt, 1H, *J* = 15.7 and 1.8 Hz), 4.22 (q, 2H, *J* =7.1 Hz), 3.06-3.01 (m, 2H), 1.32 (t, 3H, *J* = 7.1 Hz).

**$^{13}$C{H} NMR** ($CDCl_3$, 75 MHz): δ 166.6, 147.7, 144.7, 142.5, 139.8, 130.0, 128.8, 128.6, 127.8, 124.2, 122.2, 60,7, 32.9, 14.7.

**HRMS** (EI) m/z: $[M + H]^+$ Calcd for $C_{20}H_{21}O_2$ 293.1536, Found 293.1540.

The spectroscopic data of compound **3ak** were consistent with those reported in the literature.[7]

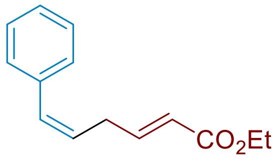


**3al**

**Ethyl (2*E*,5*Z*)-6-phenylhexa-2,5-dienoate** (**3al**). The general procedure was followed using ethyl 2-diazobut-3-enoate (**1a**, 21.0 mg, 0.15 mmol) and (*Z*)-trimethyl(styryl)silane (**2l**, 52.9 mg, 0.30 mmol). Final chromatographic purification (silica gel, hexanes/ethyl acetate 40:1) afforded compound **3al** (17.8 mg, 55%) as a yellow oil.

**$^1$H NMR** ($CDCl_3$, 300 MHz): δ 7.39-7.34 (m, 2H), 7.28-7.24 (m, 3H), 7.07 (dt, 1H, *J* = 15.7 and 6.0 Hz), 6.63 (d, 1H, *J* = 11.5 Hz), 5.94 (dt, 1H, *J* = 15.7 and 1.8 Hz), 5.72 (dt, 1H, *J* = 11.5 and 7.5 Hz), 4.22 (q, 2H, *J* = 7.1 Hz), 3.25-3.19 (m, 2H), 1.31 (t, 3H, *J* = 7.1 Hz).

**$^{13}$C{H} NMR** ($CDCl_3$, 75 MHz): δ 166.6, 146.8, 136.7, 131.6, 128.5, 128.3, 127.0, 126.8, 122.0, 60,3, 31.1, 14.3.

**HRMS** (EI) m/z: $[M + H]^+$ Calcd for $C_{14}H_{17}O_2$ 217.1223; Found 217.1223.

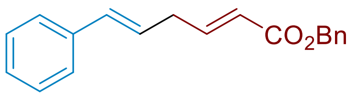


**3ba**

**Benzyl (2*E*,5*E*)-6-phenylhexa-2,5-dienoate** (**3ba**). The general procedure was followed using benzyl 2-diazobut-3-enoate (**1b**, 30.3 mg, 0.15 mmol) and (*E*)-trimethyl(styryl)silane (**2a**, 52.9 mg, 0.30 mmol). Final chromatographic purification (silica gel, hexanes/ethyl acetate 40:1) afforded compound **3ba** (30.5 mg, 73%) as a yellow oil.

**$^1$H NMR** ($CDCl_3$, 300 MHz): δ 7.48-7.22 (m, 10H), 7.13 (dt, 1H, *J* = 15.7 and 6.5 Hz), 6.45 (dt, 1H, *J* = 15.8 and 1.5 Hz), 6.22 (dt, 1H, *J* = 15.8 and 6.7 Hz), 5.99 (dt, 1H, *J* = 15.6 and 1.7 Hz), 5.22 (s, 2H), 3.16-3.12 (m, 2H).

$^{13}$**C{H} NMR** ($CDCl_3$, 75 MHz): δ 166.3, 147.2, 137.0, 136.1, 132.6, 128.6, 128.3, 128.2, 127.5, 126.2, 125.3, 122.0, 66.2, 35.3.

**HRMS** (EI) m/z: $[M + Na]^+$ Calcd for $C_{19}H_{18}O_2Na$ 301.1199; Found 301.1194.

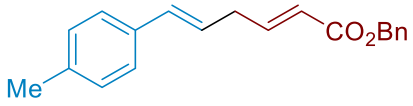


**3bb**

**Benzyl (2*E*,5*E*)-6-(*p*-tolyl)hexa-2,5-dienoate** (**3bb**). The general procedure was followed using benzyl 2-diazobut-3-enoate (**1b**, 30.3 mg, 0.15 mmol) and (*E*)-trimethyl(4-methylstyryl)silane (**2b**, 57.1 mg, 0.30 mmol). Final chromatographic purification (silica gel, hexanes/ethyl acetate 40:1) afforded compound **3bb** (35.5 mg, 81%) as a yellow oil.

$^1$**H NMR** ($CDCl_3$, 300 MHz): δ 7.46-7.34 (m, 5H), 7.29-7.26 (m, 2H), 7.21-7.07 (m, 3H), 6.45 (dt, 1H, $J$ = 15.8 and 1.5 Hz), 6.16 (dt, 1H, $J$ = 15.8 and 6.7 Hz), 5.99 (dt, 1H, $J$ = 15.6 and 1.7 Hz), 5.22 (s, 2H), 3.16-3.10 (m, 2H), 2.37 (s, 3H).

$^{13}$**C{H} NMR** ($CDCl_3$, 75 MHz): δ 166.3, 147.5, 137.3, 136.1, 134.3, 132.5, 129.3, 128.6, 128.3, 126.1, 124.2, 121.8, 66.2, 35.3, 21.2.

**HRMS** (EI) m/z: $[M + Na]^+$ Calcd for $C_{20}H_{20}O_2Na$ 315.1356; Found 315.1352.

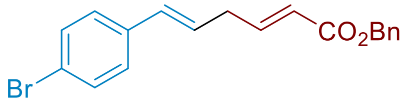


**3bf**

**Benzyl (2*E*,5*E*)-6-(4-bromophenyl)hexa-2,5-dienoate** (**3bf**). The general procedure was followed using benzyl 2-diazobut-3-enoate (**1b**, 30.3 mg, 0.15 mmol) and (*E*)-(4-bromostyryl)trimethylsilane (**2f**, 76.6 mg, 0.30 mmol). Final chromatographic purification (silica gel, hexanes/ethyl acetate 40:1) afforded compound **3bf** (43.9 mg, 82%) as a yellow oil.

$^1$**H NMR** ($CDCl_3$, 300 MHz): δ 7.48-7.32 (m, 7H), 7.29-7.18 (m, 2H), 7.10 (dt, 1H, $J$ = 15.7 and 6.5 Hz), 6.40 (dt, 1H, $J$ = 15.9 and 1.5 Hz), 6.20 (dt, 1H, $J$ = 15.9 and 6.7 Hz), 5.97 (dt, 1H, $J$ = 15.6 and 1.7 Hz), 5.21 (s, 2H), 3.15-3.10 (m, 2H).

$^{13}C\{H\}$ **NMR** ($CDCl_3$, 75 MHz): δ 166.2, 146.8, 136.0, 135.9, 131.7, 131.4, 128.6, 128.3, 127.7, 126.2, 122.1, 121.2, 66.2, 35.3.

**HRMS** (EI) m/z: $[M + Na]^+$ Calcd for $C_{19}H_{17}BrO_2Na$ 379.0304; found: 379.0303.

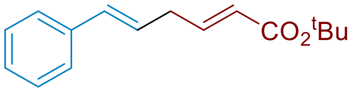


**3ca**

***tert*-butyl (2*E*,5*E*)-6-phenylhexa-2,5-dienoate** (**3ca**). The general procedure was followed using *tert*-butyl-2-diazobut-3-enoate (**1c**, 25.2 mg, 0.15 mmol) and (*E*)-trimethyl(styryl)silane (**2a**, 52.9 mg, 0.30 mmol). Final chromatographic purification (silica gel, hexanes/ethyl acetate 40:1) afforded compound **3ca** (27.9 mg, 76%) as a yellow oil.

$^{1}H$ **NMR** ($CDCl_3$, 300 MHz): δ 7.40-7.22 (m, 5H), 6.96 (dt, 1H, *J* = 15.6 and 6.5 Hz), 6.47 (d, 1H, *J* = 15.9 Hz), 6.21 (dt, 1H, *J* = 15.9 and 6.7 Hz), 5.84 (dt, 1H, *J* = 15.7 and 1.6 Hz), 3.13-3.08 (m, 2H), 1.51 (s, 9H).

$^{13}C\{H\}$ **NMR** ($CDCl_3$, 75 MHz): δ 165.9, 145.2, 134.1, 132.3, 128.6, 127.4, 126.1, 125.7, 124.0, 80.2, 35.2, 28.2.

**HRMS** (EI) m/z: $[M + Na]^+$ Calcd for $C_{16}H_{20}O_2Na$ 267.1356; found: 267.1349.

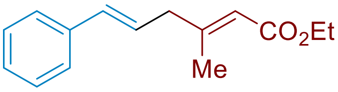


**3da**

**Ethyl (2*E*,5*E*)-3-methyl-6-phenylhexa-2,5-dienoate** (**3da**). The general procedure was followed using ethyl 2-diazo-3-methylbut-3-enoate (**1d**, 23.1 mg, 0.15 mmol) and (*E*)-trimethyl(styryl)silane (**2a**, 105.8 mg, 0.60 mmol). Final chromatographic purification (silica gel, hexanes/ethyl acetate 40:1) afforded compound **3da** [22.1 mg, 64%, inseparable 4:1 mixture of (2*E*,5*E*) and (2*Z*,5*E*) isomers] as a colorless oil.

$^{1}H$ **NMR** ($CDCl_3$, 300 MHz): δ (2*E*-isomer) 7.40-7.18 (m, 5H), 6.47 (d, 1H, *J* = 15.9 Hz), 6.19 (dt,1H, *J* = 15.8 and 7.0 Hz), 5.77-5.76 (m, 1H), 4.18 (q, 2H, *J* = 7.1 Hz), 3.04 (d, 2H, *J* = 7.1 Hz), 2.23 (d, 3H, *J* = 1.3 Hz), 1.30 (t, 3H, *J* = 7.1 Hz). (2*Z*-isomer, only clearly

assignable signals are listed) δ 3.57 (d, 2H, *J* = 7.6 Hz), 1.94 (d, 3H, *J* = 1.4 Hz),1.32 (t, 3H, *J* = 7.1 Hz). The rest of signal overlap with those of the major isomer.

**$^{13}$C{H} NMR** ($CDCl_3$, 75 MHz): δ (2*E*-isomer) 166.8, 157.9, 137.1, 133.0, 128.6, 127.4, 126.2, 125.8, 116.5, 59.6, 44.0, 19.0, 14.3.

**HRMS** (EI) m/z: $[M + Na]^+$ Calcd for $C_{15}H_{18}O_2Na$ 253.1199; Found 253.1201.

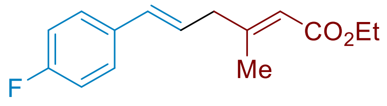


**3dd**

**Ethyl (2*E*,5*E*)-6-(4-fluorophenyl)-3-methylhexa-2,5-dienoate** (**3dd**). The general procedure was followed using ethyl 2-diazo-3-methylbut-3-enoate (**1d**, 23.1 mg, 0.15 mmol) and (*E*)-(4-fluorostyryl)trimethylsilane (**2d**, 116.6 mg, 0.60 mmol). Final chromatographic purification (silica gel, hexanes/ethyl acetate 40:1) afforded compound **3dd** [18.6 mg, 50%, inseparable 4:1 mixture of (2*E*,5*E*) and (2*Z*,5*E*) isomers] as a colorless oil.

**$^1$H NMR** ($CDCl_3$, 300 MHz): δ (2*E*-isomer) 7.36-7.31 (m, 2H), 7.05-6.99 (m, 2H), 6.43 (d, 1H, *J* = 15.8 Hz), 6.10 (dt, 1H, *J* = 15.8 and 7.0 Hz), 5.76-5.75 (m, 1H), 4.19 (q, 2H, *J* = 7.1 Hz), 3.02 (d, 2H, *J* = 6.8 Hz), 2.22 (d, 3H, *J* = 1.4 Hz), 1.30 (t, 3H, *J* = 7.0 Hz). (2*Z*-isomer, only clearly assignable signals are listed) δ 6.46 (d, 1H, *J* = 16.1 Hz). 3.55 (d, 2H, *J* = 6.8 Hz), 1.94 (d, 3H, *J* = 1.4 Hz), 1.31 (t, 3H, *J* = 7.1 Hz). The rest of signals overlap with those of the major isomer.

**$^{13}$C{H} NMR** ($CDCl_3$, 75 MHz): δ (2*E*-isomer) 166.7, 162.2 ($J_{C-F}$ = 245.0 Hz), 157.8, 133.2 ($J_{C-F}$ = 3.3 Hz), 131.8, 130.6, 125.5 ($J_{C-F}$ = 1.7 Hz), 116.6, 115.4 ($J_{C-F}$ = 21.4 Hz), 59.6, 43.9, 19.0, 14.3.

**$^{19}$F NMR** ($CDCl_3$, 282 MHz): δ - 114.8 (2*E*-isomer); -115.3 (2*Z*-isomer).

**HRMS** (EI) m/z: $[M + Na]^+$ Calcd for $C_{15}H_{17}FO_2Na$ 271.1105; found: 271.1102.

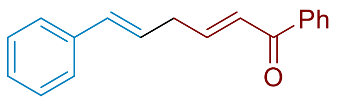


**3ea**

**(2*E*,5*E*)-1,6-diphenylhexa-2,5-dien-1-one** (**3ea**). The general procedure was followed using 2-diazo-1-phenylbut-3-en-1-one (**1e**, 25.8 mg, 0.15 mmol) and (*E*)-trimethyl(styryl)silane (**2a**, 105.8 mg, 0.60 mmol). Final chromatographic purification (silica gel, hexanes/ethyl acetate 40:1) afforded compound **3ea** (20.5 mg, 55%) as a yellow oil.

$^1$**H NMR** ($CDCl_3$, 300 MHz): δ 7.97-7.94 (m, 2H), 7.61-7.12 (m, 9H), 6.98 (dt, 1H, *J* = 15.2 and 1.5 Hz), 6.52 (d, 1H, *J* = 16.0 Hz), 6.29 (dt, 1H, *J* = 16.0 and 6.7 Hz), 3.28-3.24 (m, 2H).

$^{13}$**C{H} NMR** ($CDCl_3$, 75 MHz): δ 191.2, 147.4, 138.2, 137.5, 133.2, 133.0, 129.0, 127.9, 127.0, 126.6, 125.9, 36.3.

**HRMS** (EI) m/z: $[M + H]^+$ Calcd for $C_{18}H_{17}O$ 249.1274; Found 249.1271.

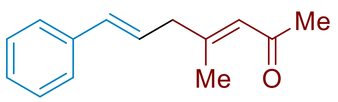


**3fa**

**(3*E*, 6*E*)-4-methyl-7-phenylhepta-3,6–dien–2-one** (**3fa**). The general procedure was followed using 3-diazo-4-methylpent-4-en-2-one (**1f**, 18.6 mg, 0.15 mmol) and (*E*)-trimethyl(styryl)silane (**2a**, 105.8 mg, 0.60 mmol). Final chromatographic purification (silica gel, hexanes/ethyl acetate 10:1) afforded compound **3fa** [12.0 mg, 40%, inseparable 40:1 mixture of (3*E*,6*E*) and (3*Z*,6*E*) isomers] as a yellow oil.

$^1$**H NMR** ($CDCl_3$, 300 MHz): δ (3*E*-isomer) 7.41-7.24 (m, 5H), 6.48 (dt, 1H, *J* = 15.9 and 1.6 Hz), 6.27-6.13 (m, 2H), 3.03 (dt, 2H, *J* = 7.0 and 1.4 Hz), 2.21 (s 3H), 2.19 (d, 3H, *J* = 1.3 Hz). (3*Z*-isomer, only clearly assignable signals are listed) δ 3.51 (d, 2H, *J* = 6.9 Hz), 2.22 (s, 3H), 1.93 (d, 3H, *J* = 1.4 Hz).

$^{13}$**C{H} NMR** ($CDCl_3$, 75 MHz): δ (3*E*-isomer) 198,9, 156.3, 137.1, 133.0, 128.6, 127.5, 126.2, 125.8, 124.3, 44.2, 31.8, 19.5.

**HRMS** (EI) m/z: $[M + Na]^+$ Calcd for $C_{14}H_{16}ONa$ 223.1093; found: 223.1091.

## 5. Experimental Procedure for the Synthesis of 3aa (1 mmol Scale)

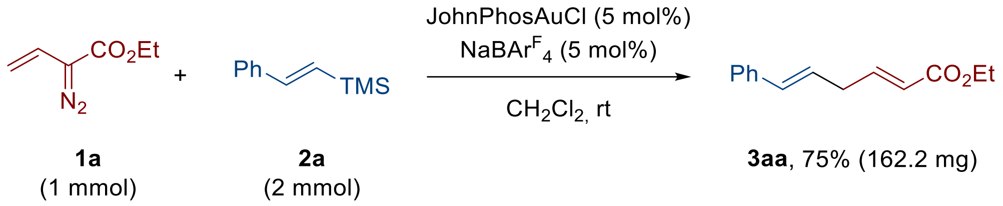


To a solution of the corresponding vinylsilane (*E*)-trimethyl(styryl)silane (**2a**, 353 mg, 2.0 mmol), JohnphosAuCl (26.5 mg, 5.0 mol%) and $NaBAr_4^F$ (44.3 mg, 5.0 mol%) in $CH_2Cl_2$ (6.5 mL) was slowly added over 30 min (via a syringe pump) a solution of the corresponding ethyl 2-diazobut-3-enoate (**1a**, 140 mg, 1.0 mmol) in $CH_2Cl_2$ (3 mL). After the addition was complete, the resulting mixture was stirred at room temperature for 2 hours. Then, the solvent was removed under reduced pressure and the resulting mixture was purified by flash chromatography (silica gel, hexanes/ethyl acetate 40:1) to yield **3aa** (75%, 162.2 mg) as a yellow oil. The spectroscopic data of compound **3aa** match with those reported for the 0.15 mmol scale (see page S-6)

## 6. Synthesis of compounds 4 from TMS-protected enoldiazoacetate 1g

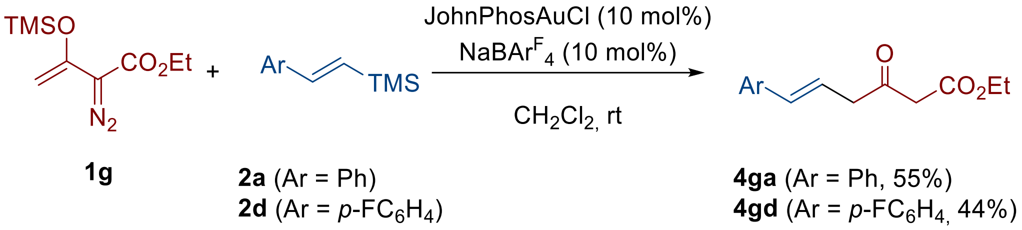


To a solution of the corresponding vinylsilane **2** (0.30 mmol), JohnphosAuCl (4.0 mg, 5.0 mol%) and $NaBAr_4^F$ (6.6 mg, 5.0 mol%) in $CH_2Cl_2$ (1.5 mL) was slowly added over 30 min (via a syringe pump) a solution of ethyl 2-diazo-3-[(trimethylsilyl)oxy]but-3-enoate **1g** (34.2 mg, 0.15 mmol) in $CH_2Cl_2$ (0.3 mL). After the addition was complete, the resulting mixture was stirred at room temperature for 2 hours. Then, the solvent was removed under reduced pressure and the resulting mixture was purified by flash chromatography to yield compounds **4** along with diethyl 2-diazo-3,6-dioxooctanedioate (**5**).

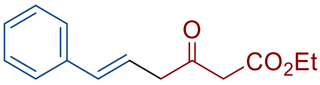


**4ga**

**Ethyl (*E*)-3-oxo-6-phenylhex-5-enoate (4ga)**. Prepared from ethyl 2-diazo-3-[(trimethylsilyl)oxy]but-3-enoate **1g** (34.2 mg, 0.15 mmol) and (*E*)-trimethyl(styryl)silane (**2a**, 52.9 mg, 0.30 mmol). Final chromatographic purification (silica gel, hexanes/ethyl acetate 40:1) afforded compound **4ga** (19.2 mg, 55%) as a yellow oil. Compound **4ga** was isolated as a 10:1 mixture of keto and enol tautomers.

**$^1$H NMR** ($CDCl_3$, 300 MHz): keto tautomer, δ 7.41-7.25 (m, 5H), 6.53 (dt, 1H, *J* = 16.0 and 1.5 Hz), 6.30 (dt, 1H, *J* = 15.9 and 7.0 Hz), 4.22 (q, 2H, *J* = 7.1 Hz), 3.54 (s, 2H), 3.48 (dd, 2H, *J* = 7.0 and 1.4 Hz), 1.30 (t, 3H, *J* = 7.1 Hz). Representative signals for the enol tautomer: δ 12.15 (s, 1H), 5.08 (s, 1H), 3.14 (d, 2H, *J* = 7.0 Hz).

**$^{13}$C{H} NMR** ($CDCl_3$, 75 MHz): keto tautomer, δ 200.7, 167.0, 136.6, 134.6, 128.6, 127.8, 126.3, 120.9, 61.5, 48.8, 46.9, 14.1.

**HRMS** (EI) m/z: $[M + Na]^+$ Calcd $C_{14}H_{16}O_3Na$ 255.0992; Found 255.0992.

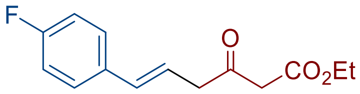


**4gd**

**Ethyl (*E*)-6-(4-fluorophenyl)-3–oxohex–5-enoate** (**4gd**). Prepared from ethyl 2-diazo-3-[(trimethylsilyl)oxy]but-3-enoate **1g** (34.2 mg, 0.15 mmol) and (*E*)-(4-fluorostyryl)trimethylsilane (**2d**, 58.3 mg, 0.30 mmol). Final chromatographic purification (silica gel, hexanes/ethyl acetate 40:1) afforded compound **4gd** (16.5 mg, 44%) as a yellow oil. Compound **4gd** was isolated as a 10:1 mixture of keto and enol tautomers.

**$^1$H NMR** ($CDCl_3$, 300 MHz): keto tautomer, δ 7.38-7.31 (m, 2H). 7.05-6.99 (m, 2H), 6.53-6.42 (m, 1H), 6.22 (dt, 1H, *J* = 15.9 and 7.0 Hz), 4.22 (q, 2H, *J* = 7.1 Hz), 3.53 (s, 2H), 3.47 (dd, 2H, *J* = 6.9 and 1.4 Hz), 1.30 (t, 3H, *J* = 7.1 Hz). Representative signals for the enol tautomer: δ 12.16 (s, 1H), 5.07 (s, 1H), 3.12 (d, 2H, *J* = 7.1 Hz).

**$^{13}$C{H} NMR** ($CDCl_3$, 75 MHz): keto tautomer, δ 200.6, 167.0, 162.4 ($J_{C\text{-}F}$= 245.3 Hz), 133.3, 132.8 ($J_{C\text{-}F}$= 3.75 Hz), 127.8 ($J_{C\text{-}F}$= 7.5 Hz), 120.6, 115.5 ($J_{C\text{-}F}$= 21.7 Hz), 61.5, 48.9, 46.8, 14.1.

**$^{19}$F NMR** ($CDCl_3$, 282 MHz): δ - 114.3.

**HRMS** (EI) m/z: $[M + Na]^+$ Calcd for $C_{14}H_{15}FO_3Na$ 273.0897; Found 273.0894.

## 7. Synthesis of diethyl 2-diazo-3,6-dioxooctanedioate (5)

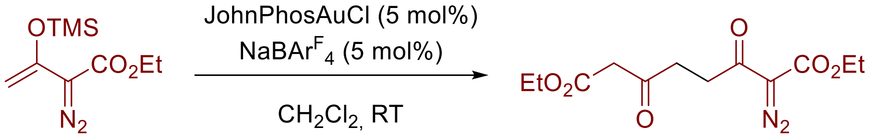


**1g** **5**, 68%

To a solution of JohnphosAuCl (4.0 mg, 5.0 mol%) and $NaBAr_4^F$ (6.6 mg, 5.0 mol%) in $CH_2Cl_2$ (1.5 mL) was added a solution of ethyl 2-diazo-3-[(trimethylsilyl)oxy]but-3-enoate **1g** (34.2 mg, 0.15 mmol) in $CH_2Cl_2$ (0.3 mL). After the addition was complete, the resulting mixture was stirred at room temperature until disappearance of **1g** (1 h, checked by TLC). The solvent was removed under reduced pressure and the resulting mixture was purified by flash chromatography (silica gel, hexanes/ethyl acetate 40:1) to yield diethyl 2-diazo-3,6-dioxooctanedioate (**5**, 14.5 mg, 68%) as a yellow oil.

**IR** (neat): 2984, 2136, 1739, 1713, 1652, 1369, 1301, 1027, 732, 702 $cm^{-1}$.

**$^1$H NMR** ($CDCl_3$, 300 MHz): $\delta$ 4.31 (q, 2H, *J* = 7.1 Hz), 4.21 (q, 2H, *J* = 7.2 Hz), 3.54 (s, 2H), 3.20-3.16 (m, 2H), 2.92-2.88 (m, 2H), 1.34 (t, 3H, *J* = 7.2 Hz), 1.29 (t, 3H, *J* = 7.1 Hz).

**$^{13}$C{H} NMR** ($CDCl_3$, 75 MHz): $\delta$ 201.9, 191.3, 167.6, 162.0, 61.9, 61.8, 49.8, 36.7, 34.8, 14.8, 14.5. The signal corresponding to the carbon of the diazo function is not observed.

**HRMS** (EI) m/z: $[M + H]^+$ Calcd for $C_{12}H_{17}N_2O_6$ 285.1081; found: 285.1078.

## 8. Mechanistic experiments

### A) Experiment in $CD_2Cl_2$ as solvent

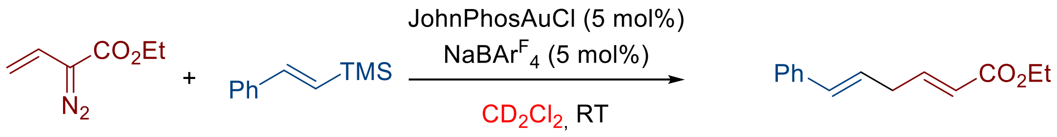


**1a** **2a** **3aa**, 80%

To a solution of (*E*)-trimethyl(styryl)silane (**2a**, 52.9 mg, 0.30 mmol), JohnphosAuCl (4.0 mg, 5.0 mol%) and $NaBAr_4^F$ (6.6 mg, 5.0 mol%) in $CD_2Cl_2$ (1.5 mL) was slowly added over 30 min (via a syringe pump) a solution of ethyl 2-diazobut-3-enoate (**1a**, 21.0 mg, 0.15 mmol) in $CD_2Cl_2$ (0.3 mL). After the addition was complete, the resulting mixture was stirred at room temperature for 2 hours. Then, the solvent was removed under reduced pressure and the resulting mixture was purified by flash chromatography (silica gel, hexanes/ethyl acetate 40:1) to yield skipped diene **3aa** (26.0 mg, 80%) without incorporation of deuterium in its structure.

### B) Reaction in the presence of $D_2O$

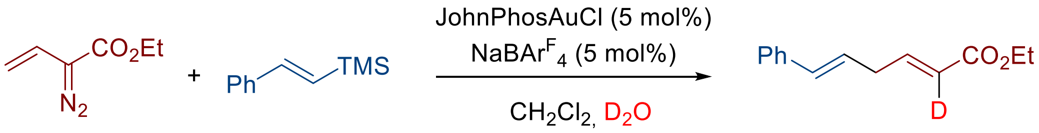


**1a** **2a** **3aa**-D

To a solution of (*E*)-trimethyl(styryl)silane (**2a**, 52.9 mg, 0.30 mmol), JohnphosAuCl (4.0 mg, 5.0 mol%) and $NaBAr_4^F$ (6.6 mg, 5.0 mol%) in $CH_2Cl_2$ (1.5 mL) was added $D_2O$ (2 equivalents). To the resulting mixture was slowly added over 30 min (via a syringe pump) a solution of ethyl 2-diazobut-3-enoate (**1a**, 21.0 mg, 0.15 mmol) in $CH_2Cl_2$ (0.3 mL). After the addition was complete, the resulting mixture was stirred at room temperature for 2 hours. Then, the solvent was removed under reduced pressure and the resulting mixture was purified by flash chromatography (silica gel, hexanes/ethyl acetate 40:1) to yield skipped diene **3aa**-D (6.4 mg, 20%) as a yellow oil. As shown in Figure S2, compound **3aa**-D incorporated the deuterium label in the 2-position.

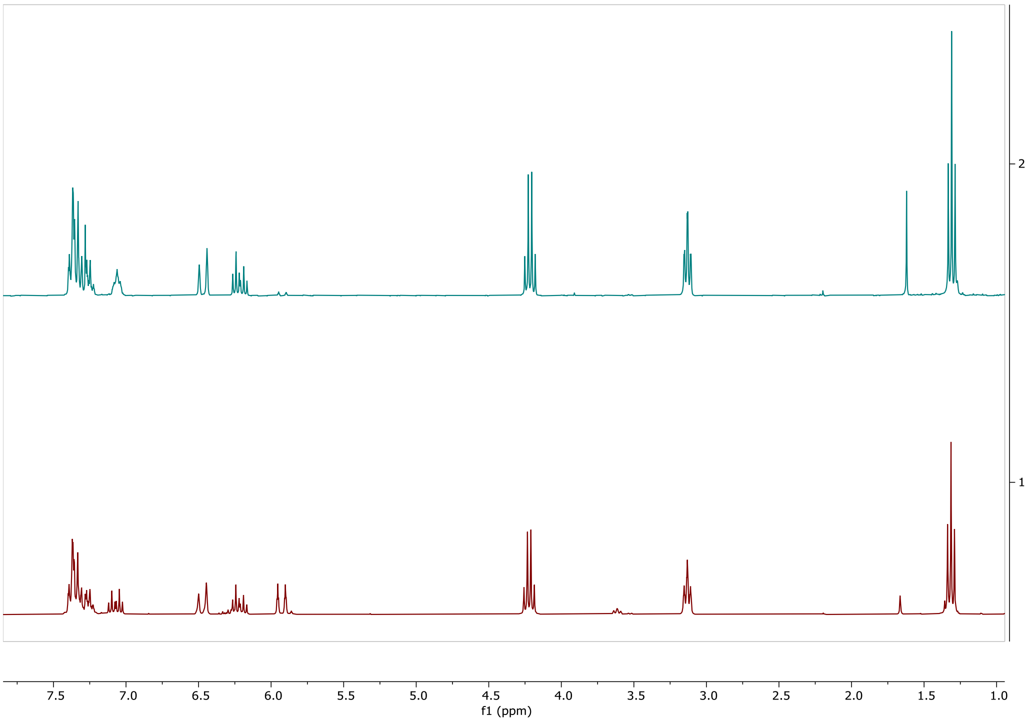


**Figure S2**. $^{1}$H-NMR ($CDCl_3$, 300 MHz) spectra of diene **3aa** (obtained under the standard conditions, red spectrum) and **3aa**-D (obtained in the presence of $D_2O$, green spectrum)

## C) Reaction of deuterated vinyldiazo compound 1c-D with silane 2a

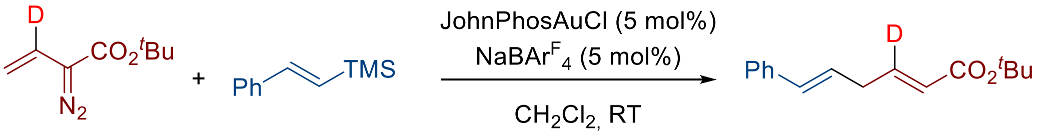


**1c**-D **2a** **3ca**-D, 62%

To a solution of (*E*)-trimethyl(styryl)silane (**2a**, 52.9 mg, 0.30 mmol), JohnphosAuCl (4.0 mg, 5.0 mol%) and $NaBAr_4^F$ (6.6 mg, 5.0 mol%) in $CH_2Cl_2$ (1.5 mL) was slowly added over 30 min (via a syringe pump) a solution of *tert*-butyl 2-diazobut-3-enoate-3-d (**1a**c-D, 25.4 mg, 0.15 mmol) in $CH_2Cl_2$ (0.3 mL). After the addition was complete, the resulting mixture was stirred at room temperature for 2 hours. Then, the solvent was removed under reduced pressure and the resulting mixture was purified by flash chromatography (silica gel, hexanes/ethyl acetate 40:1) to yield skipped diene **3ca**-D (62% yield) as a

yellow oil. As shown in Figure S3, compound **3ca**-D incorporated the deuterium label exclusively in the 3-position.

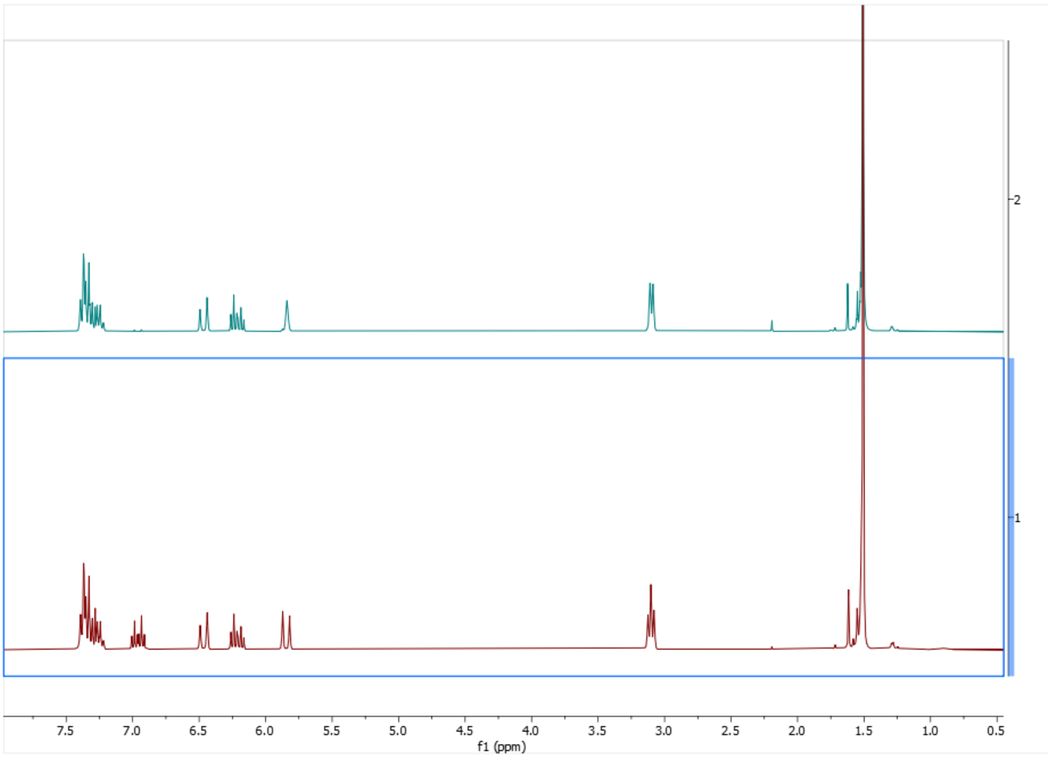


**Figure S3**. $^{1}$H-NMR ($CDCl_3$, 300 MHz) spectra of diene **3ca** (obtained from vinyldiazo compound **1c**, red spectrum) and **3ca**-D (obtained from vinyldiazo acetate **1c**-D, green spectrum)

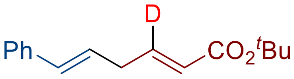


**3ca**-D

**$^{1}$H NMR** ($CDCl_3$, 300 MHz): δ 7.40-7.24 (m, 5H), 6.47 (dt, 1H, *J* =15.9 and 1.5 Hz), 6.23 (dt, 1H, *J* = 15.9 and 6.0 Hz), 5.85-5.83 (m, 1H), 3.12-3.08 (m, 2H), 1.51 (s, 9H).

**$^{13}$C{H} NMR** ($CDCl_3$, 75 MHz): δ 165.9, 144.9 ($J_{C\text{-}D}$ = 24.0 Hz), 137.2, 132.3, 128.6, 127.4, 126.1, 125.7, 124.0, 80.2, 35.0, 28.1.

**HRMS** (EI) m/z: $[M + Na]^+$ Calcd for $C_{16}H_{19}DO_2Na$ 268.1418; Found 268.1420.

## 9. General Procedure for the Synthesis of Skipped Enynes 7

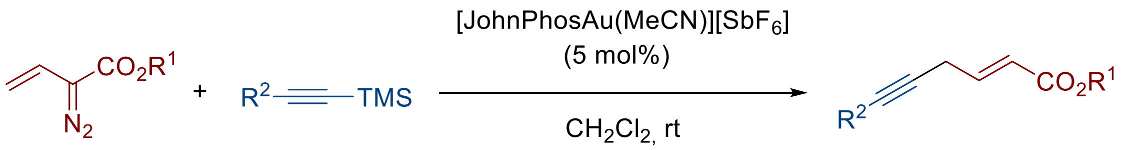


**1** **6** **7**

To a solution of the corresponding alkynylsilane **6** (0.60 mmol) and [JohnPhosAu(MeCN)][$SbF_6$] (4.0 mg, 5.0 mol%) in $CH_2Cl_2$ (1.5 mL) was slowly added over 30 min (via a syringe pump) a solution of the corresponding vinyldiazo compound **1** (0.15 mmol) in $CH_2Cl_2$ (0.3 mL). After the addition was complete, the resulting mixture was stirred at room temperature for 2 hours. Then, the solvent was removed under reduced pressure and the resulting mixture was purified by flash chromatography (silica gel, hexanes/ethyl acetate 40:1) to yield skipped enynes **7**.

All skipped enynes **7**, except **7aa**, are new compounds.

## 10. Characterization Data of Skipped Enynes 7

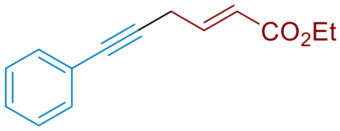


**7aa**

**Ethyl (*E*)-6-phenylhex-2-en-5-ynoate** (**7aa**). The general procedure was followed using ethyl 2-diazobut-3-enoate (**1a**, 21.0 mg, 0.15 mmol) and trimethyl(phenylethynyl)silane (**6a**, 104.6 mg, 0.60 mmol). Final chromatographic purification (silica gel, hexanes/ethyl acetate 40:1) afforded compound **7aa** (16.7 mg, 52%) as a yellow oil.

**$^{1}$H NMR** ($CDCl_3$, 300 MHz): δ 7.49-7.42 (m, 2H), 7.36-7.30 (m, 3H), 7.01 (dt, 1H, $J$ = 15.4 and 5.1 Hz), 6.25 (d, 1H, $J$ = 15.5 Hz), 4.23 (q, 2H, $J$ = 7.1 Hz), 3.38 (dd, 2H, $J$ = 5.1 and 2.0 Hz), 1.32 (t, 3H, $J$ = 7.1 Hz).

**$^{13}$C{H} NMR** ($CDCl_3$, 75 MHz): δ 166.8, 142.7, 132.1, 128.7, 128.6, 123.4, 121.1, 84.6, 84.5, 60.9, 22.9, 14.7.

**HRMS** (EI) m/z: $[M + H]^+$ Calcd for $C_{14}H_{14}O_2Na$ 237.0886; Found 237.0889.

The spectroscopic data of compound **7aa** were consistent with those reported in the literature.[8]

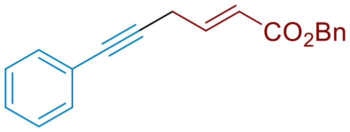


**7ba**

**Benzyl (*E*)-6-phenylhex-2-en-5-ynoate** (**7ba**). The general procedure was followed using benzyl 2-diazobut-3-enoate (**1b**, 30.3 mg, 0.15 mmol) and trimethyl(phenylethynyl)silane (**6a**, 104.6 mg, 0.60 mmol). Final chromatographic purification (silica gel, hexanes/ethyl acetate 40:1) afforded skipped enyne **7ba** (25.3 mg, 61%) as a yellow oil.

**$^{1}$H NMR** ($CDCl_3$, 300 MHz): δ 7.46-7.31 (m, 10H), 7.07 (dt, 1H, $J$ = 15.5 and 5.0 Hz), 6.30 (dt, 1H, $J$ = 15.5 and 2.0 Hz), 5.23 (s, 2H), 3.39 (dd, 2H, $J$ = 5.1 and 2.0 Hz).

**$^{13}$C{H} NMR** ($CDCl_3$, 75 MHz): δ 166.1, 143.0, 136.0, 131.7, 128.6, 128.3, 128.1, 123.1, 122.8, 84.2, 84.1, 66.3, 22.6.

**HRMS** (EI) m/z: $[M + Na]^+$ Calcd for $C_{19}H_{16}O_2Na$ 299.1043; Found 299.1043.

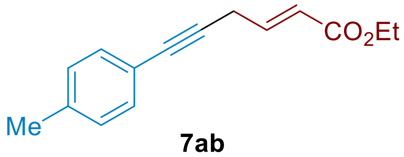


**7ab**

**Ethyl (*E*)-6-(*p*-tolyl)hex-2-en-5-ynoate** (**7ab**). The general procedure was followed using ethyl 2-diazobut-3-enoate (**1a**, 21.0 mg, 0.15 mmol) and trimethyl(p-tolylethynyl)silane (**6b**, 113.0 mg, 0.60 mmol). Final chromatographic purification(silica gel, hexanes/ethyl acetate 40:1) afforded compound **7ab** (26.0 mg, 76%) as a yellow oil.

**$^1$H NMR** ($CDCl_3$, 300 MHz): δ 7.35 (d, 2H, *J* = 8.0 Hz), 7.13 (d, 2H, *J* = 8.0 Hz), 7.01 (dt, 1H, *J* = 15.4 and 5.1 Hz), 6.24 (dt, 1H, *J* = 15.4 and 2.0 Hz), 4.23 (q, 2H, *J* = 7.1 Hz), 3.36 (dd, 2H, *J* = 5.1 and 2.0 Hz), 1.32 (t, 3H, *J* = 7.1 Hz), 2.37 (s, 3H).

**$^{13}$C{H} NMR** ($CDCl_3$, 75 MHz): δ 166.8, 142.9, 138.6, 131.9, 129.5, 123.4, 120.4, 84.5, 83.9, 60.9, 23.0, 21.9, 14.7.

**HRMS** (EI) m/z: $[M + Na]^+$ Calcd for $C_{15}H_{16}O_2Na$ 251.1043; Found 251.1045.

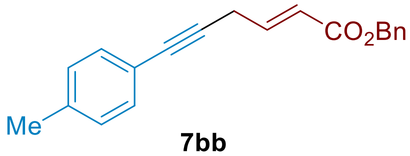


**7bb**

**Benzyl (*E*)-6-(*p*-tolyl)hex-2-en-5-ynoate** (**7bb**). The general procedure was followed using benzyl 2-diazobut-3-enoate (**1b**, 30.3 mg, 0.15 mmol) and trimethyl(p-tolylethynyl)silane (**6b**, 113.0 mg, 0.60 mmol). Final chromatographic purification (silica gel, hexanes/ethyl acetate 40:1) afforded compound **7bb** (25.3 mg, 58%) as a yellow oil.

**$^1$H NMR** ($CDCl_3$, 300 MHz): δ 7.41-7.33 (m, 7H), 7.13 (d, 2H, *J* = 7.9 Hz), 7.06 (dt, 1H, *J* = 15.4 and 5.1 Hz), 6.30 (dt, 1H, *J* = 15.5 and 2.0 Hz), 5.23 (s, 2H), 3.37 (dd, 2H, *J* = 5.1 and 2.0 Hz), 2.37 (s, 3H).

**$^{13}$C{H} NMR** ($CDCl_3$, 75 MHz): δ 166.1, 143.2, 138.2, 136.0, 131.5, 129.0, 128.6, 128.3, 122.7, 120.0, 84.2, 83.3, 66.3, 22.6, 21.4.

**HRMS** (EI) m/z: $[M + H]^+$ Calcd for $C_{20}H_{19}O_2$ 291.1380; Found 291.1380.

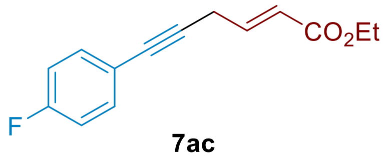

**7ac**

**Ethyl (*E*)-6-(4-fluorophenyl)hex-2-en-5-ynoate** (**7ac**). The general procedure was followed using ethyl 2-diazobut-3-enoate (**1a**, 21.0 mg, 0.15 mmol) and [(4-fluorophenyl)ethynyl]trimethylsilane (**6c**, 115.4 mg, 0.60 mmol). Final chromatographic purification (silica gel, hexanes/ethyl acetate 40:1) afforded compound **7ac** (22.0 mg, 63%) as a yellow oil.

**$^1$H NMR** ($CDCl_3$, 300 MHz): δ 7.48-7.39 (m, 2H), 7.07-6.95 (m, 3H), 6.22 (dt, 1H, *J* = 15.4 and 2.0 Hz), 4.23 (q, 2H, *J* = 7.1 Hz), 3.36 (dd, 2H, *J* = 5.1 and 2.0 Hz), 1.32 (t, 3H, *J* = 7.1 Hz).

**$^{13}$C{H} NMR** ($CDCl_3$, 75 MHz): δ 166.3, 162.4 ($J_{C-F}$= 247.5 Hz), 142.1, 133.5 ($J_{C-F}$ = 8.25 Hz), 123.1, 119.2 ($J_{C-F}$ = 3.75 Hz), 115.6 ($J_{C-F}$ = 21.75 Hz), 83.9, 82.9, 60.5, 22.4, 14.3.

**$^{19}$F NMR** ($CDCl_3$, 282 MHz): δ - 111.29.

**HRMS** (EI) m/z: $[M + H]^+$ Calcd for $C_{14}H_{14}FO_2$ 233.0972; Found 233.0976.

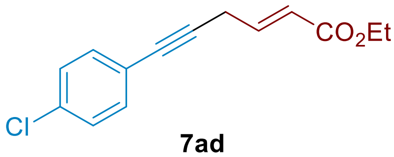

**7ad**

**Ethyl (*E*)-6-(4-chlorophenyl)hex-2-en-5-ynoate** (**7ad**). The general procedure was followed using ethyl 2-diazobut-3-enoate (**1a**, 21.0 mg, 0.15 mmol) and [(4-chlorophenyl)ethynyl]trimethylsilane (**6d**, 125.3 mg, 0.60 mmol). Final chromatographic purification (silica gel, hexanes/ethyl acetate 40:1) afforded skipped enyne **7ad** (21.3 mg, 57%) as a yellow oil.

**$^1$H NMR** ($CDCl_3$, 300 MHz): δ 7.38 (d, 2H, *J* = 8.6 Hz), 7.32-7.27 (m, 2H), 6.99 (dt, 1H, *J* = 15.5 and 5.1 Hz), 6.21 (dt, 1H, *J* = 15.4 and 2.0 Hz), 4.23 (q, 2H, *J* = 7.1 Hz), 3.36 (dd, 2H, *J* = 5.1 and 2.0 Hz), 1.32 (t, 3H, *J* = 7.1 Hz).

**$^{13}$C{H} NMR** ($CDCl_3$, 75 MHz): δ 166.7, 142.4, 134.5, 133.3, 129.0, 123.5, 122.0, 85.7, 83.4, 60.9, 22.9, 14.7.

**HRMS** (EI) m/z: $[M + H]^+$ Calcd for $C_{14}H_{14}ClO_2$ 249.0677; Found 249.0680.

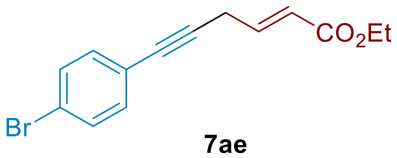

**7ae**

**Ethyl (*E*)-6-(4-bromophenyl)hex-2-en-5-ynoate** (**7ae**). The general procedure was followed using ethyl 2-diazobut-3-enoate (**1a**, 21.0 mg, 0.15 mmol) and [(4-bromophenyl)ethynyl]trimethylsilane (**6e**, 151.9 mg, 0.60 mmol). Final chromatographic purification (silica gel, hexanes/ethyl acetate 40:1) afforded compound **7ae** (25.9 mg, 59%) as a yellow oil.

**$^1$H NMR** ($CDCl_3$, 300 MHz): δ 7.46 (d, 2H, *J* = 8.6 Hz), 7.31 (d, 2H, *J* = 8.5 Hz), 6.99 (dt, 1H, *J* = 15.5, 5.1 Hz), 6.21 (dt, 1H, *J* = 15.5, 2.0 Hz), 4.23 (q, 2H, *J* = 7.1 Hz), 3.36 (dd, 2H, *J* = 5.1 and 2.0 Hz), 1.32 (t, 3H, *J* = 7.1 Hz).

**$^{13}$C{H} NMR** ($CDCl_3$, 75 MHz): δ 166.7, 142.4, 133.5, 131.9, 123.5, 122.7, 122.5, 85.9, 83.4, 60.9, 22.9, 14.7.

**HRMS** (EI) m/z: $[M + H]^+$ Calcd for $C_{14}H_{14}BrO_2$ 293.0172; Found 293.0176.

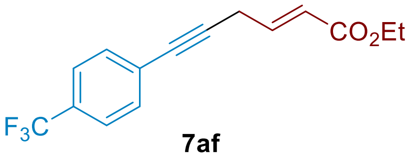

**7af**

**Ethyl (*E*)-6-(4-trifluormethylphenyl)hex-2-en-5-ynoate** (**7af**). The general procedure was followed using ethyl 2-diazobut-3-enoate (**1a**, 21.0 mg, 0.15 mmol) and trimethyl[(4-(trifluoromethyl)phenyl)ethynyl)silane (**6f**, 145.4 mg, 0.60 mmol). Final chromatographic purification (silica gel, hexanes/ethyl acetate 40:1) afforded compound **7af** (16.1 mg, 38%) as a yellow oil.

**$^1$H NMR** ($CDCl_3$, 300 MHz): δ 7.66-7.46 (m, 4H), 7.00 (dt, 1H, *J* = 15.5 and 5.2 Hz), 6.22 (dt, 1H, *J* = 15.5 Hz), 4.24 (q, 2H, *J* = 7.1 Hz), 3.39 (dd, 2H, *J* = 5.2 and 2.0 Hz), 1.33 (t, 3H, *J* = 7.1 Hz).

$^{13}$**C{H} NMR** ($CDCl_3$, 75 MHz): δ 166.2, 141.6, 131.9, 129.9 ($J_{C-F}$= 32.6 Hz), 126.9, 125.2 ($J_{C-F}$= 3.75 Hz), 123.8 ($J_{C-F}$= 288.0 Hz), 123.3, 87.0, 82.8, 60.5, 22.4, 14.3.

$^{19}$**F NMR** ($CDCl_3$, 282 MHz): δ - 62.8.

**HRMS** (EI) m/z: $[M + H]^+$ Calcd for $C_{15}H_{14}F_3O_2$ 283.0940; Found 283.0941.

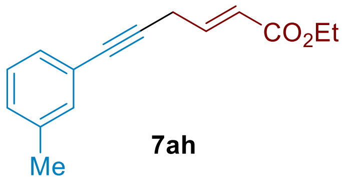


**7ah**

**Ethyl (*E*)-6-(*m*-tolyl)*hex*-2-en-5-ynoate** (**7ah**). The general procedure was followed using ethyl 2-diazobut-3-enoate (**1a**, 21.0 mg, 0.15 mmol) and trimethyl(m-tolylethynyl)silane (**6h**, 113.0 mg, 0.60 mmol). Final chromatographic purification (silica gel, hexanes/ethyl acetate 40:1) afforded compound **7ah** (16.4 mg, 48%) as a yellow oil.

$^{1}$**H NMR** ($CDCl_3$, 300 MHz): δ 7.29-7.12 (m, 4H), 7.05-6.96 (m, 1H), 6.25 (dd, 1H, *J* = 15.4 and 2.0 Hz), 4.23 (q, 2H, *J* = 7.1 Hz), *3*.37 (dd, 2H, *J* = 5.0 and 2.0 Hz), 2.35 (s, 3H), 1.32 (t, 3H, *J* = 7.1 Hz).

$^{13}$**C{H} NMR** ($CDCl_3$, 75 MHz): δ 166.8, 142.9, 138.4, 132.7, 129.4, 129.1, 128.6, 123.4, 123.3, 84.6, 84.2, 60.9, 22.9, 21.7, 14.7.

**HRMS** (EI) m/z: $[M + Na]^+$ Calcd for $C_{15}H_{16}O_2Na$ 251.1043, found 251.1046.

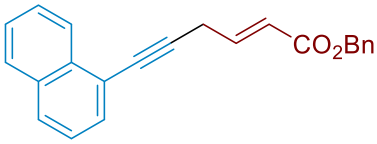


**7bi**

**Ethyl (*E*)-6-(naphthalen-1-yl)hex-2-en-5-ynoate** (**7bi**). The general procedure was followed using benzyl 2-diazobut-3-enoate (**1b**, 30.3 mg, 0.15 mmol) and trimethyl(naphthalen-1-ylethynyl)silane (**6i**, 134.6 mg, 0.60 mmol). Final chromatographic purification (silica gel, hexanes/ethyl acetate 40:1) afforded compound **7bi** (9.8 mg, 20%) as a yellow oil.

$^{1}$**H NMR** ($CDCl_3$, 300 MHz): δ 8.35-8.32 (m, 1H), 7.89-7.83 (m, 1H), 7.69 (d, 1H, *J* = 6.0 Hz), 7.61-7.53 (m, 2H), 7.47-7.35 (m, 7H), 7.15 (dt, 1H, *J* = 15.4 and 5.0 Hz), 6.39 (dt, 1H, *J* = 15.4 and 2.0 Hz), 5.23 (s, 2H), 3.55 (dd, 2H, *J* = 7.1 and 2.0 Hz).

**$^{13}$C{H} NMR** ($CDCl_3$, 75 MHz): δ 166.1, 143.0, 136.0, 133.4, 133.2, 130.5, 128.6, 128.3, 126.8, 126.4, 126.1, 125.2, 122.9, 120.7, 89.0, 82.2, 62.3, 22.9.

**HRMS** (EI) m/z: $[M + H]^+$ Calcd for $C_{23}H_{19}O_2$ 327.1380; Found 327.1383.

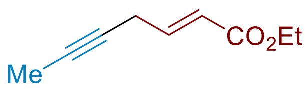


**7aj**

**Ethyl (*E*)-hept -2-en-5-ynoate** (**7aj**). The general procedure was followed using ethyl 2-diazobut-3-enoate (**1a**, 21.0 mg, 0.15 mmol) and trimethyl(prop-1-yn-1-yl)silane (**6j**, 67.3 mg, 0.60 mmol). Final chromatographic purification (silica gel, hexanes/ethyl acetate 40:1) afforded compound **7aj** (11 mg, 48%) as a colorless oil.

**$^1$H NMR** ($CDCl_3$, 300 MHz): δ 6.93 (dt, 1H, *J* = 15.4 and 5.1 Hz), 6.16 (dt, 1H, *J* = 15.4 and 2.0 Hz), 4.22 (q, 2H, *J* = 7.2 Hz), 3.09 (dt, 2H, *J* = 4.9 and 2.4 Hz), 1.85 (t, 3H, *J* = 2.5 Hz), 1.31 (t, 3H, *J* = 7.1 Hz).

**$^{13}$C{H} NMR** ($CDCl_3$, 75 MHz): δ 166.9, 143.7, 123.0, 80.0, 74,0, 60.8, 22.3, 14.7, 3.9.

**HRMS** (EI) m/z: $[M + Na]^+$ Calcd for $C_9H_{12}O_2Na$ 175.0730; Found 175.0731.

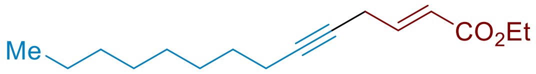


**7ak**

**Ethyl (*E*)-tetradec-2-en-5-ynoate** (**7ak**). The general procedure was followed using ethyl 2-diazobut-3-enoate (**1a**, 21.0 mg, 0.15 mmol) and dec-1-yn-1-yltrimethylsilane (**6k**, 126.3 mg, 0.60 mmol). Final chromatographic purification (silica gel, hexanes/ethyl acetate 40:1) afforded compound **7ak** (24.4 mg, 65%) as a colorless oil.

**$^1$H NMR** ($CDCl_3$, 300 MHz): δ 6.94 (dt, 1H, *J* = 15.4 and 5.0 Hz), 6.15 (dt, 1H, *J* = 15.4 and 2.0 Hz), 4.21 (q, 2H, *J* = 7.2 Hz), 3.12-3.09 (m, 2H), 2.23-2.17 (m, 2H), 1.52 (t, 3H, *J* = 7.3 Hz), 1.33-1.28 (m, 12H), 0.89 (t, 3H, *J* = 6.9 Hz).

**$^{13}$C{H} NMR** ($CDCl_3$, 75 MHz): δ 166.9, 143.8, 122.9, 84.8, 74.8, 60.7, 32.2, 29.6, 29.5, 29.3, 23.1, 22.4, 19.1, 14.7, 14.5.

**HRMS** (EI) m/z: $[M + H]^+$ Calcd for $C_{16}H_{27}O_2$ 251.2006; found: 251.2004.

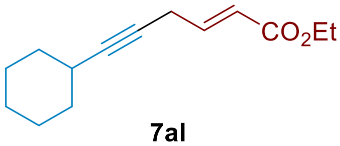

**7al**

**Ethyl (*E*)-6-cyclohexylhex-2-en-5-ynoate** (**7al**). The general procedure was followed using ethyl 2-diazobut-3-enoate (**1a**, 21.0 mg, 0.15 mmol) and (cyclohexylethynyl)trimethylsilane (**6l**, 108.2 mg, 0.60 mmol). Final chromatographic purification (silica gel, hexanes/ethyl acetate 40:1) afforded compound **7al** (21.1 mg, 64%) as a yellow oil.

**$^1$H NMR** ($CDCl_3$, 300 MHz): δ 6.95 (dt, 1H, *J* = 15.4 and 5.1 Hz), 6.15 (dt, 1H, *J* = 15.4 and 2.2 Hz), 4.22 (q, 2H, *J* = 7.1 Hz), 3.14-3.11 (m, 2H), 2.43-2.34 (m, 1H), 1.90-1.68 (m, 4H), 1.57-1.38 (m, 3H), 1.34-1.29 (m, 6H).

**$^{13}$C{H} NMR** ($CDCl_3$, 75 MHz): δ 166.9, 144.0, 122.9, 89.1, 74.6, 60.7, 33.3, 29.6, 26.3, 25.4, 22.4, 14.7.

**HRMS** (EI) m/z: $[M + H]^+$ Calcd for $C_{14}H_{21}O_2$ 221.1536, Found 221.1539.

## 11. Computational details

Geometry optimizations of the molecules were performed without symmetry constraints using the Gaussian09 (RevD.01)[9] suite of programs at the dispersion corrected B3LYP[10]–D3[11]/def2-SVP[12] level including solvent effects (solvent = dichloromethane) with the Polarization Continuum Model (PCM) method.[13] Reactants and adducts were characterized by frequency calculations, and have positive definite Hessian matrices. Transition states show only one negative eigenvalue in their diagonalized force constant matrices, and their associated eigenvectors were confirmed to correspond to the motion along the reaction coordinate under consideration using the Intrinsic Reaction Coordinate (IRC) method.[14]

We studied the process involving methyl 2-diazobut-3-enoate (**1a´**), alkene **2a** and $[Au(PhP^tBu_2)]^+$, as a model active catalysts where the biphenyl group in the phosphane ligand was replaced by a phenyl group. The corresponding computed reaction profiles are shown in Figures S4 and S5, which gather the corresponding relative free energies ($\Delta G_{298}$, at 298 K) in dichloromethane as solvent computed at the PCM-B3LYP-D3/def2-SVP level.

The process is suggested to begin with the thermal decomposition of the diazo-derivative leading to the corresponding gold(I)-vinyl carbene. Our calculations indicate that this initial step occurs stepwise from complex **INT1**, where the double bond of **1a´** is coordinated to the catalyst (Figure S4). From this intermediate, a migration of the transition metal fragment occurs first affording **INT2** via the transition state **TS1** with an activation barrier of 13.7 kcal/mol. Then, **INT2** is transformed into the key gold(I)-vinyl carbene **INT3** via **TS2**, a saddle point associated with the release of $N_2$ ($\Delta G^{\neq}$ = 9.1 kcal/mol). The computed low barrier for both steps and the overall exergonicity ($\Delta G_R$ = -21.7 kcal/mol) reflects the easiness of this initial reaction step, which is fully consistent with a process occurring at room temperature.

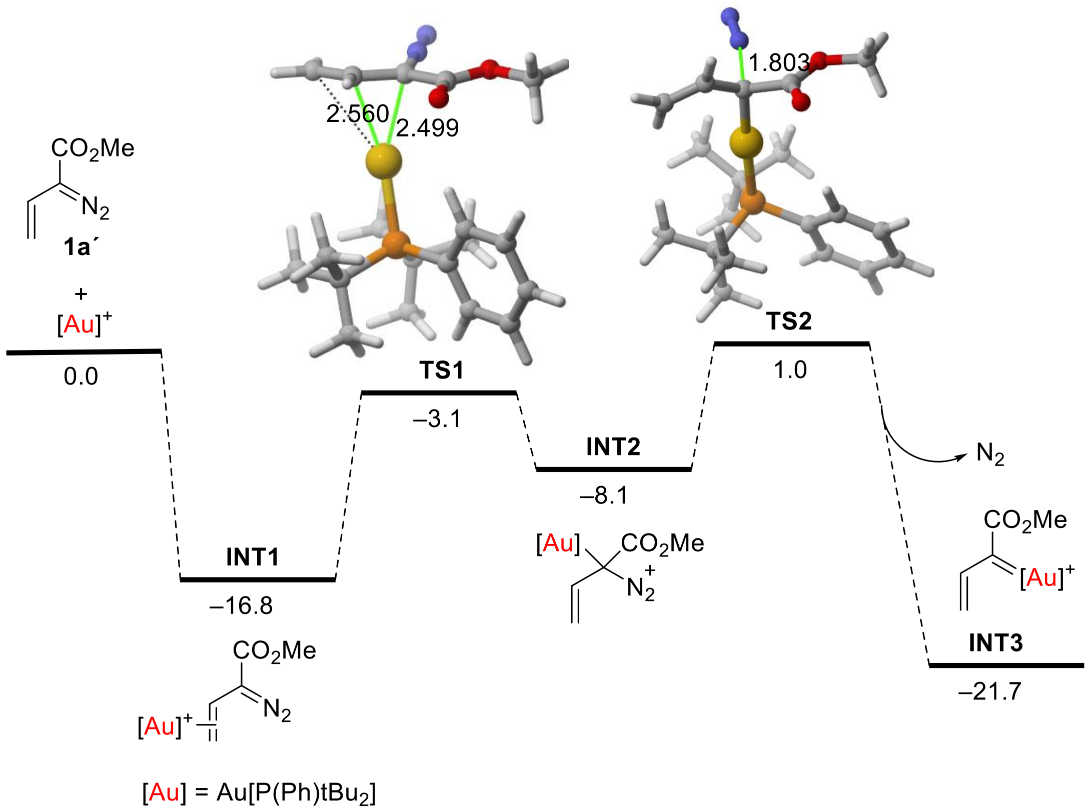


**Figure S4.** Computed reaction profile for the gold(I)-mediated decomposition of diazo compound **1a´**. Relative free energies (ΔG, at 298K) and bond distances are given in kcal/mol and angstroms, respectively. All data have been computed at the PCM(DCM)-B3LYP-D3/def2-SVP level.

Nucleophilic addition of the alkene **2a** should take place then at the vinylogous position of **INT3**, instead of at the carbenoid carbene atom. This regioselectivity is dictated by the large coefficient in the corresponding LUMO orbital and especially by the much more positive charge computed at the β-carbon atom (see inset in Figure S5). Two possible isomers can be formed in this reaction, namely **INT4** and **INT4'**. Not surprisingly, **INT4** is much more stable (ΔΔG = 14.9 kcal/mol) because the positive charge is strongly stabilized by π-conjugation from the adjacent phenyl group (i.e. benzylic position) and additionally by hyperconjugation from the TMS group, which is placed in β-relative position. Moreover, our calculations indicate that the formation of **INT4** can be considered as a barrierless reaction (Figure S6), which supports the widely documented enhanced vinylogous reactivity of gold(I)-vinyl carbenes. Once **INT4** is formed, this intermediate evolves into **INT5** via **TS3**, a saddle point associated with the intramolecular 1,4-migration of the TMS group. This unprecedented gold(I)-mediated 1,4-Si shift, which proceeds with a relatively low barrier of only 10.3 kcal/mol, produces the gold(I)-

vinylsilane **INT5** in a highly exergonic transformation ($\Delta G_R$ = -25.7 kcal/mol), which reflects the easiness of this silyl migration.

According to the experimental findings, the final 1,4-diene product incorporates a hydrogen atom at the position occupied by the TMS group in the vinylsilane intermediate. This hydrogen atom comes from the water traces present in the reaction, which is confirmed by deuterium labelling experiments. Therefore, we hypothesized that **INT5** should undergo a standard stepwise vinylsilane electrophilic substitution with replacement of the silyl group (i.e. *ipso* substitution) where a proton coming from water constitutes the electrophilic reagent. However, the electrophilicity of water is not high enough to promote such reaction as confirmed by the high barrier computed for the process involving the free vinylsilane **INT6** and a water molecule ($\Delta G^{\neq}$ > 50 kcal/mol). Alternatively, the electrophilicity of water can be substantially enhanced by the coordination of the oxygen atom to the Au(I)-catalyst. Indeed, the computed barrier for the analogous process involving $H_2O$-[Au]$^+$ as a proton source is much lower ($\Delta G^{\neq}$ = 23.2 kcal/mol). As expected, this step affords the carbocationic intermediate **INT7**, again stabilized by Si-hyperconjugation, which is finally transformed into the final 1,4-diene **3a´** by a base-mediated silyl displacement. The high exergonicity computed for this final reaction step ($\Delta G_R$ = -34.7 kcal/mol), drives the entire *ipso*-substitution forward. This final step also releases TMSOH and the active gold(I)-catalyst, the latter entering into a new catalytic cycle.

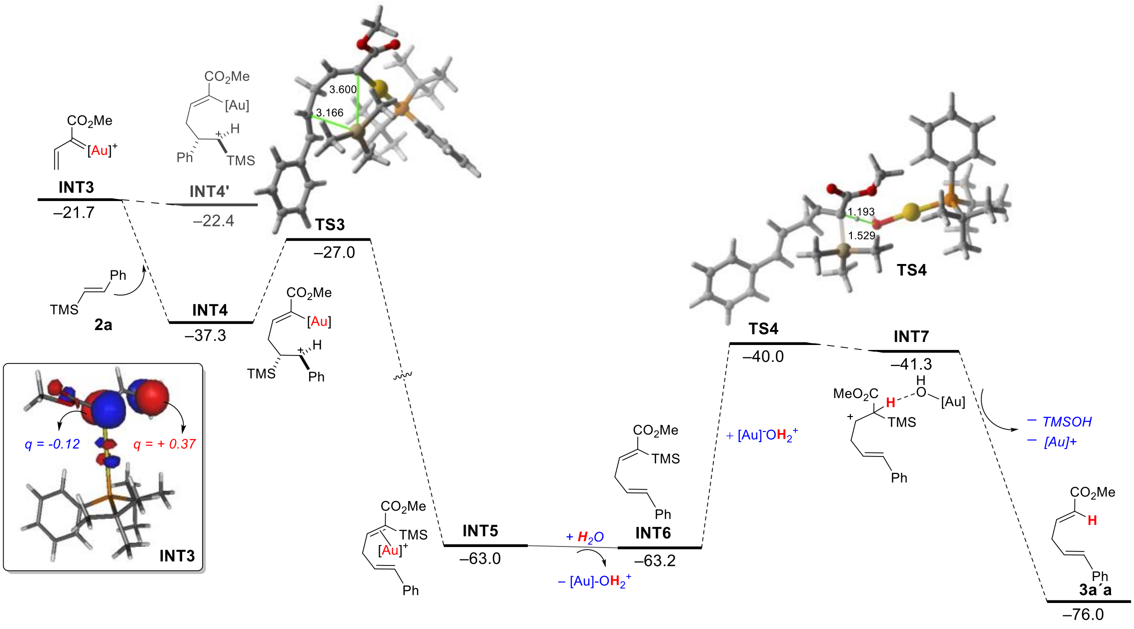


**Figure S5.** Computed reaction profile for the evolution of the key gold(I)-vinylcarbene **INT3**. Relative free energies ($\Delta G$, at 298K) and bond distances are given in kcal/mol and

angstroms, respectively. All data have been computed at the PCM(DCM)-B3LYP-D3/def2-SVP level.

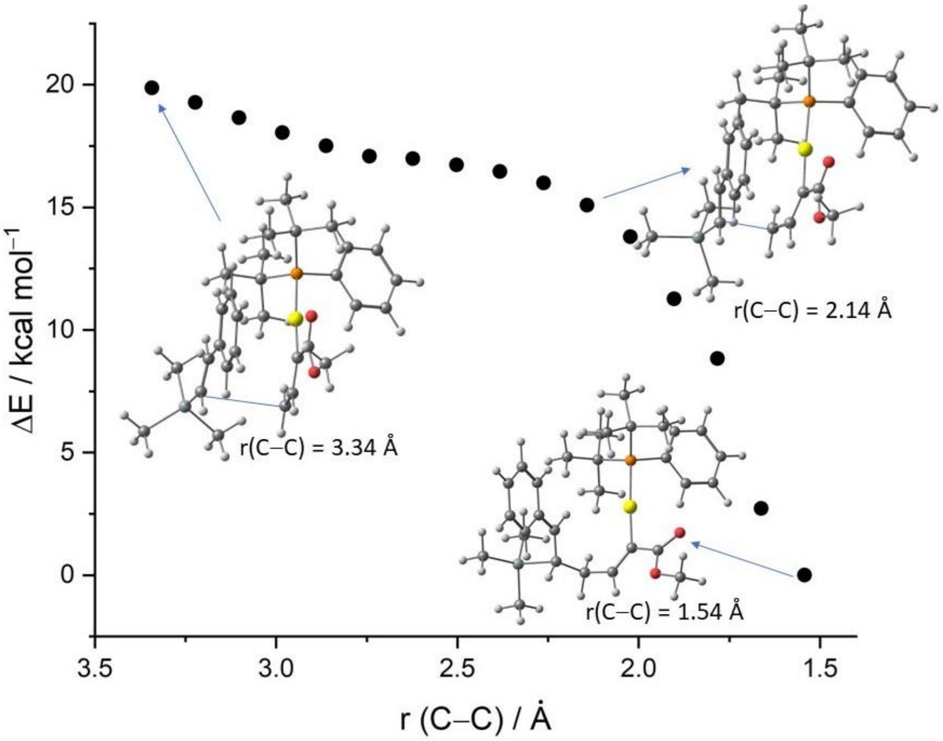


**Figure S6**. Relaxed scans showing the barrierless transformation from **INT3** + alkene **2a** into **INT4**.

For completeness, we were curious to investigate the key 1,4-TMS migration in the analogous process involving the corresponding alkyne (PhC≡CTMS, **6a**), which affords the respective 1,4-enyne. Our calculations indicate that the 1,4-silyl migration occurs similarly, that is, in a concerted intramolecular manner through the transition state **TS3-b** (Figure S7). Interestingly, this process is kinetically more difficult than that involving the alkene-derived intermediate **INT4** ($\Delta\Delta G^{\neq}$ = 6.7 kcal/mol). This can be ascribed to the higher bond strength of the breaking C-Si bond in **INT4-b** with respect to **INT4**, as confirmed by the corresponding Wiberg bond indices (0.48 and 0.42, respectively). Despite that, the computed relatively low barrier ($\Delta G^{\neq}$ = 17.0 kcal/mol) and exergonicity ($\Delta G_R$ = -17.2 kcal/mol) indicate that this 1,4-Si shift is also a feasible process at room temperature.

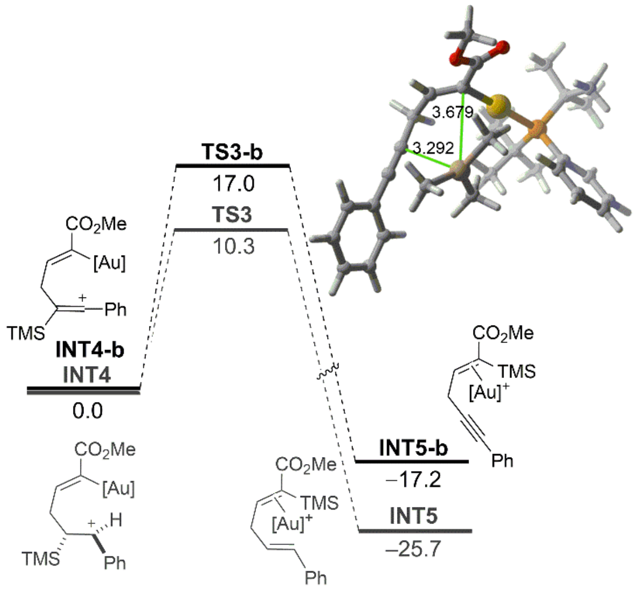


**Figure S7.** Comparison of the key 1,4-silyl migration involving in the alkene (**INT4**) and alkyne (**INT4b**) derived intermediates. Relative free energies (ΔG, at 298K) and bond distances are given in kcal/mol and angstroms, respectively. All data have been computed at the PCM(DCM)-B3LYP-D3/def2-SVP level.

Cartesian coordinates (in Å) and energies (in a.u.) of all the stationary points discussed in the text. All calculations have been performed at the PCM(dichloromethane)-B3LYP-D3/def2-SVP level.

**1a′**, E = -453.600343

| | | | |
|---|---|---|---|
| C | 0.628284000 | -0.061816000 | 0.000013000 |
| C | 1.745459000 | -1.003301000 | 0.000044000 |
| C | 3.056000000 | -0.712660000 | -0.000022000 |
| H | 1.400622000 | -2.041357000 | 0.000115000 |
| H | 3.800311000 | -1.511431000 | 0.000010000 |
| H | 3.427920000 | 0.316776000 | -0.000116000 |
| C | -0.771634000 | -0.514706000 | -0.000027000 |
| O | -1.639286000 | 0.511762000 | -0.000088000 |
| O | -1.096716000 | -1.683612000 | -0.000013000 |
| C | -3.030590000 | 0.172707000 | 0.000046000 |
| H | -3.288236000 | -0.413555000 | 0.895004000 |
| H | -3.574936000 | 1.124424000 | 0.000025000 |
| H | -3.288390000 | -0.413689000 | -0.894778000 |
| N | 0.855565000 | 1.234553000 | 0.000013000 |
| N | 1.093807000 | 2.341489000 | 0.000021000 |

**INT1**, E = -1477.230240

| | | | |
|---|---|---|---|
| Au | 0.133139000 | -1.355657000 | -0.354285000 |
| C | 3.250330000 | -0.723521000 | -0.039731000 |
| C | 2.503272000 | -1.561898000 | -0.944046000 |

| | | | |
|---|---|---|---|
| C | 1.902048000 | -2.782029000 | -0.686188000 |
| H | 2.481717000 | -1.149556000 | -1.958360000 |
| H | 1.565619000 | -3.390783000 | -1.529609000 |
| H | 2.049963000 | -3.299877000 | 0.267006000 |
| C | 3.584574000 | 0.673706000 | -0.393317000 |
| O | 4.241874000 | 1.295268000 | 0.584556000 |
| O | 3.258517000 | 1.172647000 | -1.449024000 |
| C | 4.557164000 | 2.682729000 | 0.377425000 |
| H | 3.632253000 | 3.274731000 | 0.313845000 |
| H | 5.147062000 | 2.989542000 | 1.248180000 |
| H | 5.137160000 | 2.812294000 | -0.547222000 |
| P | -1.669569000 | 0.076255000 | 0.118020000 |
| C | -2.216396000 | -0.253400000 | 1.916203000 |
| C | -1.087883000 | 1.816235000 | -0.018586000 |
| C | -1.831665000 | 2.896106000 | 0.494844000 |
| C | 0.129172000 | 2.086942000 | -0.670460000 |
| C | -1.360259000 | 4.205204000 | 0.371314000 |
| C | 0.596442000 | 3.398219000 | -0.797012000 |
| C | -0.144690000 | 4.460662000 | -0.272960000 |
| H | -1.948868000 | 5.029021000 | 0.781484000 |
| H | 1.546679000 | 3.574647000 | -1.305828000 |
| H | 0.221108000 | 5.486017000 | -0.366103000 |
| H | -2.784219000 | 2.729581000 | 0.996171000 |
| C | -1.135730000 | 0.376945000 | 2.817602000 |
| H | -1.146926000 | 1.474942000 | 2.778877000 |
| H | -1.322413000 | 0.071565000 | 3.859817000 |
| H | -0.125582000 | 0.031899000 | 2.543607000 |
| C | -3.603302000 | 0.306309000 | 2.274732000 |
| H | -3.789857000 | 0.112350000 | 3.343501000 |
| H | -3.685148000 | 1.390818000 | 2.126011000 |
| H | -4.406163000 | -0.186545000 | 1.709599000 |
| C | -2.222264000 | -1.780388000 | 2.146980000 |
| H | -2.535901000 | -1.975345000 | 3.185221000 |
| H | -2.920753000 | -2.309487000 | 1.485772000 |
| H | -1.221453000 | -2.220324000 | 2.015217000 |
| C | -3.045215000 | -0.117893000 | -1.179459000 |
| C | -2.351095000 | 0.003981000 | -2.552449000 |
| H | -3.117294000 | -0.060706000 | -3.341886000 |
| H | -1.833742000 | 0.968718000 | -2.667777000 |
| H | -1.623056000 | -0.803764000 | -2.723561000 |
| C | -4.123084000 | 0.975140000 | -1.073017000 |
| H | -4.874853000 | 0.798789000 | -1.859689000 |
| H | -4.649589000 | 0.966962000 | -0.110580000 |
| H | -3.706276000 | 1.978855000 | -1.237297000 |
| C | -3.673654000 | -1.514430000 | -1.037883000 |
| H | -2.912982000 | -2.311441000 | -1.042131000 |
| H | -4.275081000 | -1.608670000 | -0.122494000 |
| H | -4.346929000 | -1.690395000 | -1.892291000 |
| H | 0.733262000 | 1.280814000 | -1.089715000 |
| N | 3.523218000 | -1.146513000 | 1.184985000 |
| N | 3.743659000 | -1.517287000 | 2.225052000 |

**TS1**, E = -1477.210200

| | | | |
|---|---|---|---|
| Au | 0.717841000 | -0.739441000 | -0.141587000 |
| C | 3.145136000 | -0.144920000 | -0.142008000 |
| C | 3.031575000 | -1.091576000 | -1.273026000 |
| C | 3.126256000 | -2.436514000 | -1.221581000 |
| H | 2.872642000 | -0.574749000 | -2.224135000 |
| H | 3.036846000 | -3.024653000 | -2.136185000 |
| H | 3.302277000 | -2.976496000 | -0.286939000 |

| | | | |
|---|---|---|---|
| C | 3.015441000 | 1.324819000 | -0.346418000 |
| O | 3.353587000 | 2.011024000 | 0.743018000 |
| O | 2.608495000 | 1.804162000 | -1.381862000 |
| C | 3.194009000 | 3.439601000 | 0.686321000 |
| H | 2.131103000 | 3.696808000 | 0.567743000 |
| H | 3.575413000 | 3.821373000 | 1.639720000 |
| H | 3.766134000 | 3.857041000 | -0.154047000 |
| P | -1.555171000 | -0.397228000 | 0.097913000 |
| C | -2.070621000 | -0.996788000 | 1.835442000 |
| C | -1.891986000 | 1.404742000 | -0.045271000 |
| C | -3.142822000 | 1.946073000 | 0.309155000 |
| C | -0.899685000 | 2.267180000 | -0.547767000 |
| C | -3.387403000 | 3.314434000 | 0.174421000 |
| C | -1.151507000 | 3.634990000 | -0.687308000 |
| C | -2.393118000 | 4.162942000 | -0.323418000 |
| H | -4.362395000 | 3.716667000 | 0.458718000 |
| H | -0.367734000 | 4.285889000 | -1.081647000 |
| H | -2.587693000 | 5.232883000 | -0.428355000 |
| H | -3.939376000 | 1.309001000 | 0.690774000 |
| C | -1.567496000 | 0.070818000 | 2.826711000 |
| H | -2.112061000 | 1.020062000 | 2.728782000 |
| H | -1.717233000 | -0.302995000 | 3.852308000 |
| H | -0.492264000 | 0.272751000 | 2.697035000 |
| C | -3.586546000 | -1.205113000 | 2.001538000 |
| H | -3.780104000 | -1.497683000 | 3.046126000 |
| H | -4.171976000 | -0.296452000 | 1.810237000 |
| H | -3.968695000 | -2.012449000 | 1.361654000 |
| C | -1.350437000 | -2.330507000 | 2.128712000 |
| H | -1.650699000 | -2.673627000 | 3.131714000 |
| H | -1.612345000 | -3.122829000 | 1.415056000 |
| H | -0.255851000 | -2.215081000 | 2.130374000 |
| C | -2.476248000 | -1.220933000 | -1.349003000 |
| C | -1.763905000 | -0.752738000 | -2.634665000 |
| H | -2.308593000 | -1.155455000 | -3.503770000 |
| H | -1.752394000 | 0.344198000 | -2.725124000 |
| H | -0.727144000 | -1.118183000 | -2.688532000 |
| C | -3.953986000 | -0.795571000 | -1.421045000 |
| H | -4.421117000 | -1.322242000 | -2.268916000 |
| H | -4.523717000 | -1.055399000 | -0.520291000 |
| H | -4.059527000 | 0.282339000 | -1.606069000 |
| C | -2.355039000 | -2.747592000 | -1.216740000 |
| H | -1.306883000 | -3.066302000 | -1.103530000 |
| H | -2.937478000 | -3.139746000 | -0.371231000 |
| H | -2.749394000 | -3.215509000 | -2.132988000 |
| H | 0.080823000 | 1.884414000 | -0.838398000 |
| N | 3.695199000 | -0.545583000 | 1.003784000 |
| N | 4.137831000 | -0.905973000 | 1.973802000 |

**INT2,** E = -1477.216680

| | | | |
|---|---|---|---|
| Au | 0.785869000 | -0.365715000 | -0.067210000 |
| C | 3.009342000 | -0.078119000 | -0.340358000 |
| C | 3.306535000 | -0.582993000 | -1.715016000 |
| C | 3.822734000 | -1.770952000 | -2.042888000 |
| H | 3.034382000 | 0.157283000 | -2.472025000 |
| H | 3.987629000 | -2.025476000 | -3.091679000 |
| H | 4.100221000 | -2.523341000 | -1.298592000 |
| C | 3.065736000 | 1.422424000 | -0.118893000 |
| O | 3.259369000 | 1.721263000 | 1.161776000 |
| O | 2.904274000 | 2.209926000 | -1.016686000 |
| C | 3.248260000 | 3.118698000 | 1.512948000 |

| | | | |
|---|---|---|---|
| H | 2.267962000 | 3.557375000 | 1.277038000 |
| H | 3.438309000 | 3.156843000 | 2.590965000 |
| H | 4.032772000 | 3.655887000 | 0.961921000 |
| P | -1.539634000 | -0.493238000 | 0.124292000 |
| C | -1.959134000 | -0.979647000 | 1.922885000 |
| C | -2.276817000 | 1.152883000 | -0.224625000 |
| C | -3.640546000 | 1.417001000 | 0.008891000 |
| C | -1.471960000 | 2.177253000 | -0.756253000 |
| C | -4.179692000 | 2.672281000 | -0.279931000 |
| C | -2.016745000 | 3.430461000 | -1.050596000 |
| C | -3.370417000 | 3.681600000 | -0.812209000 |
| H | -5.238939000 | 2.859376000 | -0.089420000 |
| H | -1.375863000 | 4.211987000 | -1.465077000 |
| H | -3.795428000 | 4.662197000 | -1.039198000 |
| H | -4.296783000 | 0.649091000 | 0.416088000 |
| C | -1.734799000 | 0.288118000 | 2.771467000 |
| H | -2.481562000 | 1.066676000 | 2.561910000 |
| H | -1.815123000 | 0.017881000 | 3.836653000 |
| H | -0.732636000 | 0.717094000 | 2.609529000 |
| C | -3.394508000 | -1.498942000 | 2.117298000 |
| H | -3.549624000 | -1.689026000 | 3.191757000 |
| H | -4.160909000 | -0.776964000 | 1.806624000 |
| H | -3.569547000 | -2.447678000 | 1.591469000 |
| C | -0.967528000 | -2.069240000 | 2.383924000 |
| H | -1.223856000 | -2.359637000 | 3.415495000 |
| H | -1.009392000 | -2.974926000 | 1.764489000 |
| H | 0.069912000 | -1.702542000 | 2.391080000 |
| C | -2.214100000 | -1.672478000 | -1.208355000 |
| C | -1.587678000 | -1.210105000 | -2.540652000 |
| H | -2.002168000 | -1.823961000 | -3.356508000 |
| H | -1.819075000 | -0.157009000 | -2.762926000 |
| H | -0.494221000 | -1.336001000 | -2.548103000 |
| C | -3.746666000 | -1.618607000 | -1.336954000 |
| H | -4.054071000 | -2.347550000 | -2.104331000 |
| H | -4.265412000 | -1.883231000 | -0.407185000 |
| H | -4.094803000 | -0.629960000 | -1.666282000 |
| C | -1.750803000 | -3.104226000 | -0.890624000 |
| H | -0.661909000 | -3.158613000 | -0.735794000 |
| H | -2.253791000 | -3.518417000 | -0.005451000 |
| H | -1.999457000 | -3.755060000 | -1.744367000 |
| H | -0.409533000 | 2.004130000 | -0.941416000 |
| N | 3.579424000 | -0.791802000 | 0.677941000 |
| N | 4.024479000 | -1.410821000 | 1.493132000 |

**TS2**, E = -1477.201496

| | | | |
|---|---|---|---|
| Au | 0.798373000 | -0.379910000 | -0.090442000 |
| C | 2.900584000 | -0.188067000 | -0.297739000 |
| C | 3.706935000 | -1.063646000 | -1.166181000 |
| C | 3.264014000 | -2.252644000 | -1.606676000 |
| H | 4.697533000 | -0.701550000 | -1.461555000 |
| H | 3.881869000 | -2.873709000 | -2.260822000 |
| H | 2.276159000 | -2.634542000 | -1.331634000 |
| C | 3.223833000 | 1.278939000 | -0.370169000 |
| O | 3.234637000 | 1.905009000 | 0.800171000 |
| O | 3.353070000 | 1.799830000 | -1.453974000 |
| C | 3.400836000 | 3.335159000 | 0.770954000 |
| H | 2.547201000 | 3.802388000 | 0.258651000 |
| H | 3.441244000 | 3.653345000 | 1.818310000 |
| H | 4.330647000 | 3.601778000 | 0.249096000 |
| P | -1.558227000 | -0.466656000 | 0.054697000 |

| | | | |
|---|---|---|---|
| C | -2.017235000 | -1.236411000 | 1.739965000 |
| C | -2.257652000 | 1.230754000 | -0.032313000 |
| C | -3.594539000 | 1.517328000 | 0.303702000 |
| C | -1.438471000 | 2.279506000 | -0.489894000 |
| C | -4.091457000 | 2.818038000 | 0.194268000 |
| C | -1.940550000 | 3.578856000 | -0.606458000 |
| C | -3.266945000 | 3.852025000 | -0.261793000 |
| H | -5.130472000 | 3.021384000 | 0.463625000 |
| H | -1.288815000 | 4.378646000 | -0.965755000 |
| H | -3.658950000 | 4.868217000 | -0.348179000 |
| H | -4.263293000 | 0.731038000 | 0.651493000 |
| C | -1.752362000 | -0.141387000 | 2.793133000 |
| H | -2.470397000 | 0.687327000 | 2.721690000 |
| H | -1.844450000 | -0.585277000 | 3.797593000 |
| H | -0.735889000 | 0.274508000 | 2.700842000 |
| C | -3.467381000 | -1.736736000 | 1.846842000 |
| H | -3.634368000 | -2.102380000 | 2.873134000 |
| H | -4.210544000 | -0.950519000 | 1.661348000 |
| H | -3.667297000 | -2.575691000 | 1.166154000 |
| C | -1.063885000 | -2.421568000 | 2.008439000 |
| H | -1.322616000 | -2.864092000 | 2.984022000 |
| H | -1.147609000 | -3.213708000 | 1.252634000 |
| H | -0.011672000 | -2.102246000 | 2.057511000 |
| C | -2.263387000 | -1.381162000 | -1.457392000 |
| C | -1.625871000 | -0.704483000 | -2.689619000 |
| H | -2.019799000 | -1.183806000 | -3.600497000 |
| H | -1.869671000 | 0.367486000 | -2.743360000 |
| H | -0.530267000 | -0.811328000 | -2.698776000 |
| C | -3.793831000 | -1.275429000 | -1.571996000 |
| H | -4.112475000 | -1.821001000 | -2.475356000 |
| H | -4.321049000 | -1.720330000 | -0.718748000 |
| H | -4.125442000 | -0.233542000 | -1.684611000 |
| C | -1.827943000 | -2.855359000 | -1.400228000 |
| H | -0.738851000 | -2.957474000 | -1.270315000 |
| H | -2.331268000 | -3.407986000 | -0.594273000 |
| H | -2.097830000 | -3.343900000 | -2.350560000 |
| H | -0.397996000 | 2.083847000 | -0.760080000 |
| N | 3.470210000 | -0.609937000 | 1.360191000 |
| N | 3.642431000 | -1.077937000 | 2.342985000 |

**INT3**, E = -1367.787508

| | | | |
|---|---|---|---|
| Au | 0.959708000 | -0.327921000 | -0.174899000 |
| C | 2.987486000 | -0.254209000 | -0.406406000 |
| C | 3.869603000 | -1.244641000 | -0.916792000 |
| C | 3.408352000 | -2.477527000 | -1.277461000 |
| H | 4.942159000 | -1.031287000 | -0.990580000 |
| H | 4.091768000 | -3.262169000 | -1.615263000 |
| H | 2.341308000 | -2.715773000 | -1.227260000 |
| C | 3.603754000 | 1.050348000 | -0.057864000 |
| O | 3.701194000 | 1.286703000 | 1.235690000 |
| O | 3.907319000 | 1.798969000 | -0.962012000 |
| C | 4.240229000 | 2.566546000 | 1.634324000 |
| H | 3.588344000 | 3.375918000 | 1.276348000 |
| H | 4.268429000 | 2.549434000 | 2.728893000 |
| H | 5.250789000 | 2.698762000 | 1.223045000 |
| P | -1.403553000 | -0.412086000 | 0.193154000 |
| C | -1.692170000 | -0.653758000 | 2.064967000 |
| C | -2.209753000 | 1.160952000 | -0.307104000 |
| C | -3.562766000 | 1.434446000 | -0.027592000 |
| C | -1.457979000 | 2.124969000 | -1.003316000 |

| | | | |
|---|---|---|---|
| C | -4.142722000 | 2.638802000 | -0.431791000 |
| C | -2.042292000 | 3.327610000 | -1.411738000 |
| C | -3.384970000 | 3.588067000 | -1.125967000 |
| H | -5.193056000 | 2.833864000 | -0.203243000 |
| H | -1.441576000 | 4.062753000 | -1.952260000 |
| H | -3.841354000 | 4.529176000 | -1.441978000 |
| H | -4.178333000 | 0.712114000 | 0.506625000 |
| C | -1.418208000 | 0.718307000 | 2.713382000 |
| H | -2.191248000 | 1.458617000 | 2.463640000 |
| H | -1.405368000 | 0.596215000 | 3.808632000 |
| H | -0.439819000 | 1.124523000 | 2.409195000 |
| C | -3.100410000 | -1.144902000 | 2.437673000 |
| H | -3.174488000 | -1.189780000 | 3.536644000 |
| H | -3.896472000 | -0.473715000 | 2.089331000 |
| H | -3.299612000 | -2.155874000 | 2.056156000 |
| C | -0.654145000 | -1.666220000 | 2.595218000 |
| H | -0.815770000 | -1.799080000 | 3.677259000 |
| H | -0.743173000 | -2.653841000 | 2.123629000 |
| H | 0.377693000 | -1.308786000 | 2.455763000 |
| C | -2.158230000 | -1.766379000 | -0.911006000 |
| C | -1.658207000 | -1.460595000 | -2.339442000 |
| H | -2.097846000 | -2.194603000 | -3.034340000 |
| H | -1.962672000 | -0.457426000 | -2.675633000 |
| H | -0.562807000 | -1.535452000 | -2.421759000 |
| C | -3.696217000 | -1.778637000 | -0.923047000 |
| H | -4.031034000 | -2.599120000 | -1.578810000 |
| H | -4.133374000 | -1.954290000 | 0.067912000 |
| H | -4.110050000 | -0.846272000 | -1.331344000 |
| C | -1.616761000 | -3.133852000 | -0.458341000 |
| H | -0.517884000 | -3.137110000 | -0.380394000 |
| H | -2.037577000 | -3.448907000 | 0.507396000 |
| H | -1.901120000 | -3.894209000 | -1.203704000 |
| H | -0.405138000 | 1.942053000 | -1.229803000 |

**INT4,** E = -2085.572492

| | | | |
|---|---|---|---|
| Au | 1.068049000 | 0.767180000 | -0.397480000 |
| C | -0.449360000 | 2.192971000 | -0.375018000 |
| C | -1.757404000 | 1.972269000 | -0.628281000 |
| C | -2.354363000 | 0.658467000 | -1.049497000 |
| C | -3.624564000 | 0.287356000 | -0.247401000 |
| C | -4.592138000 | -0.526899000 | -0.850508000 |
| H | -2.473535000 | 2.802595000 | -0.558632000 |
| H | -1.589973000 | -0.133810000 | -1.012062000 |
| H | -2.649829000 | 0.739839000 | -2.110252000 |
| H | -4.008272000 | 1.090223000 | 0.399463000 |
| H | -4.230894000 | -1.204080000 | -1.635474000 |
| C | -0.027801000 | 3.566262000 | 0.028215000 |
| O | -0.892111000 | 4.545379000 | -0.319013000 |
| O | 1.006627000 | 3.816066000 | 0.621865000 |
| C | -0.546162000 | 5.873808000 | 0.076227000 |
| H | 0.419938000 | 6.173290000 | -0.358604000 |
| H | -1.346820000 | 6.524881000 | -0.296459000 |
| H | -0.472438000 | 5.952572000 | 1.172152000 |
| Si | -3.063474000 | -1.040791000 | 1.294598000 |
| C | -4.551247000 | -1.348091000 | 2.382580000 |
| H | -5.332788000 | -1.941825000 | 1.887507000 |
| H | -4.196810000 | -1.915449000 | 3.260307000 |
| H | -4.994537000 | -0.409785000 | 2.749738000 |
| C | -1.762714000 | -0.040915000 | 2.186278000 |
| H | -2.180685000 | 0.901910000 | 2.569634000 |

| | | | |
|---|---|---|---|
| H | -1.427704000 | -0.649442000 | 3.044042000 |
| H | -0.893970000 | 0.192809000 | 1.556816000 |
| C | -2.429116000 | -2.602661000 | 0.484250000 |
| H | -1.973904000 | -3.236528000 | 1.262843000 |
| H | -3.247703000 | -3.172051000 | 0.017689000 |
| H | -1.656986000 | -2.403780000 | -0.272498000 |
| C | -5.985467000 | -0.645356000 | -0.539309000 |
| C | -6.708415000 | -1.730048000 | -1.099729000 |
| C | -6.663077000 | 0.272170000 | 0.306200000 |
| C | -8.055566000 | -1.907077000 | -0.803306000 |
| C | -8.009080000 | 0.092738000 | 0.591409000 |
| C | -8.704182000 | -0.997985000 | 0.042764000 |
| H | -6.193980000 | -2.433322000 | -1.759001000 |
| H | -6.134264000 | 1.131164000 | 0.721455000 |
| H | -8.605656000 | -2.747302000 | -1.231063000 |
| H | -8.529842000 | 0.802706000 | 1.236699000 |
| H | -9.764089000 | -1.132455000 | 0.271398000 |
| P | 2.808539000 | -0.864971000 | -0.429528000 |
| C | 3.788609000 | -0.689455000 | -2.061793000 |
| C | 3.990667000 | -0.626543000 | 0.962406000 |
| C | 5.225003000 | -1.298008000 | 1.051735000 |
| C | 3.630646000 | 0.254656000 | 1.998510000 |
| C | 6.073212000 | -1.089909000 | 2.141935000 |
| C | 4.476642000 | 0.455902000 | 3.093594000 |
| C | 5.700764000 | -0.214005000 | 3.167417000 |
| H | 7.028839000 | -1.617571000 | 2.189832000 |
| H | 4.177212000 | 1.144615000 | 3.887236000 |
| H | 6.365012000 | -0.053569000 | 4.020203000 |
| H | 5.537783000 | -1.990260000 | 0.271304000 |
| C | 4.658666000 | 0.572575000 | -1.888238000 |
| H | 5.475677000 | 0.417966000 | -1.169102000 |
| H | 5.107441000 | 0.834878000 | -2.860485000 |
| H | 4.060948000 | 1.434400000 | -1.549779000 |
| C | 4.671905000 | -1.887756000 | -2.446768000 |
| H | 5.230421000 | -1.631691000 | -3.362531000 |
| H | 5.414663000 | -2.140876000 | -1.679053000 |
| H | 4.078409000 | -2.785640000 | -2.669903000 |
| C | 2.779748000 | -0.436557000 | -3.203186000 |
| H | 3.338683000 | -0.297574000 | -4.143306000 |
| H | 2.088212000 | -1.277189000 | -3.350918000 |
| H | 2.182944000 | 0.471098000 | -3.029522000 |
| C | 2.078870000 | -2.604158000 | -0.151915000 |
| C | 1.240102000 | -2.495837000 | 1.139084000 |
| H | 0.787958000 | -3.477410000 | 1.357151000 |
| H | 1.856423000 | -2.209673000 | 2.005128000 |
| H | 0.426338000 | -1.763936000 | 1.041885000 |
| C | 3.125463000 | -3.713897000 | 0.040826000 |
| H | 2.598971000 | -4.670685000 | 0.195079000 |
| H | 3.783489000 | -3.838375000 | -0.827950000 |
| H | 3.748176000 | -3.536696000 | 0.929022000 |
| C | 1.154891000 | -2.939844000 | -1.336045000 |
| H | 0.451355000 | -2.119218000 | -1.549588000 |
| H | 1.720799000 | -3.156979000 | -2.253609000 |
| H | 0.565015000 | -3.838923000 | -1.092919000 |
| H | 2.681366000 | 0.792942000 | 1.948114000 |

**TS3**, E = -2085.559504

| | | | |
|---|---|---|---|
| Au | 0.828528000 | 0.912525000 | -0.520692000 |
| C | -0.510406000 | 2.504091000 | -0.627323000 |
| C | -1.680384000 | 2.522818000 | -1.304847000 |

| | | | |
|---|---|---|---|
| C | -2.203528000 | 1.368531000 | -2.119848000 |
| C | -3.315428000 | 0.610629000 | -1.444240000 |
| C | -3.289431000 | -0.734362000 | -1.271382000 |
| H | -2.315819000 | 3.417925000 | -1.279519000 |
| H | -1.382470000 | 0.679905000 | -2.370127000 |
| H | -2.605623000 | 1.758489000 | -3.074240000 |
| H | -4.224063000 | 1.178202000 | -1.212997000 |
| H | -2.356484000 | -1.250382000 | -1.522849000 |
| C | -0.090853000 | 3.732120000 | 0.111385000 |
| O | -1.082539000 | 4.627344000 | 0.326070000 |
| O | 1.037852000 | 3.930839000 | 0.521970000 |
| C | -0.723268000 | 5.828636000 | 1.013783000 |
| H | 0.046260000 | 6.383608000 | 0.456168000 |
| H | -1.640778000 | 6.424911000 | 1.090127000 |
| H | -0.332112000 | 5.600303000 | 2.017205000 |
| Si | -2.600723000 | 0.646772000 | 1.640184000 |
| C | -4.423003000 | 0.910686000 | 1.750506000 |
| H | -5.008731000 | 0.125827000 | 1.257015000 |
| H | -4.654911000 | 0.888962000 | 2.833052000 |
| H | -4.707191000 | 1.903229000 | 1.369946000 |
| C | -1.589311000 | 2.018008000 | 2.346477000 |
| H | -1.922935000 | 3.001902000 | 1.990816000 |
| H | -1.776137000 | 1.961234000 | 3.437078000 |
| H | -0.514728000 | 1.894720000 | 2.161685000 |
| C | -1.866693000 | -1.044924000 | 1.687557000 |
| H | -1.517574000 | -1.208317000 | 2.723297000 |
| H | -2.590339000 | -1.823946000 | 1.418638000 |
| H | -0.987466000 | -1.102175000 | 1.031639000 |
| C | -4.379558000 | -1.613125000 | -0.832976000 |
| C | -4.080237000 | -2.947577000 | -0.487076000 |
| C | -5.724356000 | -1.190415000 | -0.762452000 |
| C | -5.079666000 | -3.817602000 | -0.047483000 |
| C | -6.722411000 | -2.060842000 | -0.324534000 |
| C | -6.403733000 | -3.375038000 | 0.041418000 |
| H | -3.048328000 | -3.301689000 | -0.561578000 |
| H | -5.994937000 | -0.178085000 | -1.069298000 |
| H | -4.825371000 | -4.845386000 | 0.222132000 |
| H | -7.758209000 | -1.716292000 | -0.278012000 |
| H | -7.188447000 | -4.054748000 | 0.381936000 |
| P | 2.416229000 | -0.869842000 | -0.409236000 |
| C | 4.130344000 | -0.182520000 | -0.901892000 |
| C | 2.596838000 | -1.524628000 | 1.304356000 |
| C | 3.501261000 | -2.546882000 | 1.652776000 |
| C | 1.815453000 | -0.951328000 | 2.324355000 |
| C | 3.607161000 | -2.986218000 | 2.974880000 |
| C | 1.919233000 | -1.392808000 | 3.646535000 |
| C | 2.814232000 | -2.414177000 | 3.975459000 |
| H | 4.316040000 | -3.779802000 | 3.222922000 |
| H | 1.301158000 | -0.930764000 | 4.420215000 |
| H | 2.899345000 | -2.760376000 | 5.008360000 |
| H | 4.136472000 | -3.009937000 | 0.899592000 |
| C | 4.588868000 | 0.670576000 | 0.298804000 |
| H | 4.822046000 | 0.055461000 | 1.179774000 |
| H | 5.501399000 | 1.221065000 | 0.017261000 |
| H | 3.825112000 | 1.411323000 | 0.585360000 |
| C | 5.190036000 | -1.247952000 | -1.221670000 |
| H | 6.150184000 | -0.742034000 | -1.417477000 |
| H | 5.358126000 | -1.945290000 | -0.389520000 |
| H | 4.938935000 | -1.826463000 | -2.122179000 |
| C | 3.959434000 | 0.744198000 | -2.124237000 |
| H | 4.938715000 | 1.187281000 | -2.369548000 |

| | | | |
|---|---|---|---|
| H | 3.606418000 | 0.210293000 | -3.016331000 |
| H | 3.260316000 | 1.569089000 | -1.919404000 |
| C | 1.827665000 | -2.331613000 | -1.484541000 |
| C | 0.386055000 | -2.628678000 | -1.022271000 |
| H | -0.014378000 | -3.475602000 | -1.603492000 |
| H | 0.344450000 | -2.905634000 | 0.042546000 |
| H | -0.275420000 | -1.765958000 | -1.186076000 |
| C | 2.650986000 | -3.624189000 | -1.352830000 |
| H | 2.249475000 | -4.366593000 | -2.062492000 |
| H | 3.712893000 | -3.485840000 | -1.590886000 |
| H | 2.569141000 | -4.060731000 | -0.348066000 |
| C | 1.802824000 | -1.870756000 | -2.953255000 |
| H | 1.270259000 | -0.914313000 | -3.075752000 |
| H | 2.814822000 | -1.764715000 | -3.369907000 |
| H | 1.277096000 | -2.627951000 | -3.557944000 |
| H | 1.125517000 | -0.140758000 | 2.084627000 |

**INT5**, E = -2085.613651

| | | | |
|---|---|---|---|
| Au | 0.701807000 | -0.221095000 | -0.396796000 |
| C | -1.368201000 | 0.850225000 | -0.617554000 |
| C | -1.603409000 | -0.482784000 | -0.915543000 |
| C | -2.187124000 | -1.551235000 | -0.021241000 |
| C | -3.680338000 | -1.597488000 | -0.249459000 |
| C | -4.580633000 | -1.244097000 | 0.684258000 |
| H | -1.568102000 | -0.758955000 | -1.979293000 |
| H | -1.965537000 | -1.368100000 | 1.035414000 |
| H | -1.732232000 | -2.517406000 | -0.297113000 |
| H | -4.003566000 | -1.895687000 | -1.252542000 |
| H | -4.204603000 | -0.937966000 | 1.667069000 |
| C | -1.137570000 | 1.705092000 | -1.855106000 |
| O | -1.690514000 | 2.910110000 | -1.711213000 |
| O | -0.552282000 | 1.351906000 | -2.855890000 |
| C | -1.561496000 | 3.827286000 | -2.810320000 |
| H | -2.038780000 | 3.413706000 | -3.710205000 |
| H | -2.065763000 | 4.747316000 | -2.494834000 |
| H | -0.499554000 | 4.019824000 | -3.021201000 |
| C | -6.045531000 | -1.200891000 | 0.551649000 |
| C | -6.806313000 | -0.694812000 | 1.624725000 |
| C | -6.731593000 | -1.630459000 | -0.603762000 |
| C | -8.199004000 | -0.612234000 | 1.547766000 |
| C | -8.121645000 | -1.549993000 | -0.680166000 |
| C | -8.863084000 | -1.039470000 | 0.394133000 |
| H | -6.293597000 | -0.357596000 | 2.530057000 |
| H | -6.175009000 | -2.036365000 | -1.451230000 |
| H | -8.766681000 | -0.213513000 | 2.392306000 |
| H | -8.633626000 | -1.889643000 | -1.584154000 |
| H | -9.952290000 | -0.978260000 | 0.330337000 |
| P | 2.993452000 | -0.676356000 | -0.180656000 |
| C | 3.843816000 | 0.069024000 | -1.722384000 |
| C | 3.687919000 | 0.137454000 | 1.309927000 |
| C | 5.072618000 | 0.161610000 | 1.566477000 |
| C | 2.821864000 | 0.762057000 | 2.225236000 |
| C | 5.571248000 | 0.797218000 | 2.705569000 |
| C | 3.323035000 | 1.394045000 | 3.366445000 |
| C | 4.698994000 | 1.414667000 | 3.608513000 |
| H | 6.648399000 | 0.808161000 | 2.886858000 |
| H | 2.632158000 | 1.873638000 | 4.063507000 |
| H | 5.092800000 | 1.911399000 | 4.498344000 |
| H | 5.776090000 | -0.313092000 | 0.884018000 |
| C | 3.879546000 | 1.592987000 | -1.488846000 |

| | | | |
|---|---|---|---|
| H | 4.554375000 | 1.875386000 | -0.668543000 |
| H | 4.240365000 | 2.080551000 | -2.408753000 |
| H | 2.877615000 | 1.996722000 | -1.269849000 |
| C | 5.264679000 | -0.464854000 | -1.968129000 |
| H | 5.690201000 | 0.068024000 | -2.833965000 |
| H | 5.942100000 | -0.291833000 | -1.121535000 |
| H | 5.268412000 | -1.536133000 | -2.212668000 |
| C | 2.974276000 | -0.219427000 | -2.965648000 |
| H | 3.477449000 | 0.213700000 | -3.845202000 |
| H | 2.847507000 | -1.293177000 | -3.156298000 |
| H | 1.977275000 | 0.242044000 | -2.895989000 |
| C | 3.225385000 | -2.549144000 | 0.052258000 |
| C | 2.281422000 | -2.951408000 | 1.205266000 |
| H | 2.435609000 | -4.018925000 | 1.430648000 |
| H | 2.487545000 | -2.381446000 | 2.124606000 |
| H | 1.221131000 | -2.814823000 | 0.941854000 |
| C | 4.664418000 | -2.937714000 | 0.434402000 |
| H | 4.717217000 | -4.036157000 | 0.507454000 |
| H | 5.406730000 | -2.622612000 | -0.309537000 |
| H | 4.951282000 | -2.530759000 | 1.413758000 |
| C | 2.800850000 | -3.266425000 | -1.240916000 |
| H | 1.802188000 | -2.949526000 | -1.581875000 |
| H | 3.517302000 | -3.105013000 | -2.058881000 |
| H | 2.760244000 | -4.350510000 | -1.047924000 |
| H | 1.745732000 | 0.760695000 | 2.048767000 |
| Si | -1.696745000 | 1.795979000 | 1.055282000 |
| C | -1.475199000 | 0.664963000 | 2.552593000 |
| H | -2.342981000 | 0.011903000 | 2.727175000 |
| H | -0.574374000 | 0.034011000 | 2.488895000 |
| H | -1.366472000 | 1.308476000 | 3.441632000 |
| C | -0.407083000 | 3.165491000 | 1.161098000 |
| H | -0.506683000 | 3.693100000 | 2.123962000 |
| H | 0.614877000 | 2.755887000 | 1.108007000 |
| H | -0.528700000 | 3.896547000 | 0.349345000 |
| C | -3.454192000 | 2.461164000 | 0.993475000 |
| H | -3.580437000 | 3.143678000 | 0.140573000 |
| H | -4.181136000 | 1.639589000 | 0.895053000 |
| H | -3.678158000 | 3.010077000 | 1.923240000 |

**INT6**, E = -1061.981397

| | | | |
|---|---|---|---|
| C | 1.982102000 | -0.348460000 | 0.654484000 |
| C | 1.168401000 | -0.860492000 | 1.605569000 |
| C | 0.003438000 | -0.209612000 | 2.306002000 |
| C | -1.283602000 | -0.593555000 | 1.614441000 |
| C | -2.095129000 | 0.281152000 | 0.995254000 |
| H | 1.317848000 | -1.918553000 | 1.861376000 |
| H | 0.101315000 | 0.880765000 | 2.343173000 |
| H | -0.022747000 | -0.576107000 | 3.348061000 |
| H | -1.514394000 | -1.664881000 | 1.601258000 |
| H | -1.817543000 | 1.341402000 | 1.016219000 |
| C | 2.944806000 | -1.349305000 | 0.085502000 |
| O | 3.334379000 | -1.005573000 | -1.160484000 |
| O | 3.335416000 | -2.364906000 | 0.623120000 |
| C | 4.252333000 | -1.882208000 | -1.818177000 |
| H | 3.817606000 | -2.886736000 | -1.932782000 |
| H | 4.448015000 | -1.438332000 | -2.801730000 |
| H | 5.188175000 | -1.966863000 | -1.245116000 |
| C | -3.338621000 | -0.011593000 | 0.262267000 |
| C | -4.004778000 | 1.043372000 | -0.393549000 |
| C | -3.901996000 | -1.302422000 | 0.174955000 |

| | | | |
|---|---|---|---|
| C | -5.182200000 | 0.821779000 | -1.112918000 |
| C | -5.077643000 | -1.524370000 | -0.541899000 |
| C | -5.724909000 | -0.464255000 | -1.191043000 |
| H | -3.587263000 | 2.052860000 | -0.338655000 |
| H | -3.418355000 | -2.144102000 | 0.675563000 |
| H | -5.677244000 | 1.657792000 | -1.613895000 |
| H | -5.495241000 | -2.533285000 | -0.594636000 |
| H | -6.646003000 | -0.641844000 | -1.751787000 |
| Si | 1.978007000 | 1.410124000 | -0.112088000 |
| C | 1.195768000 | 2.720240000 | 1.008650000 |
| H | 0.100312000 | 2.643021000 | 1.071835000 |
| H | 1.608947000 | 2.696875000 | 2.029960000 |
| H | 1.430745000 | 3.708228000 | 0.577395000 |
| C | 3.762525000 | 1.966703000 | -0.391822000 |
| H | 3.771971000 | 3.007841000 | -0.755890000 |
| H | 4.332393000 | 1.936939000 | 0.552180000 |
| H | 4.279154000 | 1.333475000 | -1.126446000 |
| C | 1.014488000 | 1.340073000 | -1.733757000 |
| H | 1.454938000 | 0.591981000 | -2.410371000 |
| H | -0.034446000 | 1.058960000 | -1.542223000 |
| H | 1.021452000 | 2.320385000 | -2.239102000 |

**TS4**, E = -2161.934136

| | | | |
|---|---|---|---|
| Au | 1.425847000 | -0.781849000 | -0.457781000 |
| C | -2.069908000 | 1.147581000 | -0.129908000 |
| C | -3.193085000 | 1.400672000 | -0.885039000 |
| C | -3.996743000 | 0.408379000 | -1.642188000 |
| C | -5.469785000 | 0.527076000 | -1.322744000 |
| C | -6.250252000 | -0.519822000 | -1.006473000 |
| H | -3.531844000 | 2.444473000 | -0.959100000 |
| H | -3.618513000 | -0.615376000 | -1.519283000 |
| H | -3.841425000 | 0.685196000 | -2.708217000 |
| H | -5.884149000 | 1.539488000 | -1.369832000 |
| H | -5.794033000 | -1.516321000 | -0.987879000 |
| C | -1.340818000 | 2.384696000 | 0.339783000 |
| O | -0.024490000 | 2.174279000 | 0.417357000 |
| O | -1.878063000 | 3.433112000 | 0.617197000 |
| C | 0.774688000 | 3.275837000 | 0.880102000 |
| H | 0.478831000 | 3.558402000 | 1.900883000 |
| H | 1.809725000 | 2.922925000 | 0.863697000 |
| H | 0.653418000 | 4.143259000 | 0.215890000 |
| C | -7.687736000 | -0.496302000 | -0.688886000 |
| C | -8.343645000 | -1.716563000 | -0.432204000 |
| C | -8.445482000 | 0.691908000 | -0.623370000 |
| C | -9.706354000 | -1.753472000 | -0.125250000 |
| C | -9.805076000 | 0.655471000 | -0.316145000 |
| C | -10.443308000 | -0.567061000 | -0.066291000 |
| H | -7.773867000 | -2.649035000 | -0.475784000 |
| H | -7.969910000 | 1.656913000 | -0.811330000 |
| H | -10.193279000 | -2.712485000 | 0.068667000 |
| H | -10.373564000 | 1.587788000 | -0.269821000 |
| H | -11.509041000 | -0.591470000 | 0.174021000 |
| P | 3.659592000 | -0.609227000 | 0.068150000 |
| C | 4.586023000 | -2.053994000 | -0.778025000 |
| C | 4.380938000 | 0.959211000 | -0.574357000 |
| C | 5.757841000 | 1.248063000 | -0.509673000 |
| C | 3.521809000 | 1.914906000 | -1.147005000 |
| C | 6.254582000 | 2.459001000 | -0.998026000 |
| C | 4.019835000 | 3.128882000 | -1.629440000 |
| C | 5.387648000 | 3.404570000 | -1.555998000 |

| | | | |
|---|---|---|---|
| H | 7.326327000 | 2.662998000 | -0.940223000 |
| H | 3.333047000 | 3.857944000 | -2.065950000 |
| H | 5.779278000 | 4.351709000 | -1.934802000 |
| H | 6.459533000 | 0.534729000 | -0.079919000 |
| C | 4.646806000 | -1.688439000 | -2.275055000 |
| H | 5.312041000 | -0.834362000 | -2.466377000 |
| H | 5.035194000 | -2.553818000 | -2.836421000 |
| H | 3.649346000 | -1.447309000 | -2.676536000 |
| C | 6.003470000 | -2.313748000 | -0.244051000 |
| H | 6.465067000 | -3.108079000 | -0.853337000 |
| H | 6.658138000 | -1.435464000 | -0.313517000 |
| H | 5.993611000 | -2.666661000 | 0.796319000 |
| C | 3.753027000 | -3.342883000 | -0.614223000 |
| H | 4.275197000 | -4.164806000 | -1.130779000 |
| H | 3.631206000 | -3.636884000 | 0.436822000 |
| H | 2.752071000 | -3.247303000 | -1.060154000 |
| C | 3.873735000 | -0.535845000 | 1.962075000 |
| C | 2.996318000 | 0.640047000 | 2.439338000 |
| H | 3.063978000 | 0.710031000 | 3.537190000 |
| H | 3.345977000 | 1.597316000 | 2.024051000 |
| H | 1.937538000 | 0.511000000 | 2.175061000 |
| C | 5.316072000 | -0.275079000 | 2.429949000 |
| H | 5.325604000 | -0.270198000 | 3.532444000 |
| H | 6.024000000 | -1.045191000 | 2.101209000 |
| H | 5.683258000 | 0.706384000 | 2.099792000 |
| C | 3.352904000 | -1.852404000 | 2.565951000 |
| H | 2.340575000 | -2.098616000 | 2.209405000 |
| H | 4.018185000 | -2.699683000 | 2.344733000 |
| H | 3.308013000 | -1.749294000 | 3.662406000 |
| H | 2.451924000 | 1.707905000 | -1.218148000 |
| Si | -2.599057000 | 0.068699000 | 1.571776000 |
| C | -3.176350000 | -1.620156000 | 1.037629000 |
| H | -4.192411000 | -1.602796000 | 0.621467000 |
| H | -2.469217000 | -2.037855000 | 0.306202000 |
| H | -3.185103000 | -2.266971000 | 1.931376000 |
| C | -0.995005000 | 0.014263000 | 2.525937000 |
| H | -1.185410000 | -0.547162000 | 3.456512000 |
| H | -0.217277000 | -0.511340000 | 1.953821000 |
| H | -0.627647000 | 1.013672000 | 2.796795000 |
| C | -3.910771000 | 1.133745000 | 2.371075000 |
| H | -3.573035000 | 2.179107000 | 2.441364000 |
| H | -4.843714000 | 1.100255000 | 1.786095000 |
| H | -4.122153000 | 0.762210000 | 3.387519000 |
| O | -0.590388000 | -1.014647000 | -0.868013000 |
| H | -1.425365000 | 0.197208000 | -0.452492000 |
| H | -0.733907000 | -1.172929000 | -1.813503000 |

**INT7**, E = -2161.933793

| | | | |
|---|---|---|---|
| Au | 1.397456000 | -0.840019000 | -0.471582000 |
| C | -1.970947000 | 1.385457000 | -0.282922000 |
| C | -3.237880000 | 1.649710000 | -0.744277000 |
| C | -4.067087000 | 0.742526000 | -1.576362000 |
| C | -5.519674000 | 0.699138000 | -1.174461000 |
| C | -6.229492000 | -0.437275000 | -1.079595000 |
| H | -3.667709000 | 2.633230000 | -0.507081000 |
| H | -3.614789000 | -0.255403000 | -1.669275000 |
| H | -3.992406000 | 1.211075000 | -2.585013000 |
| H | -5.985971000 | 1.669211000 | -0.972382000 |
| H | -5.719348000 | -1.382053000 | -1.302204000 |
| C | -1.191679000 | 2.560754000 | 0.250819000 |

| | | | |
|---|---|---|---|
| O | 0.116761000 | 2.380504000 | 0.082326000 |
| O | -1.691805000 | 3.538627000 | 0.760302000 |
| C | 0.974824000 | 3.449479000 | 0.520468000 |
| H | 0.932077000 | 3.544797000 | 1.615115000 |
| H | 1.984149000 | 3.175649000 | 0.198709000 |
| H | 0.666194000 | 4.399120000 | 0.061622000 |
| C | -7.647558000 | -0.575002000 | -0.708248000 |
| C | -8.214016000 | -1.864319000 | -0.668139000 |
| C | -8.471364000 | 0.523245000 | -0.383613000 |
| C | -9.553289000 | -2.053996000 | -0.317380000 |
| C | -9.807641000 | 0.334310000 | -0.033155000 |
| C | -10.356034000 | -0.955024000 | 0.001802000 |
| H | -7.592210000 | -2.728957000 | -0.916631000 |
| H | -8.066966000 | 1.537529000 | -0.403588000 |
| H | -9.970412000 | -3.063861000 | -0.293881000 |
| H | -10.428654000 | 1.198860000 | 0.214759000 |
| H | -11.403869000 | -1.099102000 | 0.276447000 |
| P | 3.605457000 | -0.651691000 | 0.162690000 |
| C | 4.525736000 | -2.230536000 | -0.404455000 |
| C | 4.421714000 | 0.779901000 | -0.661292000 |
| C | 5.800622000 | 1.041944000 | -0.548661000 |
| C | 3.634829000 | 1.653298000 | -1.434479000 |
| C | 6.370056000 | 2.147918000 | -1.184694000 |
| C | 4.204873000 | 2.763272000 | -2.065216000 |
| C | 5.574022000 | 3.014196000 | -1.941371000 |
| H | 7.442386000 | 2.332374000 | -1.085894000 |
| H | 3.573766000 | 3.430167000 | -2.657458000 |
| H | 6.021633000 | 3.879825000 | -2.435631000 |
| H | 6.446284000 | 0.388145000 | 0.035533000 |
| C | 4.683832000 | -2.095428000 | -1.932908000 |
| H | 5.393723000 | -1.302060000 | -2.207329000 |
| H | 5.065869000 | -3.047304000 | -2.336745000 |
| H | 3.721378000 | -1.884170000 | -2.425957000 |
| C | 5.902244000 | -2.453498000 | 0.243986000 |
| H | 6.368808000 | -3.335741000 | -0.224431000 |
| H | 6.590807000 | -1.610940000 | 0.097366000 |
| H | 5.823884000 | -2.663171000 | 1.320056000 |
| C | 3.636765000 | -3.456130000 | -0.103322000 |
| H | 4.156560000 | -4.361632000 | -0.456846000 |
| H | 3.443286000 | -3.583716000 | 0.970253000 |
| H | 2.667705000 | -3.400149000 | -0.620663000 |
| C | 3.725298000 | -0.306493000 | 2.034551000 |
| C | 2.865660000 | 0.950095000 | 2.282810000 |
| H | 2.882684000 | 1.186513000 | 3.359379000 |
| H | 3.261779000 | 1.823392000 | 1.743885000 |
| H | 1.818089000 | 0.807199000 | 1.985714000 |
| C | 5.146765000 | -0.020237000 | 2.547851000 |
| H | 5.094293000 | 0.138392000 | 3.637783000 |
| H | 5.845473000 | -0.846445000 | 2.371823000 |
| H | 5.565269000 | 0.895491000 | 2.108362000 |
| C | 3.121837000 | -1.505031000 | 2.788740000 |
| H | 2.132109000 | -1.784165000 | 2.394409000 |
| H | 3.775400000 | -2.388423000 | 2.748962000 |
| H | 2.999215000 | -1.235645000 | 3.850461000 |
| H | 2.565143000 | 1.462438000 | -1.546183000 |
| Si | -2.658247000 | 0.339626000 | 1.527573000 |
| C | -3.156773000 | -1.352132000 | 0.952510000 |
| H | -4.174723000 | -1.377207000 | 0.542489000 |
| H | -2.421221000 | -1.703805000 | 0.211456000 |
| H | -3.120903000 | -2.018280000 | 1.831781000 |
| C | -1.034986000 | 0.360478000 | 2.446426000 |

| | | | |
|---|---|---|---|
| H | -1.211869000 | -0.183777000 | 3.390656000 |
| H | -0.258621000 | -0.171349000 | 1.879195000 |
| H | -0.689213000 | 1.372865000 | 2.696963000 |
| C | -3.982293000 | 1.405755000 | 2.291558000 |
| H | -3.656869000 | 2.456405000 | 2.328671000 |
| H | -4.929535000 | 1.334511000 | 1.735117000 |
| H | -4.160615000 | 1.057807000 | 3.322926000 |
| O | -0.581606000 | -1.063158000 | -0.970607000 |
| H | -1.411654000 | 0.484178000 | -0.655592000 |
| H | -0.662824000 | -1.395766000 | -1.876488000 |

**3a´a**, E = -653.558248

| | | | |
|---|---|---|---|
| C | -2.292853000 | 0.336218000 | -0.079645000 |
| C | -2.054929000 | 1.652228000 | -0.190530000 |
| C | -0.706986000 | 2.300549000 | -0.339066000 |
| C | 0.441504000 | 1.359676000 | -0.560288000 |
| C | 1.487347000 | 1.227397000 | 0.273260000 |
| H | -2.919005000 | 2.326897000 | -0.165672000 |
| H | -0.528343000 | 2.934505000 | 0.549521000 |
| H | -0.777148000 | 3.012027000 | -1.185365000 |
| H | 0.383346000 | 0.752331000 | -1.470859000 |
| H | 1.503901000 | 1.850350000 | 1.176317000 |
| C | -3.674107000 | -0.176337000 | 0.068411000 |
| O | -3.681272000 | -1.518558000 | 0.178743000 |
| O | -4.686672000 | 0.494150000 | 0.091519000 |
| C | -4.960274000 | -2.139360000 | 0.328848000 |
| H | -5.603601000 | -1.924517000 | -0.538389000 |
| H | -4.772577000 | -3.217352000 | 0.403542000 |
| H | -5.466634000 | -1.777764000 | 1.237093000 |
| C | 2.645989000 | 0.329944000 | 0.122908000 |
| C | 3.664769000 | 0.364491000 | 1.096113000 |
| C | 2.790634000 | -0.573824000 | -0.951274000 |
| C | 4.784854000 | -0.466124000 | 1.003560000 |
| C | 3.907753000 | -1.403546000 | -1.044395000 |
| C | 4.912012000 | -1.354925000 | -0.067968000 |
| H | 3.573717000 | 1.056215000 | 1.938473000 |
| H | 2.020116000 | -0.631504000 | -1.723115000 |
| H | 5.560570000 | -0.418664000 | 1.772272000 |
| H | 3.997292000 | -2.096049000 | -1.885513000 |
| H | 5.785835000 | -2.006879000 | -0.144048000 |
| H | -1.488194000 | -0.402660000 | -0.085959000 |

**INT4-b**, E = -2084.355191

| | | | |
|---|---|---|---|
| Au | 0.933442000 | 0.695644000 | -0.420364000 |
| C | -0.788678000 | 1.866435000 | -0.451333000 |
| C | -2.019205000 | 1.504822000 | -0.871469000 |
| C | -2.366187000 | 0.159384000 | -1.449909000 |
| C | -3.421364000 | -0.544719000 | -0.601806000 |
| C | -4.666832000 | -0.422215000 | -0.431701000 |
| H | -2.861486000 | 2.205534000 | -0.801747000 |
| H | -1.467903000 | -0.464336000 | -1.537563000 |
| H | -2.800400000 | 0.272522000 | -2.457082000 |
| C | -0.622569000 | 3.237005000 | 0.117913000 |
| O | -1.500214000 | 4.145237000 | -0.357550000 |
| O | 0.227026000 | 3.537011000 | 0.937183000 |
| C | -1.399612000 | 5.470263000 | 0.169436000 |
| H | -0.400824000 | 5.891919000 | -0.021451000 |
| H | -2.166129000 | 6.064485000 | -0.343297000 |

| | | | |
|---|---|---|---|
| H | -1.578067000 | 5.476315000 | 1.255939000 |
| Si | -2.807523000 | -1.969205000 | 0.673436000 |
| C | -4.277454000 | -2.771452000 | 1.511250000 |
| H | -4.967112000 | -3.229521000 | 0.785563000 |
| H | -3.900086000 | -3.570016000 | 2.171491000 |
| H | -4.839697000 | -2.057170000 | 2.132129000 |
| C | -1.728371000 | -1.034883000 | 1.883388000 |
| H | -2.342045000 | -0.354098000 | 2.493470000 |
| H | -1.240157000 | -1.760631000 | 2.555589000 |
| H | -0.950040000 | -0.444119000 | 1.378451000 |
| C | -1.904742000 | -3.176356000 | -0.439810000 |
| H | -1.461686000 | -3.964605000 | 0.191850000 |
| H | -2.602575000 | -3.651733000 | -1.146399000 |
| H | -1.091863000 | -2.704065000 | -1.008232000 |
| C | -6.028061000 | -0.370169000 | -0.146863000 |
| C | -6.943024000 | -1.179322000 | -0.877753000 |
| C | -6.501635000 | 0.493440000 | 0.881329000 |
| C | -8.297023000 | -1.119844000 | -0.579490000 |
| C | -7.859113000 | 0.538086000 | 1.165829000 |
| C | -8.751267000 | -0.265602000 | 0.438177000 |
| H | -6.567301000 | -1.837883000 | -1.662659000 |
| H | -5.789063000 | 1.108205000 | 1.433969000 |
| H | -9.007485000 | -1.736255000 | -1.133154000 |
| H | -8.232555000 | 1.195574000 | 1.952925000 |
| H | -9.818695000 | -0.225958000 | 0.668239000 |
| P | 2.957101000 | -0.568061000 | -0.384316000 |
| C | 4.062057000 | -0.000649000 | -1.838881000 |
| C | 3.892605000 | -0.282161000 | 1.176903000 |
| C | 5.221667000 | -0.705597000 | 1.367711000 |
| C | 3.238512000 | 0.372338000 | 2.236818000 |
| C | 5.875315000 | -0.478343000 | 2.581106000 |
| C | 3.891465000 | 0.593149000 | 3.453544000 |
| C | 5.211518000 | 0.169727000 | 3.628509000 |
| H | 6.908206000 | -0.811508000 | 2.707716000 |
| H | 3.364725000 | 1.103402000 | 4.263500000 |
| H | 5.724416000 | 0.345780000 | 4.577270000 |
| H | 5.760761000 | -1.216671000 | 0.571247000 |
| C | 4.638959000 | 1.365940000 | -1.417419000 |
| H | 5.374967000 | 1.273862000 | -0.606216000 |
| H | 5.143059000 | 1.824651000 | -2.283842000 |
| H | 3.845724000 | 2.054932000 | -1.085191000 |
| C | 5.200564000 | -0.960150000 | -2.221396000 |
| H | 5.795544000 | -0.495895000 | -3.025388000 |
| H | 5.888754000 | -1.167228000 | -1.391383000 |
| H | 4.822021000 | -1.916576000 | -2.608363000 |
| C | 3.158046000 | 0.207898000 | -3.073611000 |
| H | 3.780681000 | 0.563884000 | -3.910968000 |
| H | 2.664598000 | -0.718268000 | -3.398462000 |
| H | 2.379851000 | 0.963567000 | -2.888755000 |
| C | 2.585678000 | -2.437514000 | -0.389914000 |
| C | 1.590407000 | -2.661396000 | 0.767270000 |
| H | 1.332604000 | -3.732061000 | 0.818402000 |
| H | 2.018231000 | -2.371355000 | 1.739126000 |
| H | 0.659489000 | -2.097320000 | 0.622369000 |
| C | 3.812470000 | -3.334806000 | -0.156137000 |
| H | 3.483922000 | -4.387603000 | -0.169065000 |
| H | 4.581568000 | -3.223121000 | -0.930582000 |
| H | 4.273218000 | -3.149170000 | 0.824370000 |
| C | 1.908255000 | -2.797462000 | -1.723979000 |
| H | 1.074986000 | -2.115850000 | -1.956716000 |
| H | 2.615076000 | -2.777853000 | -2.565896000 |

| | | | |
|---|---|---|---|
| H | 1.501318000 | -3.820007000 | -1.659881000 |
| H | 2.209525000 | 0.717043000 | 2.110692000 |

**TS3-b**, E = -2084.329517

| | | | |
|---|---|---|---|
| Au | 0.985216000 | 0.929371000 | -0.473321000 |
| C | -0.517665000 | 2.368853000 | -0.534998000 |
| C | -1.671073000 | 2.281560000 | -1.232937000 |
| C | -2.037061000 | 1.120412000 | -2.129972000 |
| C | -3.148296000 | 0.324187000 | -1.606141000 |
| C | -4.095793000 | -0.369873000 | -1.274922000 |
| H | -2.417093000 | 3.085017000 | -1.183709000 |
| H | -1.163134000 | 0.464302000 | -2.275625000 |
| H | -2.325408000 | 1.496515000 | -3.128816000 |
| C | -0.265800000 | 3.603107000 | 0.266913000 |
| O | -1.382663000 | 4.285786000 | 0.610113000 |
| O | 0.837802000 | 3.975144000 | 0.617496000 |
| C | -1.191102000 | 5.493580000 | 1.353472000 |
| H | -0.579006000 | 6.209604000 | 0.784666000 |
| H | -2.191677000 | 5.906334000 | 1.531098000 |
| H | -0.688774000 | 5.285354000 | 2.310643000 |
| Si | -2.563611000 | 0.210594000 | 1.631581000 |
| C | -4.367453000 | 0.395792000 | 1.961895000 |
| H | -4.952257000 | -0.478103000 | 1.651729000 |
| H | -4.476215000 | 0.549584000 | 3.050758000 |
| H | -4.760413000 | 1.297181000 | 1.464078000 |
| C | -1.511949000 | 1.494736000 | 2.430269000 |
| H | -1.896360000 | 2.502604000 | 2.216875000 |
| H | -1.611038000 | 1.316536000 | 3.518396000 |
| H | -0.454730000 | 1.432093000 | 2.145003000 |
| C | -1.812341000 | -1.391296000 | 1.121313000 |
| H | -1.322399000 | -1.823856000 | 2.012840000 |
| H | -2.564964000 | -2.091872000 | 0.738012000 |
| H | -1.029204000 | -1.226688000 | 0.367166000 |
| C | -5.206107000 | -1.201101000 | -0.922614000 |
| C | -5.037443000 | -2.595863000 | -0.783814000 |
| C | -6.481266000 | -0.637609000 | -0.700715000 |
| C | -6.117201000 | -3.401471000 | -0.420056000 |
| C | -7.554552000 | -1.451045000 | -0.337918000 |
| C | -7.375824000 | -2.832360000 | -0.193490000 |
| H | -4.057705000 | -3.040636000 | -0.970941000 |
| H | -6.616862000 | 0.440299000 | -0.808983000 |
| H | -5.976489000 | -4.479813000 | -0.315438000 |
| H | -8.537163000 | -1.005306000 | -0.166509000 |
| H | -8.218973000 | -3.465816000 | 0.092094000 |
| P | 2.680706000 | -0.748891000 | -0.403059000 |
| C | 4.394830000 | 0.080808000 | -0.556109000 |
| C | 2.681140000 | -1.655285000 | 1.202423000 |
| C | 3.614672000 | -2.659797000 | 1.523392000 |
| C | 1.716114000 | -1.310728000 | 2.165925000 |
| C | 3.571131000 | -3.304490000 | 2.762388000 |
| C | 1.669653000 | -1.957599000 | 3.404008000 |
| C | 2.596417000 | -2.959475000 | 3.704727000 |
| H | 4.305226000 | -4.080631000 | 2.991400000 |
| H | 0.909658000 | -1.671842000 | 4.135438000 |
| H | 2.564452000 | -3.466545000 | 4.672109000 |
| H | 4.388202000 | -2.947263000 | 0.813237000 |
| C | 4.645981000 | 0.755249000 | 0.808616000 |
| H | 4.812422000 | 0.020740000 | 1.609790000 |
| H | 5.546434000 | 1.386680000 | 0.734356000 |
| H | 3.805067000 | 1.403703000 | 1.102926000 |

| | | | |
|---|---|---|---|
| C | 5.553263000 | -0.871887000 | -0.889233000 |
| H | 6.495246000 | -0.298476000 | -0.879101000 |
| H | 5.662941000 | -1.682566000 | -0.156227000 |
| H | 5.453113000 | -1.312359000 | -1.891290000 |
| C | 4.313672000 | 1.176714000 | -1.639319000 |
| H | 5.283416000 | 1.699163000 | -1.685174000 |
| H | 4.110801000 | 0.770516000 | -2.639071000 |
| H | 3.540438000 | 1.924972000 | -1.408834000 |
| C | 2.317820000 | -2.054221000 | -1.746003000 |
| C | 0.849184000 | -2.477428000 | -1.527537000 |
| H | 0.589179000 | -3.260475000 | -2.258624000 |
| H | 0.687750000 | -2.893598000 | -0.520964000 |
| H | 0.153067000 | -1.637942000 | -1.674819000 |
| C | 3.196410000 | -3.315441000 | -1.687997000 |
| H | 2.931667000 | -3.964174000 | -2.539523000 |
| H | 4.268927000 | -3.097314000 | -1.764562000 |
| H | 3.019107000 | -3.895086000 | -0.771667000 |
| C | 2.451725000 | -1.381370000 | -3.124158000 |
| H | 1.886156000 | -0.437704000 | -3.177723000 |
| H | 3.500138000 | -1.176833000 | -3.385043000 |
| H | 2.048448000 | -2.059098000 | -3.894419000 |
| H | 0.998247000 | -0.519895000 | 1.945248000 |

**INT5-b**, E = -2084.384801

| | | | |
|---|---|---|---|
| Au | 0.719706000 | 0.335461000 | -0.403070000 |
| C | -1.411185000 | 0.905725000 | 0.352063000 |
| C | -1.558867000 | 0.651569000 | -1.000803000 |
| C | -2.054241000 | -0.629286000 | -1.657977000 |
| C | -3.506054000 | -0.726923000 | -1.502403000 |
| C | -4.701576000 | -0.770368000 | -1.285362000 |
| H | -1.515464000 | 1.518623000 | -1.673132000 |
| H | -1.573176000 | -1.519923000 | -1.229183000 |
| H | -1.782148000 | -0.606600000 | -2.724717000 |
| C | -1.205301000 | 2.384176000 | 0.659788000 |
| O | -1.649761000 | 2.687271000 | 1.878815000 |
| O | -0.712077000 | 3.194410000 | -0.094480000 |
| C | -1.527661000 | 4.055459000 | 2.301391000 |
| H | -2.089413000 | 4.714885000 | 1.624555000 |
| H | -1.944543000 | 4.095658000 | 3.313717000 |
| H | -0.470546000 | 4.358256000 | 2.304862000 |
| C | -6.104590000 | -0.830493000 | -1.005591000 |
| C | -6.564881000 | -1.441022000 | 0.181353000 |
| C | -7.044430000 | -0.275570000 | -1.899965000 |
| C | -7.930565000 | -1.490706000 | 0.463228000 |
| C | -8.408302000 | -0.330649000 | -1.609893000 |
| C | -8.855380000 | -0.936555000 | -0.429653000 |
| H | -5.842144000 | -1.873175000 | 0.876532000 |
| H | -6.694515000 | 0.198323000 | -2.819481000 |
| H | -8.275623000 | -1.965061000 | 1.385148000 |
| H | -9.127685000 | 0.102594000 | -2.309068000 |
| H | -9.924215000 | -0.976939000 | -0.206047000 |
| P | 3.029014000 | -0.055415000 | -0.565967000 |
| C | 3.902192000 | 1.598013000 | -0.188977000 |
| C | 3.555008000 | -1.304411000 | 0.671818000 |
| C | 4.912167000 | -1.552731000 | 0.953147000 |
| C | 2.577244000 | -2.055593000 | 1.348380000 |
| C | 5.274970000 | -2.520928000 | 1.892030000 |
| C | 2.942813000 | -3.027925000 | 2.283047000 |
| C | 4.292722000 | -3.260958000 | 2.558969000 |
| H | 6.332575000 | -2.697774000 | 2.100333000 |
| H | 2.166987000 | -3.599835000 | 2.797110000 |

| | | | |
|---|---|---|---|
| H | 4.580630000 | -4.017834000 | 3.292332000 |
| H | 5.699162000 | -0.997585000 | 0.444484000 |
| C | 3.810814000 | 1.786531000 | 1.338627000 |
| H | 4.425018000 | 1.059100000 | 1.887554000 |
| H | 4.172624000 | 2.796184000 | 1.591081000 |
| H | 2.771888000 | 1.704047000 | 1.697480000 |
| C | 5.369126000 | 1.646904000 | -0.647988000 |
| H | 5.799879000 | 2.610053000 | -0.329820000 |
| H | 5.986513000 | 0.855950000 | -0.202665000 |
| H | 5.462252000 | 1.593461000 | -1.741505000 |
| C | 3.121256000 | 2.739454000 | -0.876090000 |
| H | 3.638609000 | 3.688876000 | -0.663686000 |
| H | 3.073966000 | 2.626528000 | -1.967152000 |
| H | 2.093438000 | 2.829950000 | -0.493537000 |
| C | 3.405345000 | -0.808347000 | -2.271781000 |
| C | 2.440787000 | -2.003690000 | -2.419911000 |
| H | 2.645380000 | -2.507309000 | -3.378424000 |
| H | 2.575742000 | -2.744390000 | -1.616750000 |
| H | 1.386516000 | -1.684931000 | -2.425329000 |
| C | 4.851881000 | -1.317868000 | -2.395434000 |
| H | 4.984892000 | -1.732818000 | -3.407834000 |
| H | 5.599994000 | -0.525346000 | -2.265723000 |
| H | 5.065127000 | -2.124857000 | -1.680672000 |
| C | 3.107833000 | 0.236017000 | -3.361245000 |
| H | 2.094028000 | 0.656433000 | -3.267249000 |
| H | 3.832374000 | 1.062455000 | -3.353102000 |
| H | 3.176909000 | -0.251788000 | -4.346853000 |
| H | 1.519386000 | -1.882228000 | 1.145638000 |
| Si | -1.844502000 | -0.273324000 | 1.844283000 |
| C | -1.794835000 | -2.083585000 | 1.318514000 |
| H | -2.629018000 | -2.345096000 | 0.650286000 |
| H | -0.846582000 | -2.365494000 | 0.833689000 |
| H | -1.893969000 | -2.696097000 | 2.230480000 |
| C | -0.546988000 | 0.001805000 | 3.182152000 |
| H | -0.773739000 | -0.640125000 | 4.049499000 |
| H | 0.461271000 | -0.264095000 | 2.824485000 |
| H | -0.532015000 | 1.048057000 | 3.519142000 |
| C | -3.581237000 | 0.170390000 | 2.412336000 |
| H | -3.627037000 | 1.202740000 | 2.787249000 |
| H | -4.296240000 | 0.064113000 | 1.581466000 |
| H | -3.889760000 | -0.514689000 | 3.219602000 |

## 12. References


1. Sarabia, F. J.; Li, Q.; Ferreira, E. M. *Angew. Chem. Int. Ed.* **2018**, *57*, 11015.
2. Barluenga, J.; Lonzi, G.; Riesgo, L.; Tomás, M.; López, L. A. *J. Am. Chem. Soc.* **2011**, *133*, 18138.
3. Ueda, Y.; Roberge, G.; Vinet, V. *Can. J. Chem.* **1984**, *62*, 2936.
4. (a) Boelke, A.; Caspers, L. D.; Nachtsheim, B. J. *Org. Lett.* **2017**, *19*, 5344. (b) Faβbender, S. I.; Molloy, J. J.; Mück-Lichtenfeld, C. Gilmour, R. *Angew. Chem. Int.Ed.* **2019**, *58*,18619.
5. (a) Chuprun, S.; Acosta, C. M.; Mathivathanan, L.; Bukhryakov, K. V. *Organometallics* **2020**, *39*, 3453. (b) Rajkiewicz, A. A.; Wojciechowska, N.; Kalek, M. *ACS Catal.* **2020**, *10*, 831.
6. Chen, J.; Che, C.-M. *Angew. Chem. Int. Ed.* **2004**, *43*, 4950.
7. Barluenga, J.; Lonzi, G.; Tomás, M. López, L. A. *Chem. Eur. J.* **2013**, *19*, 1573.
8. Ma, S.; Zhang, A.; Yu, Y.; Xia, W. *J. Org. Chem.* **2000**, *65*, 2287.
9. Gaussian 09, Revision D.01, Frisch, M. J.; Trucks, G. W.; Schlegel, H. B.; Scuseria, G. E.; Robb, M. A.; Cheeseman, J. R.; Scalmani, G.; Barone, V.; Mennucci, B.; Petersson, G. A.; Nakatsuji, H.; Caricato, M.; Li, X.; Hratchian, H. P.; Izmaylov, A. F.; Bloino, J.; Zheng, G.; Sonnenberg, J. L.; Hada, M.; Ehara, M.; Toyota, K.; Fukuda, R.; Hasegawa, J.; Ishida, M.; Nakajima, T.; Honda, Y.; Kitao, O.; Nakai, H.; Vreven, T.; Montgomery, J. A., Jr.; Peralta, J. E.; Ogliaro, F.; Bearpark, M.; Heyd, J. J.; Brothers, E.; Kudin, K. N.; Staroverov, V. N.; Kobayashi, R.; Normand, J.; Raghavachari, K.; Rendell, A.; Burant, J. C.; Iyengar, S. S.; Tomasi, J.; Cossi, M.; Rega, N.; Millam, J. M.; Klene, M.; Knox, J. E.; Cross, J. B.; Bakken, V.; Adamo, C.; Jaramillo, J.; Gomperts, R.; Stratmann, R. E.; Yazyev, O.; Austin, A. J.; Cammi, R.; Pomelli, C.; Ochterski, J. W.; Martin, R. L.; Morokuma, K.; Zakrzewski, V. G.; Voth, G. A.; Salvador, P.; Dannenberg, J. J.; Dapprich, S.; Daniels, A. D.; Farkas, Ö.; Foresman, J. B.; Ortiz, J. V.; Cioslowski, J.; Fox, D. J. Gaussian, Inc., Wallingford CT, 2009.
10. (a) Becke, A. D. Density-functional thermochemistry. III. The role of exact exchange. *J. Chem. Phys.* **1993**, *98*, 5648. (b) Lee, C.; Yan, W.; Parr, R. G. Development of the Colle-Salvetti correlation-energy formula into a functional of the electron density. *Phys. Rev. B* **1988**, *37*, 785. (c) Vosko, S. H.; Wilk, L.; Nusair, M. Accurate spin-dependent electron liquid correlation energies for local spin density calculations: a critical analysis. *Can. J. Phys*. **1980**, *58*, 1200.
11. Grimme, S.; Antony, J.; Ehrlich, S.; Krieg, H. A consistent and accurate ab initio

parametrization of density functional dispersion correction (DFT-D) for the 94 elements H-Pu. *J. Chem. Phys*. **2010**, *132*, 154104.

12. (a) Weigend, F.; Ahlrichs, R. Balanced basis sets of split valence, triple zeta valence and quadruple zeta valence quality for H to Rn: Design and assessment of accuracy, *Phys. Chem. Chem. Phys.* **2005**, 7, 3297-3305. (b) Weigend, F. Accurate Coulomb-fitting basis sets for H to Rn, *Phys. Chem. Chem. Phys.* **2006**, 8, 1057-1065.
13. (a) Miertuš, S.; Scrocco, E.; Tomasi, J. Electrostatic interaction of a solute with a continuum. A direct utilization of ab-initio molecular potentials for the prevision of solvent effects. *Chem. Phys*. **1981**, *55*, 117-129. (b) Pascual-Ahuir, J. L.; Silla, E.; Tuñón, I. GEPOL: An improved description of molecular surfaces. III. A new algorithm for the computation of a solvent-excluding surface. *J. Comp. Chem*. **1994**, 15, 1127-1138. (c) Barone, V.; Cossi, M. Quantum Calculation of Molecular Energies and Energy Gradients in Solution by a Conductor Solvent Model. *J. Phys. Chem. A*, **1998**, *102*, 1995-2001.
14. Gonzalez, C.; Schlegel, H. B. Reaction path following in mass-weighted internal coordinates. *J. Phys. Chem*. **1990**, *94*, 5523-5527.

## 13. $^1$H- and $^{13}$C-NMR spectra for new compounds

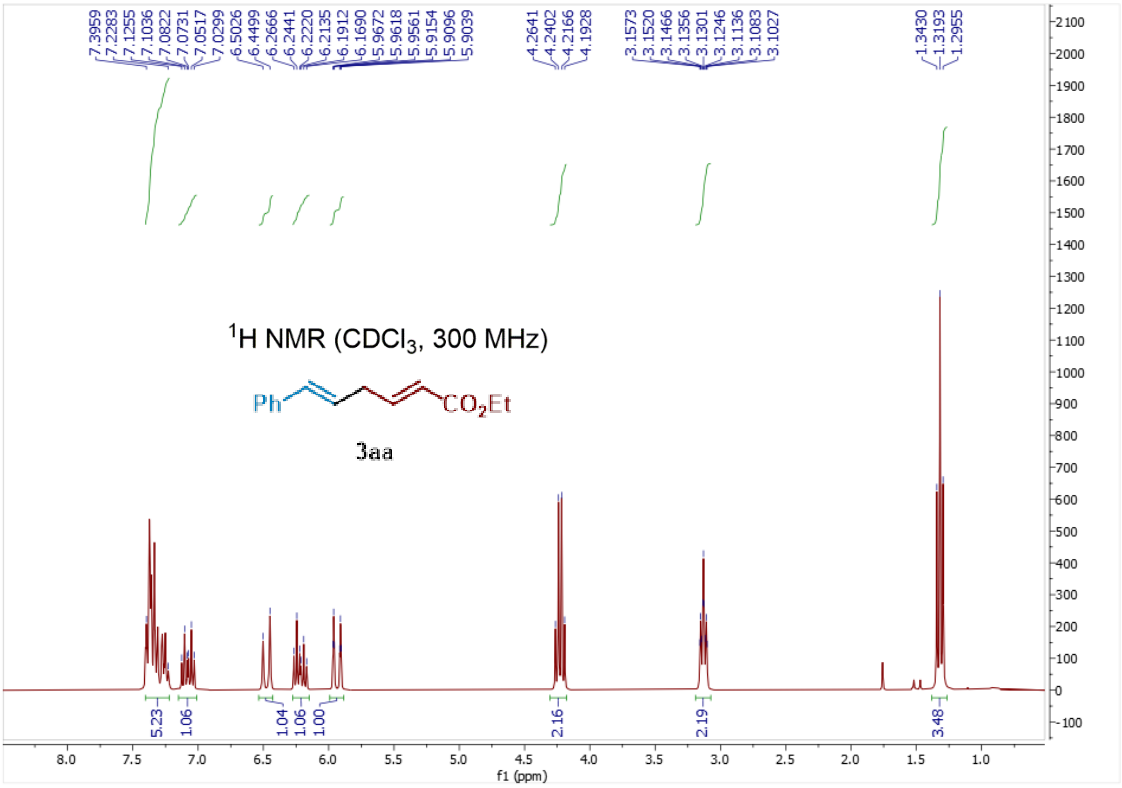


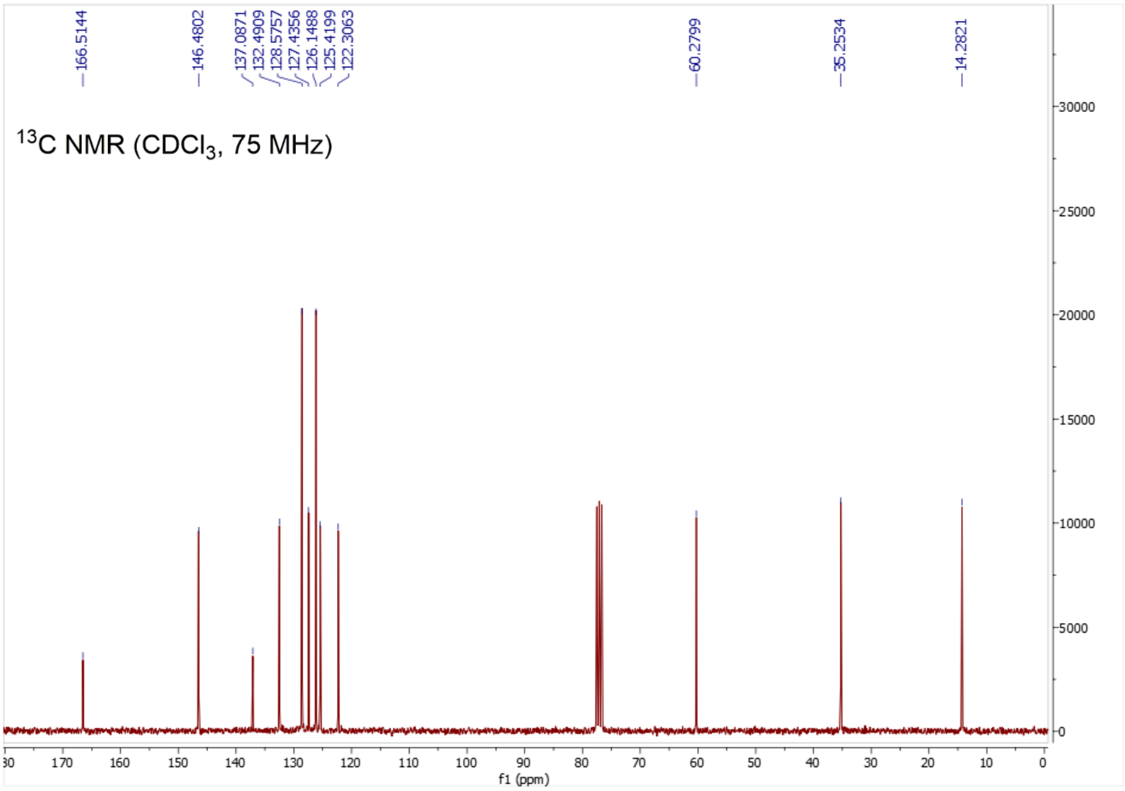

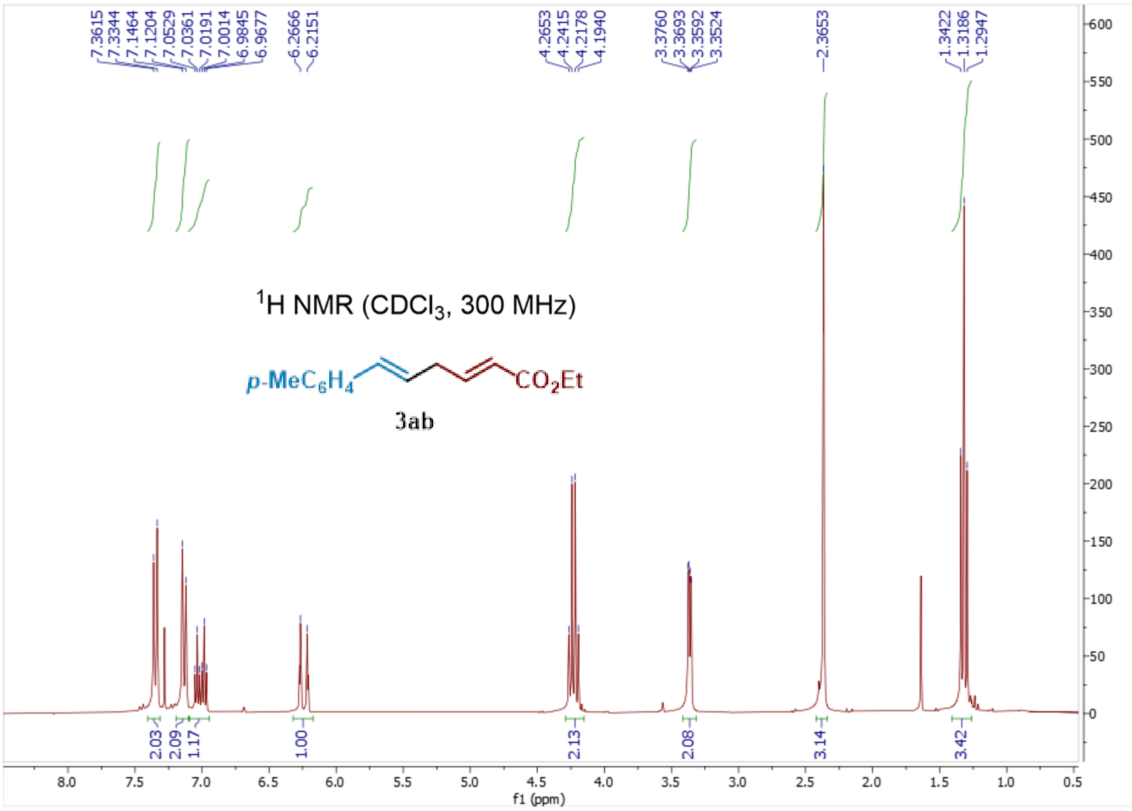

7.3615
7.3344
7.1464
7.1204
7.0529
7.0361
7.0191
7.0014
6.9845
6.9677
6.2666
6.2151
4.2653
4.2415
4.2178
4.1940
3.3760
3.3693
3.3592
3.3524
2.3653
1.3422
1.3186
1.2947
¹H NMR (CDCl₃, 300 MHz)
p-MeC₆H₄
CO₂Et
3ab
2.03
2.09
1.17
1.00
2.13
2.08
3.14
3.42
f1 (ppm)


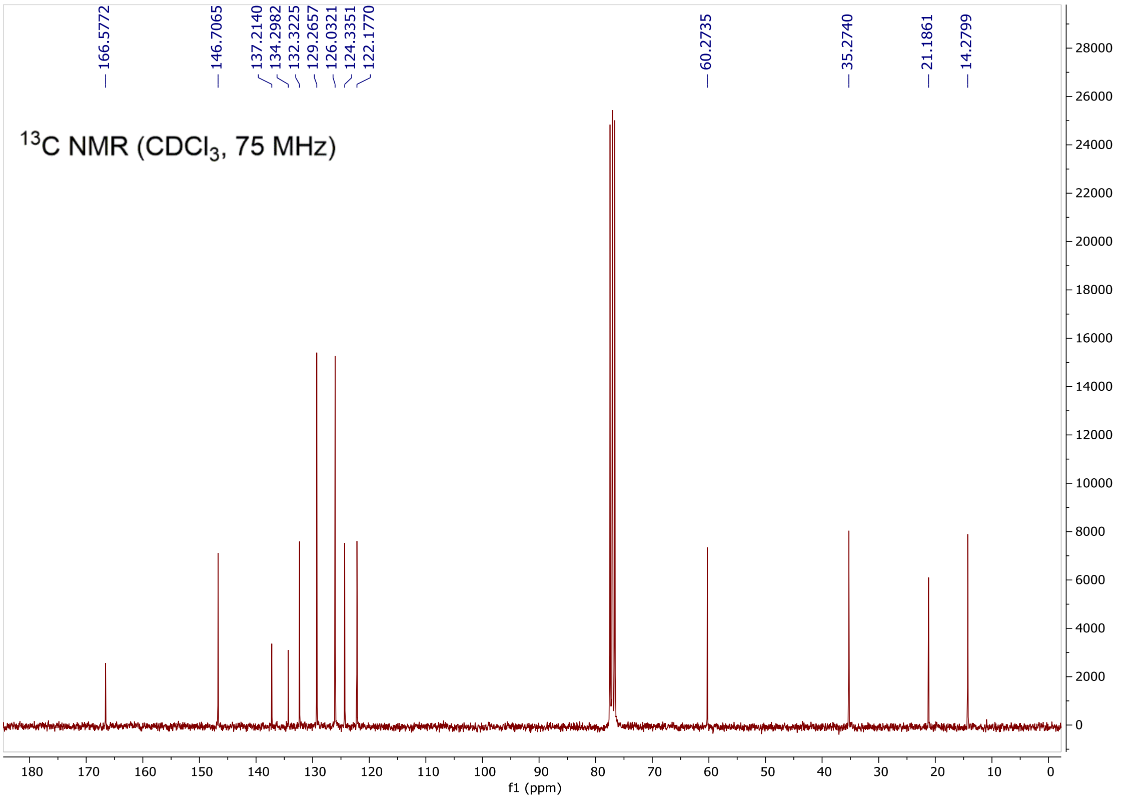

166.5772
146.7065
137.2140
134.2982
132.3225
129.2657
126.0321
124.3351
122.1770
60.2735
35.2740
21.1861
14.2799
¹³C NMR (CDCl₃, 75 MHz)
f1 (ppm)

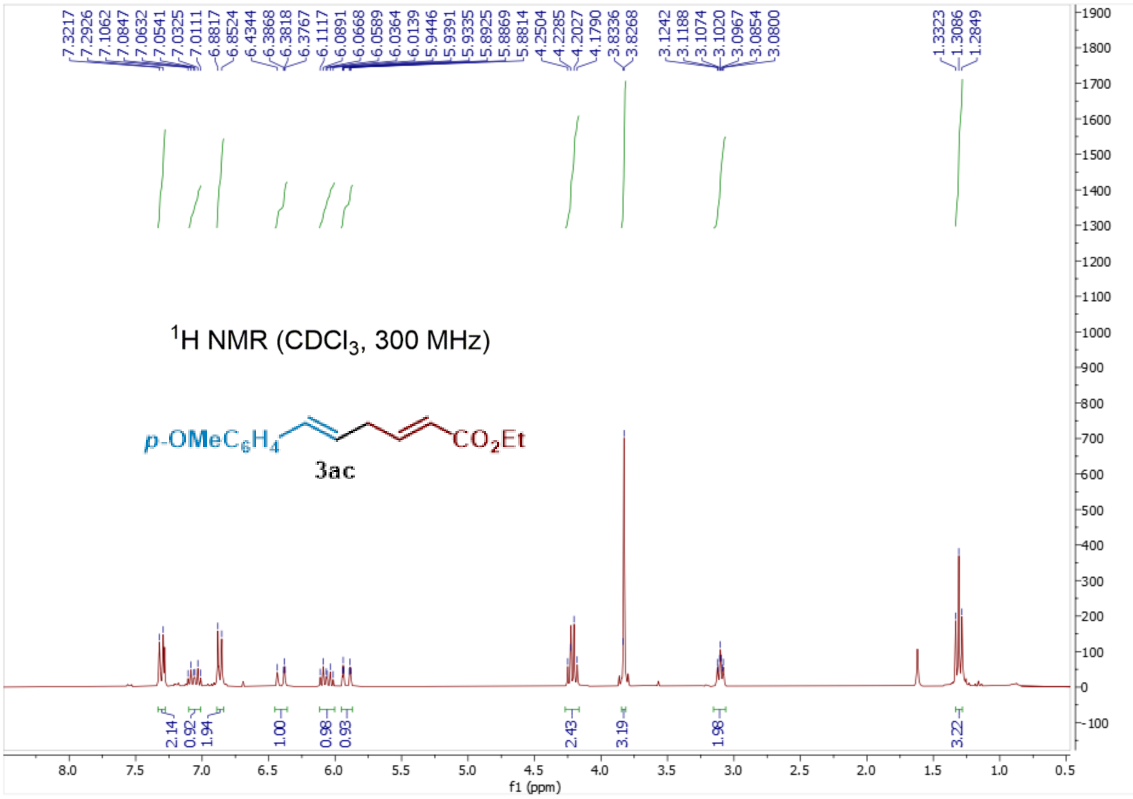
7.3217
7.2926
7.1062
7.0847
7.0632
7.0541
7.0325
7.0111
6.8817
6.8524
6.4344
6.3868
6.3818
6.3767
6.1117
6.0891
6.0668
6.0589
6.0364
6.0139
5.9446
5.9391
5.9335
5.8925
5.8869
5.8814
4.2504
4.2285
4.2027
4.1790
3.8336
3.8268
3.1242
3.1188
3.1074
3.1020
3.0967
3.0854
3.0800
1.3323
1.3086
1.2849
¹H NMR (CDCl₃, 300 MHz)
p-OMeC₆H₄
CO₂Et
3ac
2.14
0.92
1.94
1.00
0.98
0.93
2.43
3.19
1.98
3.22
f1 (ppm)

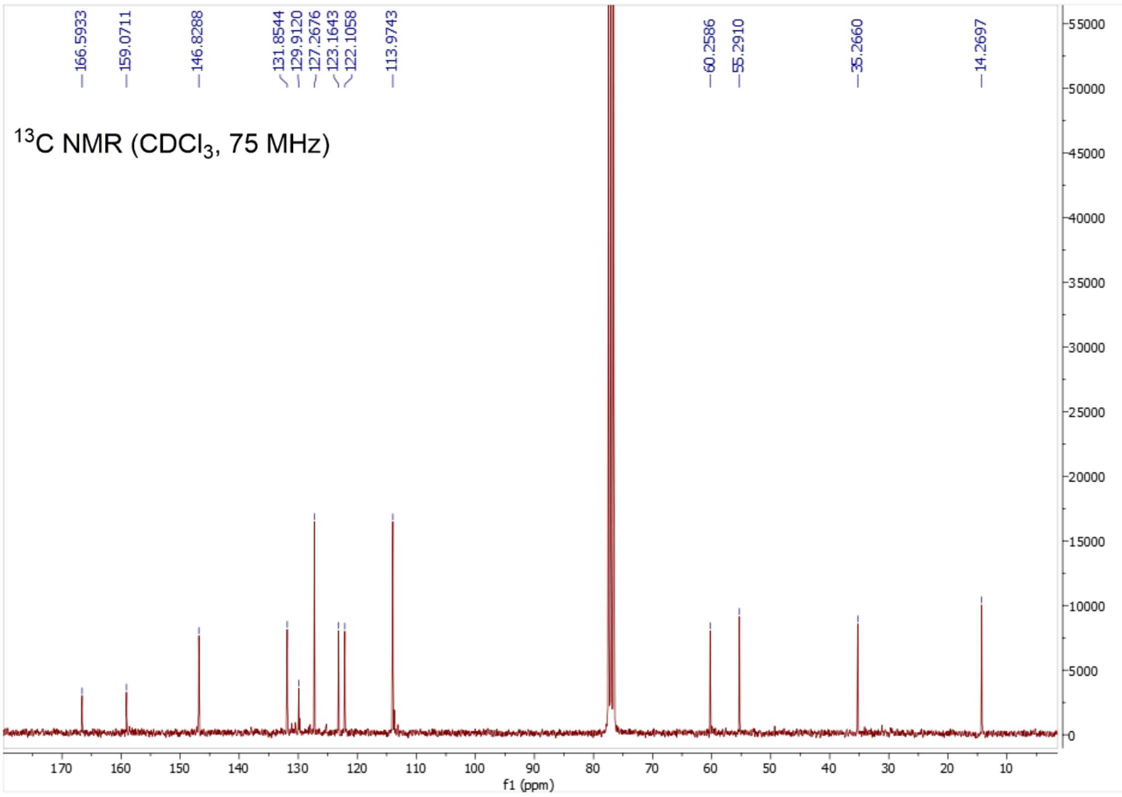
166.5933
159.0711
146.8288
131.8544
129.9120
127.2676
123.1643
122.1058
113.9743
60.2586
55.2910
35.2660
14.2697
¹³C NMR (CDCl₃, 75 MHz)
f1 (ppm)

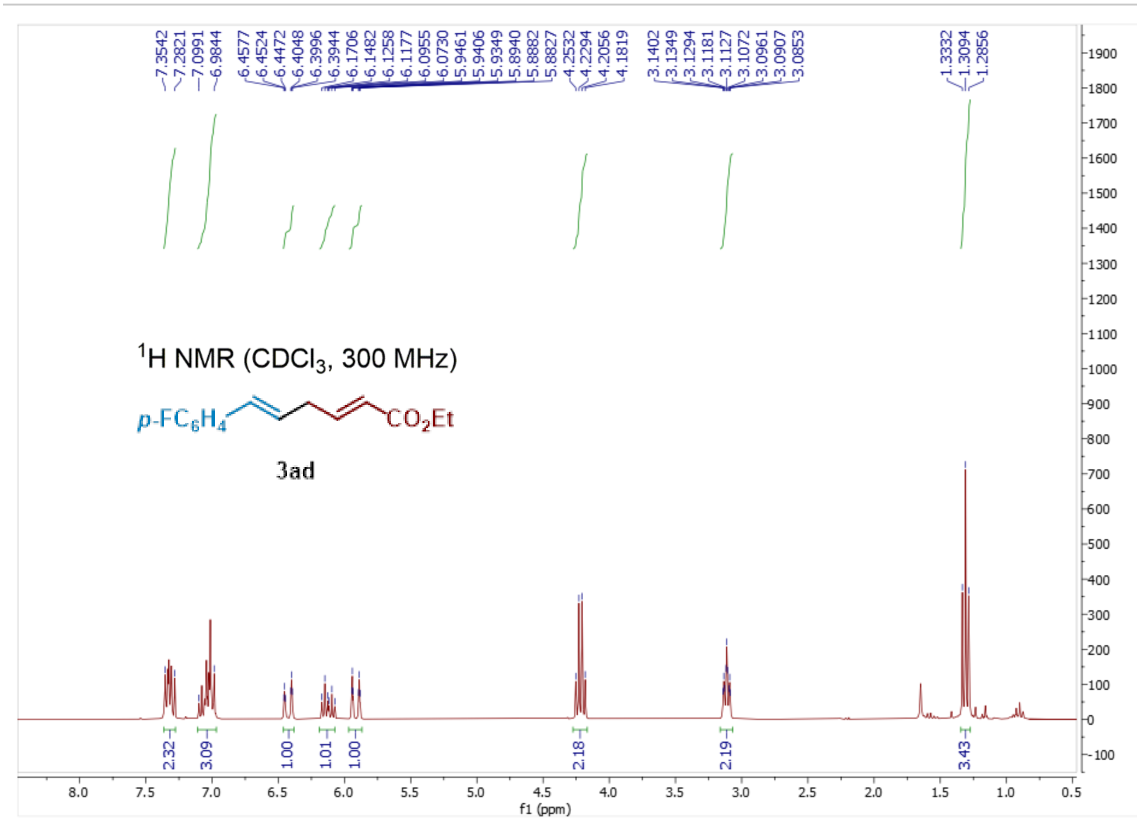

$^1$H NMR (CDCl$_3$, 300 MHz)
p-FC$_6$H$_4$ CO$_2$Et
3ad
7.3542 7.2821 7.0991 6.9844 6.4577 6.4524 6.4472 6.4048 6.3996 6.3944 6.1706 6.1482 6.1258 6.1177 6.0955 6.0730 5.9461 5.9406 5.9349 5.8940 5.8882 5.8827 4.2532 4.2294 4.2056 4.1819 3.1402 3.1349 3.1294 3.1181 3.1127 3.1072 3.0961 3.0907 3.0853 1.3332 1.3094 1.2856
2.32 3.09 1.00 1.01 1.00 2.18 2.19 3.43
f1 (ppm)


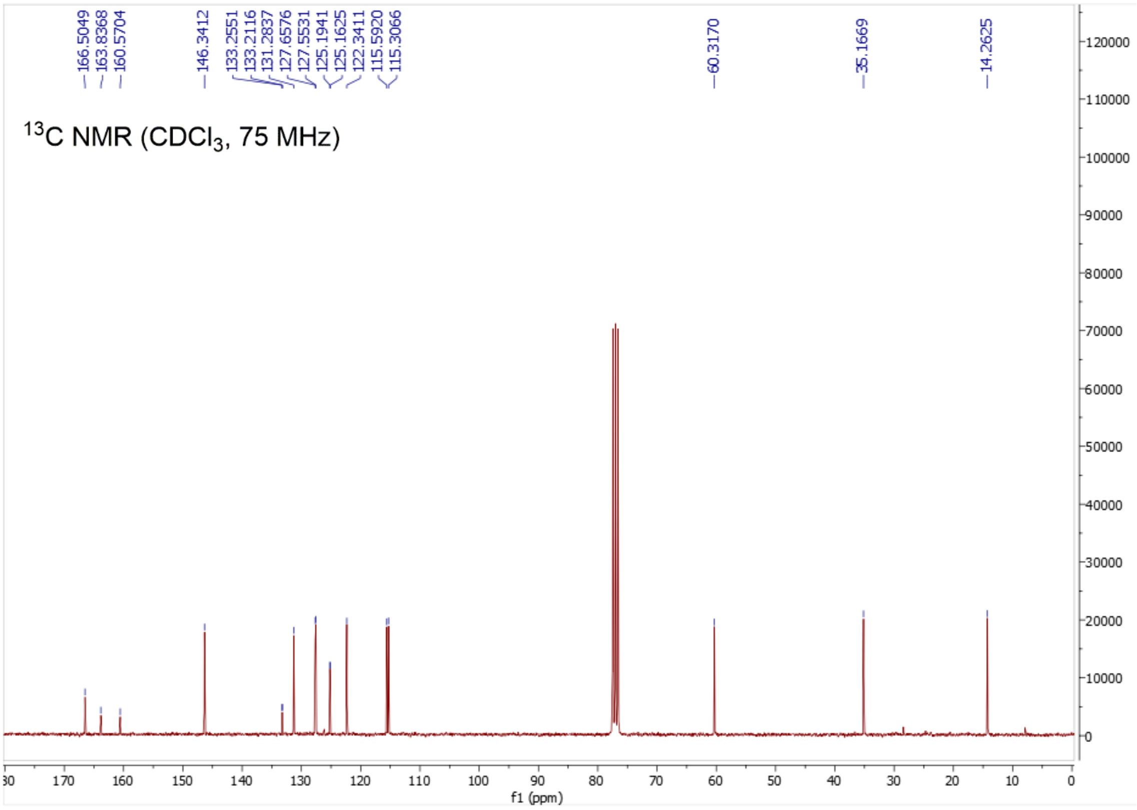

$^{13}$C NMR (CDCl$_3$, 75 MHz)
166.5049 163.8368 160.5704 146.3412 133.2551 133.2116 131.2837 127.6576 127.5531 125.1941 125.1625 122.3411 115.5920 115.3066 60.3170 35.1669 14.2625
f1 (ppm)

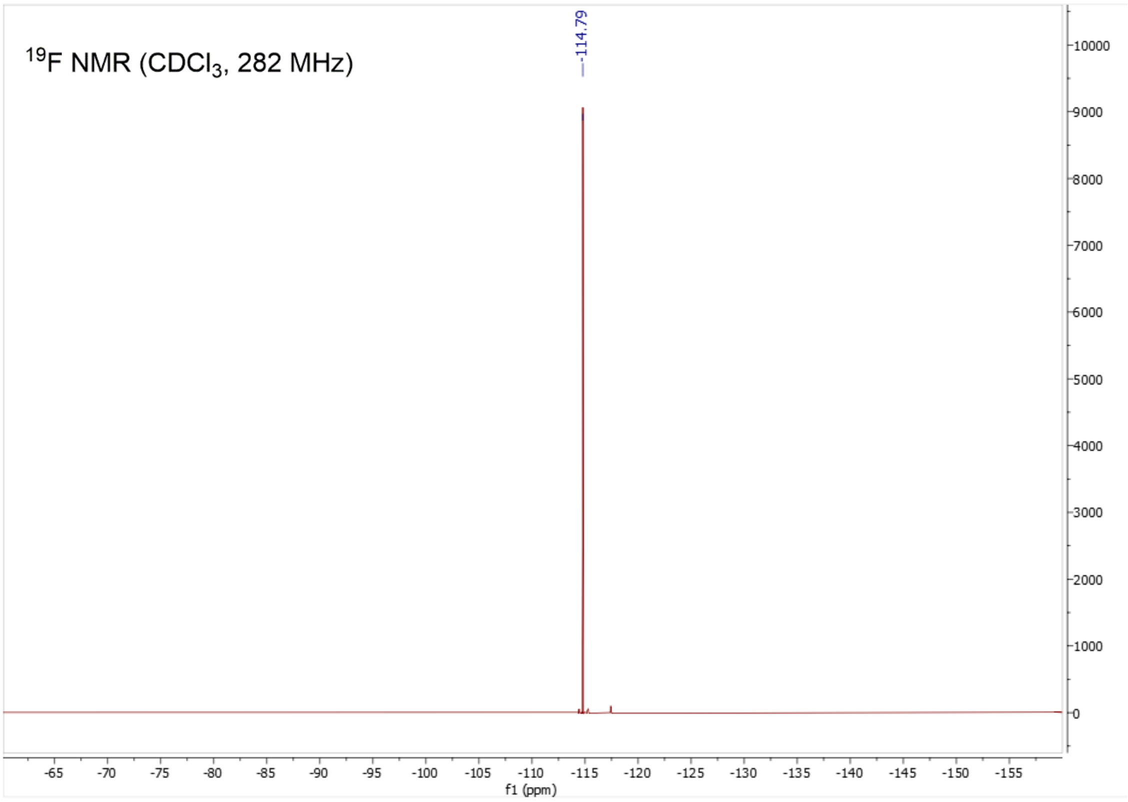

19F NMR (CDCl3, 282 MHz)
-114.79
f1 (ppm)

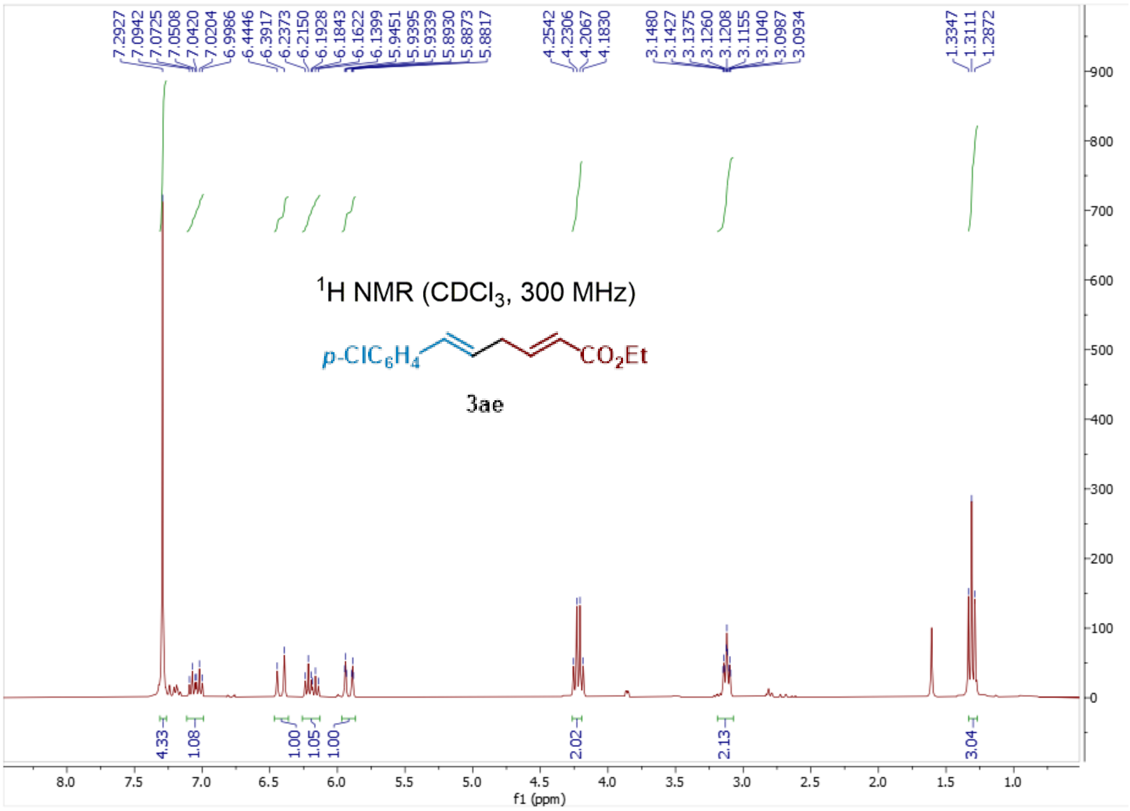

7.2927
7.0942
7.0725
7.0508
7.0420
7.0204
6.9986
6.4446
6.3917
6.2373
6.2150
6.1928
6.1843
6.1622
6.1399
5.9451
5.9395
5.9339
5.8930
5.8873
5.8817
4.2542
4.2306
4.2067
4.1830
3.1480
3.1427
3.1375
3.1260
3.1208
3.1155
3.1040
3.0987
3.0934
1.3347
1.3111
1.2872
$^1$H NMR ($CDCl_3$, 300 MHz)
p-ClC$_6$H$_4$
CO$_2$Et
3ae
4.33
1.08
1.00
1.05
1.00
2.02
2.13
3.04
f1 (ppm)


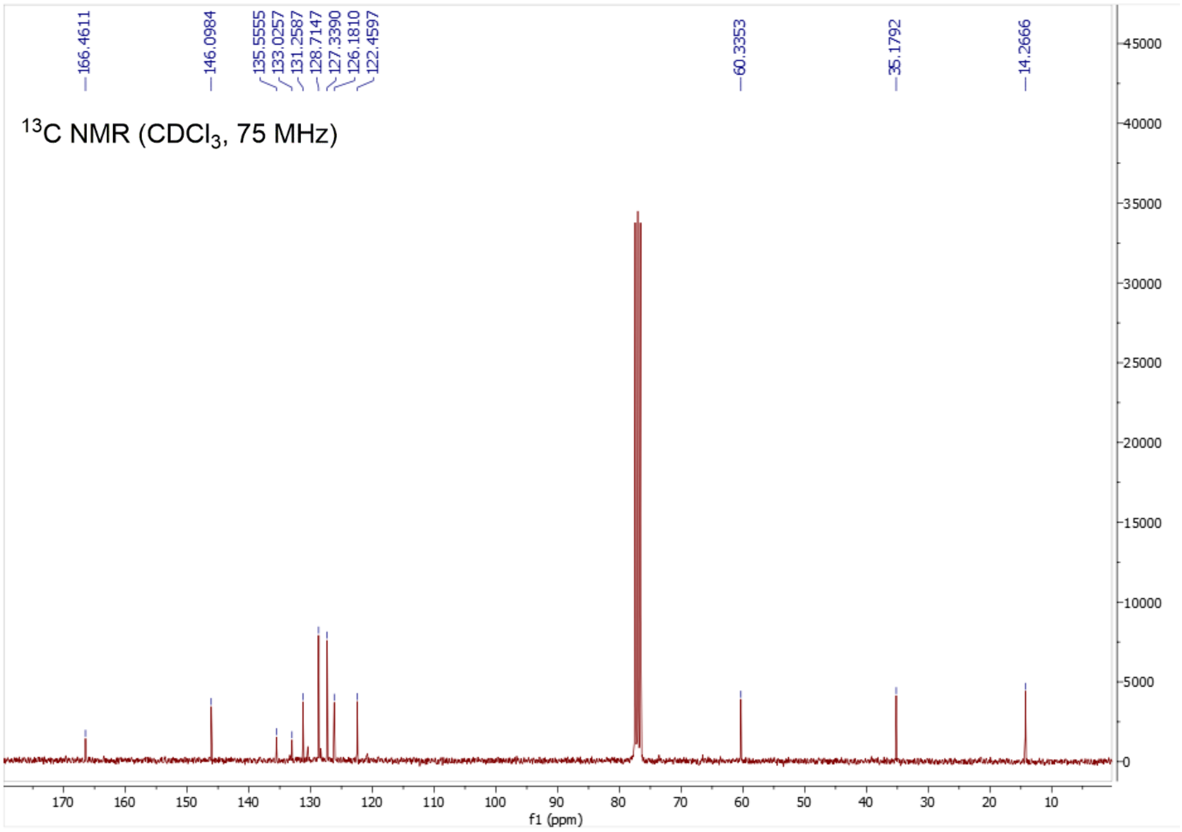

166.4611
146.0984
135.5555
133.0257
131.2587
128.7147
127.3390
126.1810
122.4597
60.3353
35.1792
14.2666
$^{13}$C NMR ($CDCl_3$, 75 MHz)
f1 (ppm)

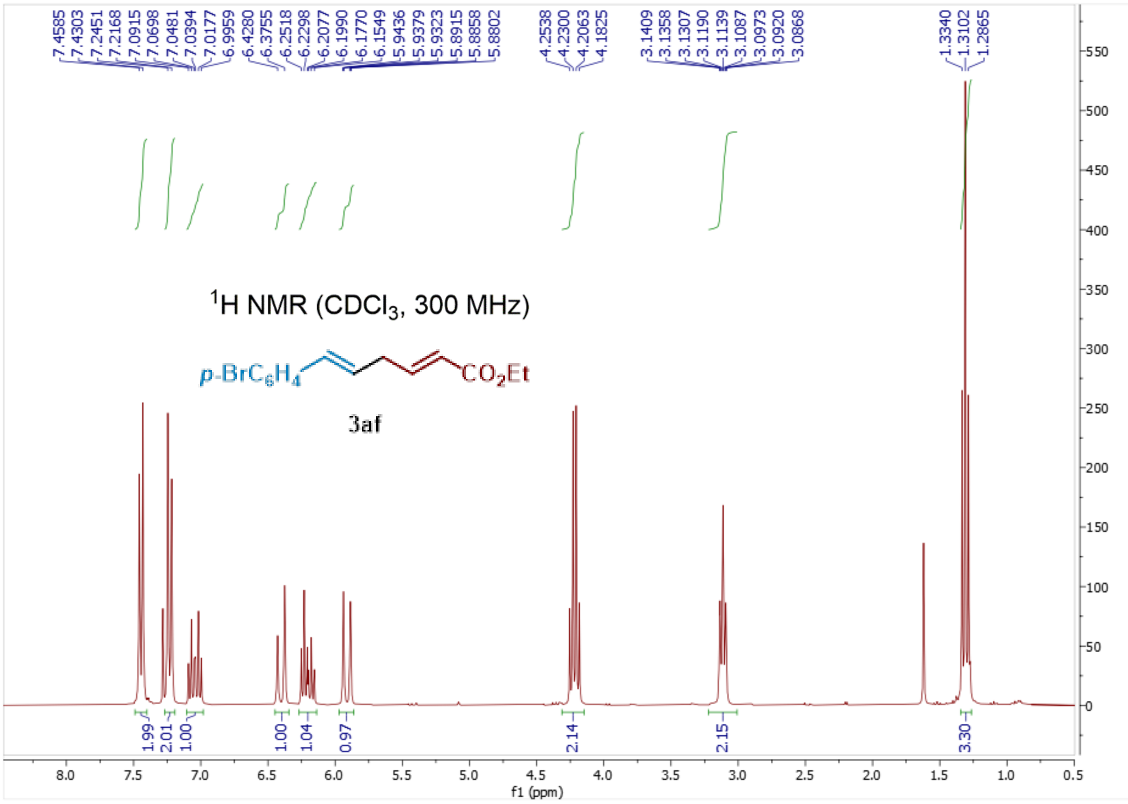

7.4585
7.4303
7.2451
7.2168
7.0915
7.0698
7.0481
7.0394
7.0177
6.9959
6.4280
6.3755
6.2518
6.2298
6.2077
6.1990
6.1770
6.1549
5.9436
5.9379
5.9323
5.8915
5.8858
5.8802
4.2538
4.2300
4.2063
4.1825
3.1409
3.1358
3.1307
3.1190
3.1139
3.1087
3.0973
3.0920
3.0868
1.3340
1.3102
1.2865
1H NMR (CDCl3, 300 MHz)
p-BrC6H4
CO2Et
3af
1.99
2.01
1.00
1.00
1.04
0.97
2.14
2.15
3.30
f1 (ppm)


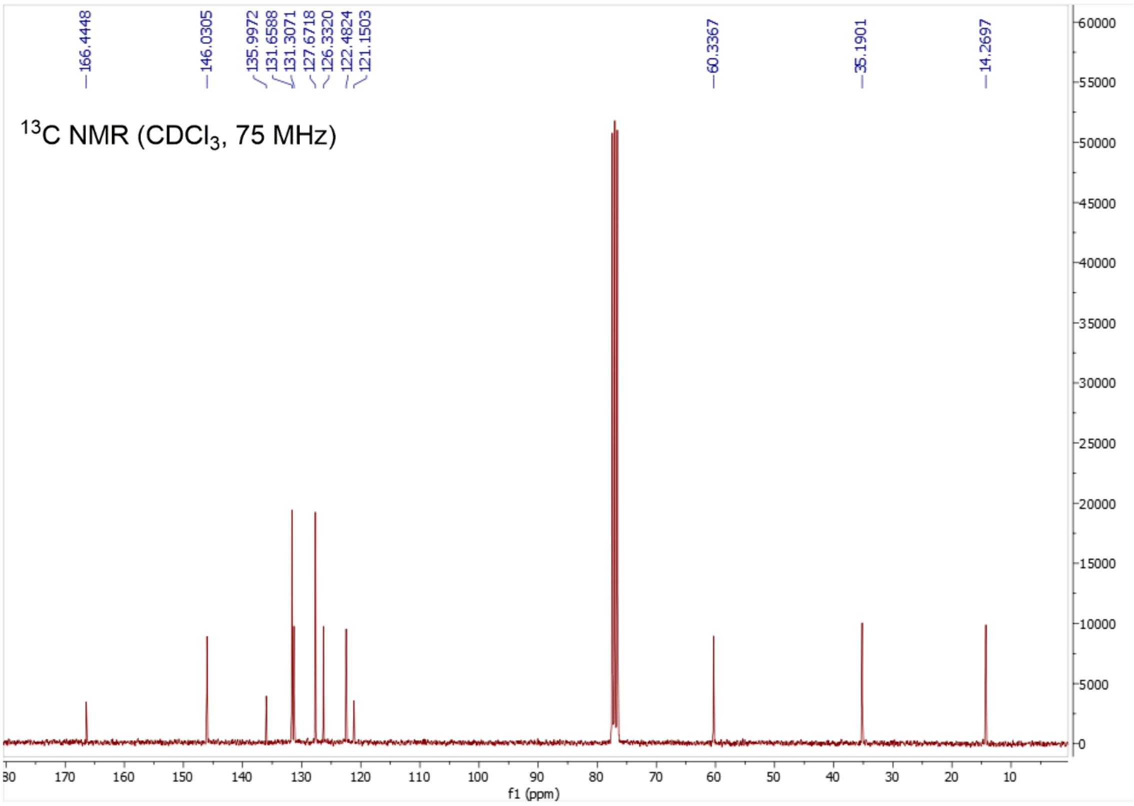

166.4448
146.0305
135.9972
131.6588
131.3071
127.6718
126.3320
122.4824
121.1503
60.3367
35.1901
14.2697
13C NMR (CDCl3, 75 MHz)
f1 (ppm)

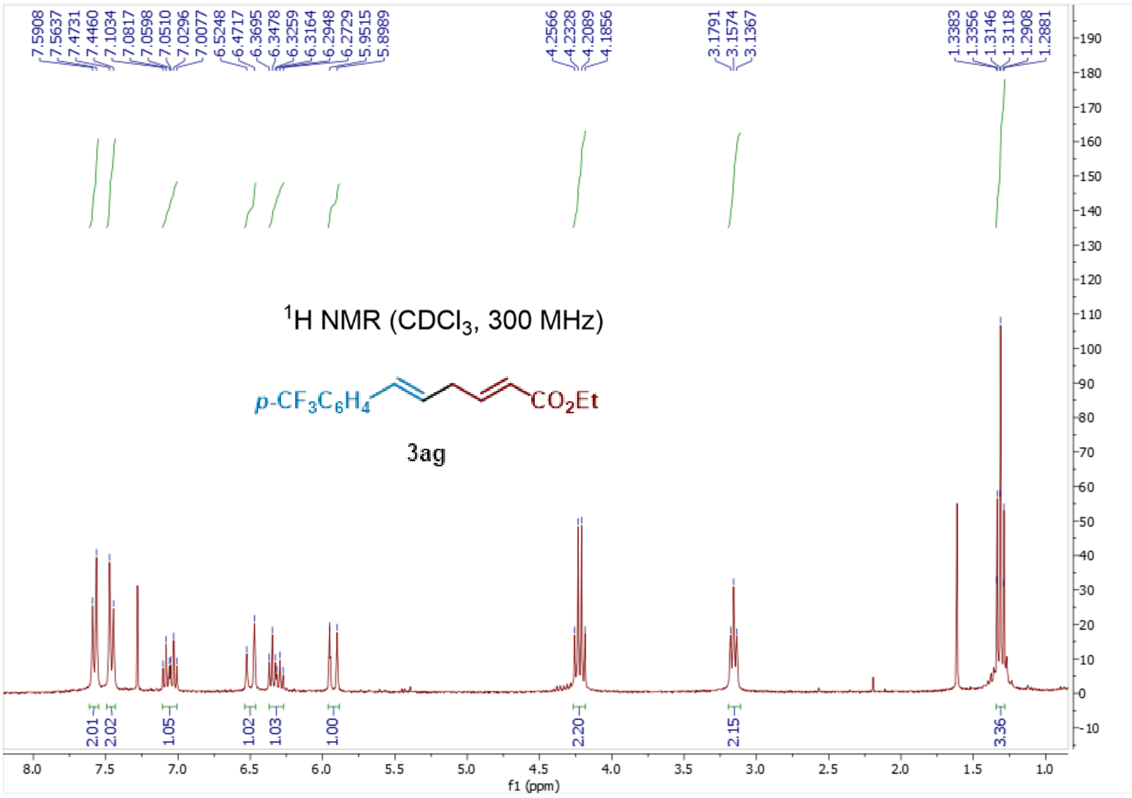
1H NMR (CDCl3, 300 MHz)
p-CF3C6H4
CO2Et
3ag
7.5908 7.5637 7.4731 7.4460 7.1034 7.0817 7.0598 7.0510 7.0296 7.0077 6.5248 6.4717 6.3695 6.3478 6.3259 6.3164 6.2948 6.2729 5.9515 5.8989
4.2566 4.2328 4.2089 4.1856
3.1791 3.1574 3.1367
1.3383 1.3356 1.3146 1.3118 1.2908 1.2881
2.01 2.02 1.05 1.02 1.03 1.00 2.20 2.15 3.36
f1 (ppm)

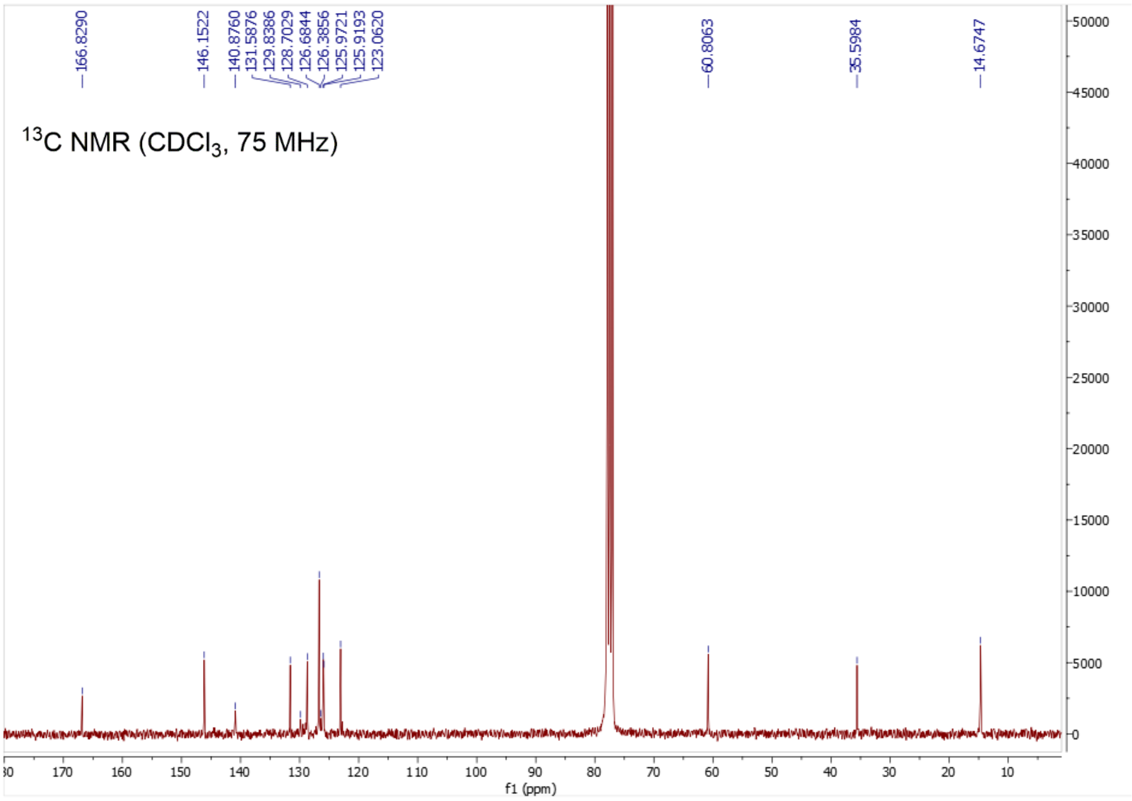
13C NMR (CDCl3, 75 MHz)
166.8290
146.1522
140.8760 131.5876 129.8386 128.7029 126.6844 126.3856 125.9721 125.9193 123.0620
60.8063
35.5984
14.6747
f1 (ppm)

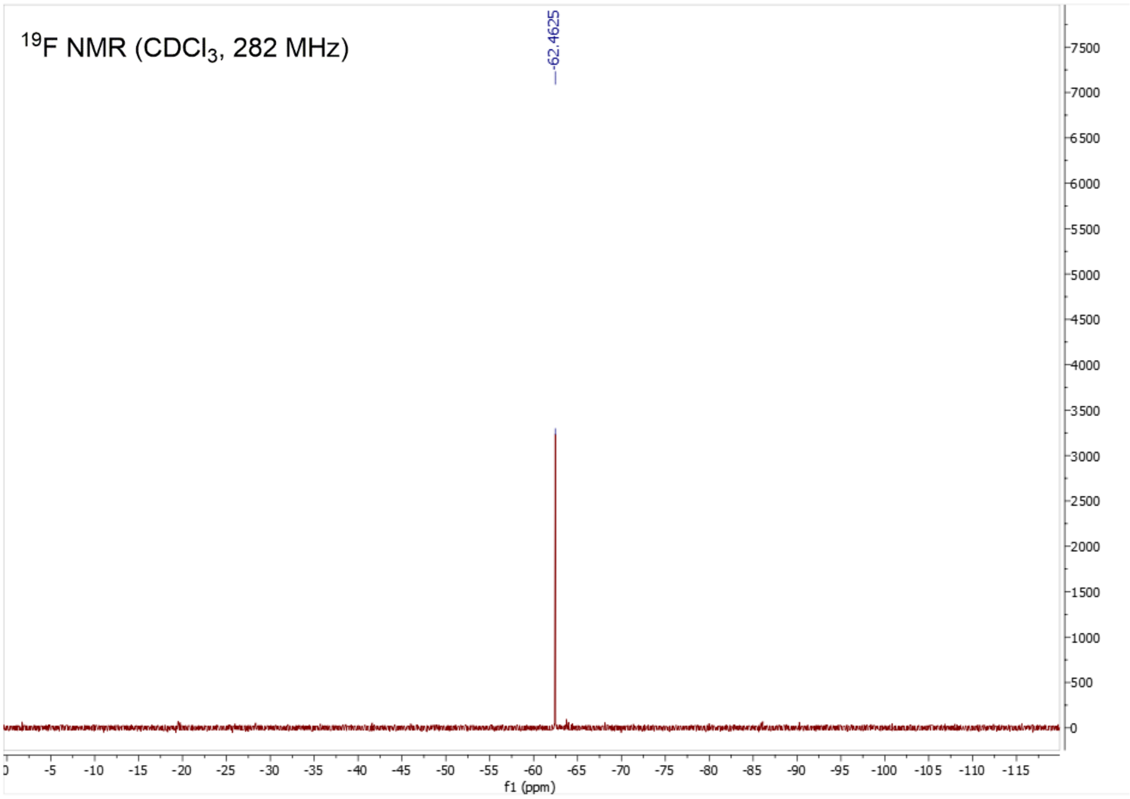

$^{19}F$ NMR ($CDCl_3$, 282 MHz)
-62.4625
f1 (ppm)

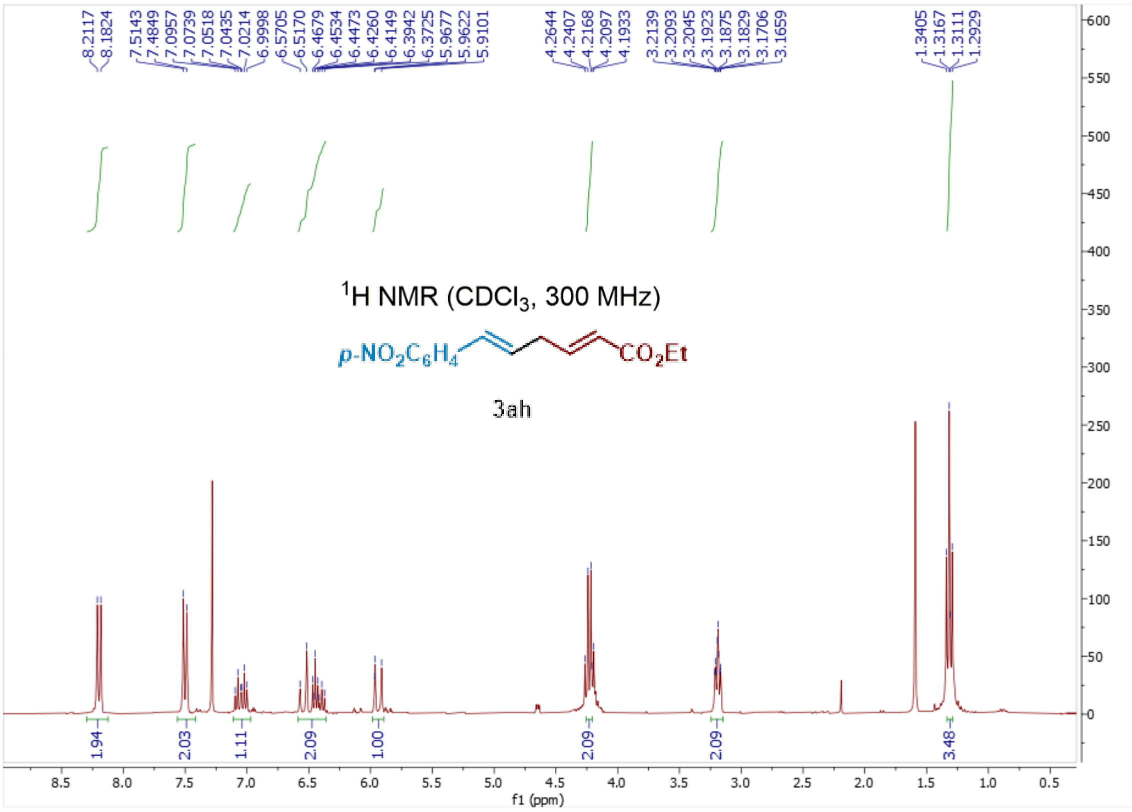
1H NMR (CDCl3, 300 MHz)
p-NO2C6H4
CO2Et
3ah
f1 (ppm)

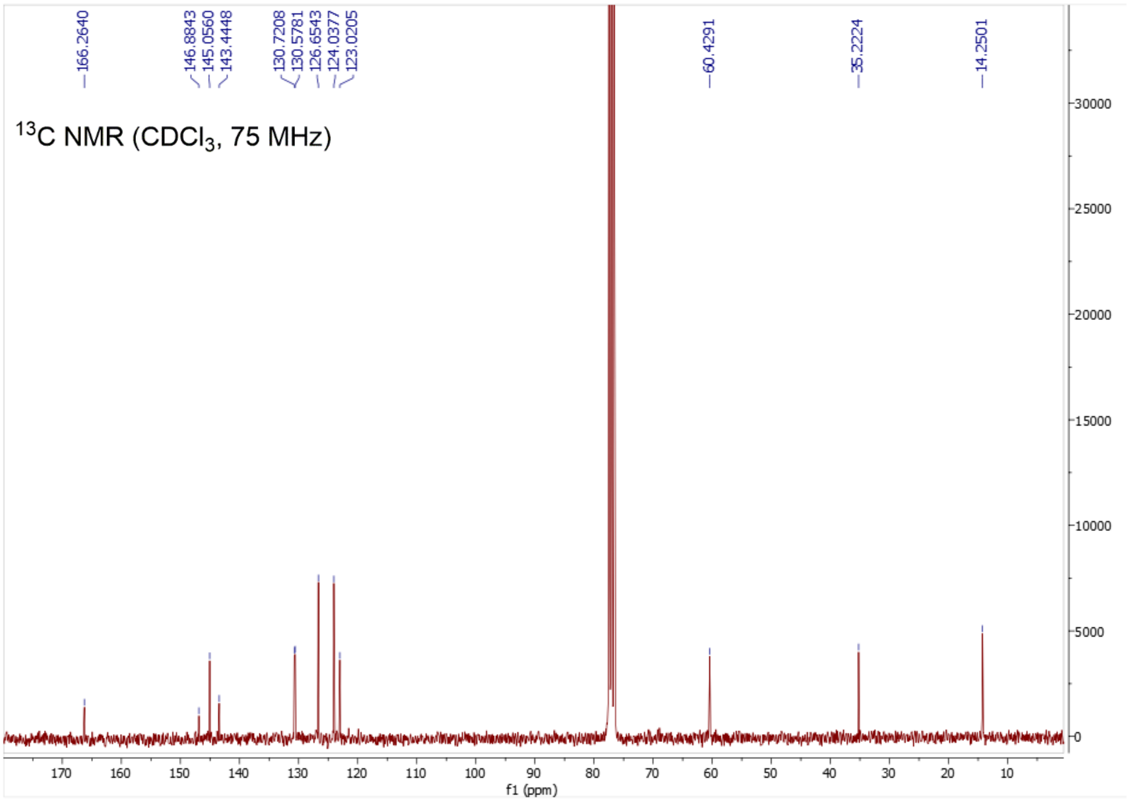
13C NMR (CDCl3, 75 MHz)
f1 (ppm)

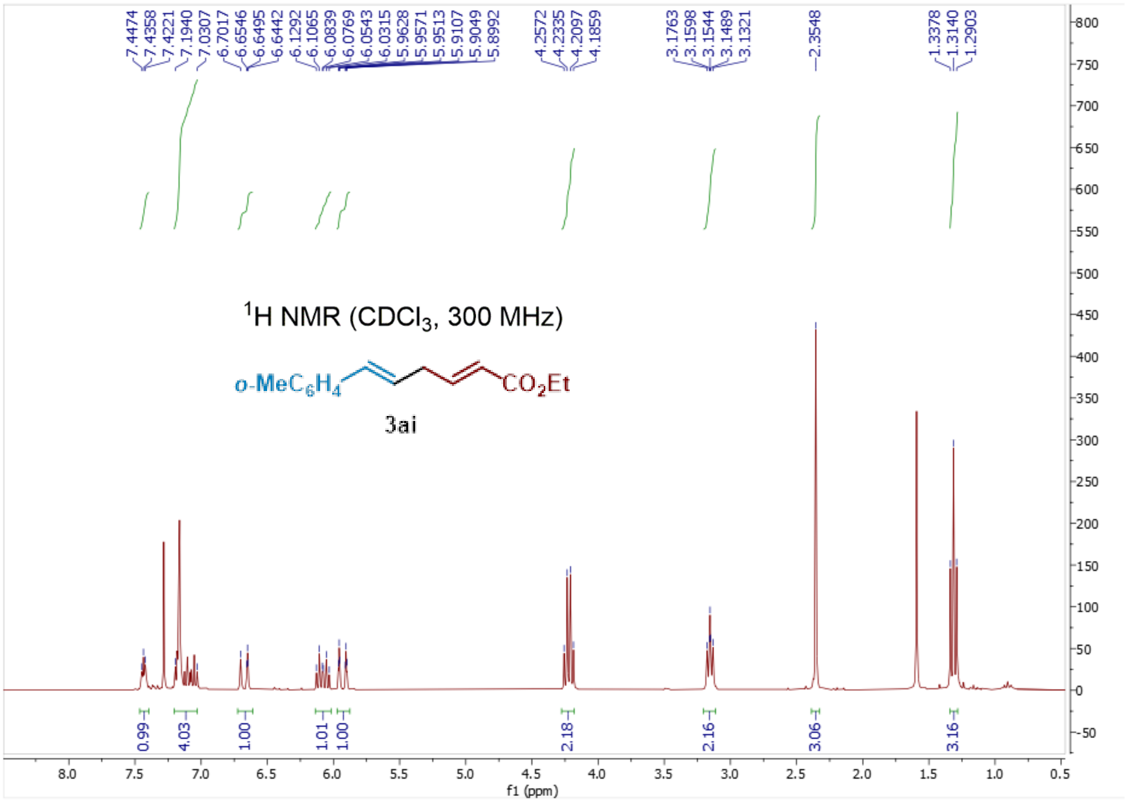

1H NMR (CDCl3, 300 MHz)
o-MeC6H4
CO2Et
3ai
f1 (ppm)


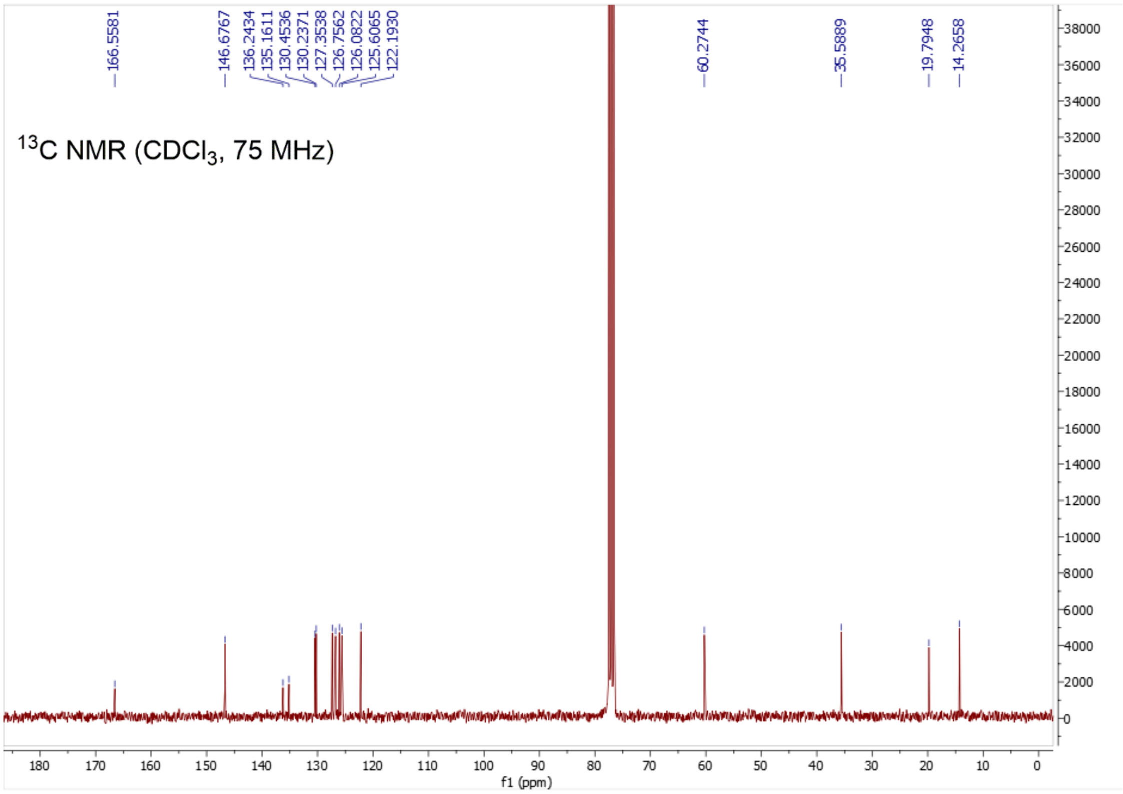

13C NMR (CDCl3, 75 MHz)
f1 (ppm)

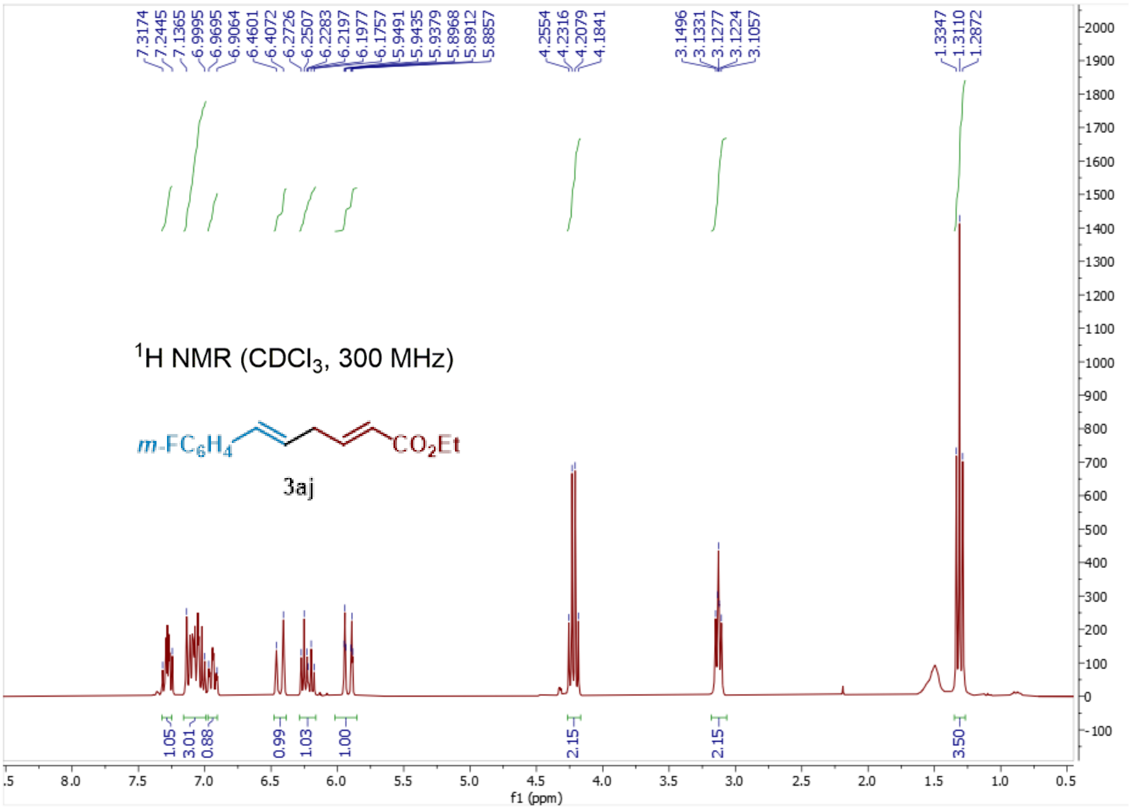
7.3174
7.2445
7.1365
6.9995
6.9695
6.9064
6.4601
6.4072
6.2726
6.2507
6.2283
6.2197
6.1977
6.1757
5.9491
5.9435
5.9379
5.8968
5.8912
5.8857
4.2554
4.2316
4.2079
4.1841
3.1496
3.1331
3.1277
3.1224
3.1057
1.3347
1.3110
1.2872
$^{1}$H NMR ($CDCl_3$, 300 MHz)
$m$-$FC_6H_4$
$CO_2Et$
3aj
1.05
3.01
0.88
0.99
1.03
1.00
2.15
2.15
3.50
f1 (ppm)

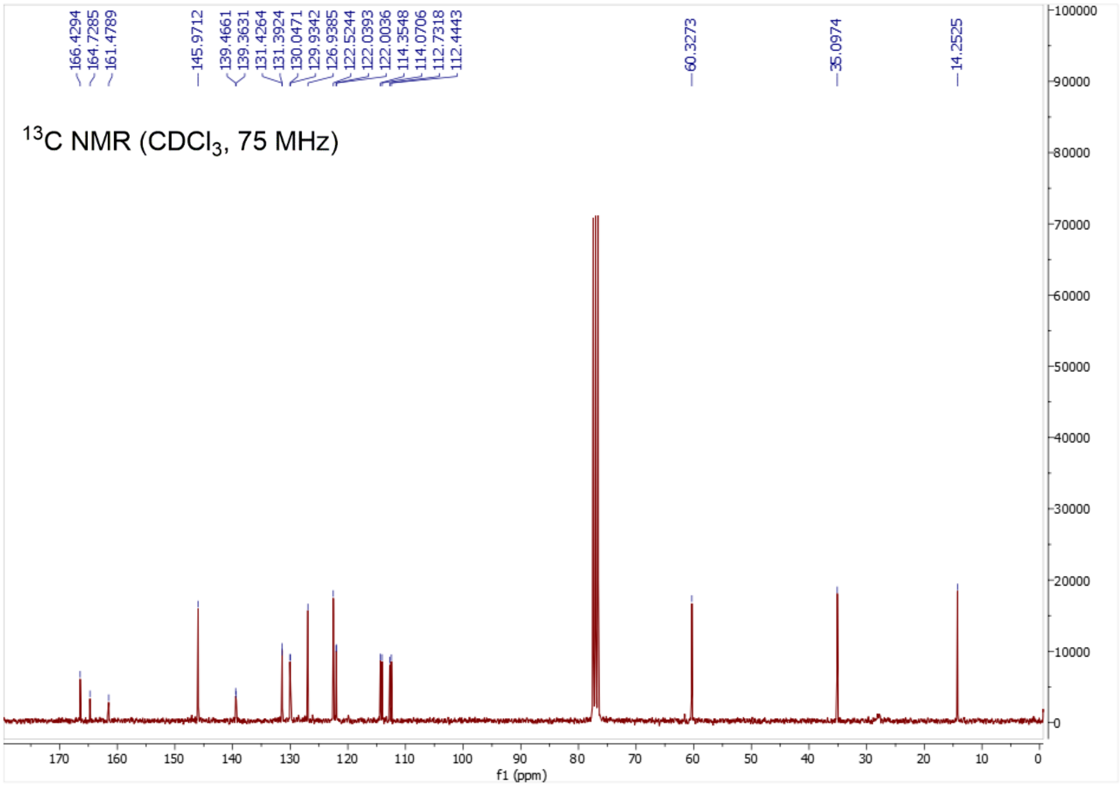
166.4294
164.7285
161.4789
145.9712
139.4661
139.3631
131.4264
131.3924
130.0471
129.9342
126.9385
122.5244
122.0393
122.0036
114.3548
114.0706
112.7318
112.4443
60.3273
35.0974
14.2525
$^{13}$C NMR ($CDCl_3$, 75 MHz)
f1 (ppm)

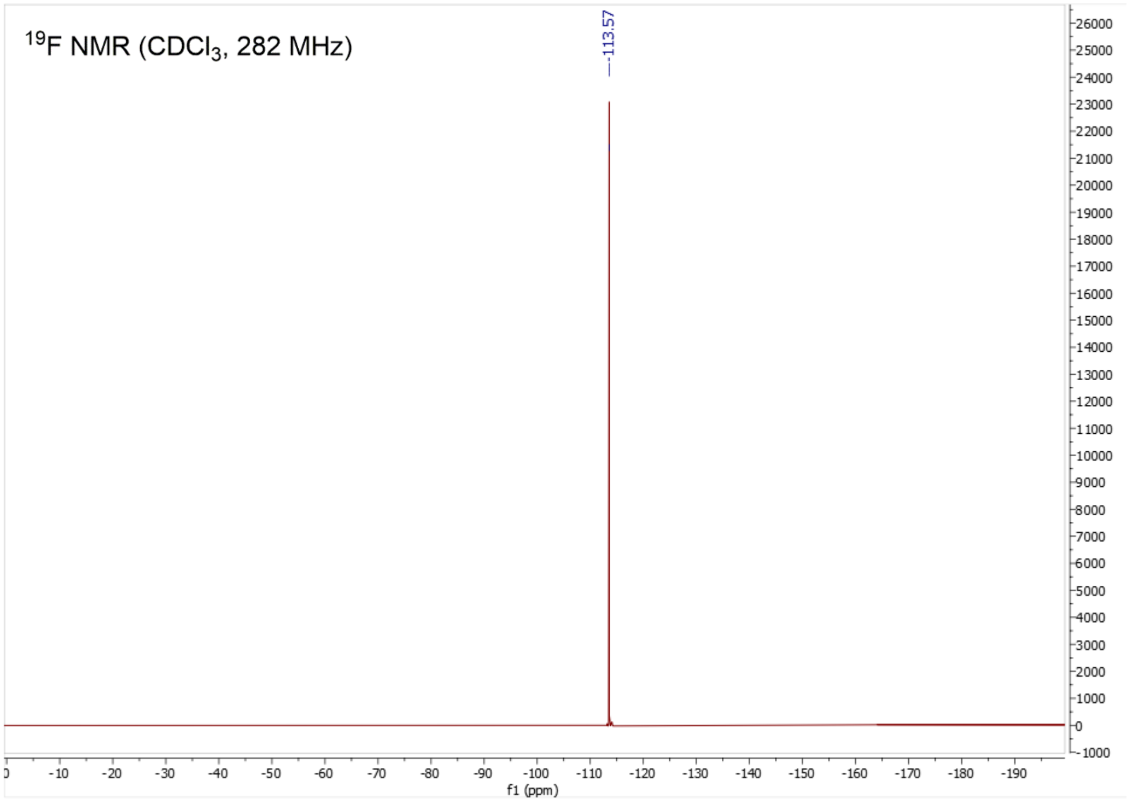

$^{19}F$ NMR ($CDCl_3$, 282 MHz)
-113.57
f1 (ppm)

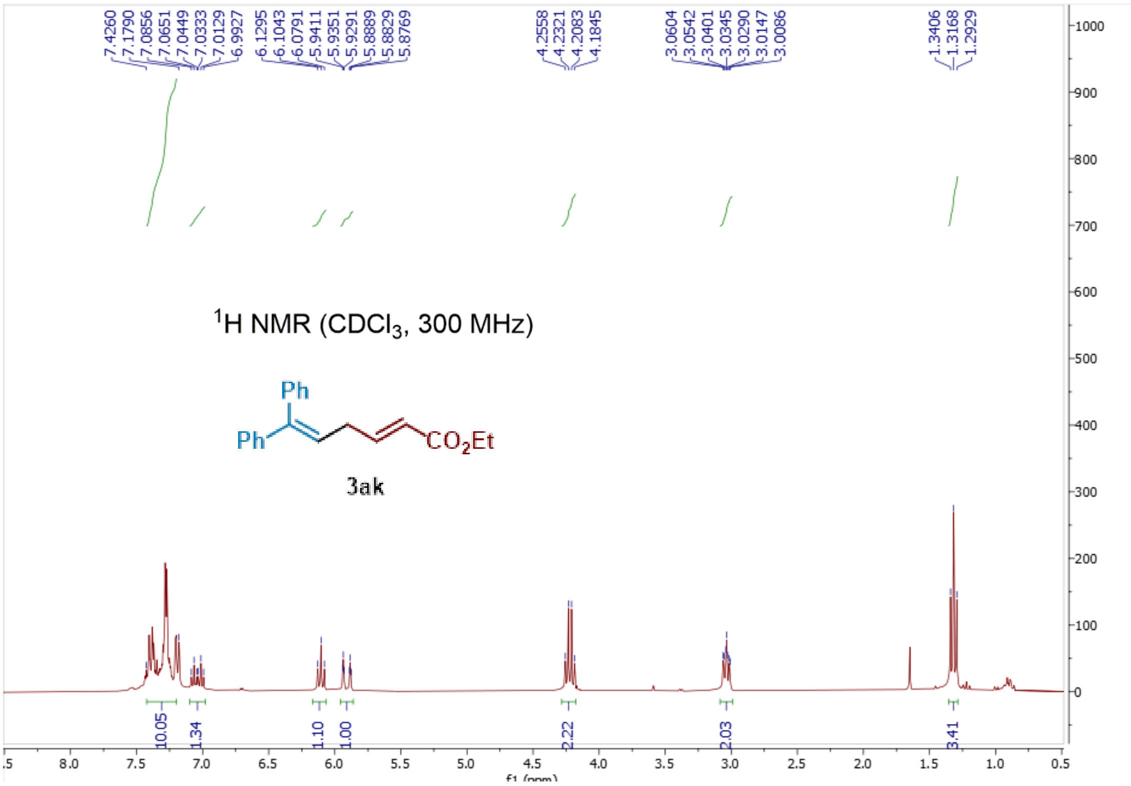
7.4260
7.1790
7.0856
7.0651
7.0449
7.0333
7.0129
6.9927
6.1295
6.1043
6.0791
5.9411
5.9351
5.9291
5.8889
5.8829
5.8769
4.2558
4.2321
4.2083
4.1845
3.0604
3.0542
3.0401
3.0345
3.0290
3.0147
3.0086
1.3406
1.3168
1.2929
$^1$H NMR (CDCl$_3$, 300 MHz)
Ph
Ph
CO$_2$Et
3ak
10.05
1.34
1.10
1.00
2.22
2.03
3.41
f1 (ppm)

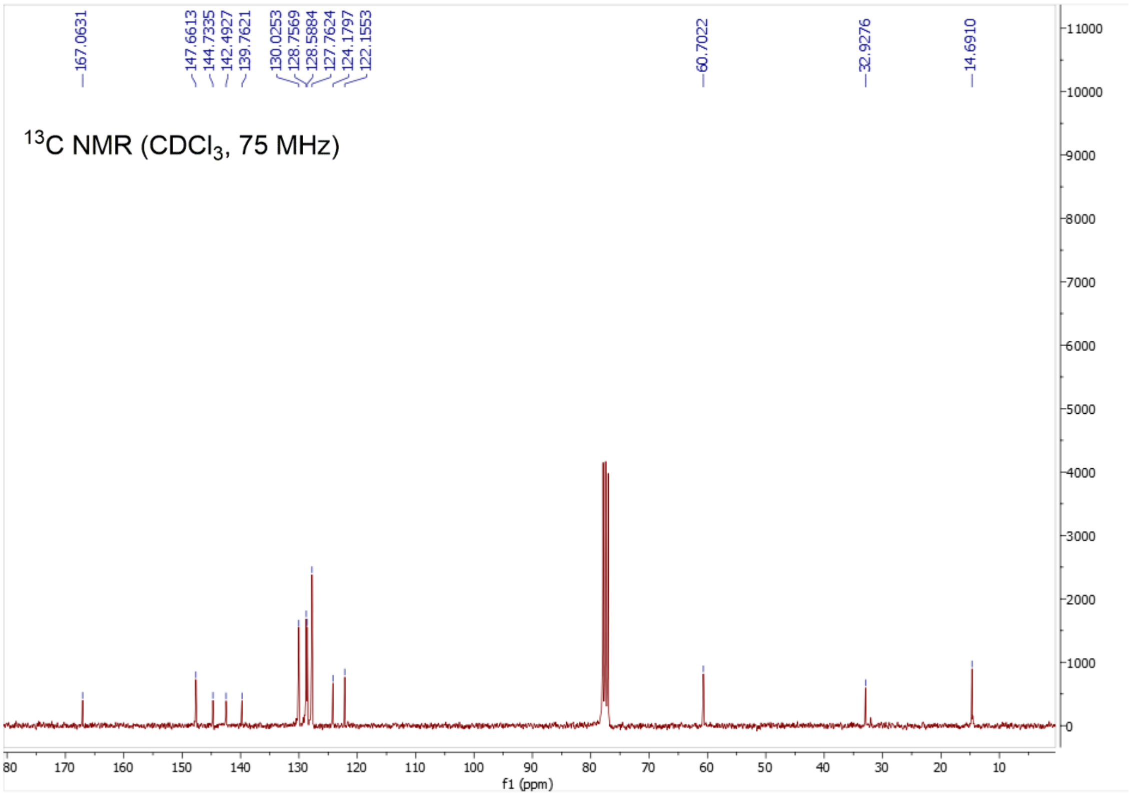
167.0631
147.6613
144.7335
142.4927
139.7621
130.0253
128.7569
128.5884
127.7624
124.1797
122.1553
60.7022
32.9276
14.6910
$^{13}$C NMR (CDCl$_3$, 75 MHz)
f1 (ppm)

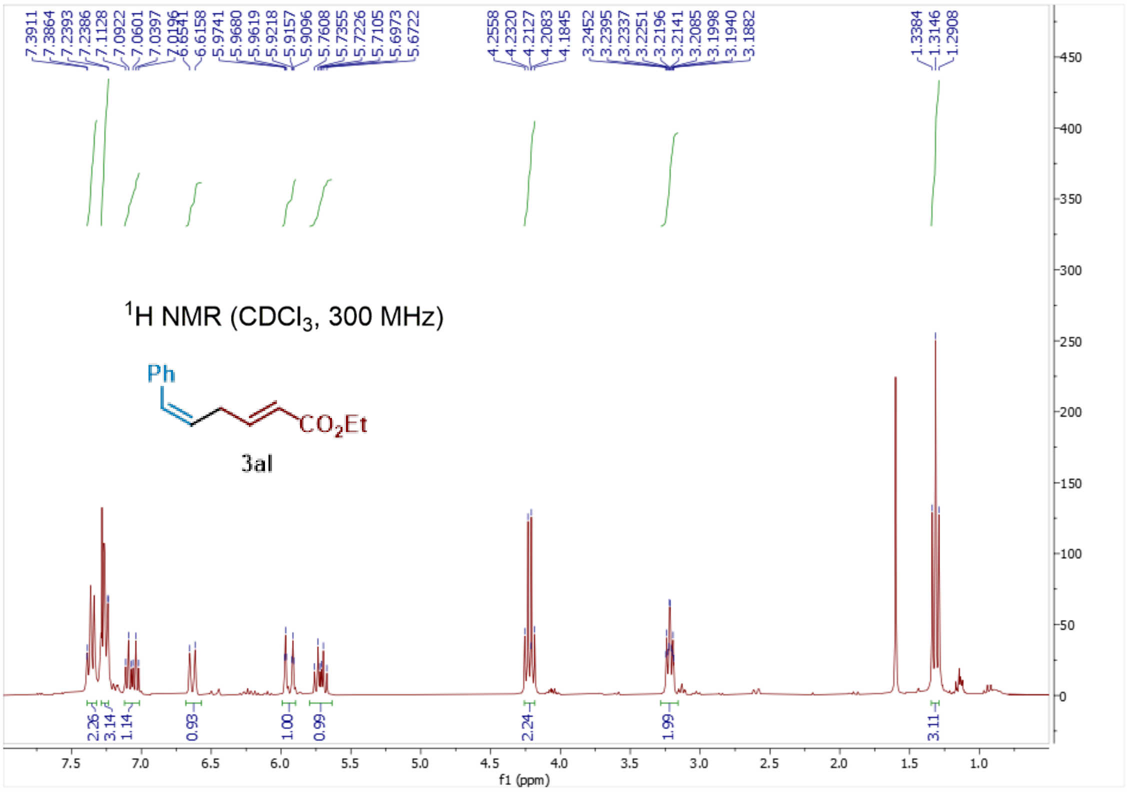
1H NMR (CDCl3, 300 MHz)
Ph
CO2Et
3al
f1 (ppm)

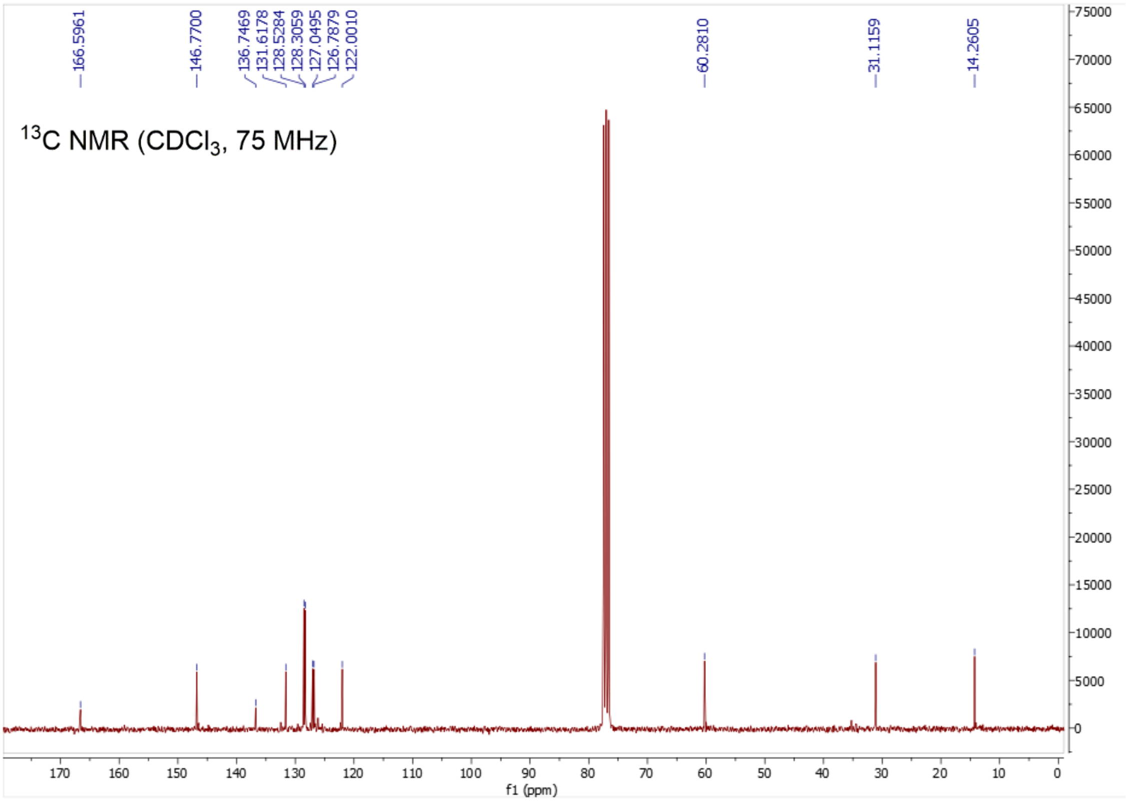
13C NMR (CDCl3, 75 MHz)
166.5961
146.7700
136.7469
131.6178
128.5284
128.3059
127.0495
126.7879
122.0010
60.2810
31.1159
14.2605
f1 (ppm)

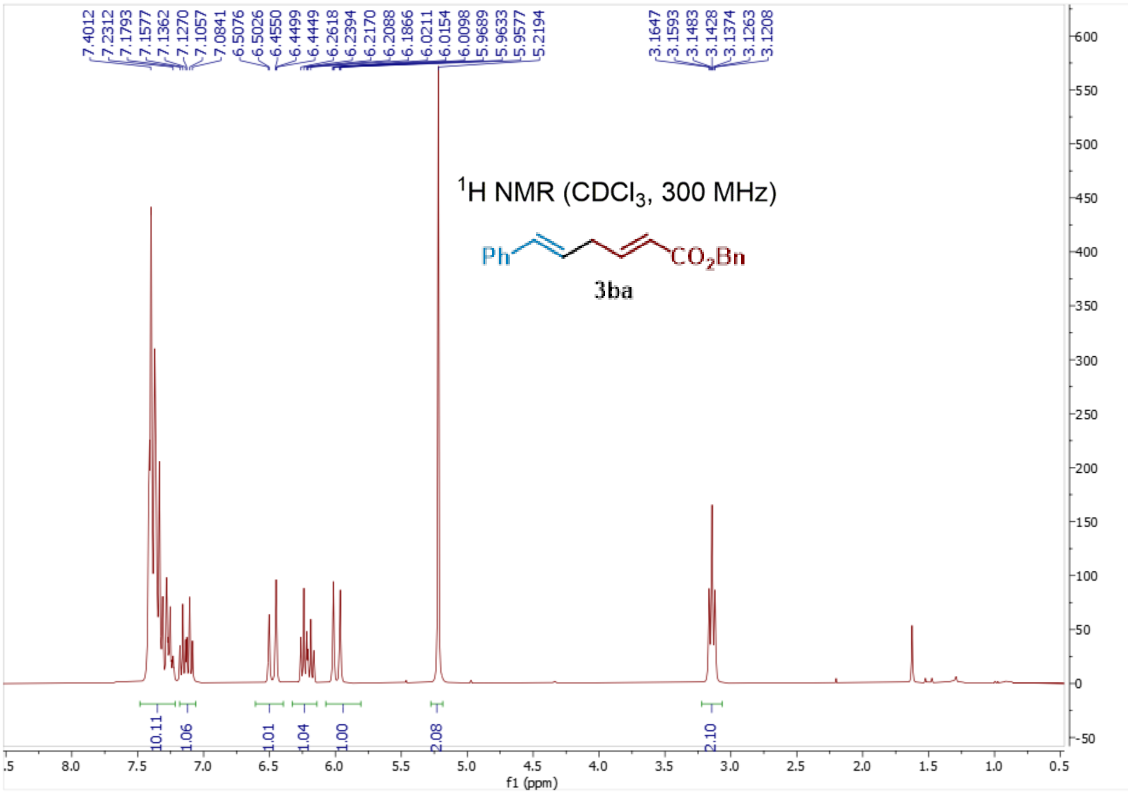
1H NMR (CDCl3, 300 MHz)
Ph
CO2Bn
3ba
7.4012
7.2312
7.1793
7.1577
7.1362
7.1270
7.1057
7.0841
6.5076
6.5026
6.4550
6.4499
6.4449
6.2618
6.2394
6.2170
6.2088
6.1866
6.0211
6.0154
6.0098
5.9689
5.9633
5.9577
5.2194
3.1647
3.1593
3.1483
3.1428
3.1374
3.1263
3.1208
10.11
1.06
1.01
1.04
1.00
2.08
2.10
f1 (ppm)

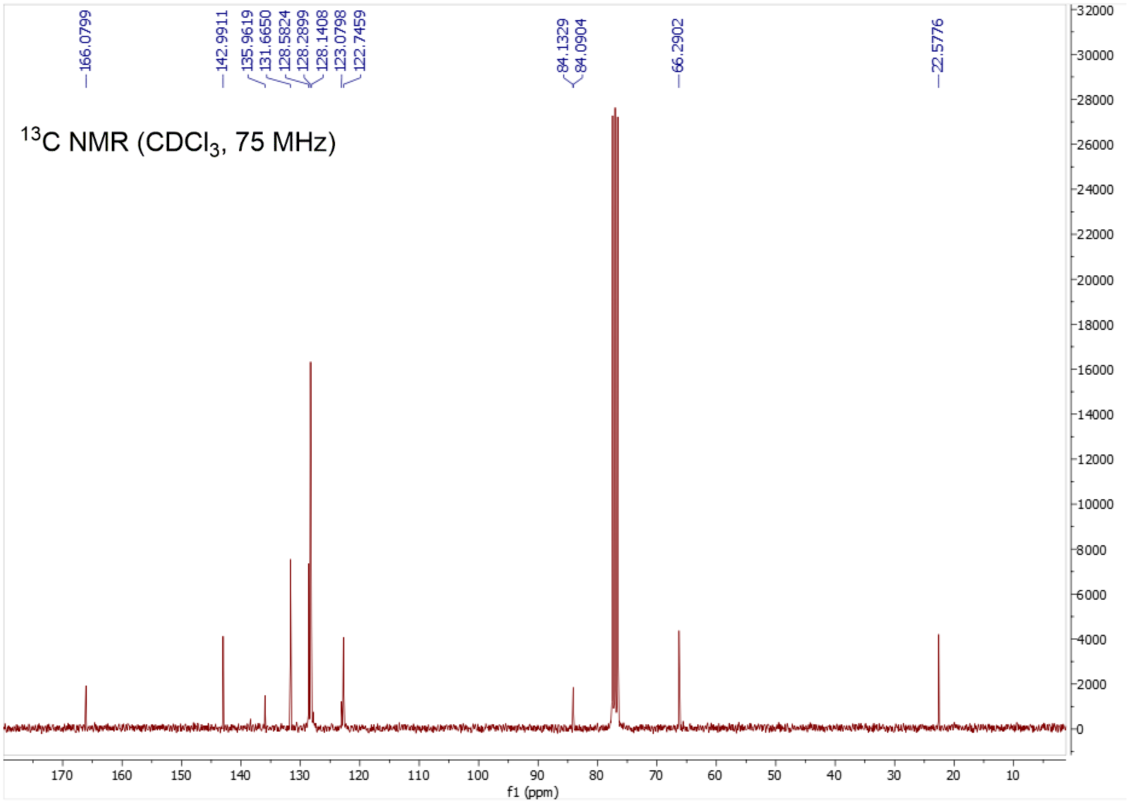
13C NMR (CDCl3, 75 MHz)
166.0799
142.9911
135.9619
131.6650
128.5824
128.2899
128.1408
123.0798
122.7459
84.1329
84.0904
66.2902
22.5776
f1 (ppm)

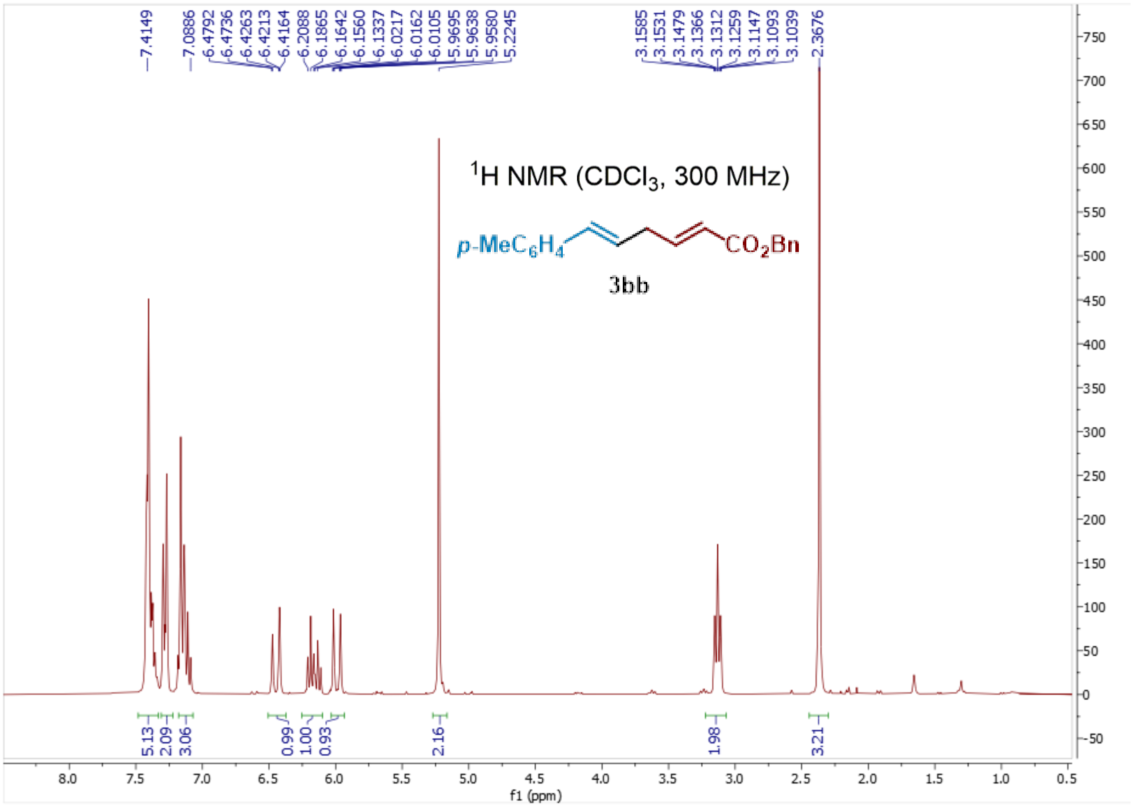

$^1$H NMR (CDCl$_3$, 300 MHz)
p-MeC$_6$H$_4$
CO$_2$Bn
3bb
f1 (ppm)


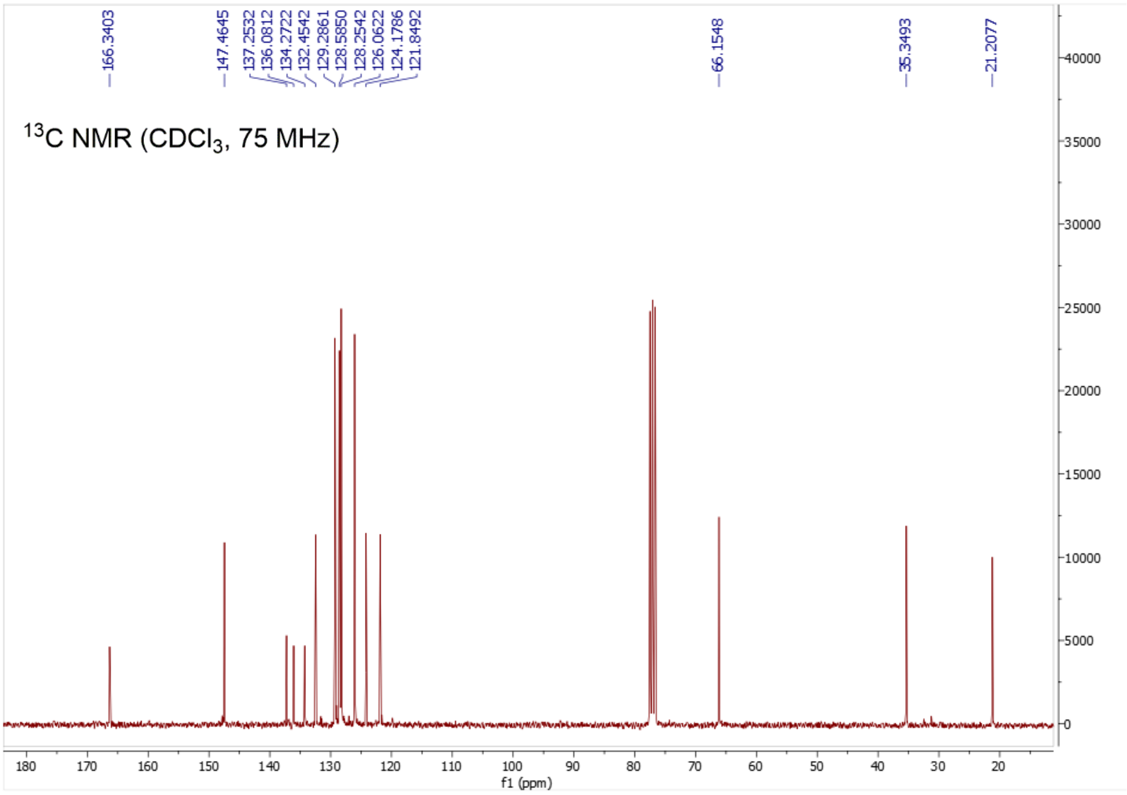

$^{13}$C NMR (CDCl$_3$, 75 MHz)
f1 (ppm)

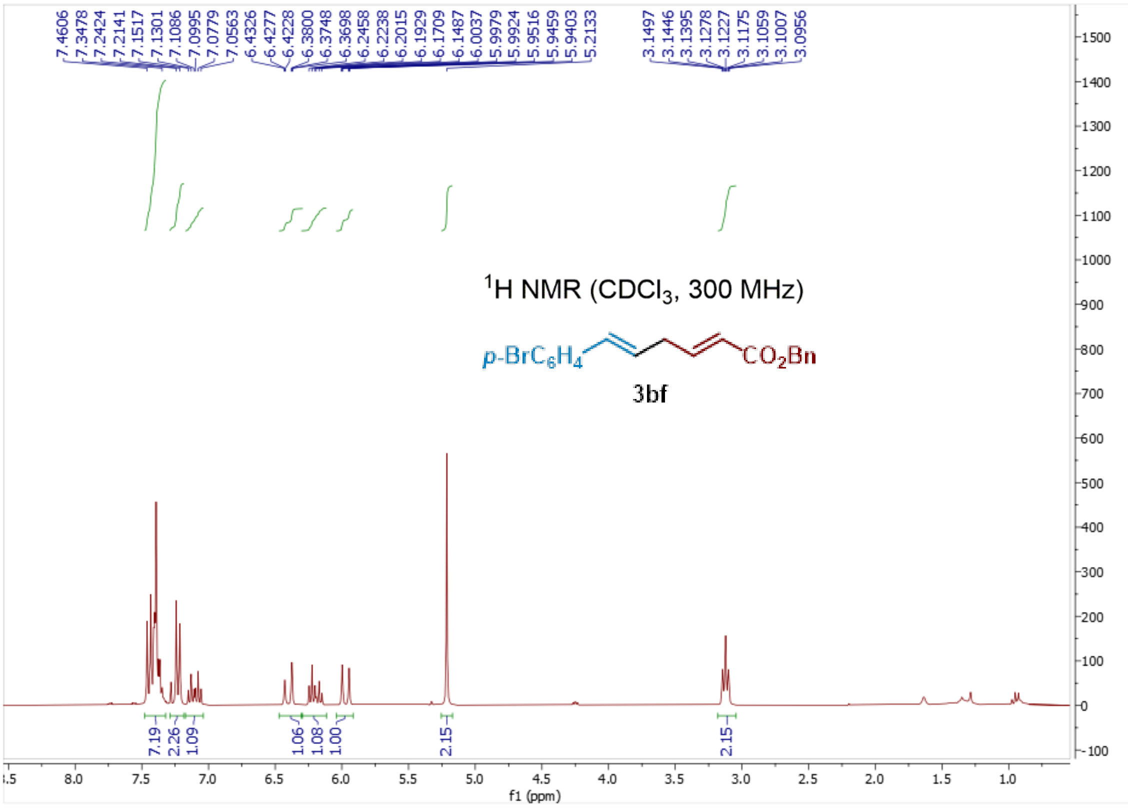
7.4606
7.3478
7.2424
7.2141
7.1517
7.1301
7.1086
7.0995
7.0779
7.0563
6.4326
6.4277
6.4228
6.3800
6.3748
6.3698
6.2458
6.2238
6.2015
6.1929
6.1709
6.1487
6.0037
5.9979
5.9924
5.9516
5.9459
5.9403
5.2133
3.1497
3.1446
3.1395
3.1278
3.1227
3.1175
3.1059
3.1007
3.0956
$^1$H NMR ($CDCl_3$, 300 MHz)
p-$BrC_6H_4$
$CO_2Bn$
3bf
7.19
2.26
1.09
1.06
1.08
1.00
2.15
2.15
f1 (ppm)

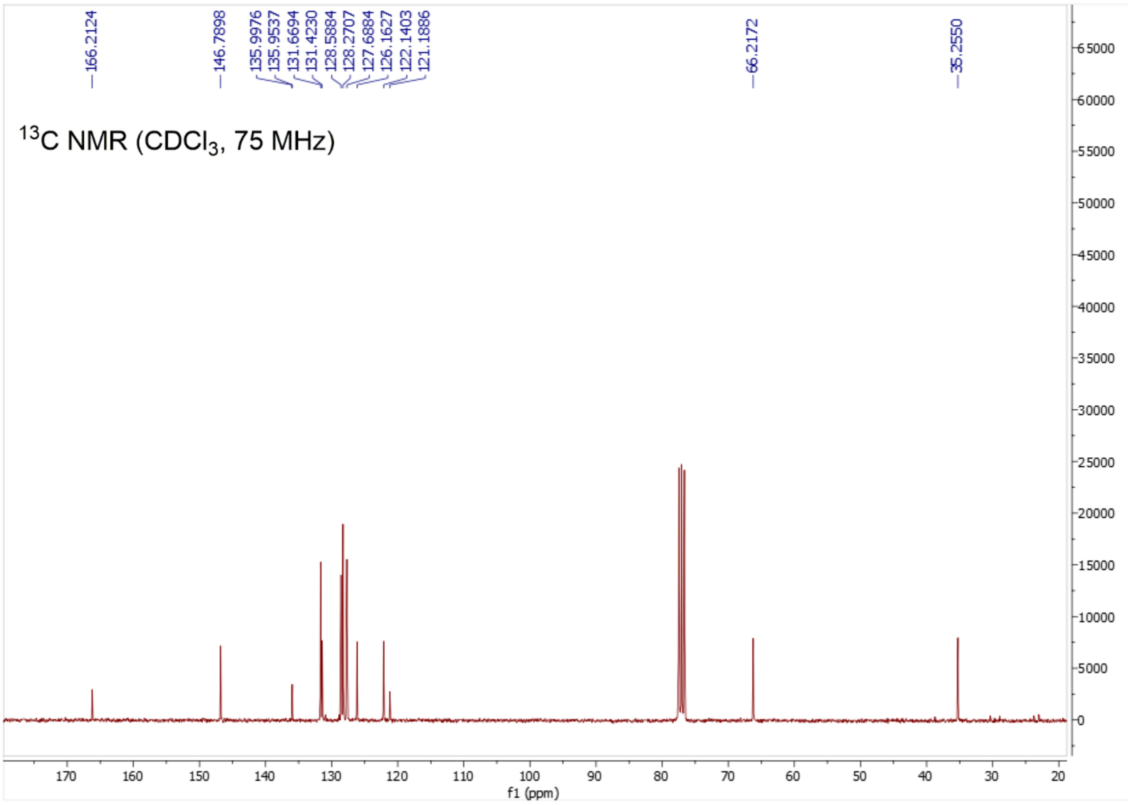
166.2124
146.7898
135.9976
135.9537
131.6694
131.4230
128.5884
128.2707
127.6884
126.1627
122.1403
121.1886
66.2172
35.2550
$^{13}$C NMR ($CDCl_3$, 75 MHz)
f1 (ppm)

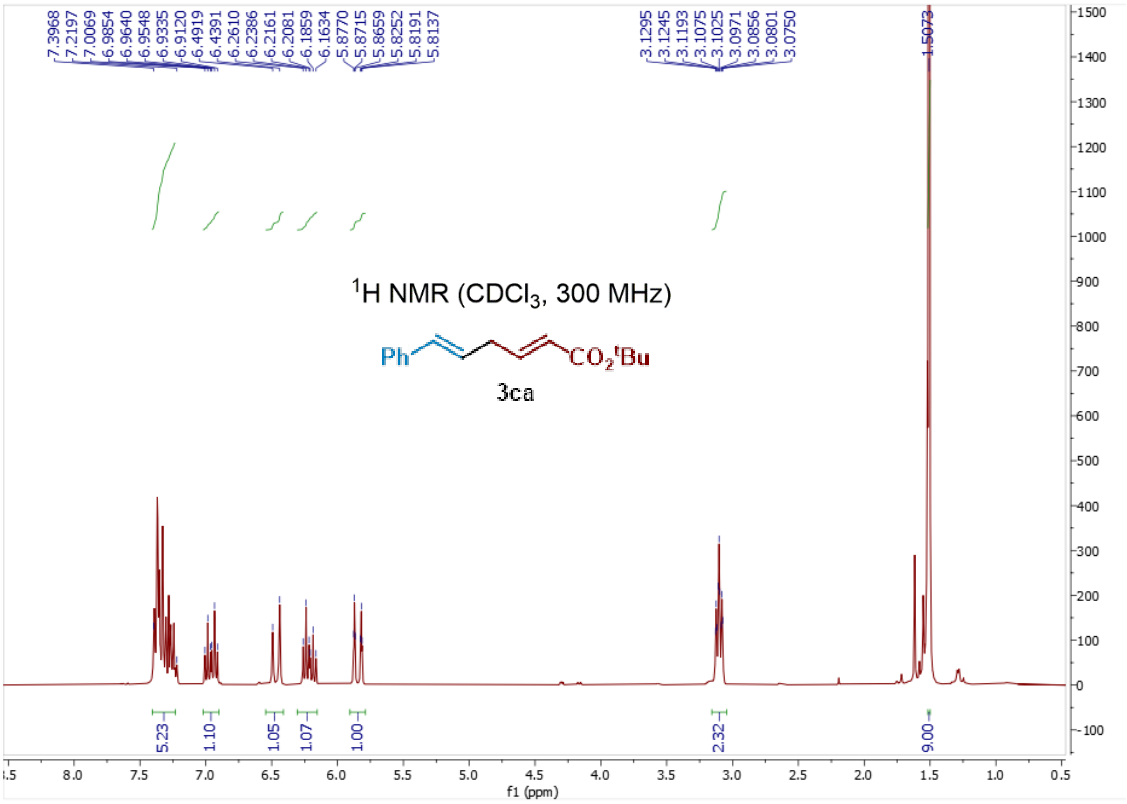
1H NMR (CDCl3, 300 MHz)
Ph
CO2tBu
3ca
7.3968
7.2197
7.0069
6.9854
6.9640
6.9548
6.9335
6.9120
6.4919
6.4391
6.2610
6.2386
6.2161
6.2081
6.1859
6.1634
5.8770
5.8715
5.8659
5.8252
5.8191
5.8137
3.1295
3.1245
3.1193
3.1075
3.1025
3.0971
3.0856
3.0801
3.0750
1.5073
5.23
1.10
1.05
1.07
1.00
2.32
9.00
f1 (ppm)

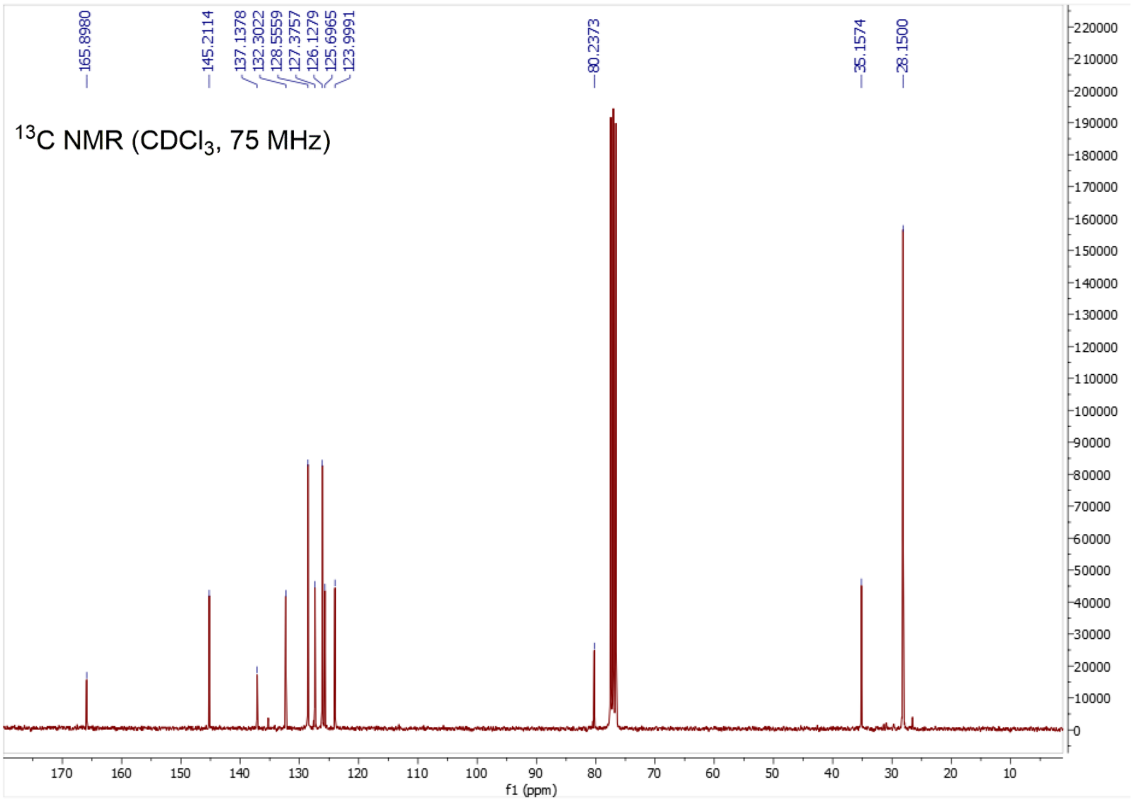
13C NMR (CDCl3, 75 MHz)
165.8980
145.2114
137.1378
132.3022
128.5559
127.3757
126.1279
125.6965
123.9991
80.2373
35.1574
28.1500
f1 (ppm)

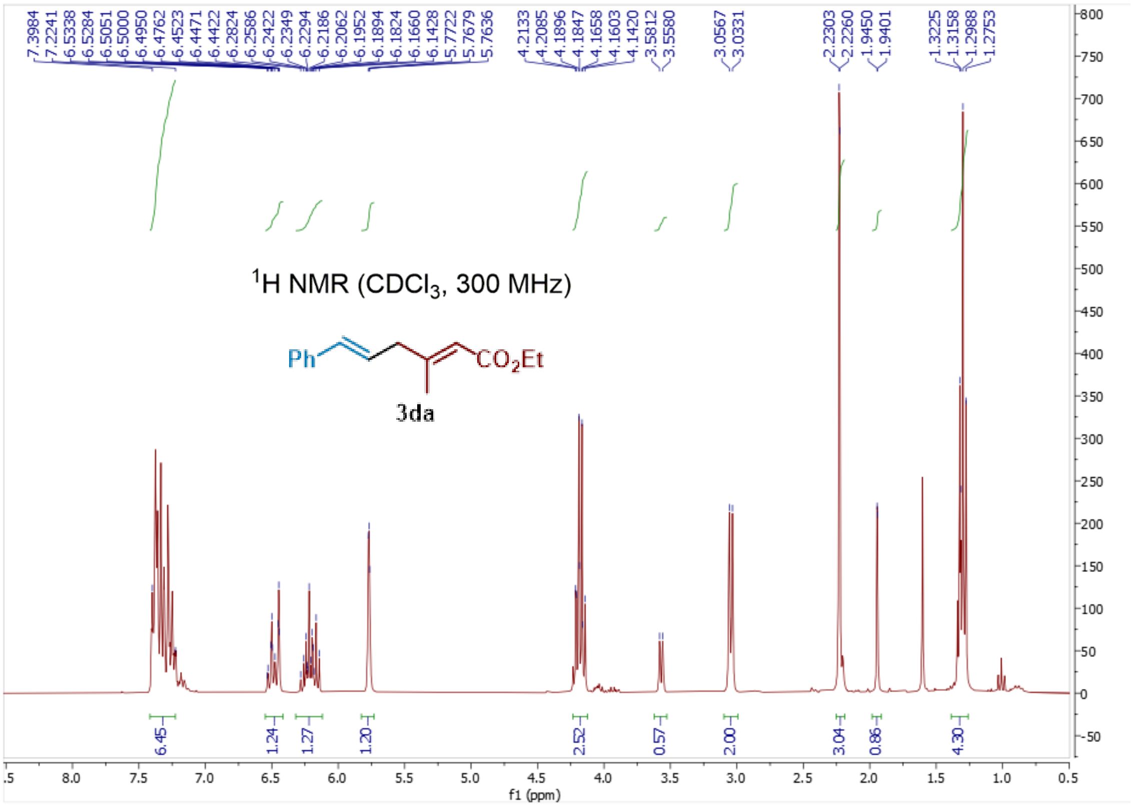

$^1$H NMR (CDCl$_3$, 300 MHz)
Ph
CO$_2$Et
3da
f1 (ppm)


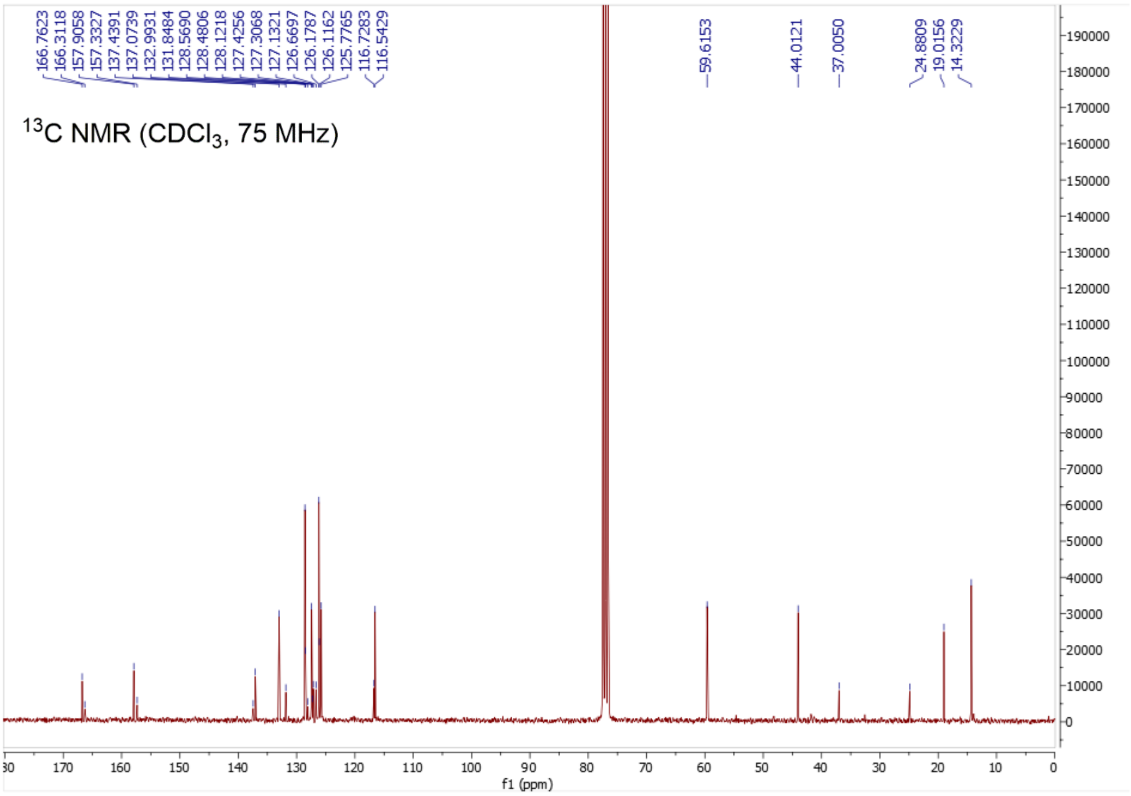

$^{13}$C NMR (CDCl$_3$, 75 MHz)
f1 (ppm)

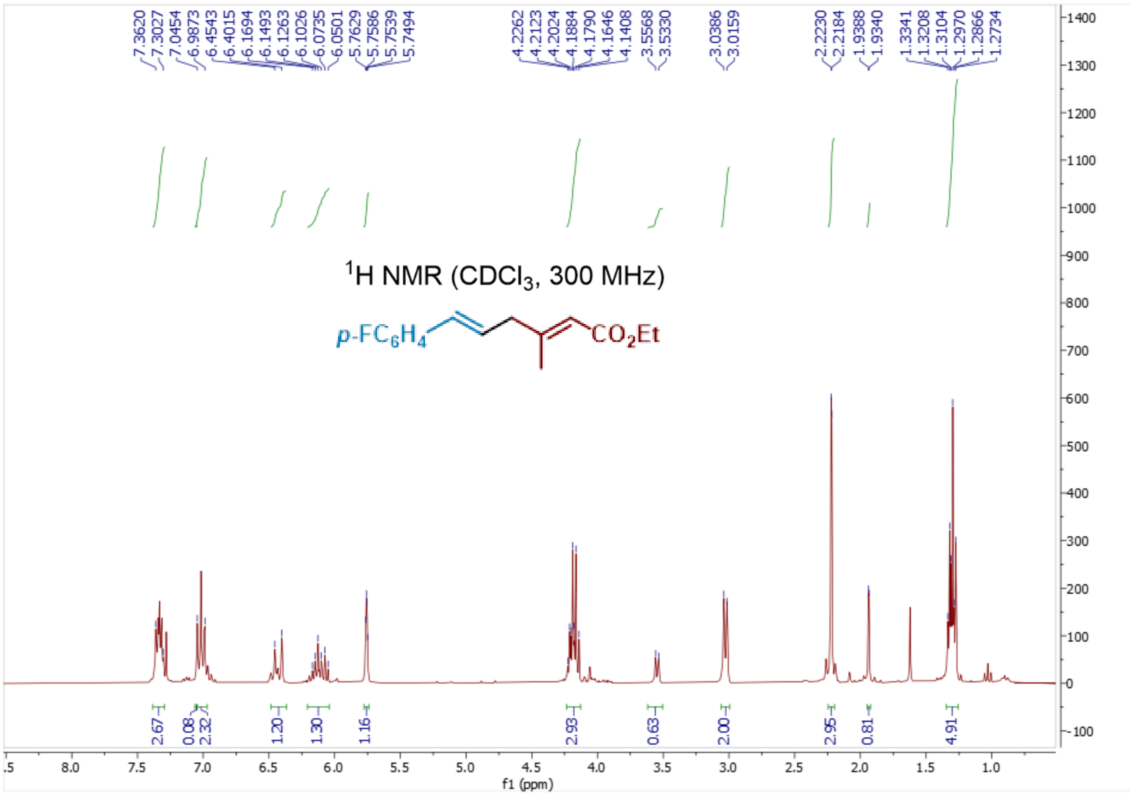

$^1$H NMR (CDCl$_3$, 300 MHz)
p-FC$_6$H$_4$ CO$_2$Et
f1 (ppm)


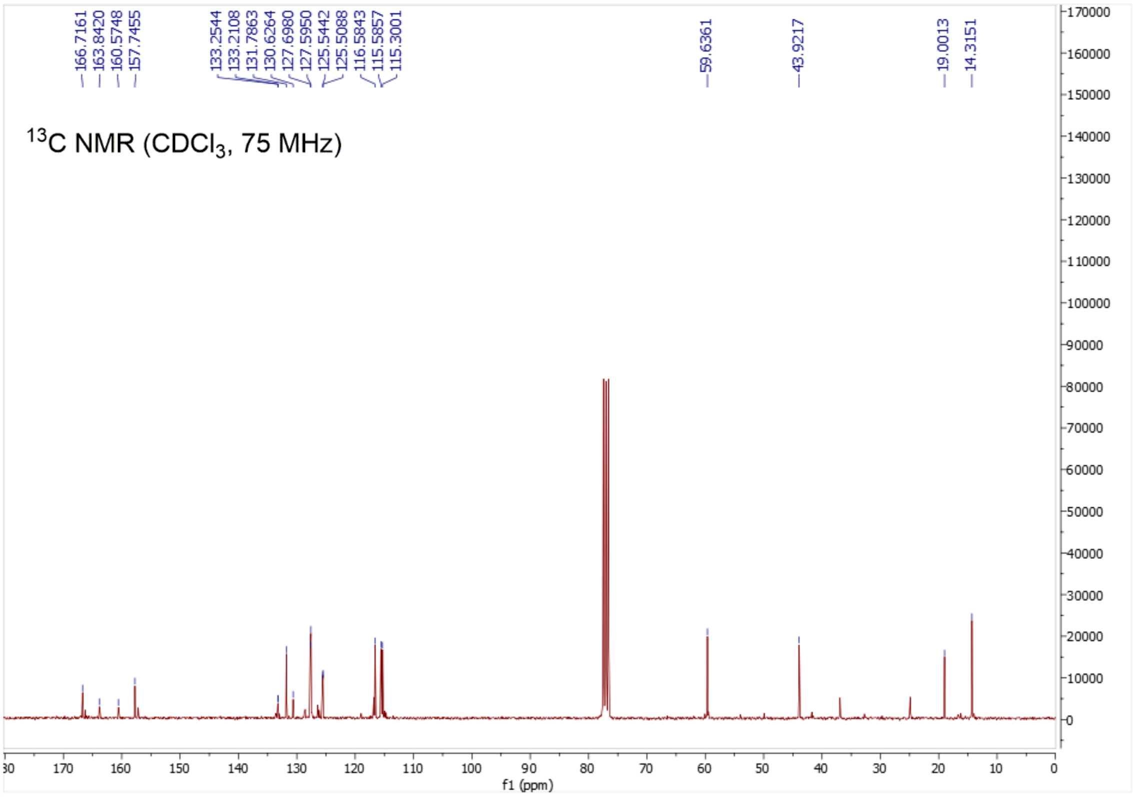

$^{13}$C NMR (CDCl$_3$, 75 MHz)
f1 (ppm)

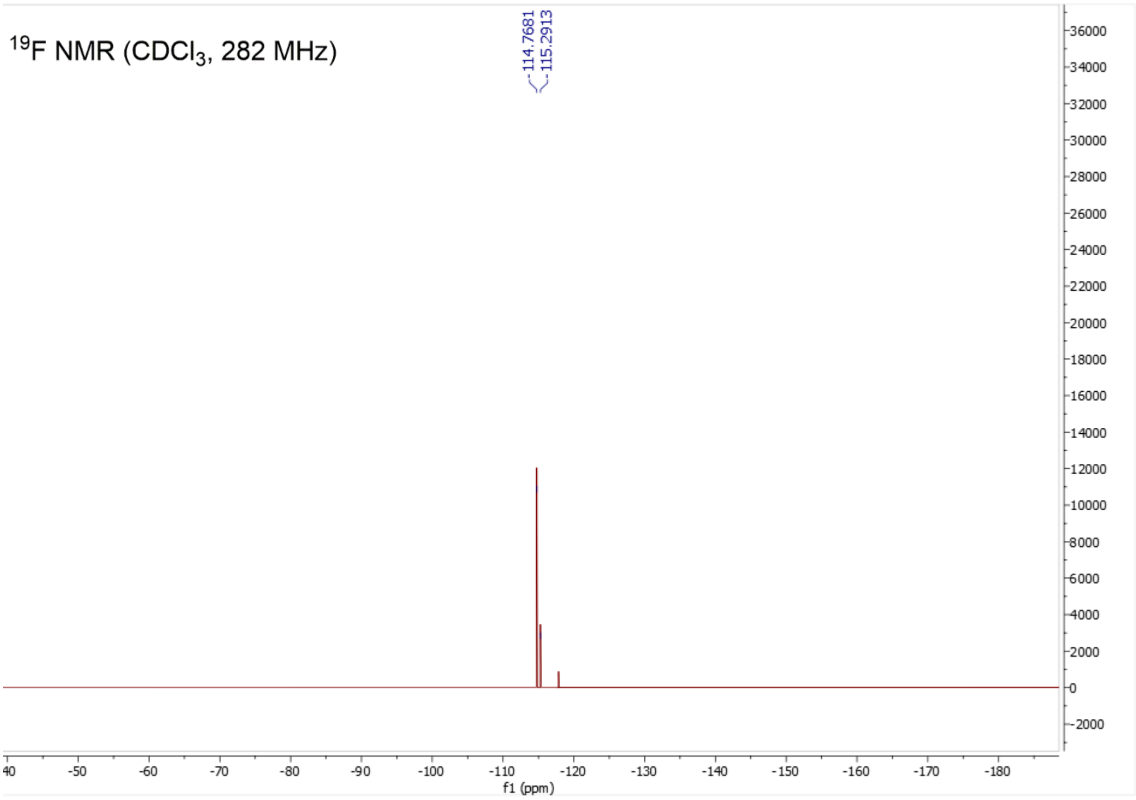
19F NMR (CDCl3, 282 MHz)
-114.7681
-115.2913
f1 (ppm)

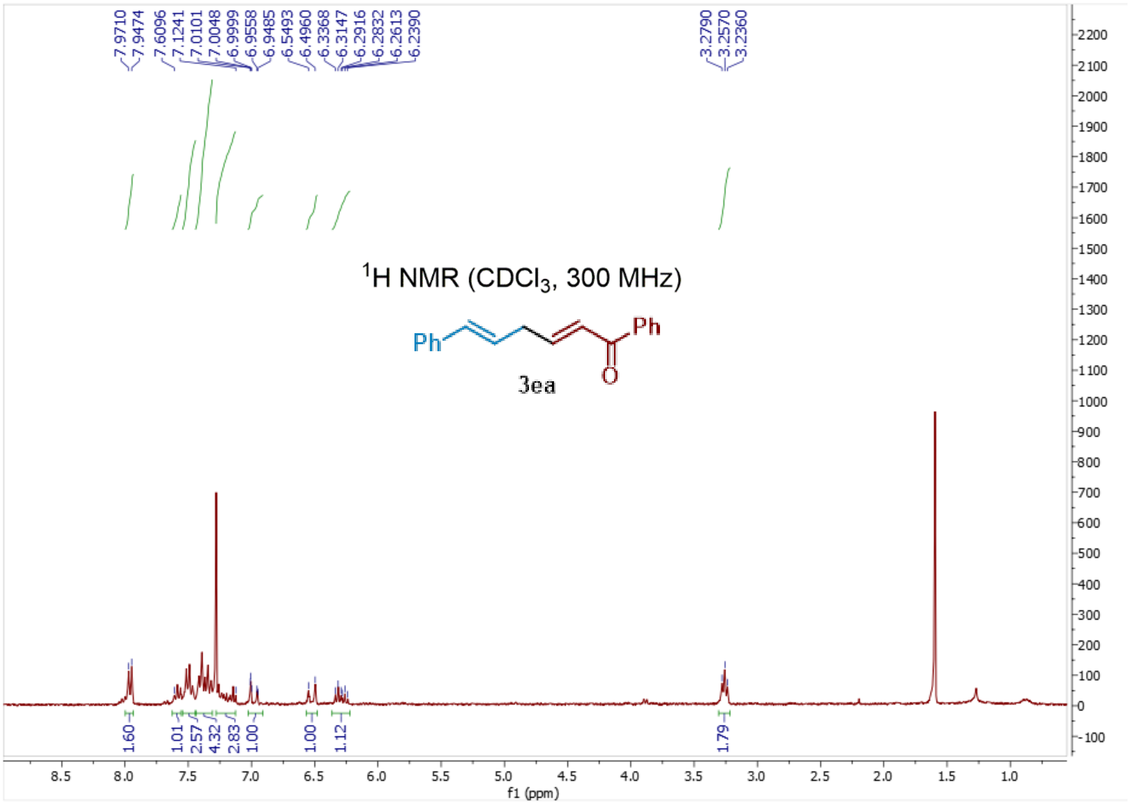
7.9710
7.9474
7.6096
7.1241
7.0101
7.0048
6.9999
6.9558
6.9485
6.5493
6.4960
6.3368
6.3147
6.2916
6.2832
6.2613
6.2390
3.2790
3.2570
3.2360
$^{1}$H NMR ($CDCl_3$, 300 MHz)
Ph
Ph
O
3ea
1.60
1.01
2.57
4.32
2.83
1.00
1.00
1.12
1.79
f1 (ppm)

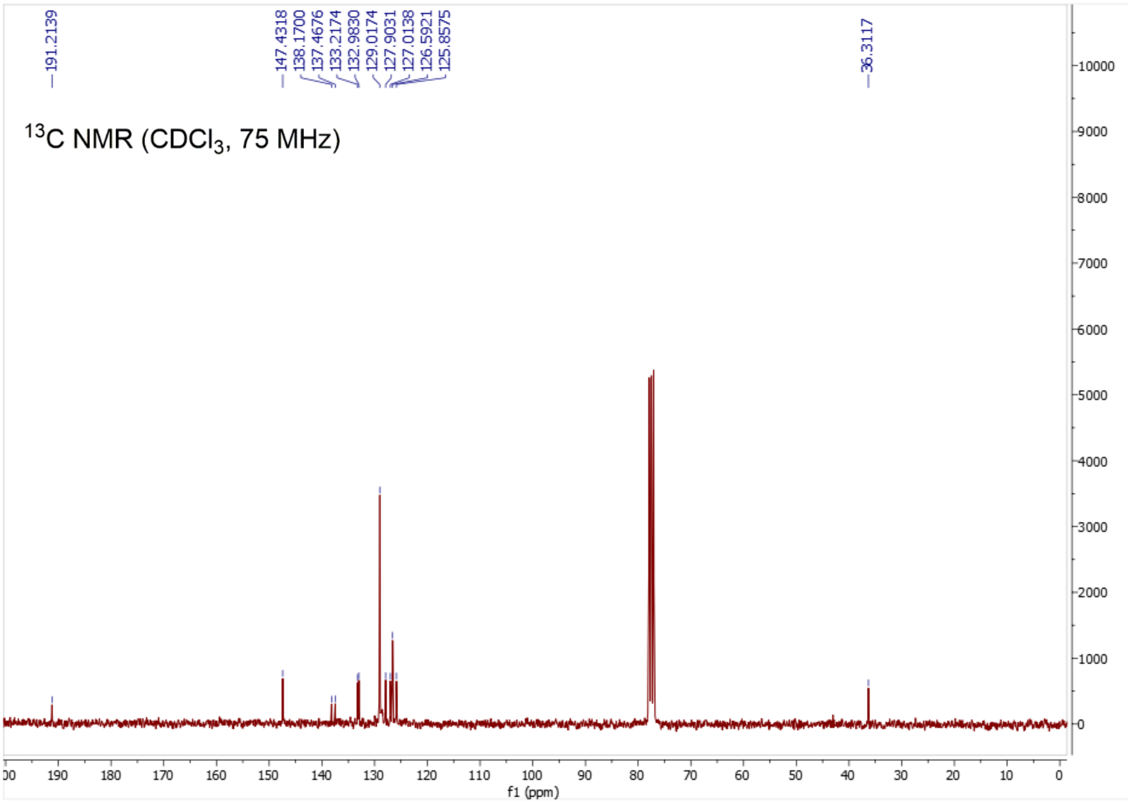
191.2139
147.4318
138.1700
137.4676
133.2174
132.9830
129.0174
127.9031
127.0138
126.5921
125.8575
36.3117
$^{13}$C NMR ($CDCl_3$, 75 MHz)
f1 (ppm)

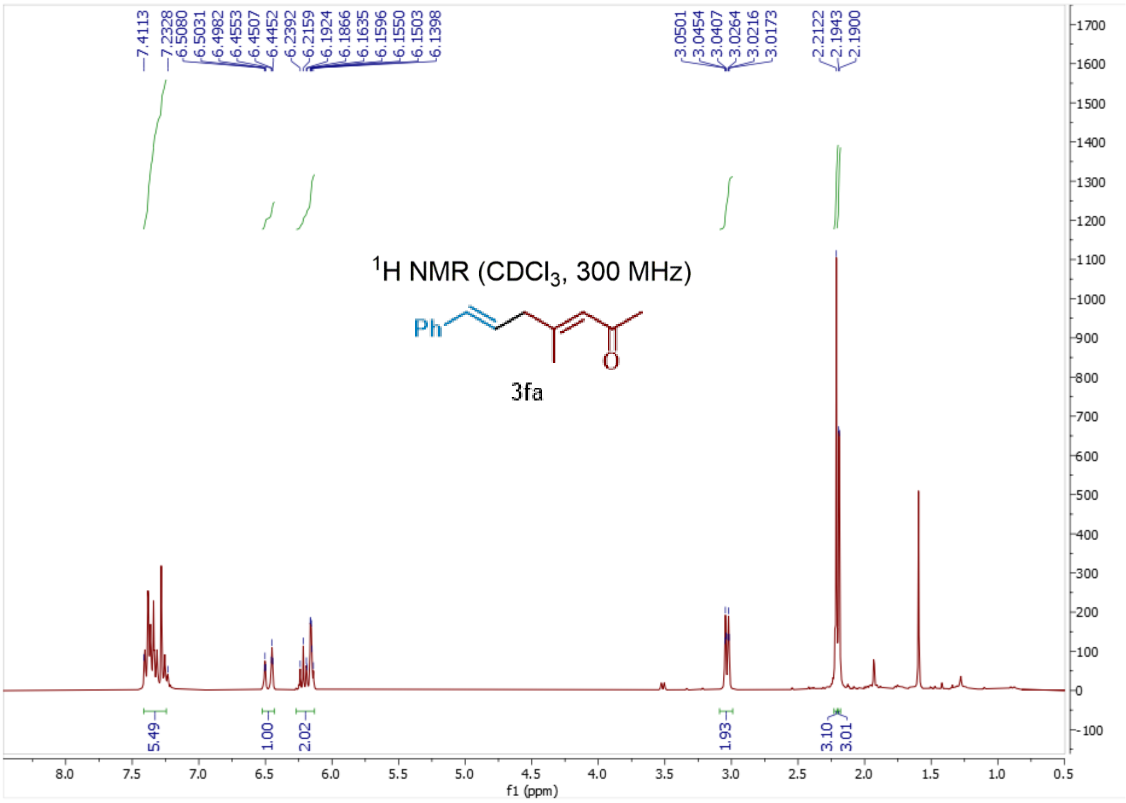

1H NMR (CDCl3, 300 MHz)
Ph
O
3fa
7.4113
7.2328
6.5080
6.5031
6.4982
6.4553
6.4507
6.4452
6.2392
6.2159
6.1924
6.1866
6.1635
6.1596
6.1550
6.1503
6.1398
3.0501
3.0454
3.0407
3.0264
3.0216
3.0173
2.2122
2.1943
2.1900
5.49
1.00
2.02
1.93
3.10
3.01
f1 (ppm)


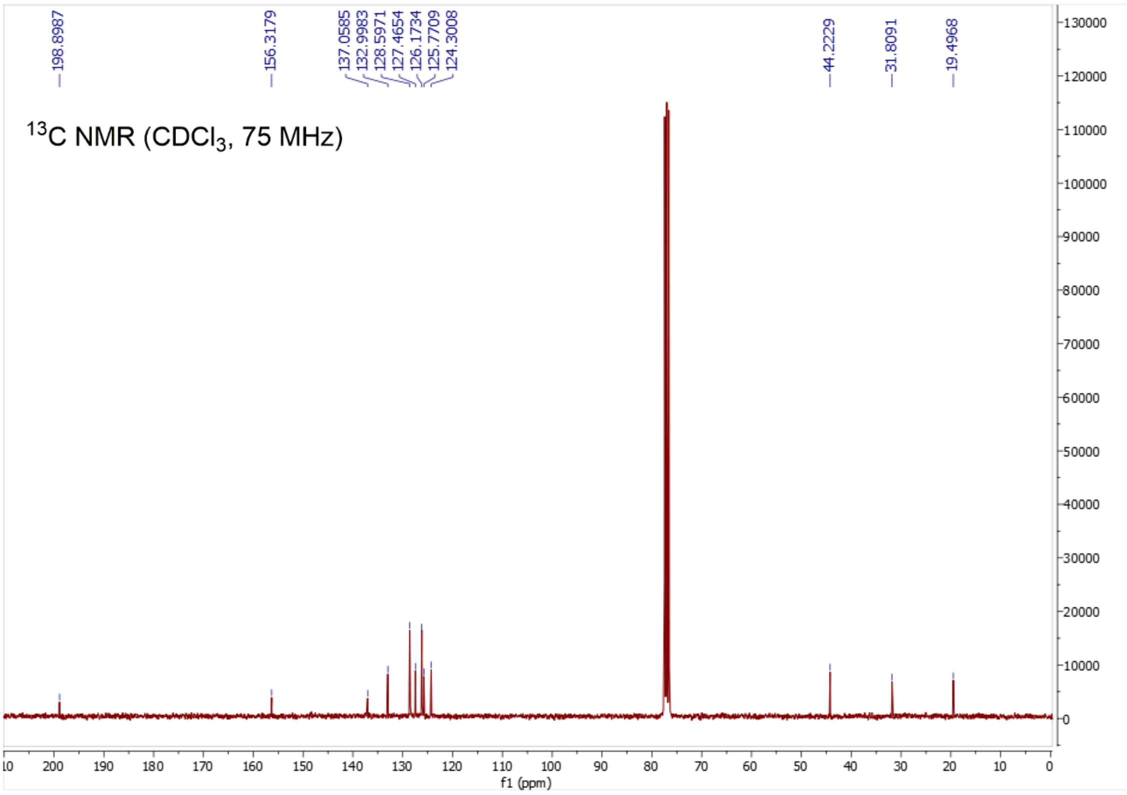

13C NMR (CDCl3, 75 MHz)
198.8987
156.3179
137.0585
132.9983
128.5971
127.4654
126.1734
125.7709
124.3008
44.2229
31.8091
19.4968
f1 (ppm)

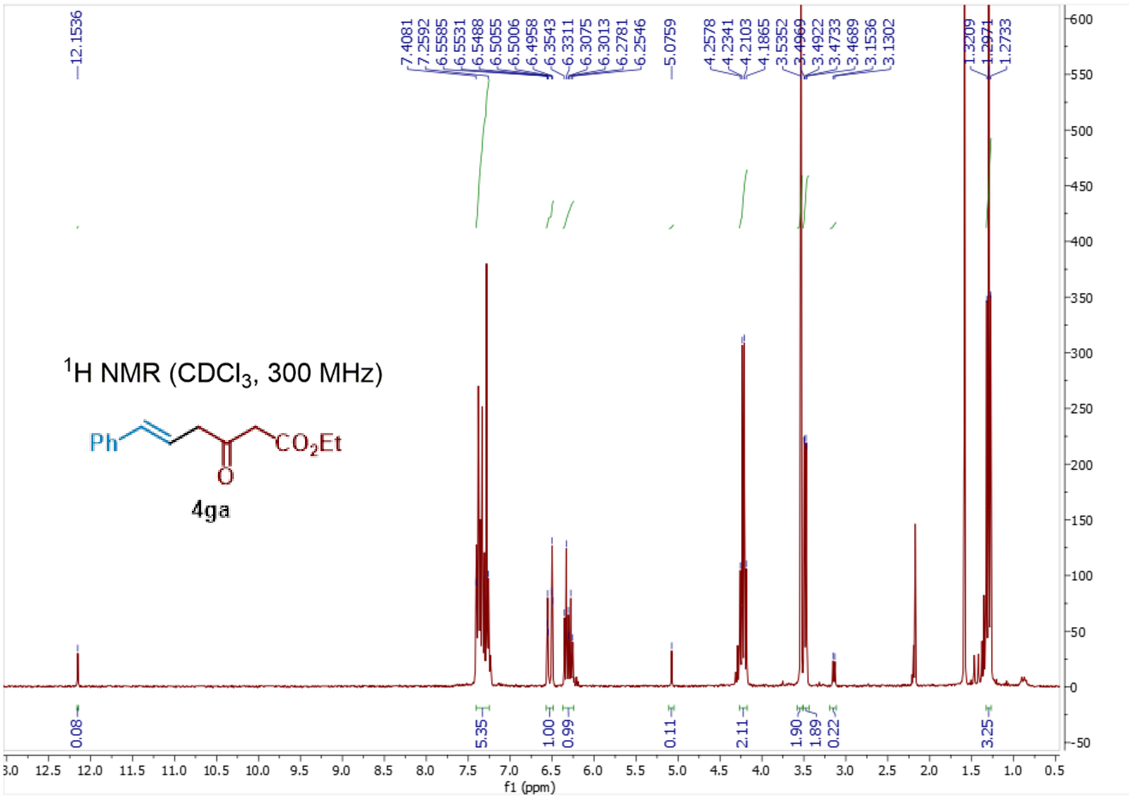
1H NMR (CDCl3, 300 MHz)
Ph
CO2Et
O
4ga
f1 (ppm)

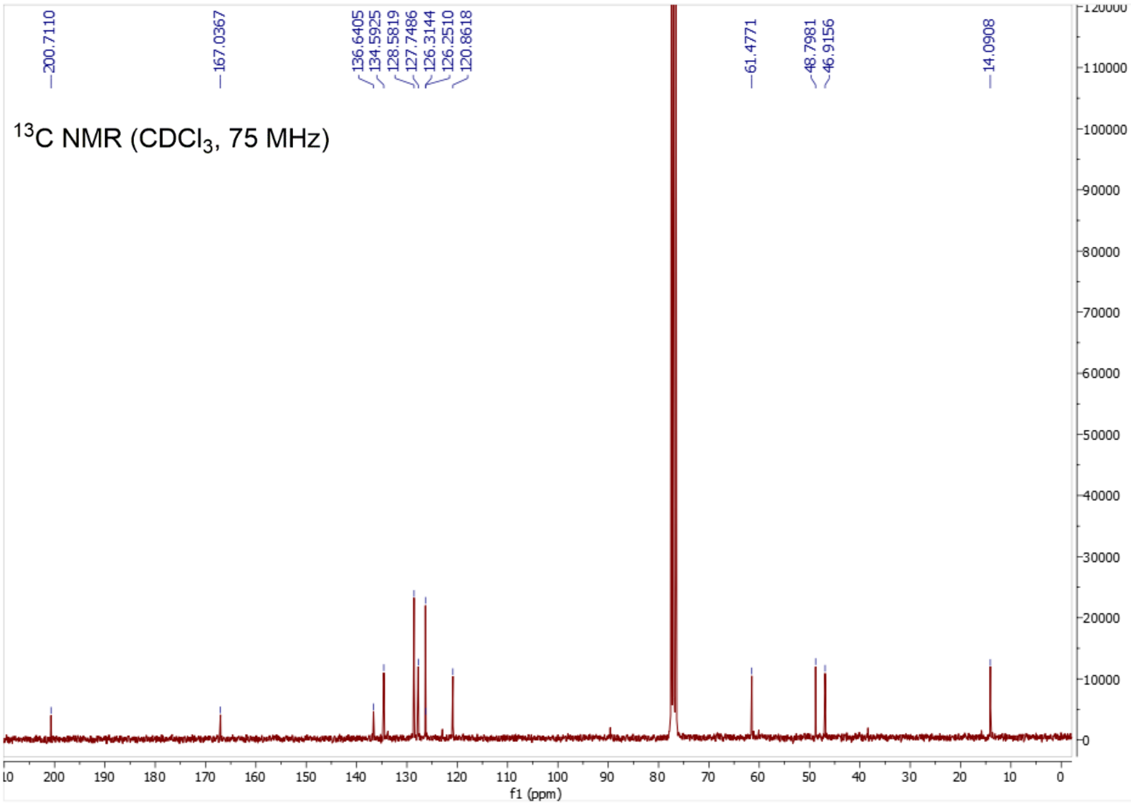
13C NMR (CDCl3, 75 MHz)
f1 (ppm)

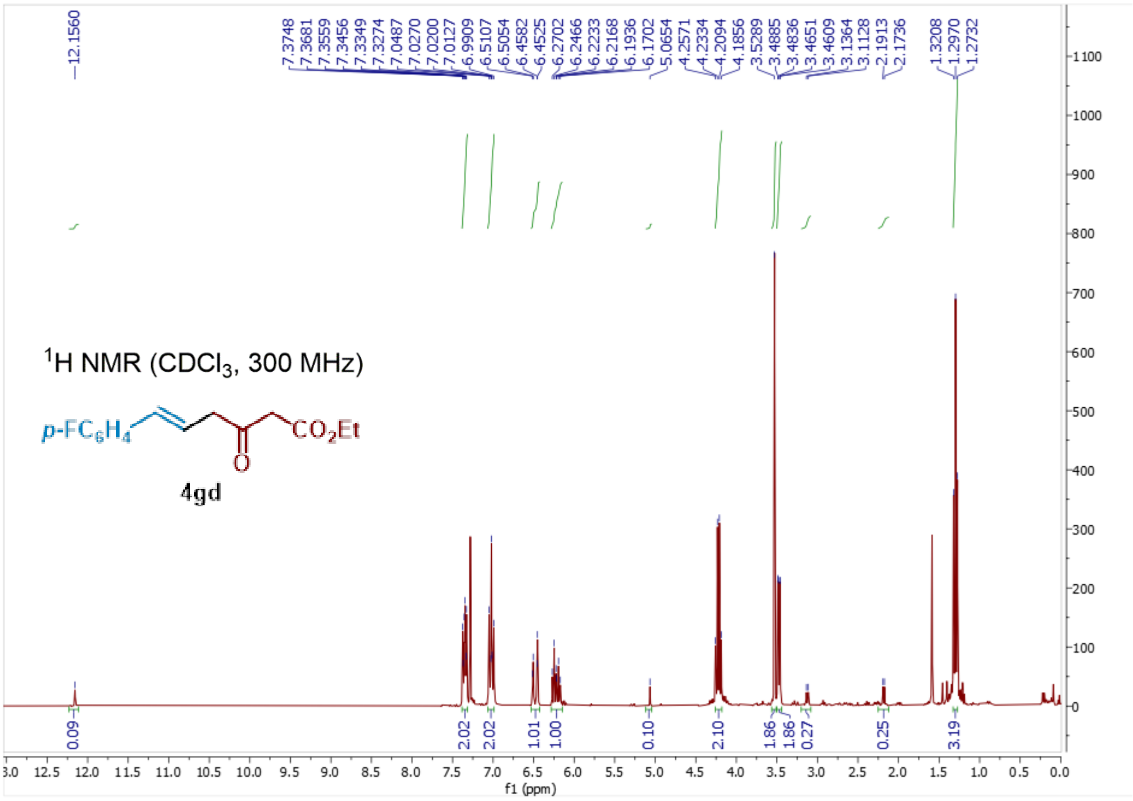

$^{1}H$ NMR ($CDCl_3$, 300 MHz)
p-FC6H4
CO2Et
O
4gd
f1 (ppm)


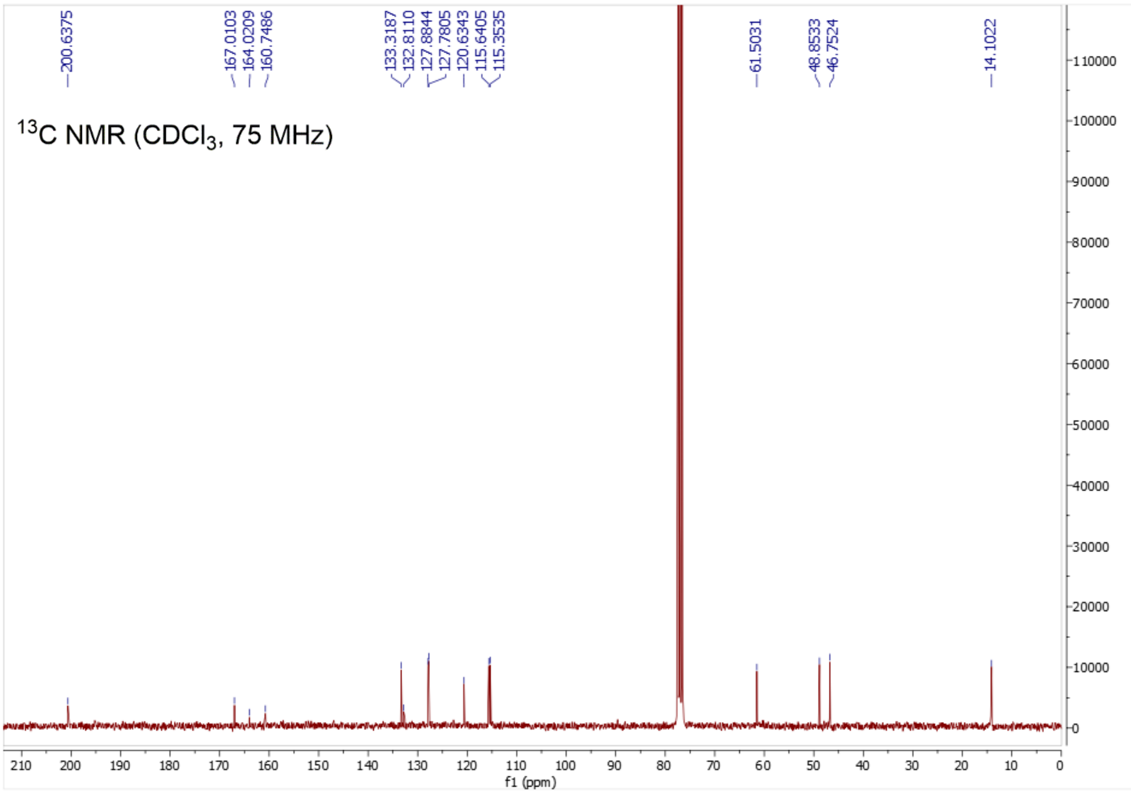

$^{13}C$ NMR ($CDCl_3$, 75 MHz)
200.6375
167.0103
164.0209
160.7486
133.3187
132.8110
127.8844
127.7805
120.6343
115.6405
115.3535
61.5031
48.8533
46.7524
14.1022
f1 (ppm)

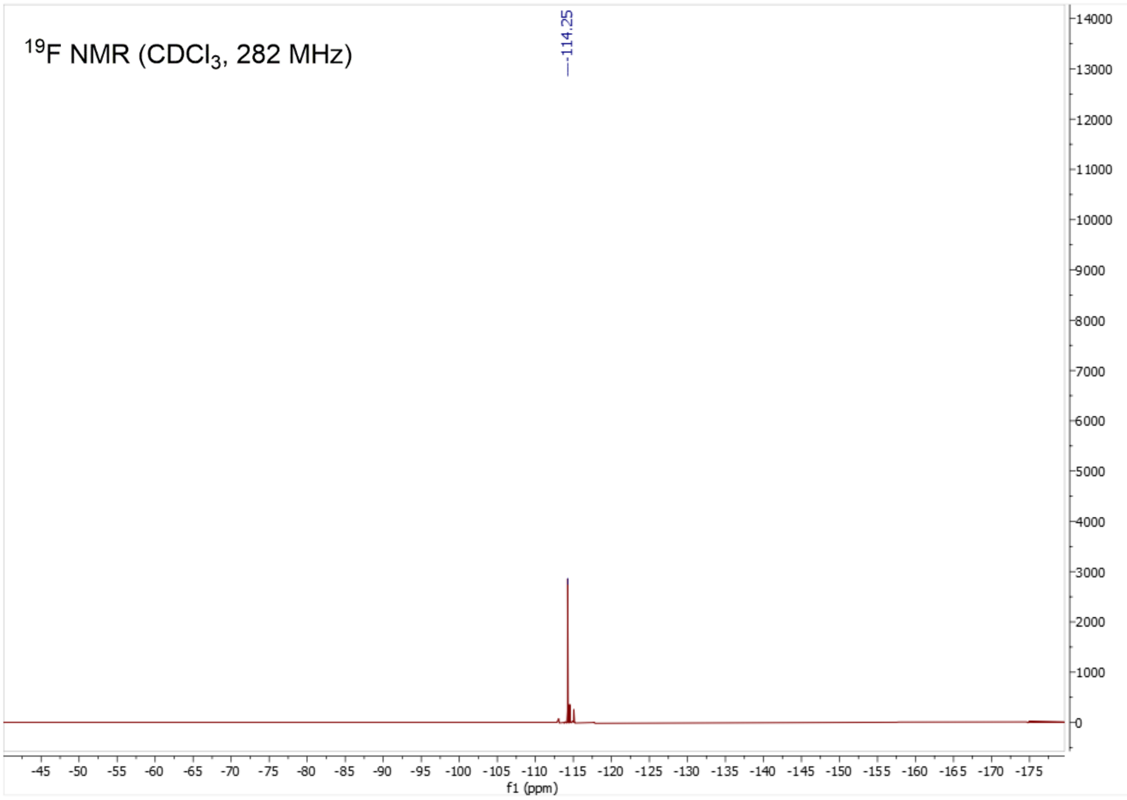
$^{19}$F NMR ($CDCl_3$, 282 MHz)
-114.25
f1 (ppm)

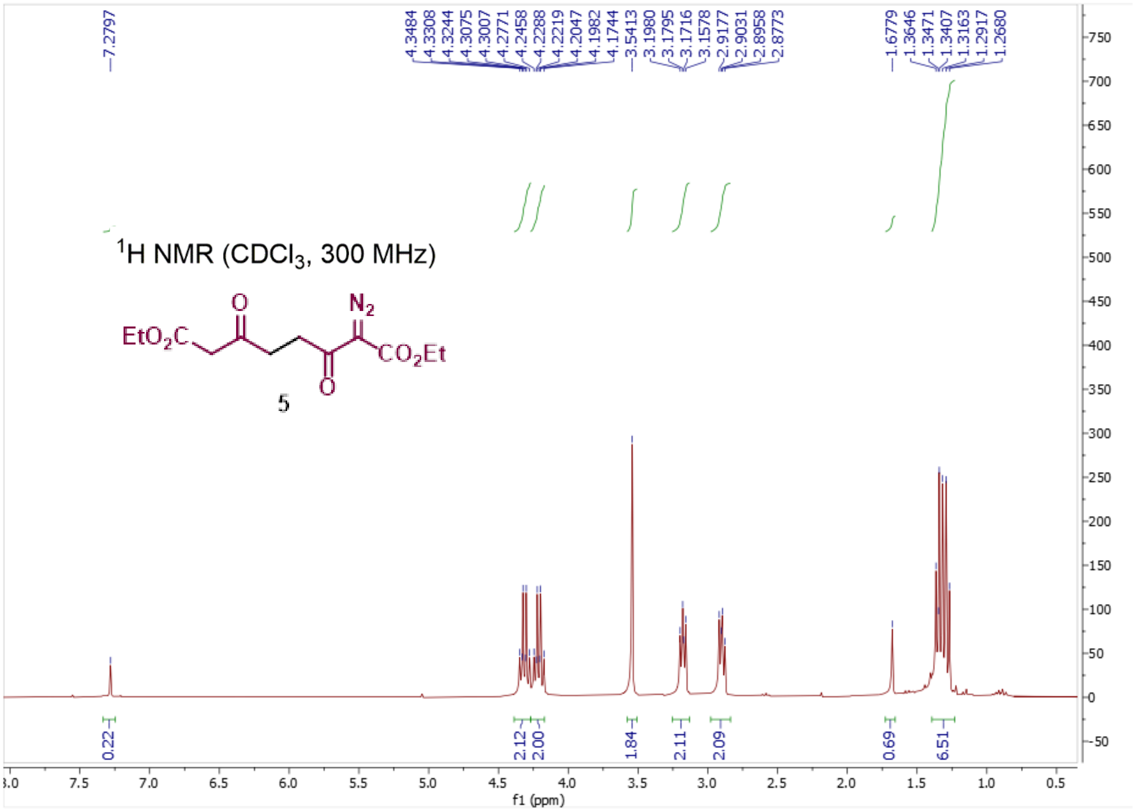

7.2797
4.3484
4.3308
4.3244
4.3075
4.3007
4.2771
4.2458
4.2288
4.2219
4.2047
4.1982
4.1744
3.5413
3.1980
3.1795
3.1716
3.1578
2.9177
2.9031
2.8958
2.8773
1.6779
1.3646
1.3471
1.3407
1.3163
1.2917
1.2680
1H NMR (CDCl3, 300 MHz)
EtO2C
O
N2
CO2Et
O
5
0.22
2.12
2.00
1.84
2.11
2.09
0.69
6.51
f1 (ppm)


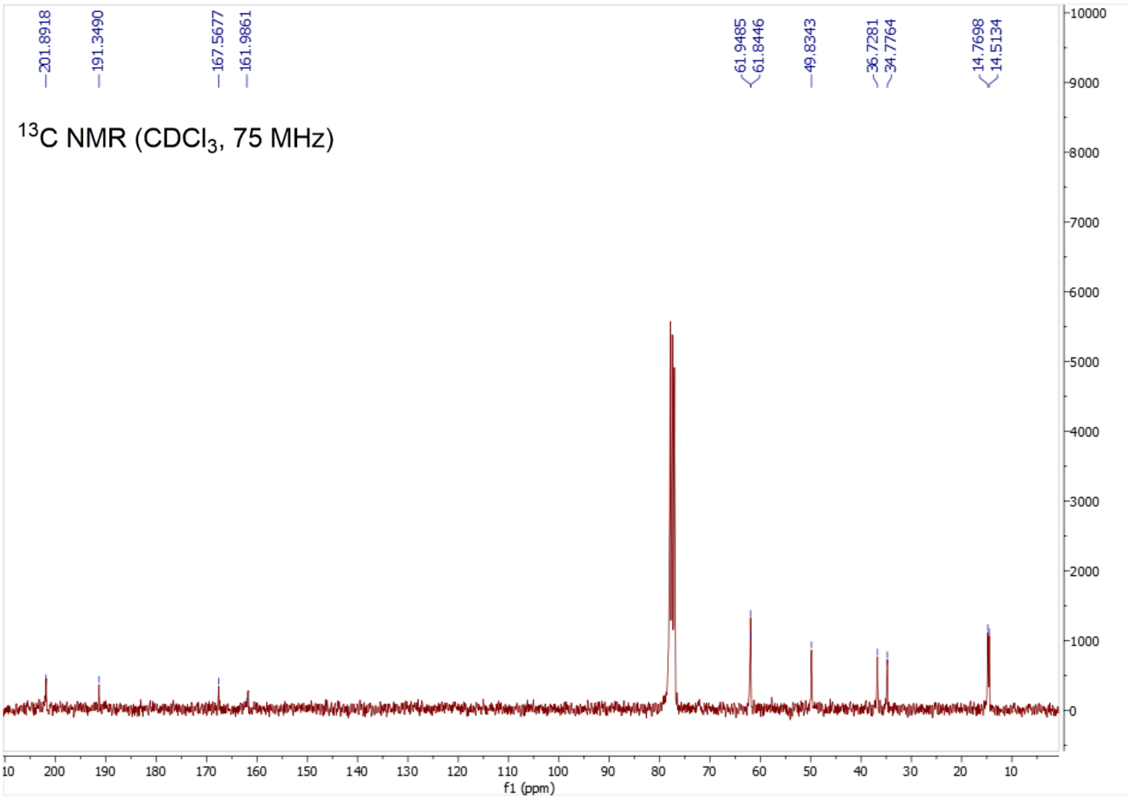

201.8918
191.3490
167.5677
161.9861
61.9485
61.8446
49.8343
36.7281
34.7764
14.7698
14.5134
13C NMR (CDCl3, 75 MHz)
f1 (ppm)

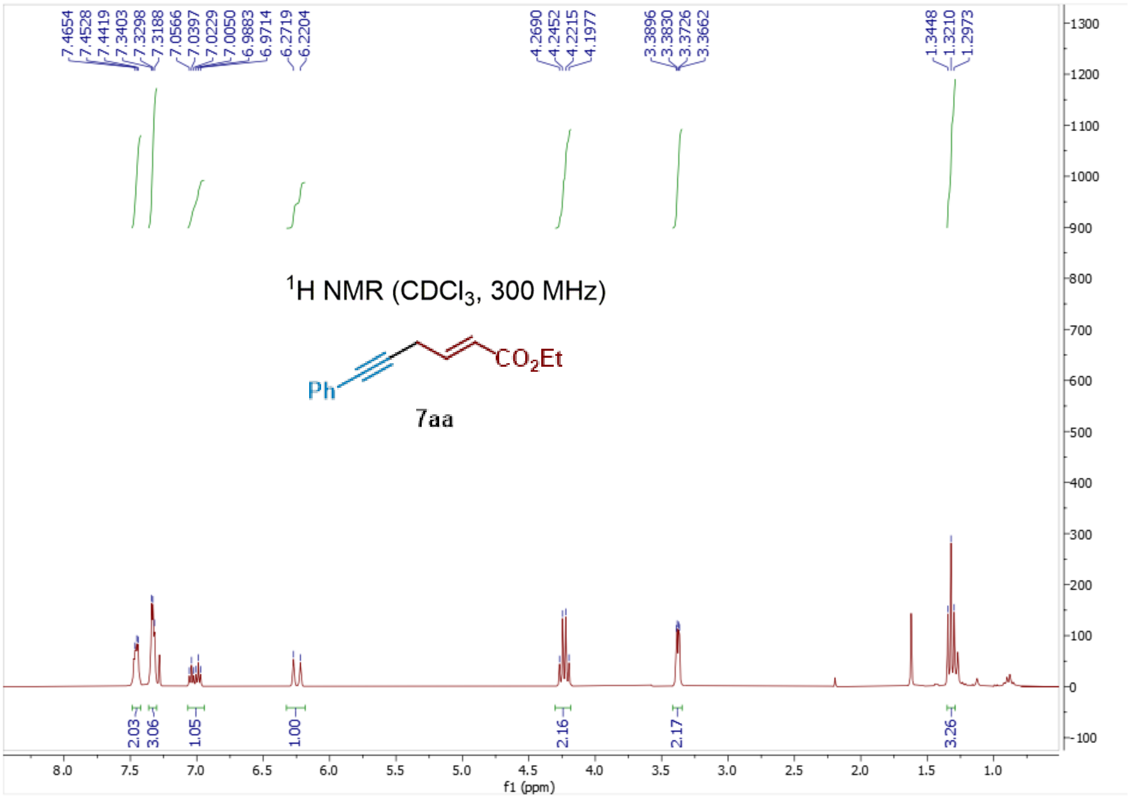
7.4654
7.4528
7.4419
7.3403
7.3298
7.3188
7.0566
7.0397
7.0229
7.0050
6.9883
6.9714
6.2719
6.2204
4.2690
4.2452
4.2215
4.1977
3.3896
3.3830
3.3726
3.3662
1.3448
1.3210
1.2973
1H NMR (CDCl3, 300 MHz)
Ph
CO2Et
7aa
2.03
3.06
1.05
1.00
2.16
2.17
3.26
f1 (ppm)

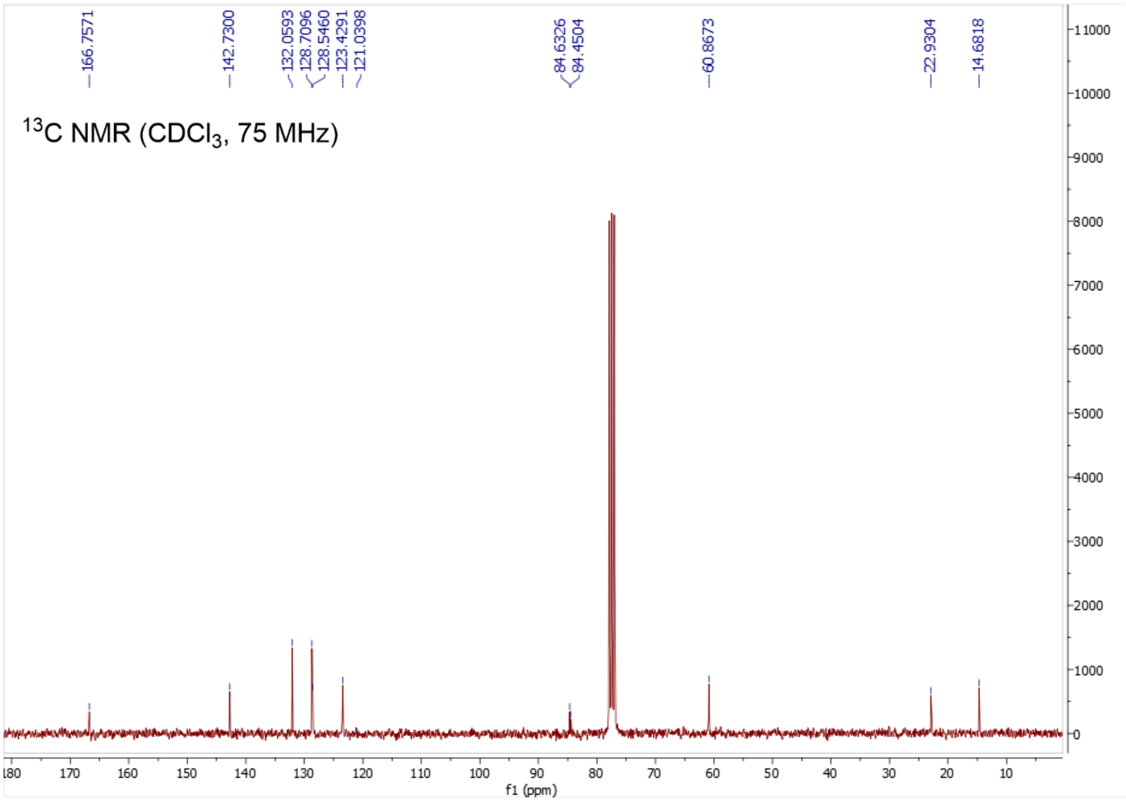
166.7571
142.7300
132.0593
128.7096
128.5460
123.4291
121.0398
84.6326
84.4504
60.8673
22.9304
14.6818
13C NMR (CDCl3, 75 MHz)
f1 (ppm)

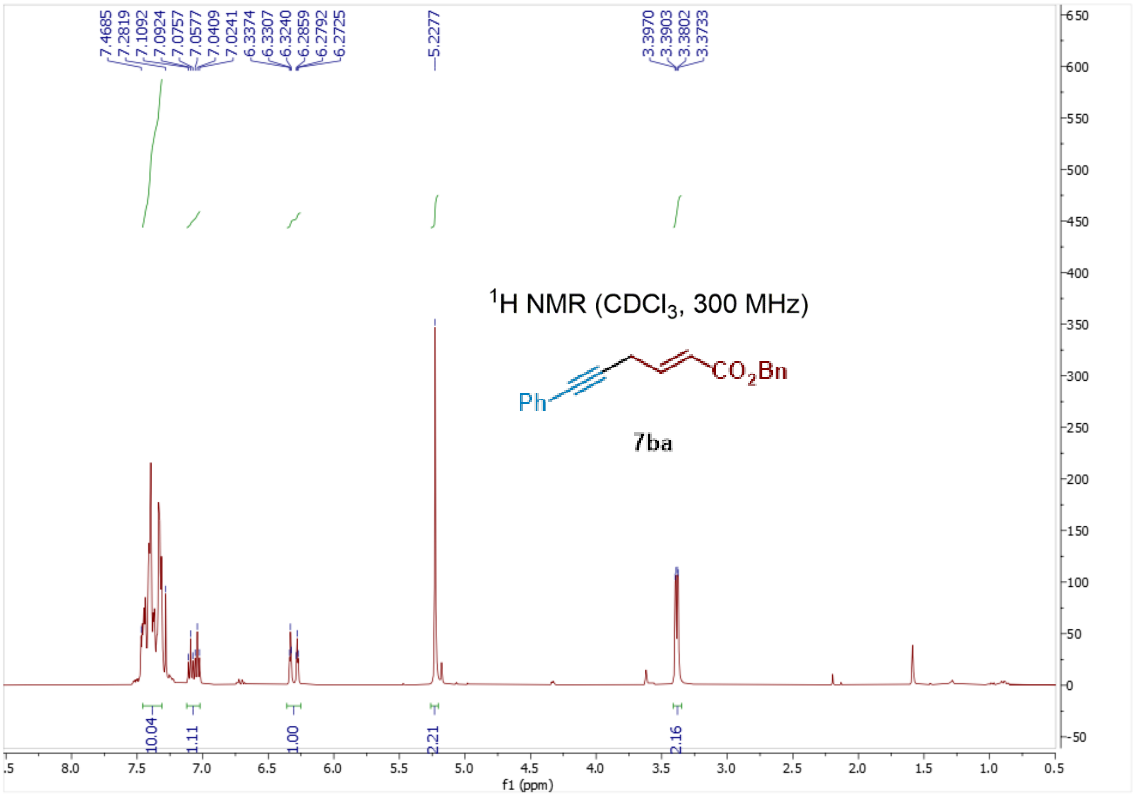
7.4685
7.2819
7.1092
7.0924
7.0757
7.0577
7.0409
7.0241
6.3374
6.3307
6.3240
6.2859
6.2792
6.2725
5.2277
3.3970
3.3903
3.3802
3.3733
1H NMR (CDCl3, 300 MHz)
Ph
CO2Bn
7ba
10.04
1.11
1.00
2.21
2.16
f1 (ppm)

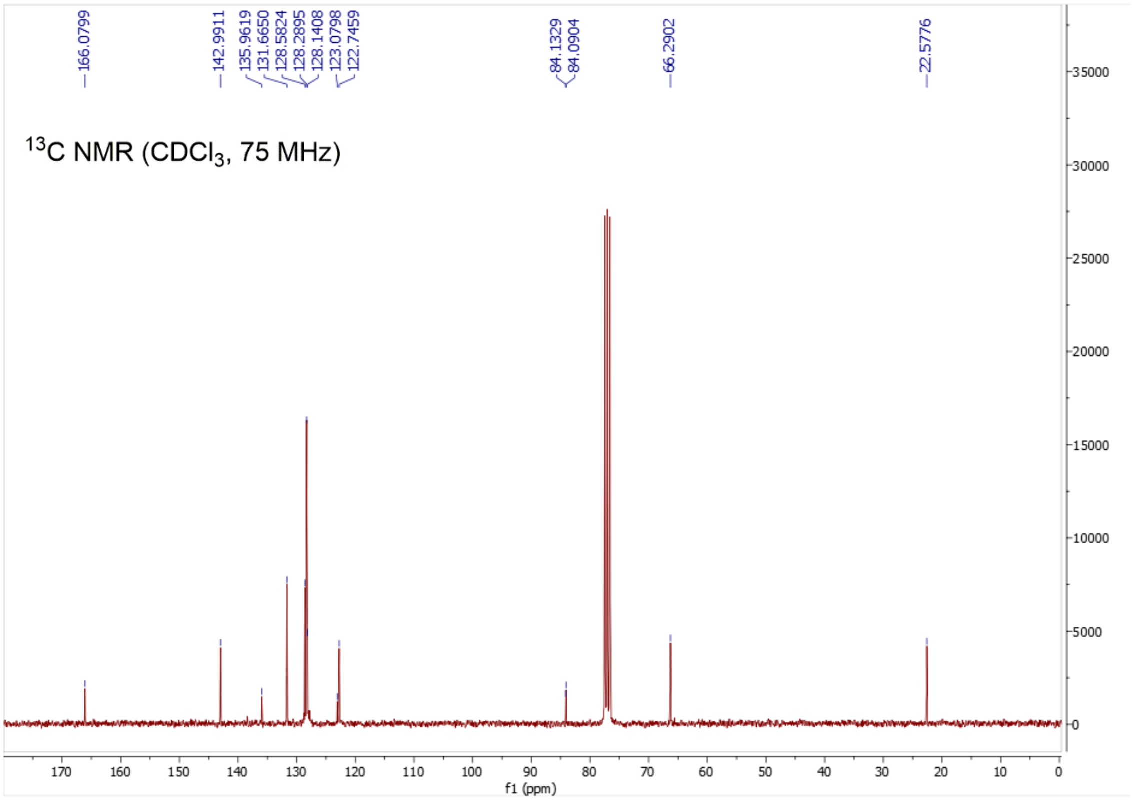
166.0799
142.9911
135.9619
131.6650
128.5824
128.2895
128.1408
123.0798
122.7459
84.1329
84.0904
66.2902
22.5776
13C NMR (CDCl3, 75 MHz)
f1 (ppm)

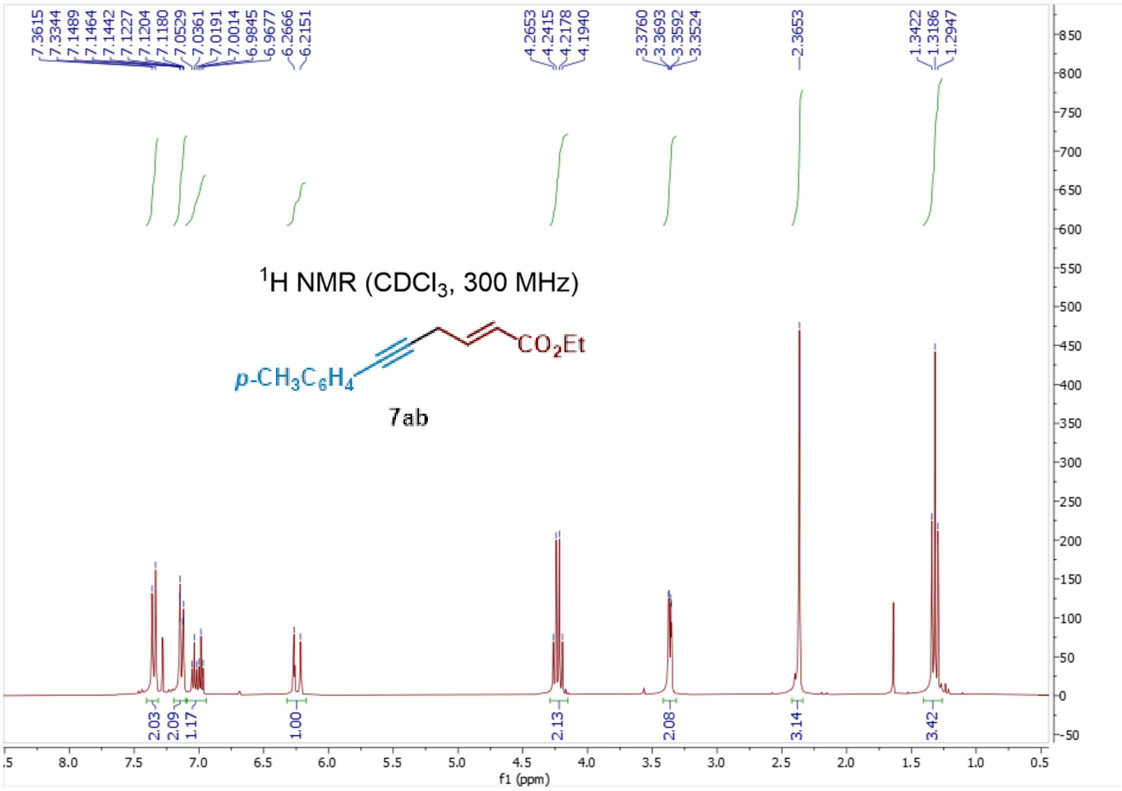

7.3615
7.3344
7.1489
7.1464
7.1442
7.1227
7.1204
7.1180
7.0529
7.0361
7.0191
7.0014
6.9845
6.9677
6.2666
6.2151
4.2653
4.2415
4.2178
4.1940
3.3760
3.3693
3.3592
3.3524
2.3653
1.3422
1.3186
1.2947
1H NMR (CDCl3, 300 MHz)
p-CH3C6H4
CO2Et
7ab
2.03
2.09
1.17
1.00
2.13
2.08
3.14
3.42
f1 (ppm)


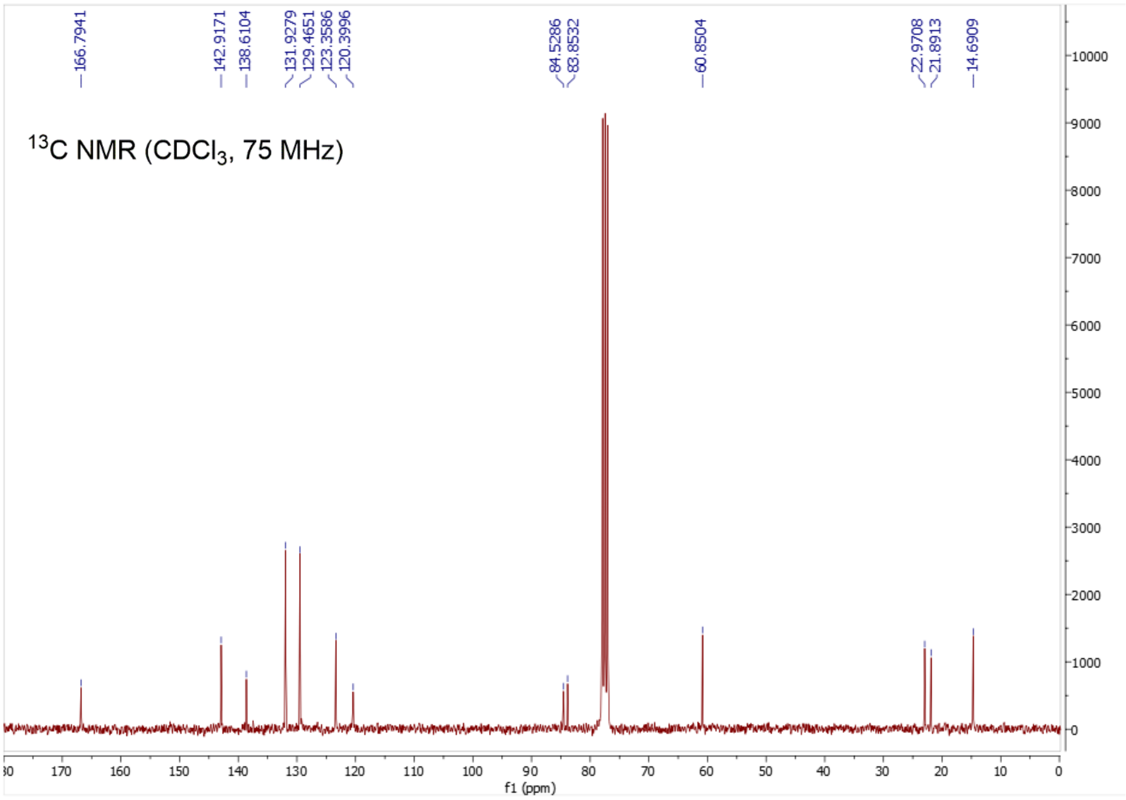

166.7941
142.9171
138.6104
131.9279
129.4651
123.3586
120.3996
84.5286
83.8532
60.8504
22.9708
21.8913
14.6909
13C NMR (CDCl3, 75 MHz)
f1 (ppm)

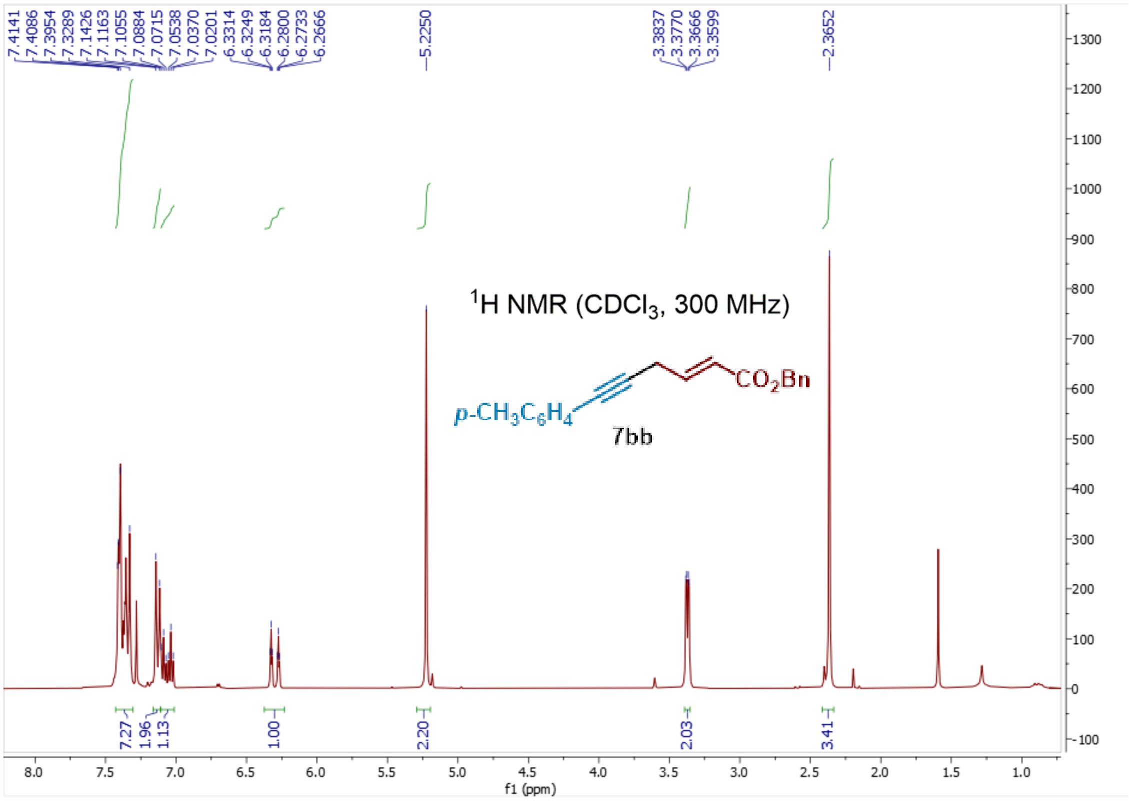
7.4141
7.4086
7.3954
7.3289
7.1426
7.1163
7.1055
7.0884
7.0715
7.0538
7.0370
7.0201
6.3314
6.3249
6.3184
6.2800
6.2733
6.2666
5.2250
3.3837
3.3770
3.3666
3.3599
2.3652
1H NMR (CDCl3, 300 MHz)
p-CH3C6H4
CO2Bn
7bb
7.27
1.96
1.13
1.00
2.20
2.03
3.41
f1 (ppm)

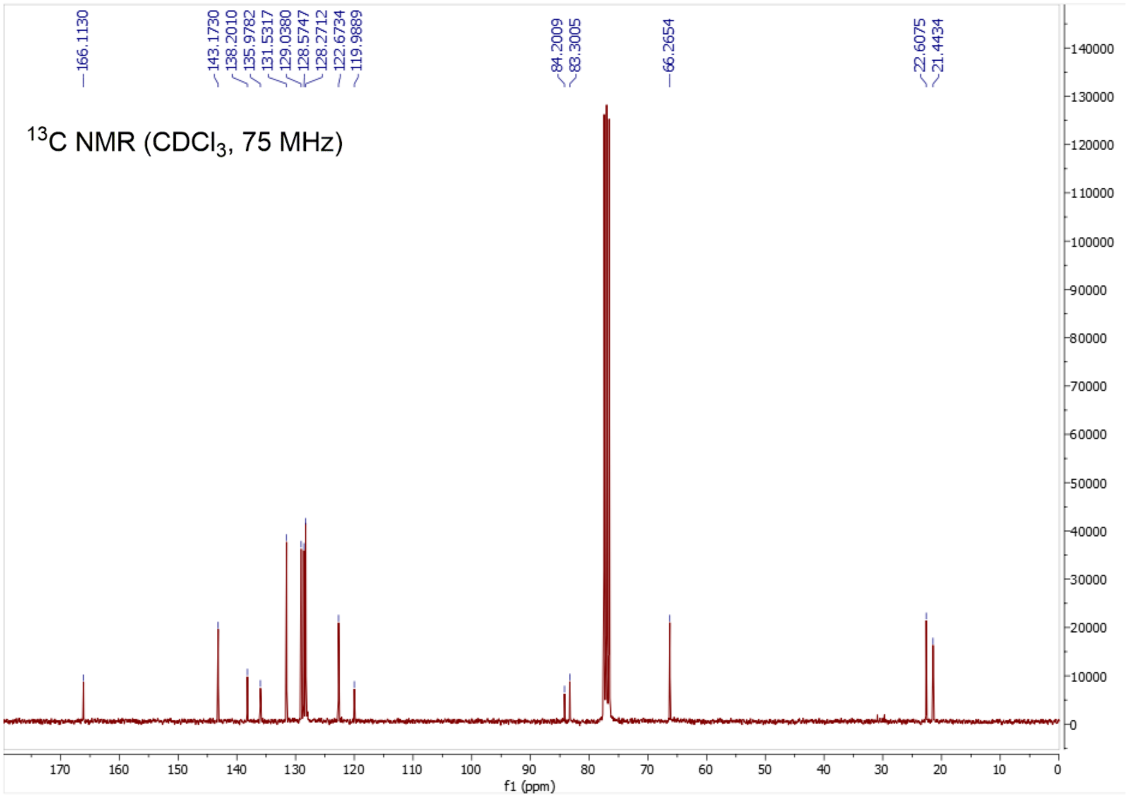
166.1130
143.1730
138.2010
135.9782
131.5317
129.0380
128.5747
128.2712
122.6734
119.9889
84.2009
83.3005
66.2654
22.6075
21.4434
13C NMR (CDCl3, 75 MHz)
f1 (ppm)

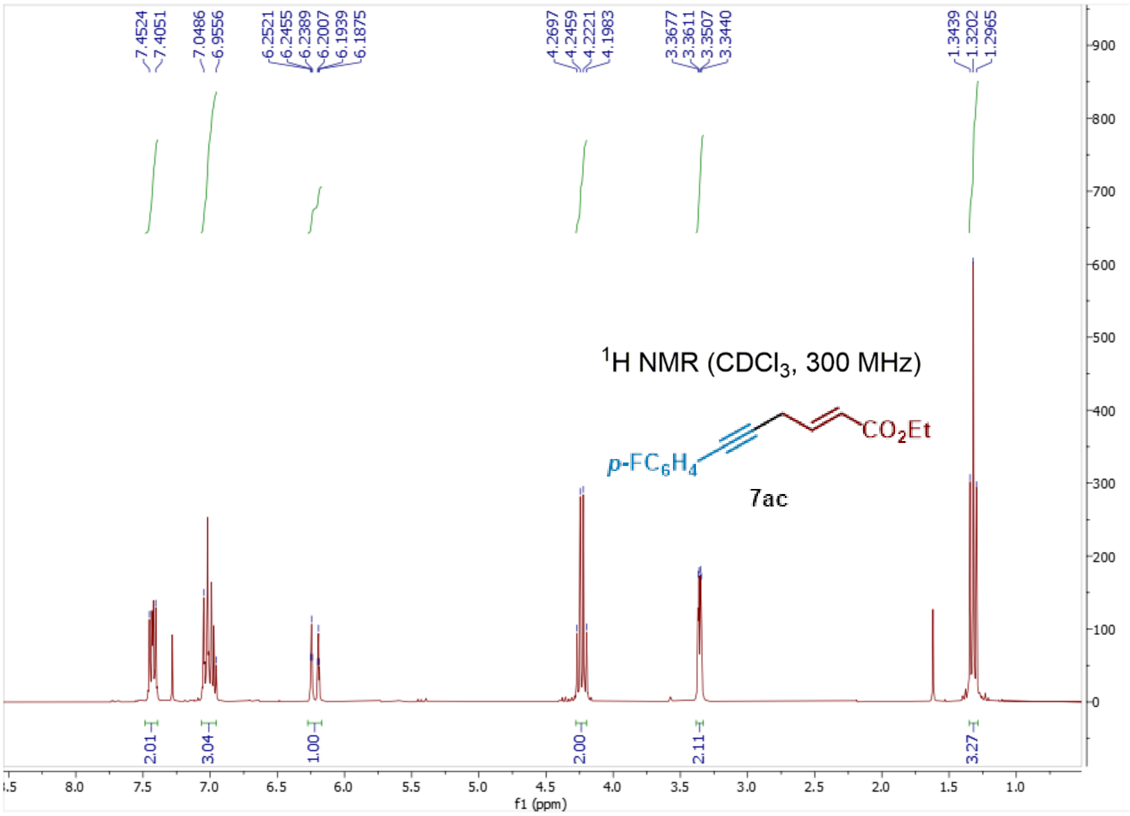
7.4524
7.4051
7.0486
6.9556
6.2521
6.2455
6.2389
6.2007
6.1939
6.1875
4.2697
4.2459
4.2221
4.1983
3.3677
3.3611
3.3507
3.3440
1.3439
1.3202
1.2965
$^{1}$H NMR ($CDCl_3$, 300 MHz)
p-$FC_6H_4$
$CO_2Et$
7ac
2.01
3.04
1.00
2.00
2.11
3.27
f1 (ppm)

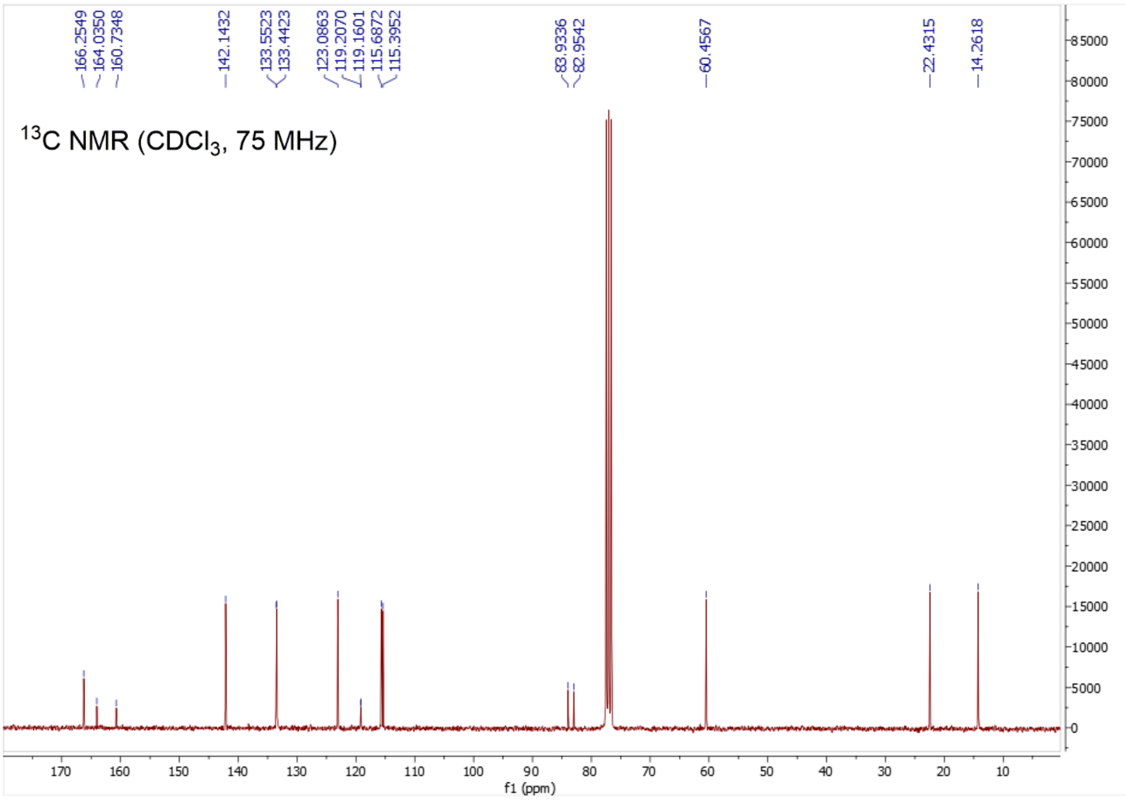
166.2549
164.0350
160.7348
142.1432
133.5523
133.4423
123.0863
119.2070
119.1601
115.6872
115.3952
83.9336
82.9542
60.4567
22.4315
14.2618
$^{13}$C NMR ($CDCl_3$, 75 MHz)
f1 (ppm)

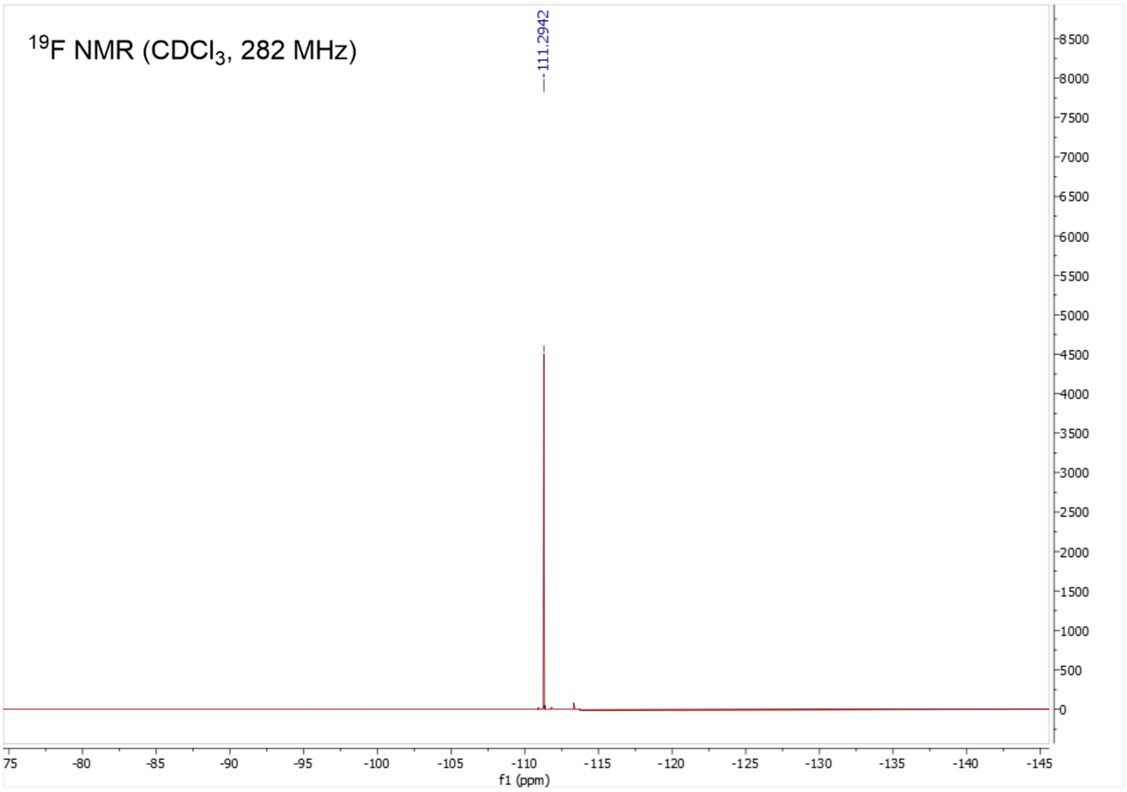

19F NMR (CDCl3, 282 MHz)
-111.2942
f1 (ppm)

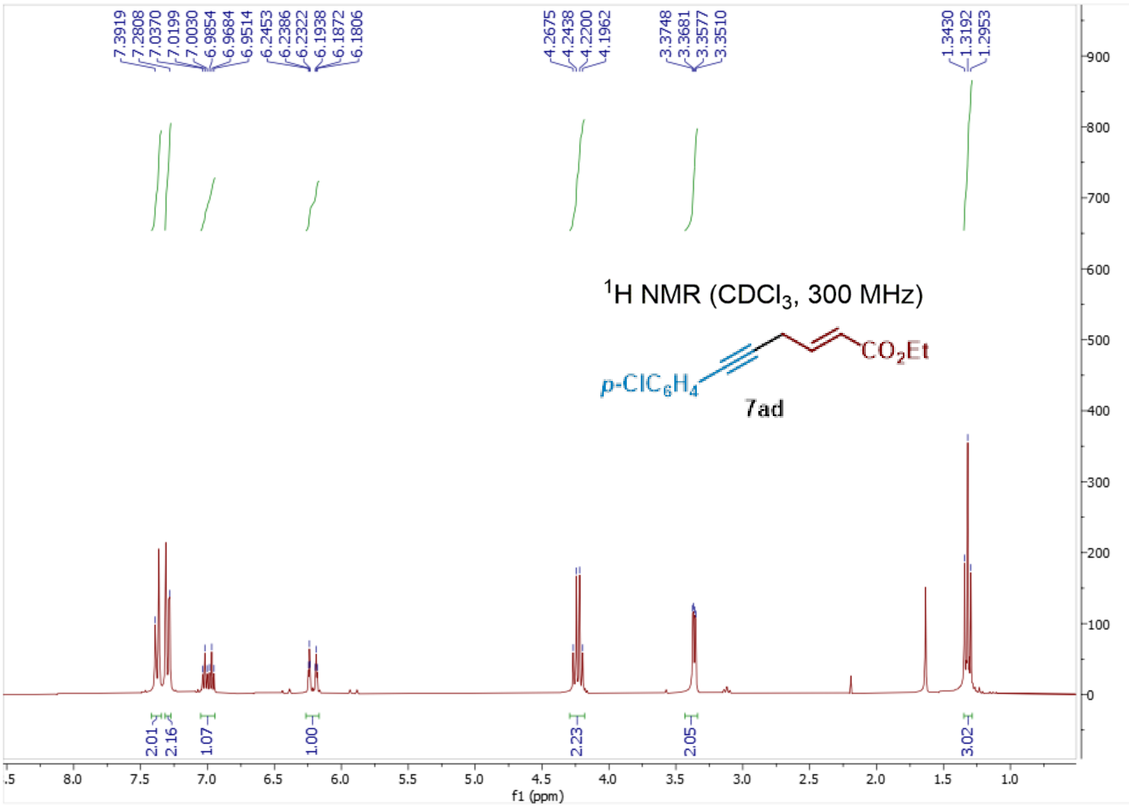

7.3919
7.2808
7.0370
7.0199
7.0030
6.9854
6.9684
6.9514
6.2453
6.2386
6.2322
6.1938
6.1872
6.1806
4.2675
4.2438
4.2200
4.1962
3.3748
3.3681
3.3577
3.3510
1.3430
1.3192
1.2953
1H NMR (CDCl3, 300 MHz)
p-ClC6H4
CO2Et
7ad
2.01
2.16
1.07
1.00
2.23
2.05
3.02
f1 (ppm)


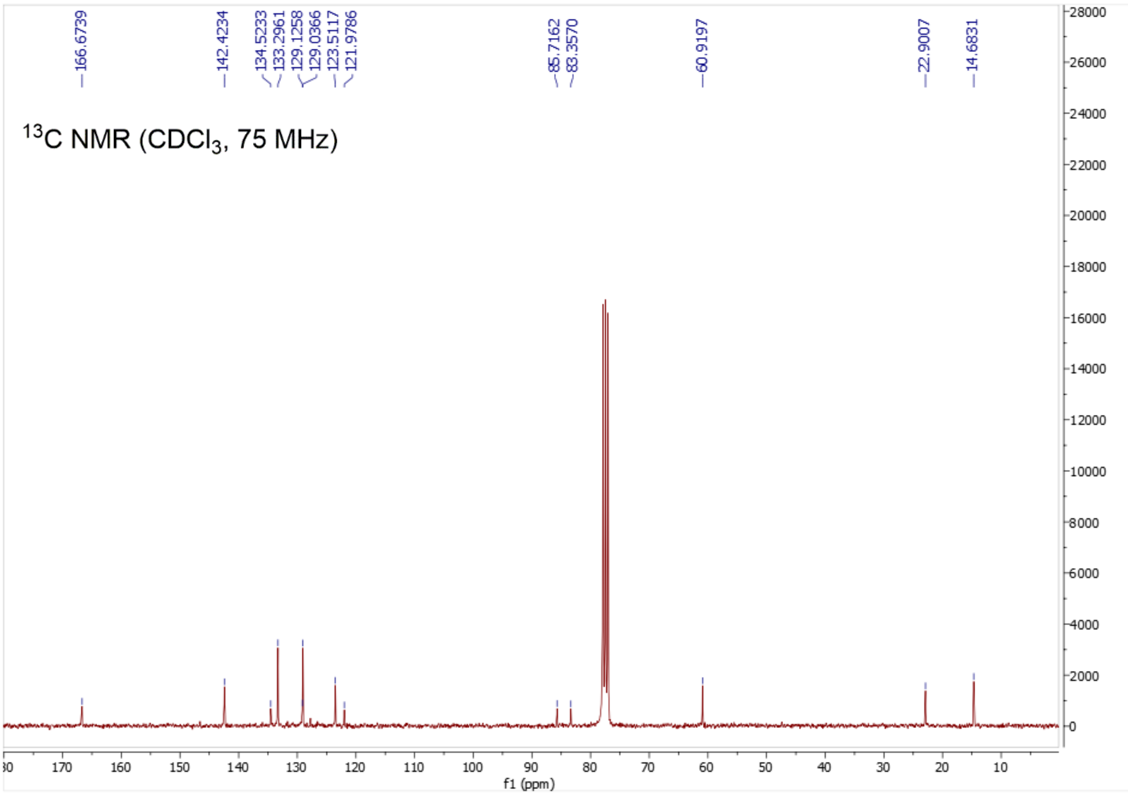

166.6739
142.4234
134.5233
133.2961
129.1258
129.0366
123.5117
121.9786
85.7162
83.3570
60.9197
22.9007
14.6831
13C NMR (CDCl3, 75 MHz)
f1 (ppm)

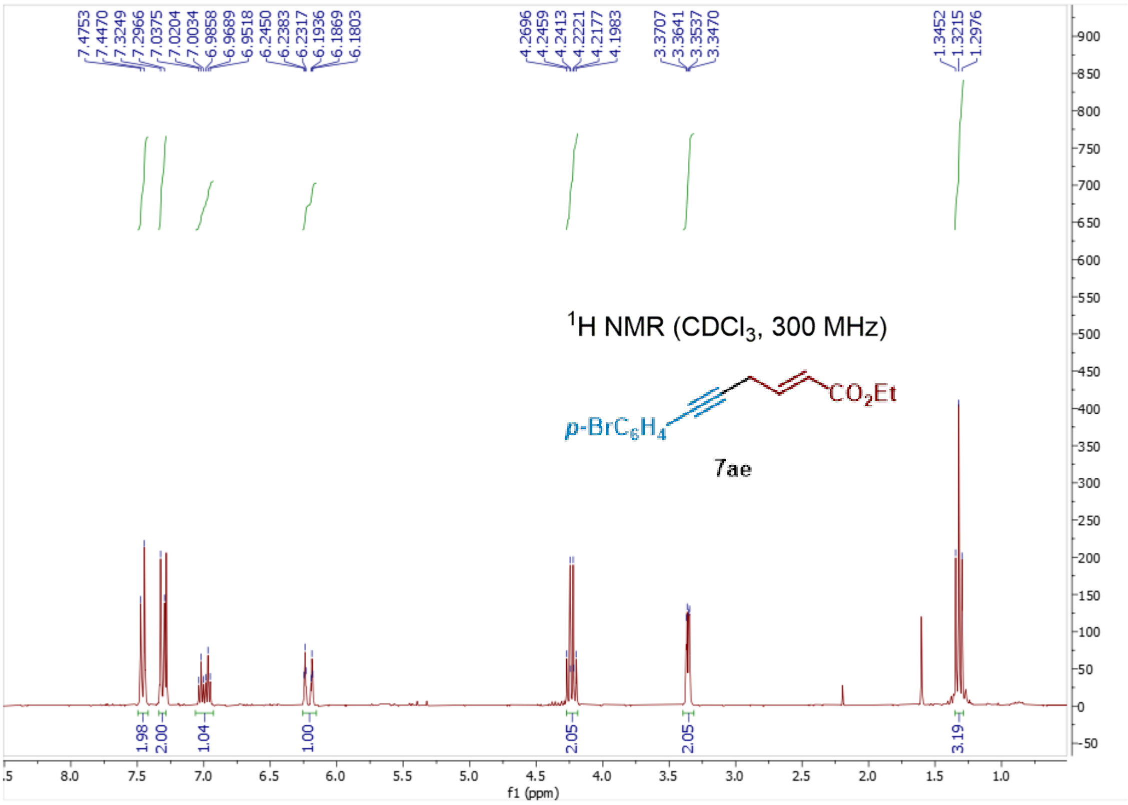

7.4753
7.4470
7.3249
7.2966
7.0375
7.0204
7.0034
6.9858
6.9689
6.9518
6.2450
6.2383
6.2317
6.1936
6.1869
6.1803
4.2696
4.2459
4.2413
4.2221
4.2177
4.1983
3.3707
3.3641
3.3537
3.3470
1.3452
1.3215
1.2976
$^1$H NMR ($CDCl_3$, 300 MHz)
p-BrC6H4
CO2Et
7ae
1.98
2.00
1.04
1.00
2.05
2.05
3.19
f1 (ppm)


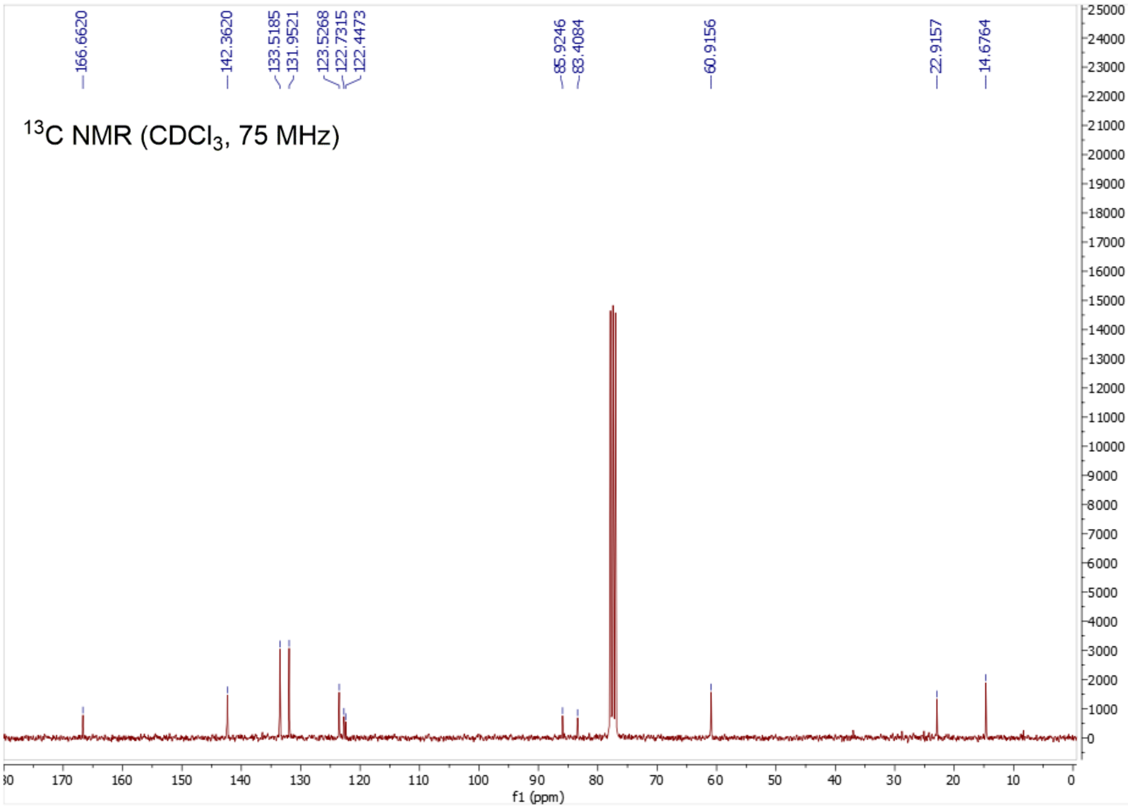

166.6620
142.3620
133.5185
131.9521
123.5268
122.7315
122.4473
85.9246
83.4084
60.9156
22.9157
14.6764
$^{13}$C NMR ($CDCl_3$, 75 MHz)
f1 (ppm)

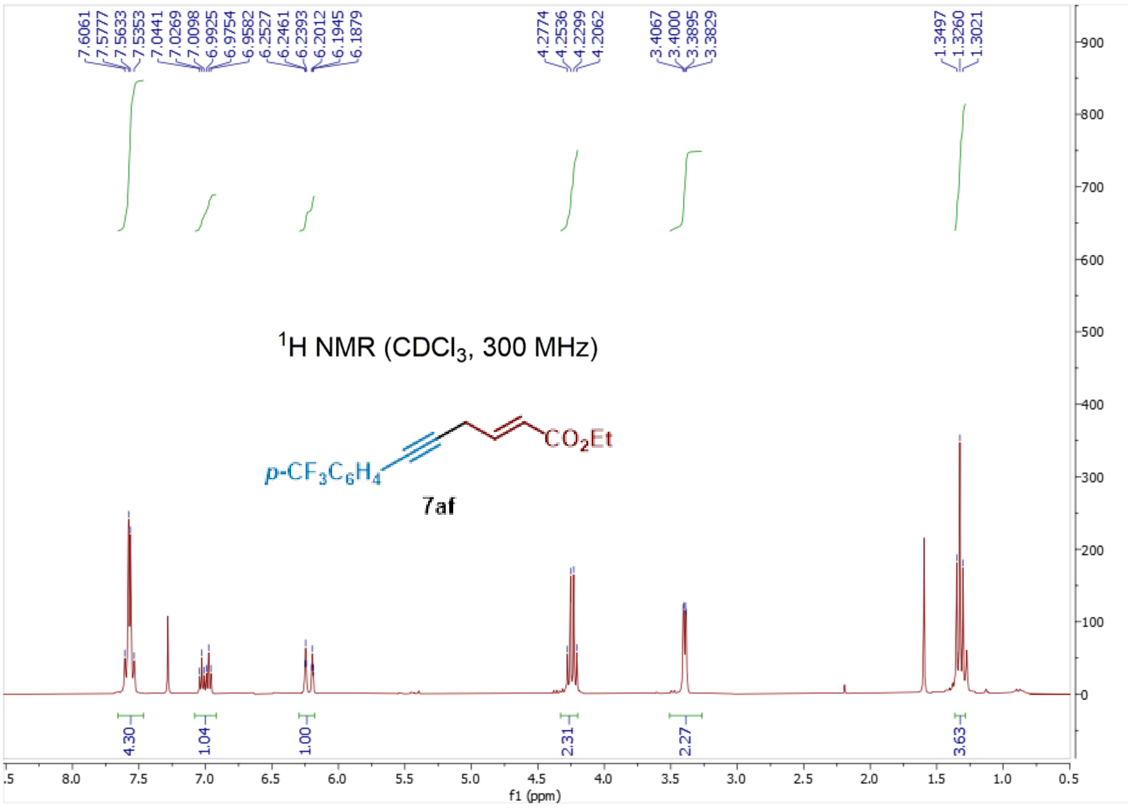
7.6061
7.5777
7.5633
7.5353
7.0441
7.0269
7.0098
6.9925
6.9754
6.9582
6.2527
6.2461
6.2393
6.2012
6.1945
6.1879
4.2774
4.2536
4.2299
4.2062
3.4067
3.4000
3.3895
3.3829
1.3497
1.3260
1.3021
1H NMR (CDCl3, 300 MHz)
p-CF3C6H4
CO2Et
7af
4.30
1.04
1.00
2.31
2.27
3.63
f1 (ppm)

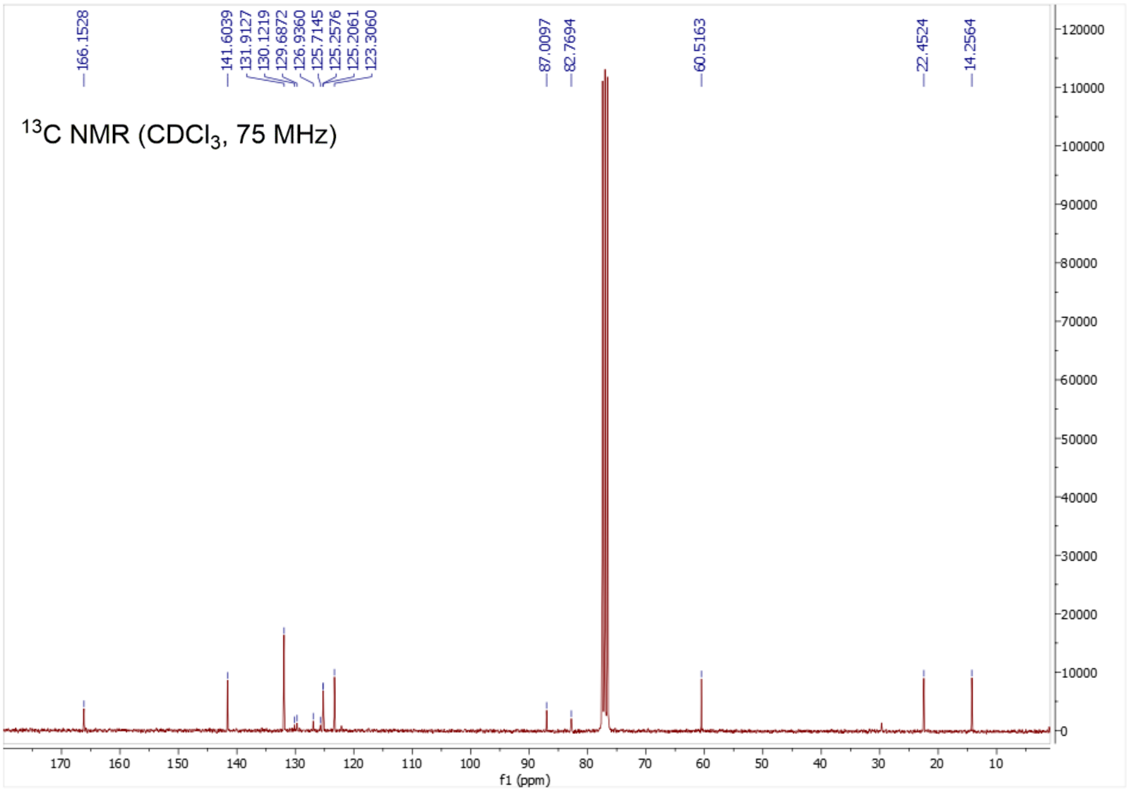
166.1528
141.6039
131.9127
130.1219
129.6872
126.9360
125.7145
125.2576
125.2061
123.3060
87.0097
82.7694
60.5163
22.4524
14.2564
13C NMR (CDCl3, 75 MHz)
f1 (ppm)

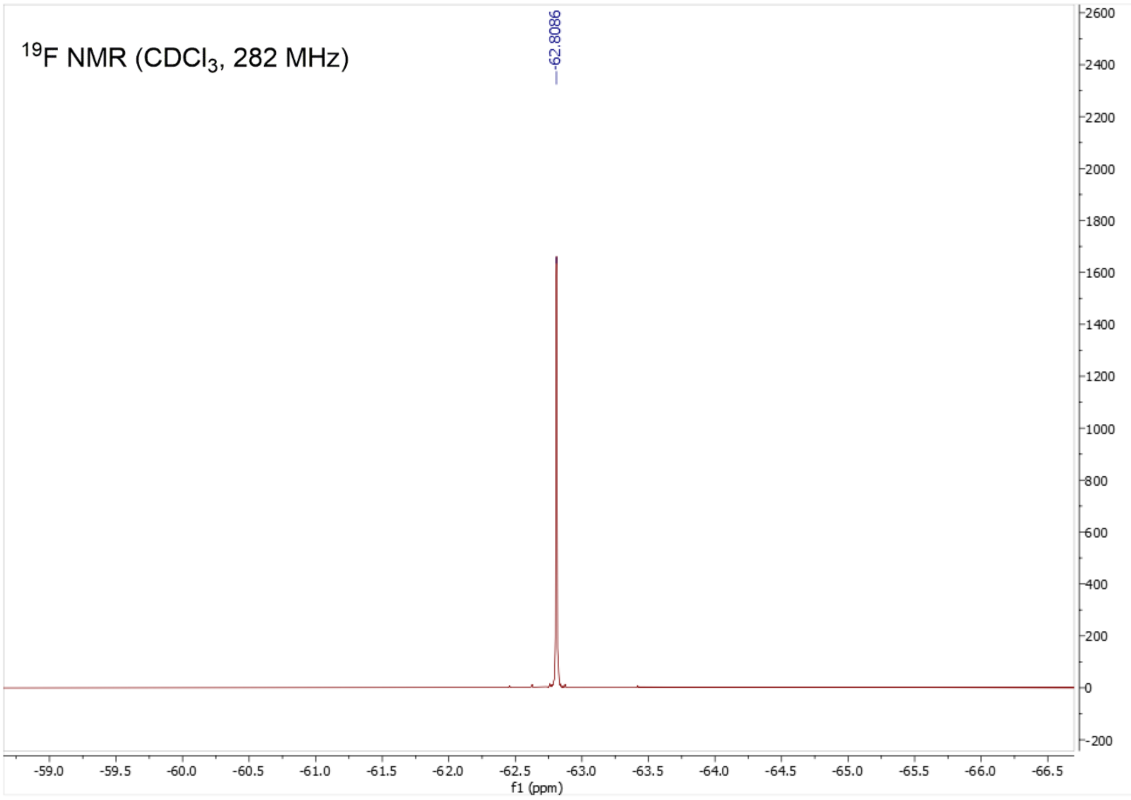

19F NMR (CDCl3, 282 MHz)
-62.8086
f1 (ppm)

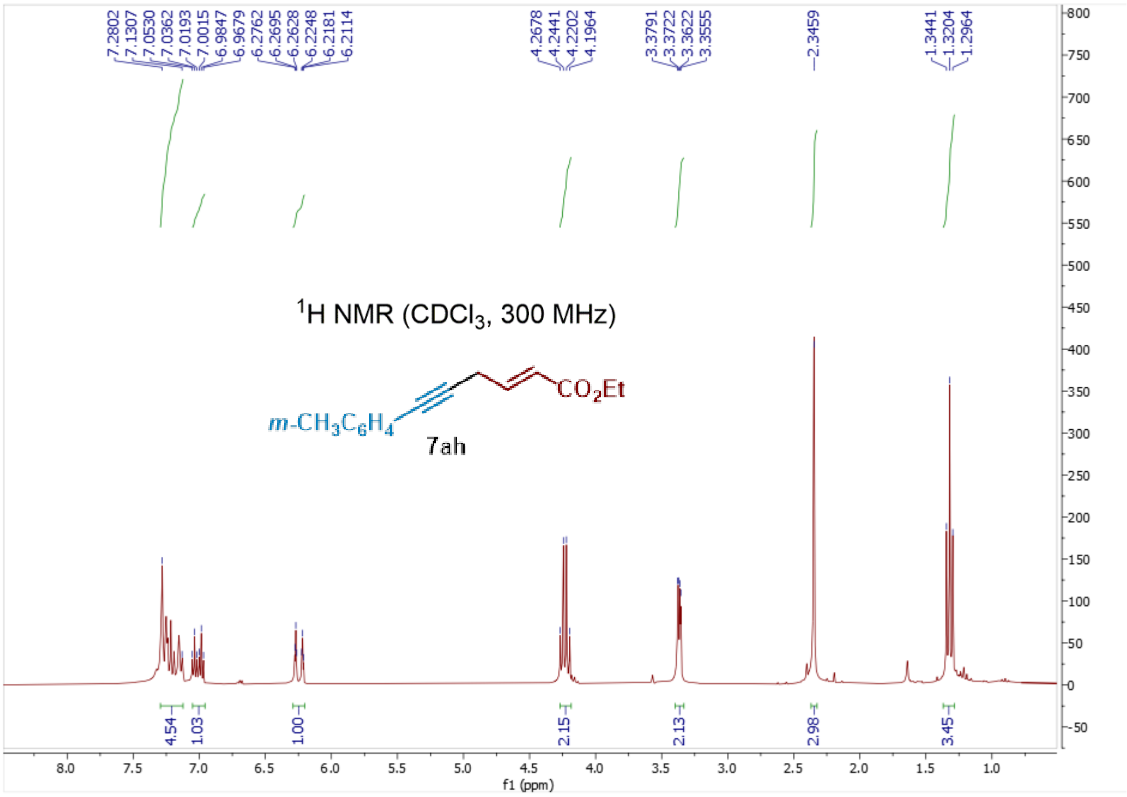
7.2802
7.1307
7.0530
7.0362
7.0193
7.0015
6.9847
6.9679
6.2762
6.2695
6.2628
6.2248
6.2181
6.2114
4.2678
4.2441
4.2202
4.1964
3.3791
3.3722
3.3622
3.3555
2.3459
1.3441
1.3204
1.2964
1H NMR (CDCl3, 300 MHz)
CO2Et
m-CH3C6H4
7ah
4.54
1.03
1.00
2.15
2.13
2.98
3.45
f1 (ppm)

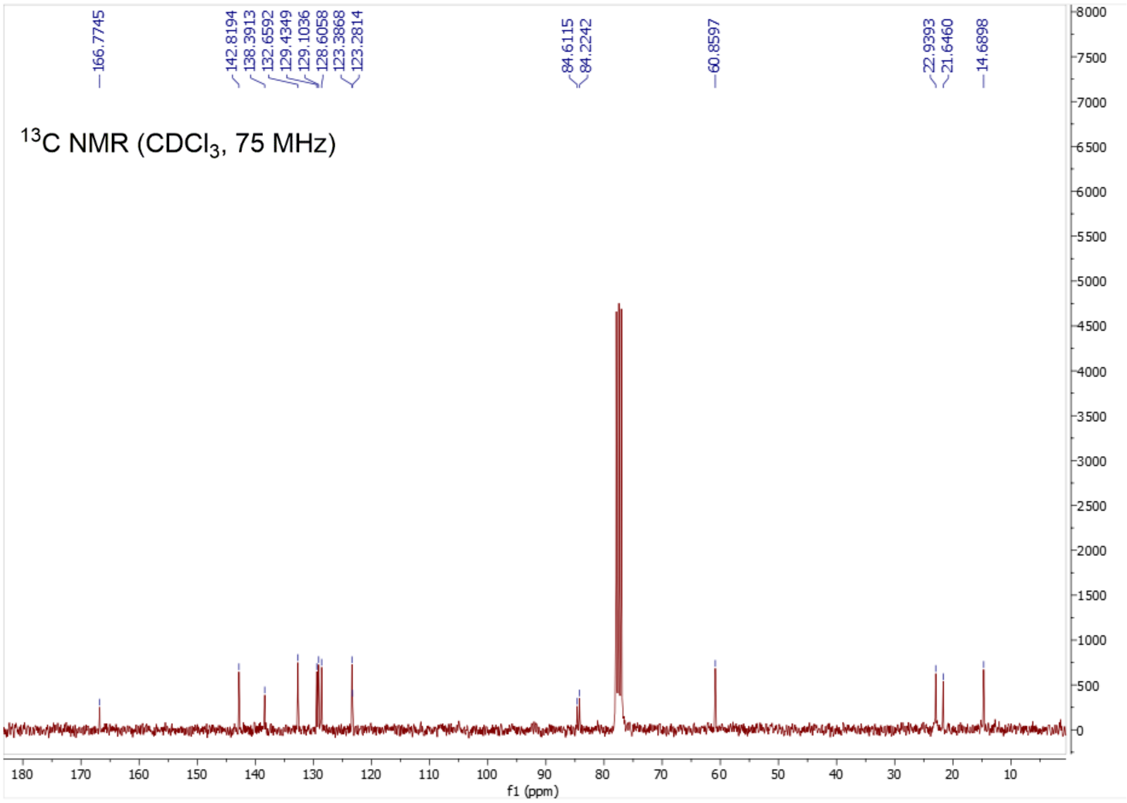
166.7745
142.8194
138.3913
132.6592
129.4349
129.1036
128.6058
123.3868
123.2814
84.6115
84.2242
60.8597
22.9393
21.6460
14.6898
13C NMR (CDCl3, 75 MHz)
f1 (ppm)

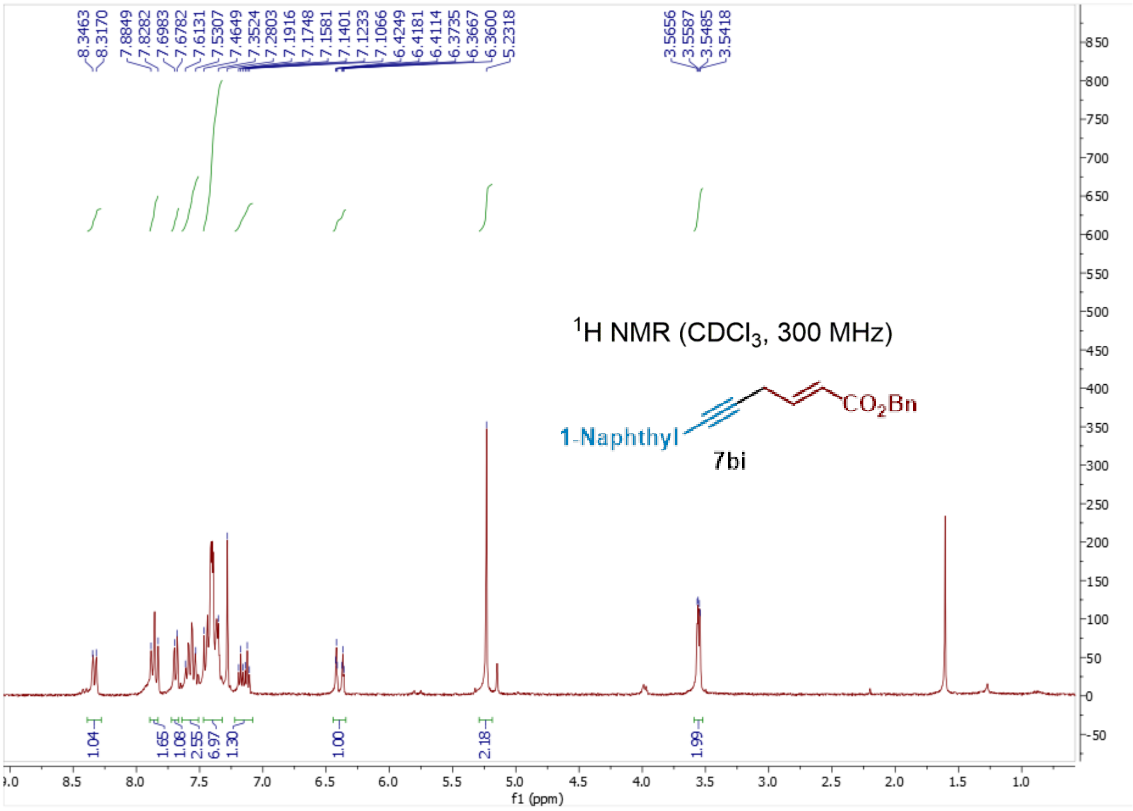

8.3463
8.3170
7.8849
7.8282
7.6983
7.6782
7.6131
7.5307
7.4649
7.3524
7.2803
7.1916
7.1748
7.1581
7.1401
7.1233
7.1066
6.4249
6.4181
6.4114
6.3735
6.3667
6.3600
5.2318
3.5656
3.5587
3.5485
3.5418
$^1$H NMR (CDCl$_3$, 300 MHz)
1-Naphthyl
CO$_2$Bn
7bi
1.04
1.65
1.08
2.55
6.97
1.30
1.00
2.18
1.99
f1 (ppm)


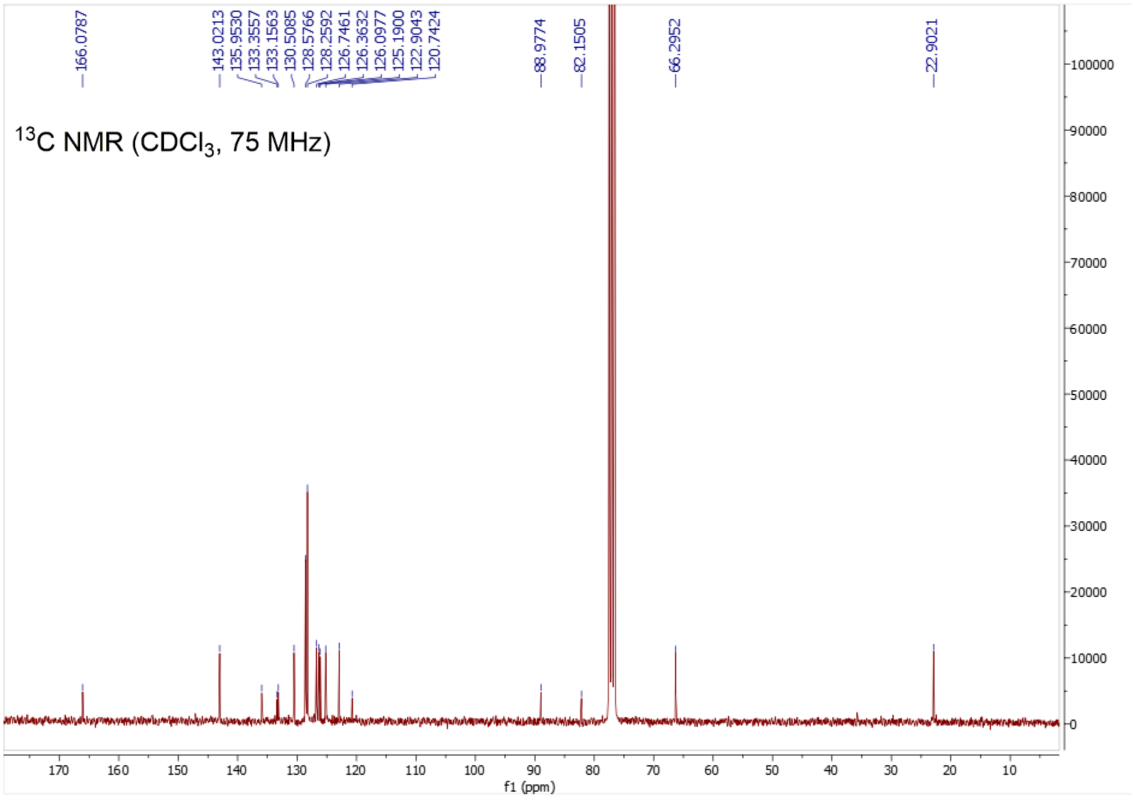

166.0787
143.0213
135.9530
133.3557
133.1563
130.5085
128.5766
128.2592
126.7461
126.3632
126.0977
125.1900
122.9043
120.7424
88.9774
82.1505
66.2952
22.9021
$^{13}$C NMR (CDCl$_3$, 75 MHz)
f1 (ppm)

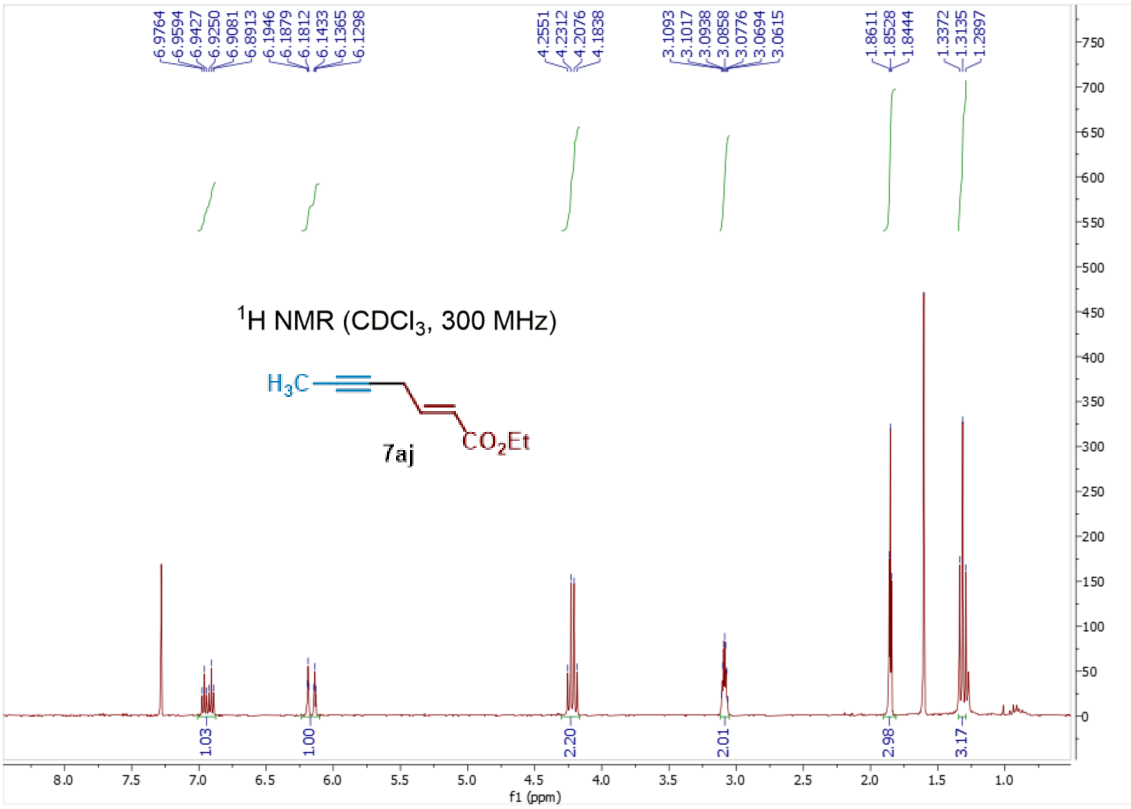
1H NMR (CDCl3, 300 MHz)
H3C
CO2Et
7aj
6.9764
6.9594
6.9427
6.9250
6.9081
6.8913
6.1946
6.1879
6.1812
6.1433
6.1365
6.1298
4.2551
4.2312
4.2076
4.1838
3.1093
3.1017
3.0938
3.0858
3.0776
3.0694
3.0615
1.8611
1.8528
1.8444
1.3372
1.3135
1.2897
1.03
1.00
2.20
2.01
2.98
3.17
f1 (ppm)

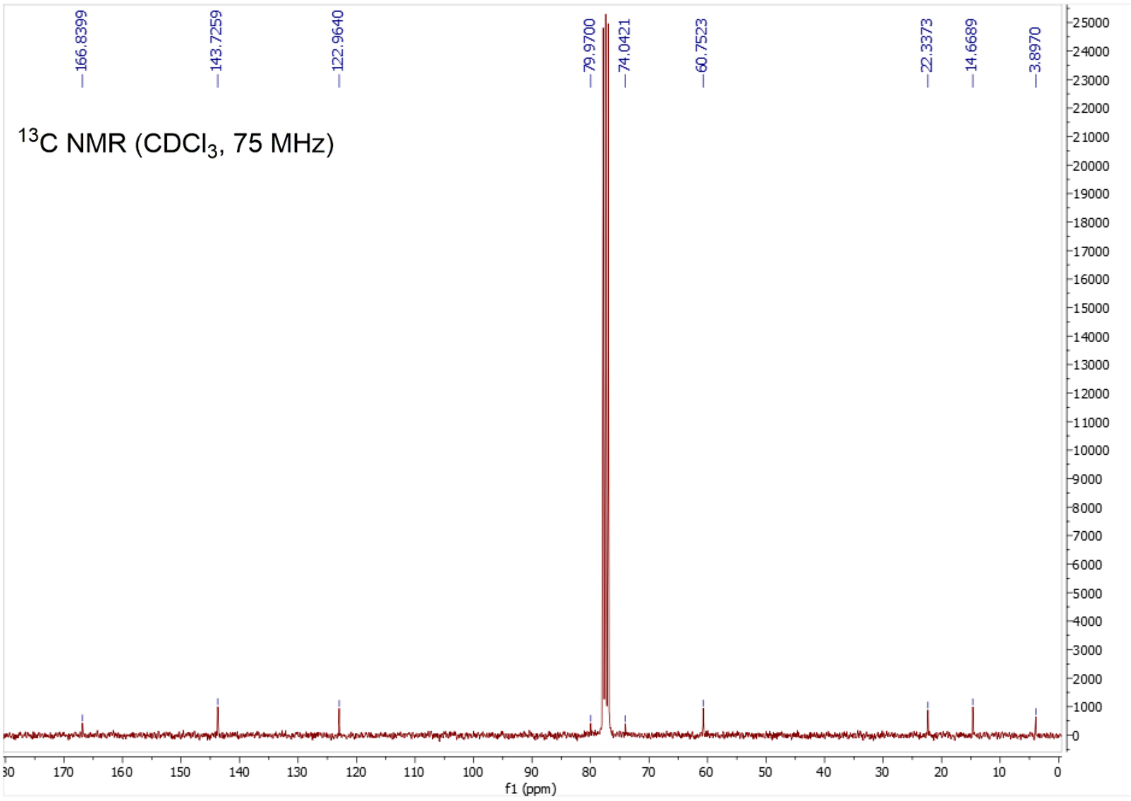
13C NMR (CDCl3, 75 MHz)
166.8399
143.7259
122.9640
79.9700
74.0421
60.7523
22.3373
14.6689
3.8970
f1 (ppm)

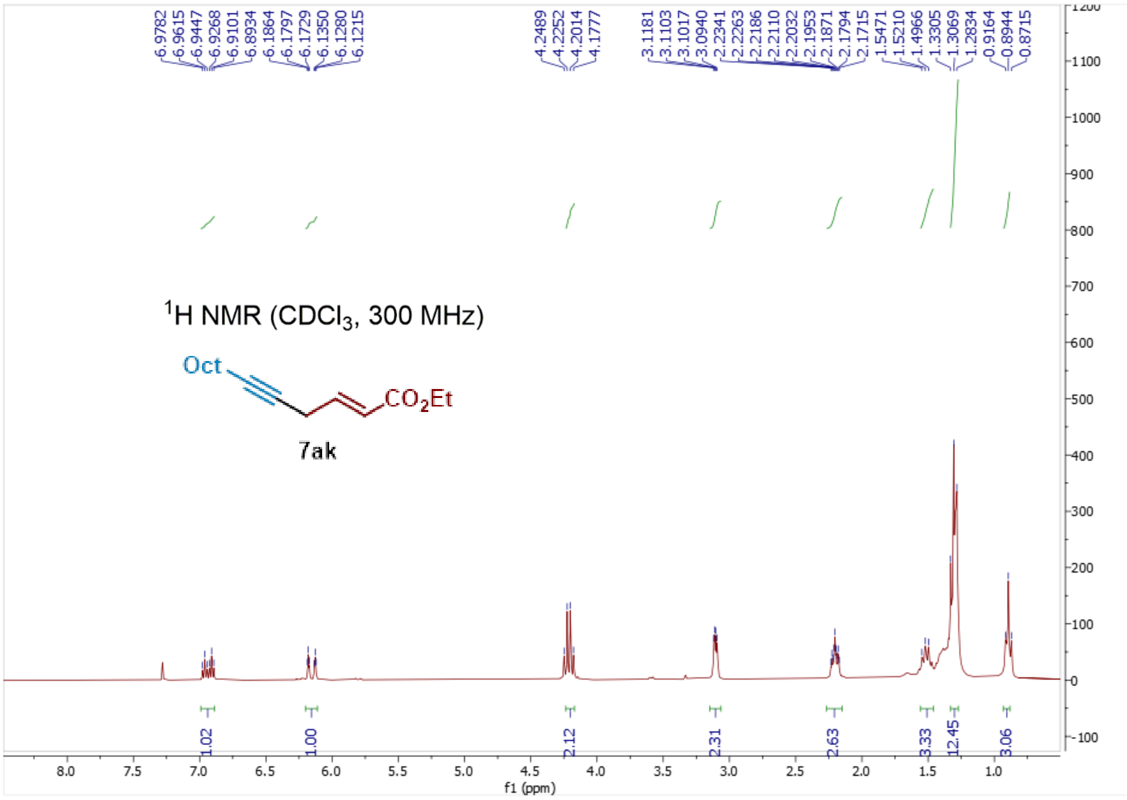
6.9782
6.9615
6.9447
6.9268
6.9101
6.8934
6.1864
6.1797
6.1729
6.1350
6.1280
6.1215
4.2489
4.2252
4.2014
4.1777
3.1181
3.1103
3.1017
3.0940
2.2341
2.2263
2.2186
2.2110
2.2032
2.1953
2.1871
2.1794
2.1715
1.5471
1.5210
1.4966
1.3305
1.3069
1.2834
0.9164
0.8944
0.8715
$^1$H NMR ($CDCl_3$, 300 MHz)
Oct
$CO_2Et$
7ak
1.02
1.00
2.12
2.31
2.63
3.33
12.45
3.06
f1 (ppm)

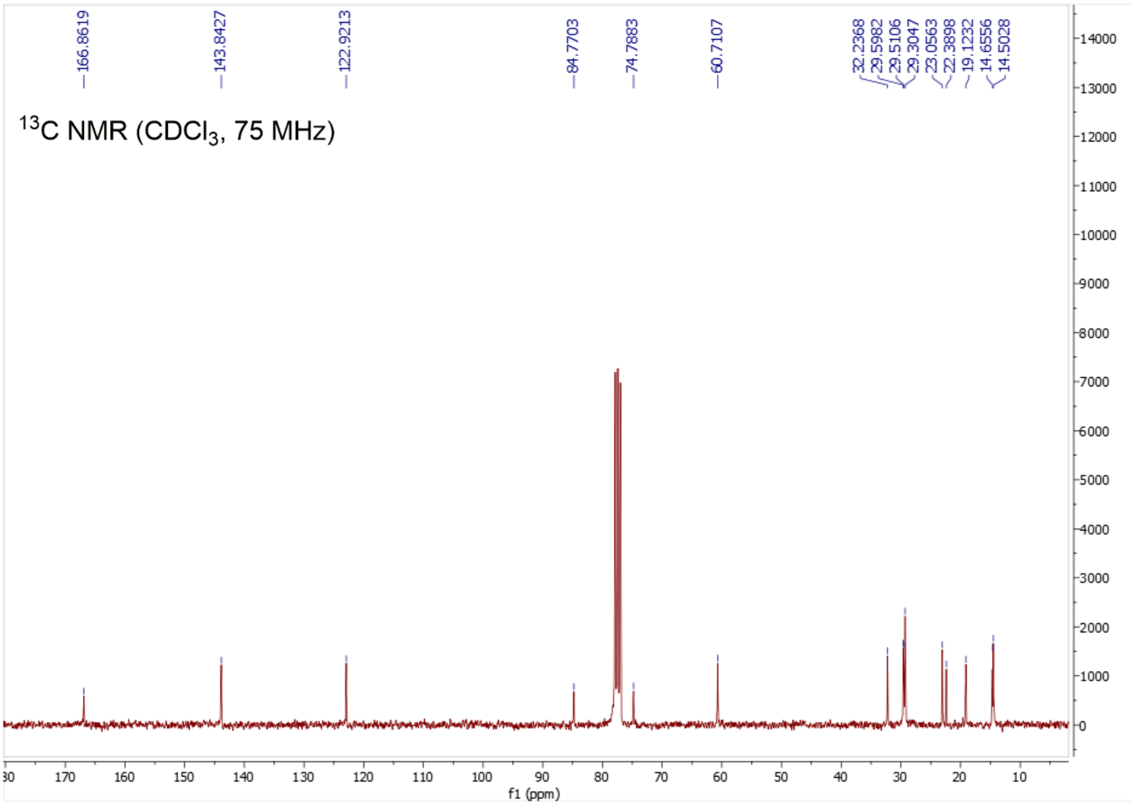
166.8619
143.8427
122.9213
84.7703
74.7883
60.7107
32.2368
29.5982
29.5106
29.3047
23.0563
22.3898
19.1232
14.6556
14.5028
$^{13}$C NMR ($CDCl_3$, 75 MHz)
f1 (ppm)

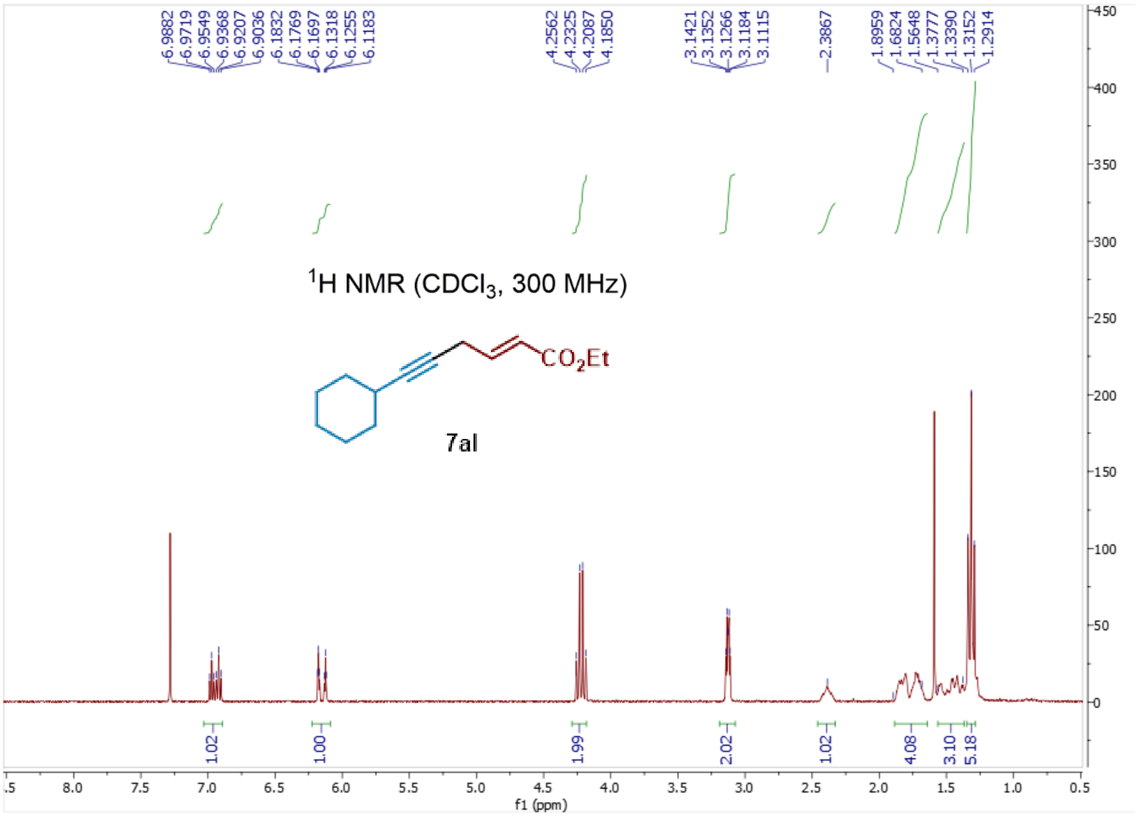
6.9882
6.9719
6.9549
6.9368
6.9207
6.9036
6.1832
6.1769
6.1697
6.1318
6.1255
6.1183
4.2562
4.2325
4.2087
4.1850
3.1421
3.1352
3.1266
3.1184
3.1115
2.3867
1.8959
1.6824
1.5648
1.3777
1.3390
1.3152
1.2914
1H NMR (CDCl3, 300 MHz)
CO2Et
7al
1.02
1.00
1.99
2.02
1.02
4.08
3.10
5.18
f1 (ppm)

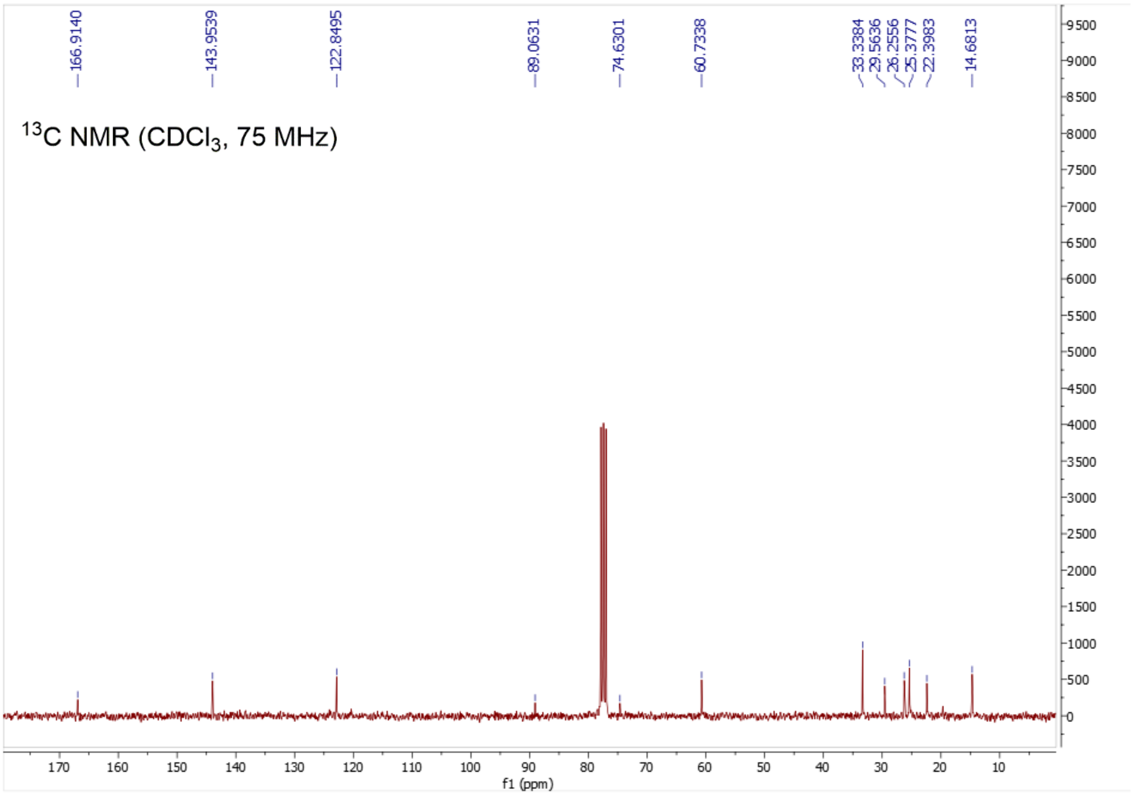
166.9140
143.9539
122.8495
89.0631
74.6301
60.7338
33.3384
29.5636
26.2556
25.3777
22.3983
14.6813
13C NMR (CDCl3, 75 MHz)
f1 (ppm)

## 14. HRMS Analysis Reports

### HRMS compound 3aa

# Mass Spectrum SmartFormula Report

**Analysis Info**

| | | | | |
|---|---|---|---|---|
| Analysis Name | D:\Data\KY_298_BA1_01_5454.d | Acquisition Date | 11/08/2020 12:52:59 | |
| Method | FantasmaMASAEXACTA_POS.m | Operator | Demo User | |
| Sample Name | KY_298 | Instrument | impact II | 1825265.10101 |
| Comment | | | | |

**Acquisition Parameter**

| | | | | | |
|---|---|---|---|---|---|
| Source Type | ESI | Ion Polarity | Positive | Set Nebulizer | 2.4 Bar |
| Focus | Active | Set Capillary | 3500 V | Set Dry Heater | 250 °C |
| Scan Begin | 50 m/z | Set End Plate Offset | -500 V | Set Dry Gas | 6.0 l/min |
| Scan End | 1500 m/z | Set Charging Voltage | 2000 V | Set Divert Valve | Source |
| | | Set Corona | 0 nA | Set APCI Heater | 0 °C |

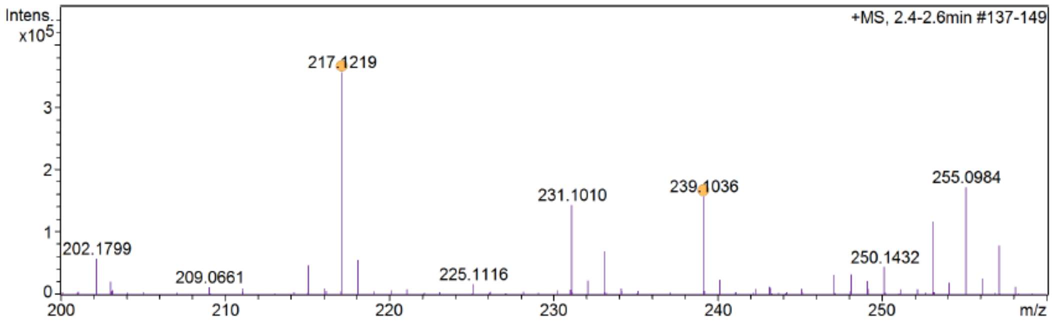


| Meas. m/z | # | Ion Formula | m/z | err [ppm] | mSigma | # mSigma | Score | rdb | e⁻ Conf | N-Rule | Adduct |
|---|---|---|---|---|---|---|---|---|---|---|---|
| 217.1219 | 1 | C14H17O2 | 217.1223 | 1.9 | 1.8 | 1 | 100.00 | 7.0 | even | ok | M+H |
| 239.1036 | 1 | C14H16NaO2 | 239.1043 | 2.5 | 6.0 | 1 | 100.00 | 7.0 | even | ok | M+Na |

### HRMS compound 3ab

# Mass Spectrum SmartFormula Report

**Analysis Info**

| | | | | |
|---|---|---|---|---|
| Analysis Name | D:\Data\KY_310_BA1_01_5452.d | Acquisition Date | 11/08/2020 12:38:18 | |
| Method | FantasmaMASAEXACTA_POS.m | Operator | Demo User | |
| Sample Name | KY_310 | Instrument | impact II | 1825265.10101 |
| Comment | | | | |

**Acquisition Parameter**

| | | | | | |
|---|---|---|---|---|---|
| Source Type | ESI | Ion Polarity | Positive | Set Nebulizer | 2.4 Bar |
| Focus | Active | Set Capillary | 3500 V | Set Dry Heater | 250 °C |
| Scan Begin | 50 m/z | Set End Plate Offset | -500 V | Set Dry Gas | 6.0 l/min |
| Scan End | 1500 m/z | Set Charging Voltage | 2000 V | Set Divert Valve | Source |
| | | Set Corona | 0 nA | Set APCI Heater | 0 °C |

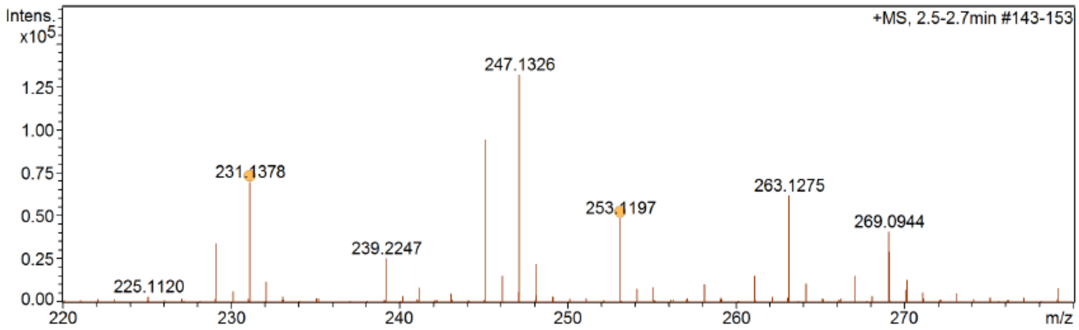


| Meas. m/z | # | Ion Formula | m/z | err [ppm] | mSigma | # mSigma | Score | rdb | e⁻ Conf | N-Rule | Adduct |
|---|---|---|---|---|---|---|---|---|---|---|---|
| 231.1378 | 1 | C15H19O2 | 231.1380 | 0.5 | 1.3 | 1 | 100.00 | 7.0 | even | ok | M+H |
| 253.1197 | 1 | C15H18NaO2 | 253.1199 | 0.8 | 5.2 | 1 | 100.00 | 7.0 | even | ok | M+Na |

## HRMS compound 3ac

# Mass Spectrum SmartFormula Report

**Analysis Info**

| | | | |
|---|---|---|---|
| Analysis Name | D:\Data\KY_311_BA1_01_5449.d | Acquisition Date | 11/08/2020 11:37:25 |
| Method | FantasmaMASAEXACTA_POS.m | Operator | Demo User |
| Sample Name | KY_311 | Instrument | impact II 1825265.10101 |
| Comment | | | |

**Acquisition Parameter**

| | | | | | |
|---|---|---|---|---|---|
| Source Type | ESI | Ion Polarity | Positive | Set Nebulizer | 2.4 Bar |
| Focus | Active | Set Capillary | 3500 V | Set Dry Heater | 250 °C |
| Scan Begin | 50 m/z | Set End Plate Offset | -500 V | Set Dry Gas | 6.0 l/min |
| Scan End | 1500 m/z | Set Charging Voltage | 2000 V | Set Divert Valve | Source |
| | | Set Corona | 0 nA | Set APCI Heater | 0 °C |

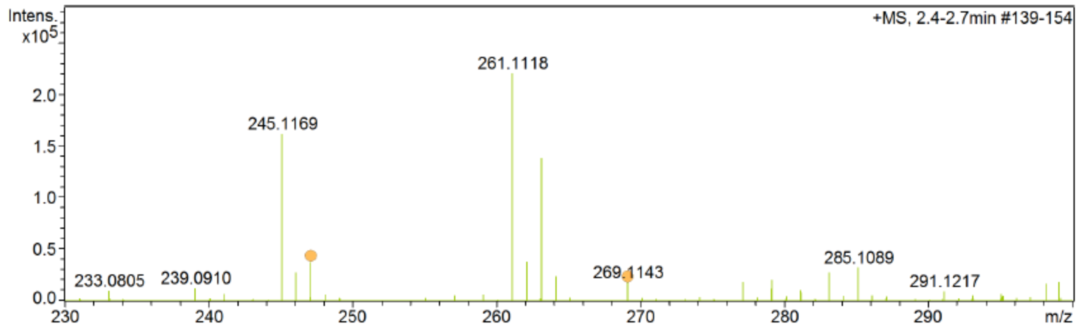


| Meas. m/z | # | Ion Formula | m/z | err [ppm] | mSigma | # mSigma | Score | rdb | e⁻ Conf | N-Rule | Adduct |
|---|---|---|---|---|---|---|---|---|---|---|---|
| 247.1321 | 1 | C15H19O3 | 247.1329 | 3.3 | 20.4 | 1 | 100.00 | 7.0 | even | ok | M+H |
| 269.1143 | 1 | C15H18NaO3 | 269.1148 | 2.0 | 51.9 | 1 | 100.00 | 7.0 | even | ok | M+Na |

## HRMS compound 3ad

# Mass Spectrum SmartFormula Report

**Analysis Info**

| | | | |
|---|---|---|---|
| Analysis Name | \\IMPACT_II-10101\Data\Masa_Exacta\OBG_441.d | Acquisition Date | 3/23/2021 3:47:32 PM |
| Method | MS_MASA_EXACTA.m | Operator | Demo User |
| Sample Name | OBG_441 | Instrument | impact II 1825265.10101 |
| Comment | | | |

**Acquisition Parameter**

| | | | | | |
|---|---|---|---|---|---|
| Source Type | ESI | Ion Polarity | Positive | Set Nebulizer | 2.4 Bar |
| Focus | Active | Set Capillary | 4000 V | Set Dry Heater | 250 °C |
| Scan Begin | 50 m/z | Set End Plate Offset | -500 V | Set Dry Gas | 6.0 l/min |
| Scan End | 1500 m/z | Set Charging Voltage | 2000 V | Set Divert Valve | Source |
| | | Set Corona | 0 nA | Set APCI Heater | 0 °C |

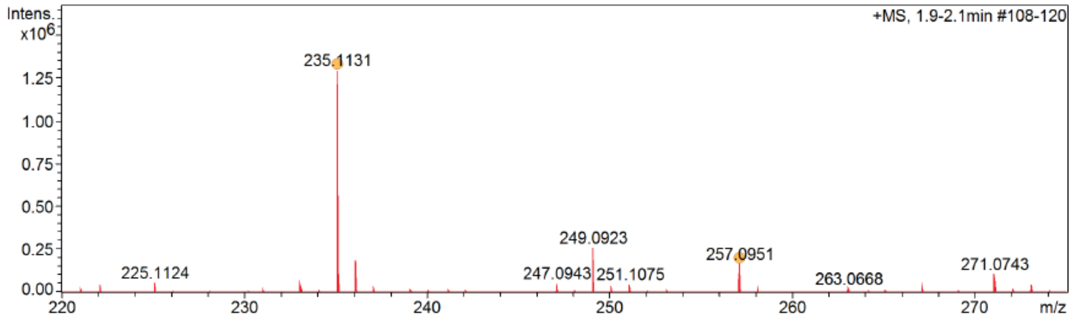


| Meas. m/z | # | Ion Formula | m/z | err [ppm] | mSigma | # mSigma | Score | rdb | e⁻ Conf | N-Rule | Adduct |
|---|---|---|---|---|---|---|---|---|---|---|---|
| 235.1131 | 1 | C14H16FO2 | 235.1129 | -0.9 | 3.4 | 1 | 100.00 | 7.0 | even | ok | M+H |
| 257.0951 | 1 | C14H15FNaO2 | 257.0948 | -1.2 | 0.8 | 1 | 100.00 | 7.0 | even | ok | M+Na |

## HRMS compound 3ae

# Mass Spectrum SmartFormula Report

**Analysis Info**

| | | | |
|---|---|---|---|
| Analysis Name | D:\Data\OBG_432_BA1_01_5447.d | Acquisition Date | 10/08/2020 13:47:54 |
| Method | FantasmaMASAEXACTA_POS.m | Operator | Demo User |
| Sample Name | OBG_432 | Instrument | impact II 1825265.10101 |
| Comment | | | |

**Acquisition Parameter**

| | | | | | |
|---|---|---|---|---|---|
| Source Type | ESI | Ion Polarity | Positive | Set Nebulizer | 2.4 Bar |
| Focus | Active | Set Capillary | 3500 V | Set Dry Heater | 250 °C |
| Scan Begin | 50 m/z | Set End Plate Offset | -500 V | Set Dry Gas | 6.0 l/min |
| Scan End | 1500 m/z | Set Charging Voltage | 2000 V | Set Divert Valve | Source |
| | | Set Corona | 0 nA | Set APCI Heater | 0 °C |

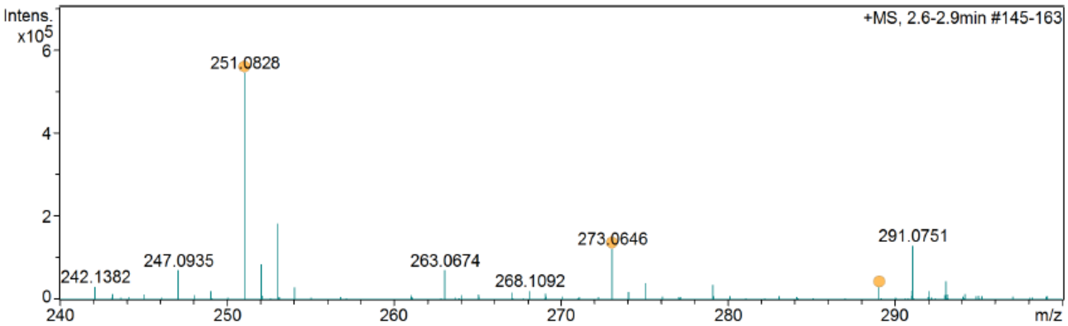


| Meas. m/z | # | Ion Formula | m/z | err [ppm] | mSigma | # mSigma | Score | rdb | $e^-$ Conf | N-Rule | Adduct |
|---|---|---|---|---|---|---|---|---|---|---|---|
| 251.0828 | 1 | C14H16ClO2 | 251.0833 | 2.2 | 1.9 | 1 | 100.00 | 7.0 | even | ok | M+H |
| 273.0646 | 1 | C14H15ClNaO2 | 273.0653 | 2.6 | 9.2 | 1 | 100.00 | 7.0 | even | ok | M+Na |
| 289.0392 | 1 | C14H15ClKO2 | 289.0392 | 0.0 | 185.7 | 1 | 100.00 | 7.0 | even | ok | M+K |

## HRMS compound 3af

# Mass Spectrum SmartFormula Report

**Analysis Info**

| | | | |
|---|---|---|---|
| Analysis Name | D:\Data\OBG_365_BA1_01_5443.d | Acquisition Date | 10/08/2020 13:03:03 |
| Method | FantasmaMASAEXACTA_POS.m | Operator | Demo User |
| Sample Name | OBG_365 | Instrument | impact II 1825265.10101 |
| Comment | | | |

**Acquisition Parameter**

| | | | | | |
|---|---|---|---|---|---|
| Source Type | ESI | Ion Polarity | Positive | Set Nebulizer | 2.4 Bar |
| Focus | Active | Set Capillary | 3500 V | Set Dry Heater | 250 °C |
| Scan Begin | 50 m/z | Set End Plate Offset | -500 V | Set Dry Gas | 6.0 l/min |
| Scan End | 1500 m/z | Set Charging Voltage | 2000 V | Set Divert Valve | Source |
| | | Set Corona | 0 nA | Set APCI Heater | 0 °C |

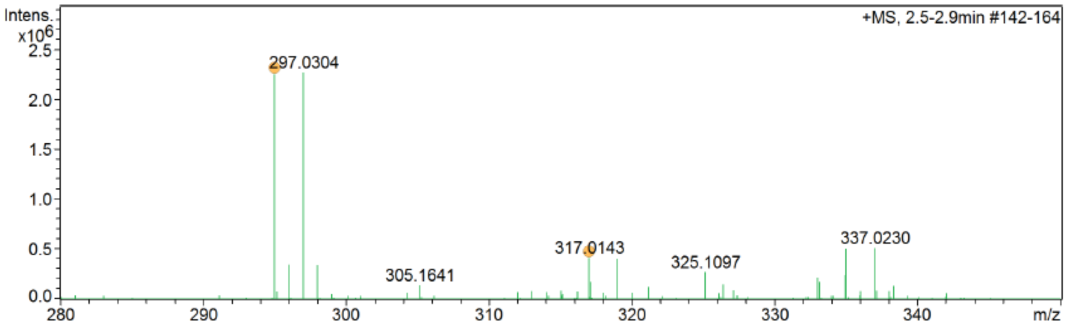


| Meas. m/z | # | Ion Formula | m/z | err [ppm] | mSigma | # mSigma | Score | rdb | $e^-$ Conf | N-Rule | Adduct |
|---|---|---|---|---|---|---|---|---|---|---|---|
| 295.0324 | 1 | C14H16BrO2 | 295.0328 | 1.5 | 7.0 | 1 | 100.00 | 7.0 | even | ok | M+H |
| 317.0143 | 1 | C14H15BrNaO2 | 317.0148 | 1.6 | 9.3 | 1 | 100.00 | 7.0 | even | ok | M+Na |

## HRMS compound 3ag

# Mass Spectrum SmartFormula Report

**Analysis Info**

| | | | |
|---|---|---|---|
| Analysis Name | D:\Data\KY_316_BA1_01_5450.d | Acquisition Date | 11/08/2020 11:51:55 |
| Method | FantasmaMASAEXACTA_POS.m | Operator | Demo User |
| Sample Name | KY_316 | Instrument | impact II 1825265.10101 |
| Comment | | | |

**Acquisition Parameter**

| | | | | | |
|---|---|---|---|---|---|
| Source Type | ESI | Ion Polarity | Positive | Set Nebulizer | 2.4 Bar |
| Focus | Active | Set Capillary | 3500 V | Set Dry Heater | 250 °C |
| Scan Begin | 50 m/z | Set End Plate Offset | -500 V | Set Dry Gas | 6.0 l/min |
| Scan End | 1500 m/z | Set Charging Voltage | 2000 V | Set Divert Valve | Source |
| | | Set Corona | 0 nA | Set APCI Heater | 0 °C |

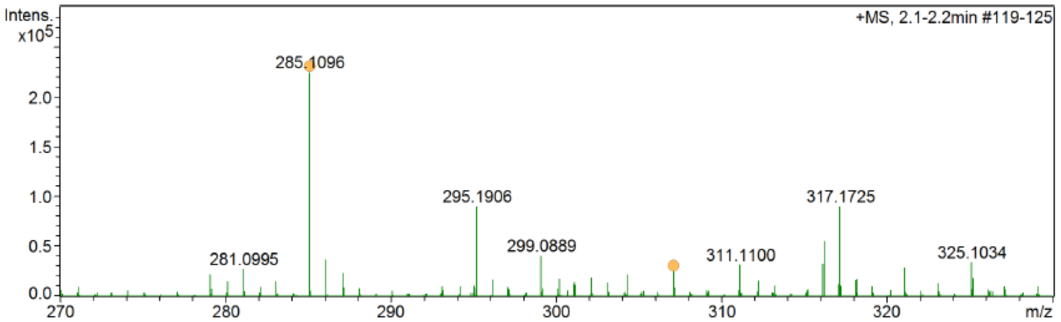


| Meas. m/z | # | Ion Formula | m/z | err [ppm] | mSigma | # mSigma | Score | rdb | e⁻ Conf | N-Rule | Adduct |
|---|---|---|---|---|---|---|---|---|---|---|---|
| 285.1096 | 1 | C15H16F3O2 | 285.1097 | 0.2 | 50.1 | 1 | 100.00 | 7.0 | even | ok | M+H |
| 307.0918 | 1 | C15H15F3NaO2 | 307.0916 | -0.6 | 127.1 | 1 | 100.00 | 7.0 | even | ok | M+Na |

## HRMS compound 3ah

# Mass Spectrum SmartFormula Report

**Analysis Info**

| | | | |
|---|---|---|---|
| Analysis Name | D:\Data\OBG_429_BA1_01_5442.d | Acquisition Date | 10/08/2020 12:51:39 |
| Method | FantasmaMASAEXACTA_POS.m | Operator | Demo User |
| Sample Name | OBG_429 | Instrument | impact II 1825265.10101 |
| Comment | | | |

**Acquisition Parameter**

| | | | | | |
|---|---|---|---|---|---|
| Source Type | ESI | Ion Polarity | Positive | Set Nebulizer | 2.4 Bar |
| Focus | Active | Set Capillary | 3500 V | Set Dry Heater | 250 °C |
| Scan Begin | 50 m/z | Set End Plate Offset | -500 V | Set Dry Gas | 6.0 l/min |
| Scan End | 1500 m/z | Set Charging Voltage | 2000 V | Set Divert Valve | Source |
| | | Set Corona | 0 nA | Set APCI Heater | 0 °C |

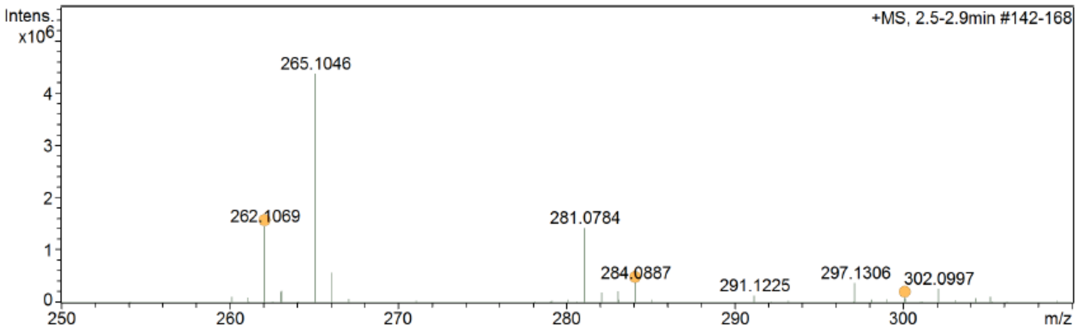


| Meas. m/z | # | Ion Formula | m/z | err [ppm] | mSigma | # mSigma | Score | rdb | e⁻ Conf | N-Rule | Adduct |
|---|---|---|---|---|---|---|---|---|---|---|---|
| 262.1069 | 1 | C14H16NO4 | 262.1074 | 1.9 | 1.1 | 1 | 100.00 | 8.0 | even | ok | M+H |
| 284.0887 | 1 | C14H15NNaO4 | 284.0893 | 2.4 | 1.2 | 1 | 100.00 | 8.0 | even | ok | M+Na |
| 300.0628 | 1 | C14H15KNO4 | 300.0633 | 1.4 | 48.0 | 2 | 100.00 | 8.0 | even | ok | M+K |

## HRMS compound 3ai

# Mass Spectrum SmartFormula Report

**Analysis Info**

| | | | |
|---|---|---|---|
| Analysis Name | D:\Data\Masa_Exacta\OBG_445.d | Acquisition Date | 21/12/2020 13:23:30 |
| Method | MS 50-1000 orgánica.m | Operator | Operator |
| Sample Name | OBG_445 | Instrument | impact II 1825265.10101 |
| Comment | | | |

**Acquisition Parameter**

| | | | | | |
|---|---|---|---|---|---|
| Source Type | ESI | Ion Polarity | Positive | Set Nebulizer | 2.5 Bar |
| Focus | Active | Set Capillary | 4500 V | Set Dry Heater | 250 °C |
| Scan Begin | 50 m/z | Set End Plate Offset | -500 V | Set Dry Gas | 6.0 l/min |
| Scan End | 1500 m/z | Set Charging Voltage | 2000 V | Set Divert Valve | Source |
| | | Set Corona | 0 nA | Set APCI Heater | 0 °C |

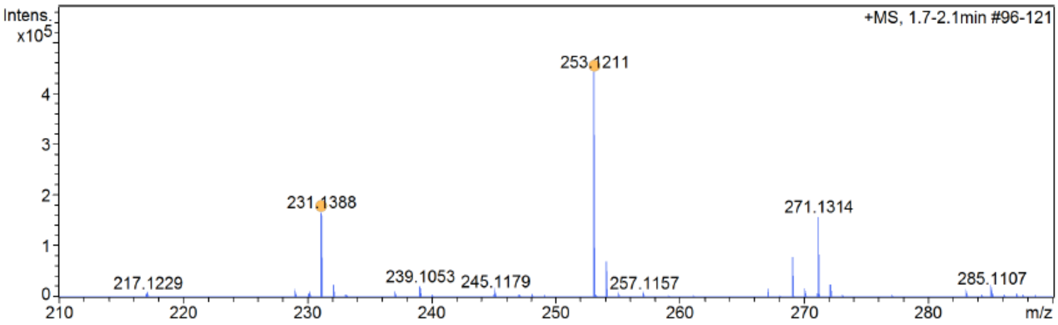


| Meas. m/z | # | Ion Formula | m/z | err [ppm] | mSigma | # mSigma | Score | rdb | e⁻ Conf | N-Rule | Adduct |
|---|---|---|---|---|---|---|---|---|---|---|---|
| 231.1388 | 1 | C15H19O2 | 231.1380 | -3.8 | 5.2 | 1 | 100.00 | 7.0 | even | ok | M+H |
| 253.1211 | 1 | C15H18NaO2 | 253.1199 | -4.7 | 3.0 | 2 | 60.26 | 7.0 | even | ok | M+Na |

## HRMS compound 3aj

# Mass Spectrum SmartFormula Report

**Analysis Info**

| | | | |
|---|---|---|---|
| Analysis Name | \\IMPACT_II-10101\Data\Masa_Exacta\OBG_609.d | Acquisition Date | 3/23/2021 3:57:44 PM |
| Method | MS_MASA_EXACTA.m | Operator | Demo User |
| Sample Name | OBG_609 | Instrument | impact II 1825265.10101 |
| Comment | | | |

**Acquisition Parameter**

| | | | | | |
|---|---|---|---|---|---|
| Source Type | ESI | Ion Polarity | Positive | Set Nebulizer | 2.4 Bar |
| Focus | Active | Set Capillary | 4000 V | Set Dry Heater | 250 °C |
| Scan Begin | 50 m/z | Set End Plate Offset | -500 V | Set Dry Gas | 6.0 l/min |
| Scan End | 1500 m/z | Set Charging Voltage | 2000 V | Set Divert Valve | Source |
| | | Set Corona | 0 nA | Set APCI Heater | 0 °C |

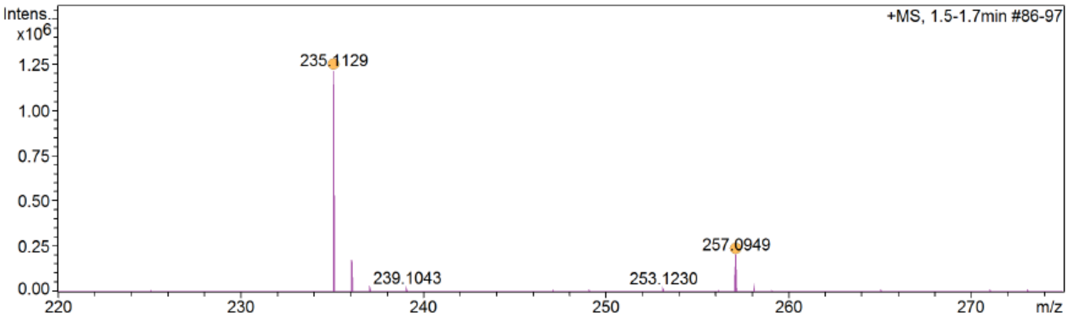


| Meas. m/z | # | Ion Formula | m/z | err [ppm] | mSigma | # mSigma | Score | rdb | e⁻ Conf | N-Rule | Adduct |
|---|---|---|---|---|---|---|---|---|---|---|---|
| 235.1129 | 1 | C14H16FO2 | 235.1129 | 0.0 | 4.0 | 1 | 100.00 | 7.0 | even | ok | M+H |
| 257.0949 | 1 | C14H15FNaO2 | 257.0948 | -0.2 | 13.8 | 1 | 100.00 | 7.0 | even | ok | M+Na |

## HRMS compound 3ak

# Mass Spectrum SmartFormula Report

**Analysis Info**

Acquisition Date 21/12/2020 13:48:03

Analysis Name D:\Data\Masa_Exacta\OBG_465.d
Method MS 50-1000 orgánica.m
Sample Name OBG_465
Comment

Operator Operator
Instrument impact II 1825265.10101

**Acquisition Parameter**

| | | | | | |
|---|---|---|---|---|---|
| Source Type | ESI | Ion Polarity | Positive | Set Nebulizer | 2.5 Bar |
| Focus | Active | Set Capillary | 3500 V | Set Dry Heater | 250 °C |
| Scan Begin | 50 m/z | Set End Plate Offset | -500 V | Set Dry Gas | 6.0 l/min |
| Scan End | 1500 m/z | Set Charging Voltage | 2000 V | Set Divert Valve | Source |
| | | Set Corona | 0 nA | Set APCI Heater | 0 °C |

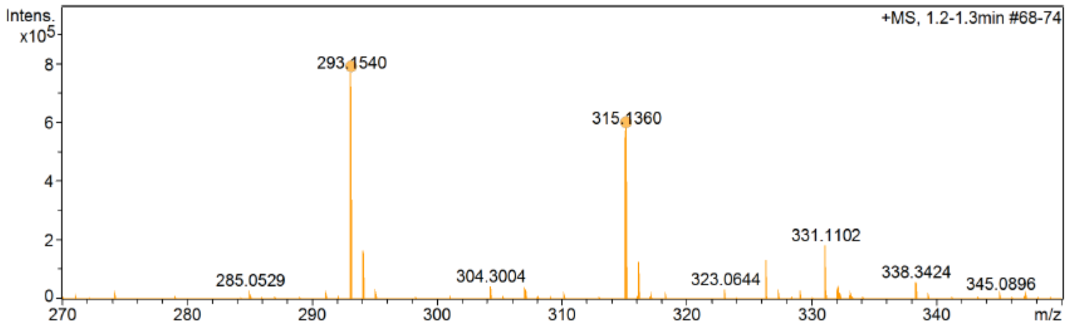


| Meas. m/z | # | Ion Formula | m/z | err [ppm] | mSigma | # mSigma | Score | rdb | e⁻ Conf | N-Rule | Adduct |
|---|---|---|---|---|---|---|---|---|---|---|---|
| 293.1540 | 1 | C20H21O2 | 293.1536 | -1.4 | 1.2 | 1 | 100.00 | 11.0 | even | ok | M+H |
| 315.1360 | 1 | C20H20NaO2 | 315.1356 | -1.5 | 0.8 | 2 | 100.00 | 11.0 | even | ok | M+Na |

## HRMS compound 3al

# Mass Spectrum SmartFormula Report

**Analysis Info**

Acquisition Date 10/08/2020 13:40:36

Analysis Name D:\Data\OBG_430_BA1_01_5446.d
Method FantasmaMASAEXACTA_POS.m
Sample Name OBG_430
Comment

Operator Demo User
Instrument impact II 1825265.10101

**Acquisition Parameter**

| | | | | | |
|---|---|---|---|---|---|
| Source Type | ESI | Ion Polarity | Positive | Set Nebulizer | 2.4 Bar |
| Focus | Active | Set Capillary | 3500 V | Set Dry Heater | 250 °C |
| Scan Begin | 50 m/z | Set End Plate Offset | -500 V | Set Dry Gas | 6.0 l/min |
| Scan End | 1500 m/z | Set Charging Voltage | 2000 V | Set Divert Valve | Source |
| | | Set Corona | 0 nA | Set APCI Heater | 0 °C |

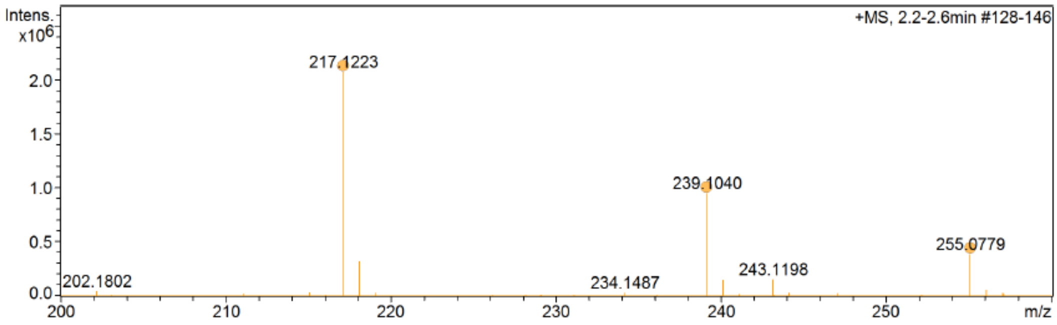


| Meas. m/z | # | Ion Formula | m/z | err [ppm] | mSigma | # mSigma | Score | rdb | e⁻ Conf | N-Rule | Adduct |
|---|---|---|---|---|---|---|---|---|---|---|---|
| 217.1223 | 1 | C14H17O2 | 217.1223 | -0.1 | 1.2 | 1 | 100.00 | 7.0 | even | ok | M+H |
| 239.1040 | 1 | C14H16NaO2 | 239.1043 | 0.9 | 1.7 | 1 | 100.00 | 7.0 | even | ok | M+Na |
| 255.0779 | 1 | C14H16KO2 | 255.0782 | 1.1 | 3.8 | 1 | 100.00 | 7.0 | even | ok | M+K |

## HRMS compound 3ba

# Mass Spectrum SmartFormula Report

**Analysis Info**

| | | | |
|---|---|---|---|
| Analysis Name | D:\Data\KY_351_BA1_01_5457.d | Acquisition Date | 11/08/2020 13:17:32 |
| Method | FantasmaMASAEXACTA_POS.m | Operator | Demo User |
| Sample Name | KY_351 | Instrument | impact II 1825265.10101 |
| Comment | | | |

**Acquisition Parameter**

| | | | | | |
|---|---|---|---|---|---|
| Source Type | ESI | Ion Polarity | Positive | Set Nebulizer | 2.4 Bar |
| Focus | Active | Set Capillary | 3500 V | Set Dry Heater | 250 °C |
| Scan Begin | 50 m/z | Set End Plate Offset | -500 V | Set Dry Gas | 6.0 l/min |
| Scan End | 1500 m/z | Set Charging Voltage | 2000 V | Set Divert Valve | Source |
| | | Set Corona | 0 nA | Set APCI Heater | 0 °C |

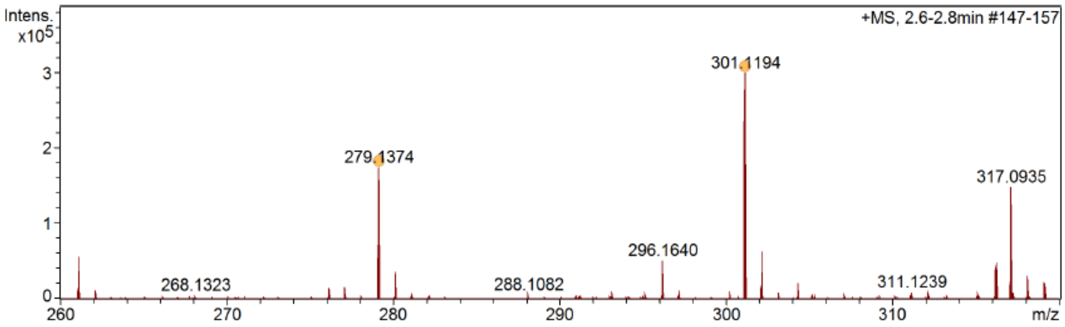


| Meas. m/z | # | Ion Formula | m/z | err [ppm] | mSigma | # mSigma | Score | rdb | e⁻ Conf | N-Rule | Adduct |
|---|---|---|---|---|---|---|---|---|---|---|---|
| 279.1374 | 1 | C19H19O2 | 279.1380 | 1.8 | 1.8 | 1 | 100.00 | 11.0 | even | ok | M+H |
| 301.1194 | 1 | C19H18NaO2 | 301.1199 | 1.7 | 2.9 | 1 | 100.00 | 11.0 | even | ok | M+Na |

## HRMS compound 3bb

# Mass Spectrum SmartFormula Report

**Analysis Info**

| | | | |
|---|---|---|---|
| Analysis Name | D:\Data\KY_419_BA1_01_5456.d | Acquisition Date | 11/08/2020 13:10:49 |
| Method | FantasmaMASAEXACTA_POS.m | Operator | Demo User |
| Sample Name | KY_419 | Instrument | impact II 1825265.10101 |
| Comment | | | |

**Acquisition Parameter**

| | | | | | |
|---|---|---|---|---|---|
| Source Type | ESI | Ion Polarity | Positive | Set Nebulizer | 2.4 Bar |
| Focus | Active | Set Capillary | 3500 V | Set Dry Heater | 250 °C |
| Scan Begin | 50 m/z | Set End Plate Offset | -500 V | Set Dry Gas | 6.0 l/min |
| Scan End | 1500 m/z | Set Charging Voltage | 2000 V | Set Divert Valve | Source |
| | | Set Corona | 0 nA | Set APCI Heater | 0 °C |

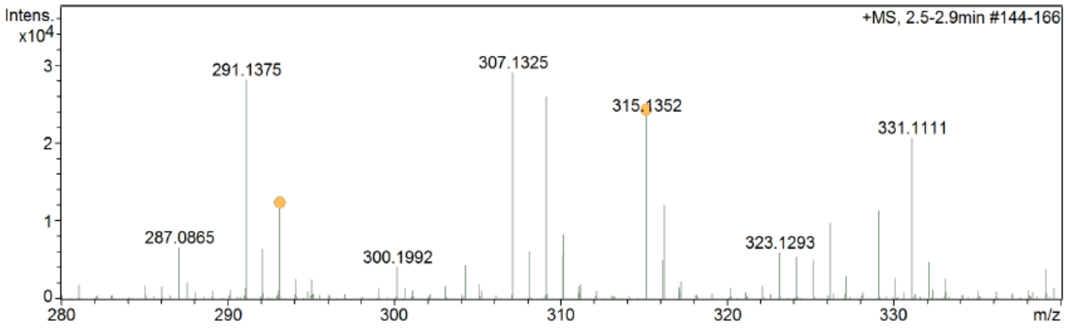


| Meas. m/z | # | Ion Formula | m/z | err [ppm] | mSigma | # mSigma | Score | rdb | e⁻ Conf | N-Rule | Adduct |
|---|---|---|---|---|---|---|---|---|---|---|---|
| 293.1528 | 1 | C20H21O2 | 293.1536 | 2.7 | 6.4 | 1 | 100.00 | 11.0 | even | ok | M+H |
| 315.1352 | 1 | C20H20NaO2 | 315.1356 | 1.2 | 9.1 | 1 | 100.00 | 11.0 | even | ok | M+Na |

## HRMS compound 3bf

# Mass Spectrum SmartFormula Report

**Analysis Info**

| | | | |
|---|---|---|---|
| Analysis Name | D:\Data\KY_472_BA1_01_5451.d | Acquisition Date | 11/08/2020 12:31:10 |
| Method | FantasmaMASAEXACTA_POS.m | Operator | Demo User |
| Sample Name | KY_472 | Instrument | impact II 1825265.10101 |
| Comment | | | |

**Acquisition Parameter**

| | | | | | |
|---|---|---|---|---|---|
| Source Type | ESI | Ion Polarity | Positive | Set Nebulizer | 2.4 Bar |
| Focus | Active | Set Capillary | 3500 V | Set Dry Heater | 250 °C |
| Scan Begin | 50 m/z | Set End Plate Offset | -500 V | Set Dry Gas | 6.0 l/min |
| Scan End | 1500 m/z | Set Charging Voltage | 2000 V | Set Divert Valve | Source |
| | | Set Corona | 0 nA | Set APCI Heater | 0 °C |

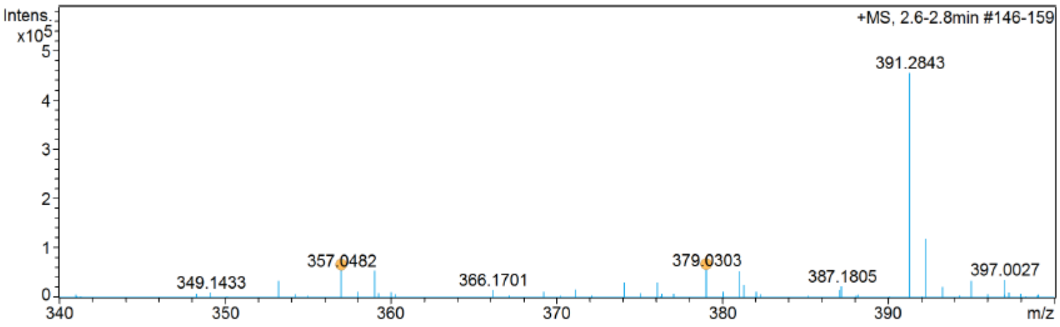


| Meas. m/z | # | Ion Formula | m/z | err [ppm] | mSigma | # mSigma | Score | rdb | e⁻ Conf | N-Rule | Adduct |
|---|---|---|---|---|---|---|---|---|---|---|---|
| 357.0482 | 1 | C19H18BrO2 | 357.0485 | 0.8 | 3.9 | 1 | 100.00 | 11.0 | even | ok | M+H |
| 379.0303 | 1 | C19H17BrNaO2 | 379.0304 | 0.2 | 15.0 | 1 | 100.00 | 11.0 | even | ok | M+Na |

## HRMS compound 3ca

# Mass Spectrum SmartFormula Report

**Analysis Info**

| | | | |
|---|---|---|---|
| Analysis Name | D:\Data\KY_346_BA1_01_5455.d | Acquisition Date | 11/08/2020 13:03:30 |
| Method | FantasmaMASAEXACTA_POS.m | Operator | Demo User |
| Sample Name | KY_346 | Instrument | impact II 1825265.10101 |
| Comment | | | |

**Acquisition Parameter**

| | | | | | |
|---|---|---|---|---|---|
| Source Type | ESI | Ion Polarity | Positive | Set Nebulizer | 2.4 Bar |
| Focus | Active | Set Capillary | 3500 V | Set Dry Heater | 250 °C |
| Scan Begin | 50 m/z | Set End Plate Offset | -500 V | Set Dry Gas | 6.0 l/min |
| Scan End | 1500 m/z | Set Charging Voltage | 2000 V | Set Divert Valve | Source |
| | | Set Corona | 0 nA | Set APCI Heater | 0 °C |

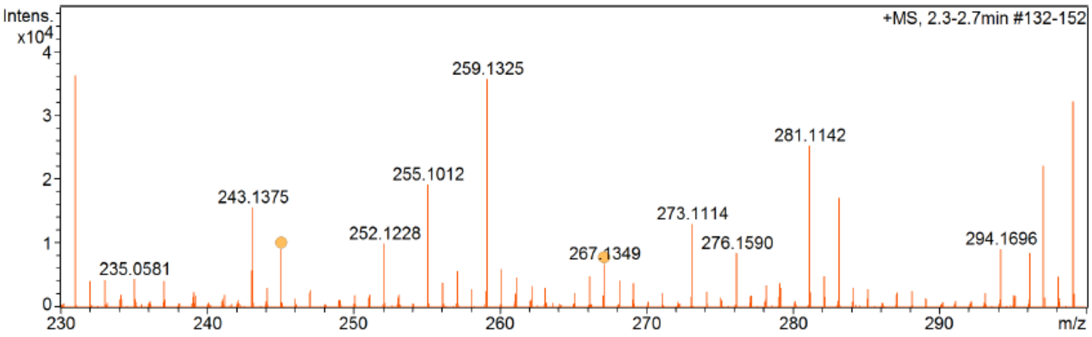


| Meas. m/z | # | Ion Formula | m/z | err [ppm] | mSigma | # mSigma | Score | rdb | e⁻ Conf | N-Rule | Adduct |
|---|---|---|---|---|---|---|---|---|---|---|---|
| 245.0782 | 1 | C17H10NO | 244.0757 | 3.2 | 703.4 | 1 | 100.00 | 14.0 | even | ok | M+H |
| 267.1349 | 1 | C16H20NaO2 | 267.1356 | 2.4 | 107.4 | 1 | 100.00 | 7.0 | even | ok | M+Na |

## HRMS compound 3da

# Mass Spectrum SmartFormula Report

**Analysis Info**

| | | | |
|---|---|---|---|
| Analysis Name | \\IMPACT_II-10101\Data\Masa_Exacta\OBG_621.d | Acquisition Date | 3/23/2021 4:02:50 PM |
| Method | MS_MASA_EXACTA.m | Operator | Demo User |
| Sample Name | OBG_621 | Instrument | impact II 1825265.10101 |
| Comment | | | |

**Acquisition Parameter**

| | | | | | |
|---|---|---|---|---|---|
| Source Type | ESI | Ion Polarity | Positive | Set Nebulizer | 2.4 Bar |
| Focus | Active | Set Capillary | 4000 V | Set Dry Heater | 250 °C |
| Scan Begin | 50 m/z | Set End Plate Offset | -500 V | Set Dry Gas | 6.0 l/min |
| Scan End | 1500 m/z | Set Charging Voltage | 2000 V | Set Divert Valve | Source |
| | | Set Corona | 0 nA | Set APCI Heater | 0 °C |

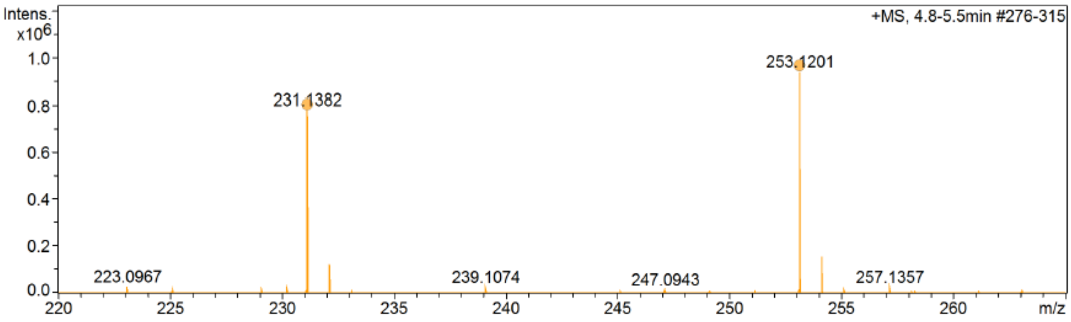


| Meas. m/z | # | Ion Formula | m/z | err [ppm] | mSigma | # mSigma | Score | rdb | e⁻ Conf | N-Rule | Adduct |
|---|---|---|---|---|---|---|---|---|---|---|---|
| 231.1382 | 1 | C15H19O2 | 231.1380 | -0.9 | 3.1 | 1 | 100.00 | 7.0 | even | ok | M+H |
| 253.1201 | 1 | C15H18NaO2 | 253.1199 | -0.6 | 1.6 | 2 | 100.00 | 7.0 | even | ok | M+Na |

## HRMS compound 3dd

# Mass Spectrum SmartFormula Report

**Analysis Info**

| | | | |
|---|---|---|---|
| Analysis Name | \\IMPACT_II-10101\Data\Masa_Exacta\OBG_622.d | Acquisition Date | 3/23/2021 4:33:52 PM |
| Method | MS_MASA_EXACTA.m | Operator | Demo User |
| Sample Name | OBG_622 | Instrument | impact II 1825265.10101 |
| Comment | | | |

**Acquisition Parameter**

| | | | | | |
|---|---|---|---|---|---|
| Source Type | ESI | Ion Polarity | Positive | Set Nebulizer | 2.4 Bar |
| Focus | Active | Set Capillary | 4000 V | Set Dry Heater | 250 °C |
| Scan Begin | 50 m/z | Set End Plate Offset | -500 V | Set Dry Gas | 6.0 l/min |
| Scan End | 1500 m/z | Set Charging Voltage | 2000 V | Set Divert Valve | Source |
| | | Set Corona | 0 nA | Set APCI Heater | 0 °C |

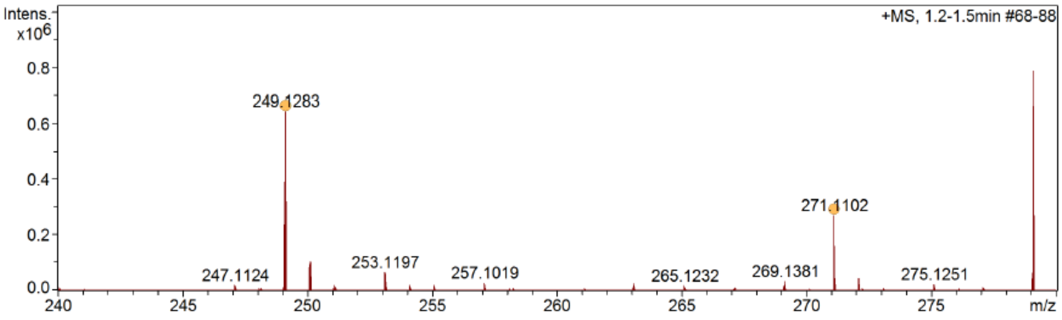


| Meas. m/z | # | Ion Formula | m/z | err [ppm] | mSigma | # mSigma | Score | rdb | e⁻ Conf | N-Rule | Adduct |
|---|---|---|---|---|---|---|---|---|---|---|---|
| 249.1283 | 1 | C15H18FO2 | 249.1285 | 1.0 | 4.4 | 1 | 100.00 | 7.0 | even | ok | M+H |
| 271.1102 | 1 | C15H17FNaO2 | 271.1105 | 1.0 | 9.9 | 2 | 100.00 | 7.0 | even | ok | M+Na |

**HRMS compound 3ea**

## Mass Spectrum SmartFormula Report

**Analysis Info**

| | | | |
|---|---|---|---|
| Analysis Name | \\IMPACT_II-10101\Data\Masa_Exacta\OBG_594.d | Acquisition Date | 3/23/2021 4:13:48 PM |
| Method | MS_MASA_EXACTA.m | Operator | Demo User |
| Sample Name | OBG_594 | Instrument | impact II 1825265.10101 |
| Comment | | | |

**Acquisition Parameter**

| | | | | | |
|---|---|---|---|---|---|
| Source Type | ESI | Ion Polarity | Positive | Set Nebulizer | 2.4 Bar |
| Focus | Active | Set Capillary | 4000 V | Set Dry Heater | 250 °C |
| Scan Begin | 50 m/z | Set End Plate Offset | -500 V | Set Dry Gas | 6.0 l/min |
| Scan End | 1500 m/z | Set Charging Voltage | 2000 V | Set Divert Valve | Source |
| | | Set Corona | 0 nA | Set APCI Heater | 0 °C |

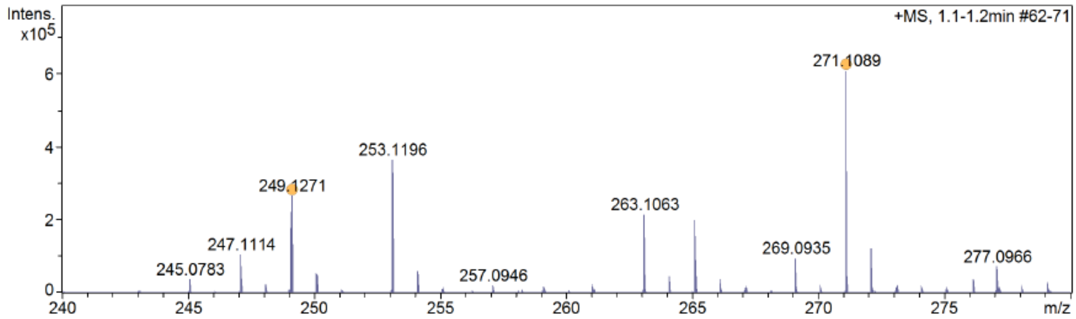


| Meas. m/z | # | Ion Formula | m/z | err [ppm] | mSigma | # mSigma | Score | rdb | e⁻ Conf | N-Rule | Adduct |
|---|---|---|---|---|---|---|---|---|---|---|---|
| 249.1271 | 1 | C18H17O | 249.1274 | 1.2 | 1.0 | 1 | 100.00 | 11.0 | even | ok | M+H |
| 271.1089 | 1 | C18H16NaO | 271.1093 | 1.6 | 3.0 | 1 | 100.00 | 11.0 | even | ok | M+Na |

**HRMS compound 3fa**

## Mass Spectrum SmartFormula Report

**Analysis Info**

| | | | |
|---|---|---|---|
| Analysis Name | \\IMPACT_II-10101\Data\Masa_Exacta\OBG_560.d | Acquisition Date | 3/23/2021 4:17:43 PM |
| Method | MS_MASA_EXACTA.m | Operator | Demo User |
| Sample Name | OBG_560 | Instrument | impact II 1825265.10101 |
| Comment | | | |

**Acquisition Parameter**

| | | | | | |
|---|---|---|---|---|---|
| Source Type | ESI | Ion Polarity | Positive | Set Nebulizer | 2.4 Bar |
| Focus | Active | Set Capillary | 4000 V | Set Dry Heater | 250 °C |
| Scan Begin | 50 m/z | Set End Plate Offset | -500 V | Set Dry Gas | 6.0 l/min |
| Scan End | 1500 m/z | Set Charging Voltage | 2000 V | Set Divert Valve | Source |
| | | Set Corona | 0 nA | Set APCI Heater | 0 °C |

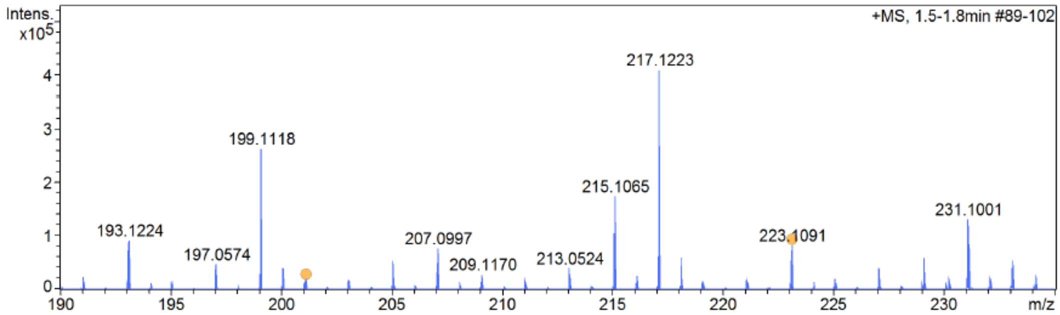


| Meas. m/z | # | Ion Formula | m/z | err [ppm] | mSigma | # mSigma | Score | rdb | e⁻ Conf | N-Rule | Adduct |
|---|---|---|---|---|---|---|---|---|---|---|---|
| 201.1267 | 1 | C14H17O | 201.1274 | 3.3 | 16.7 | 1 | 100.00 | 7.0 | even | ok | M+H |
| 223.1091 | 1 | C14H16NaO | 223.1093 | 1.1 | 81.7 | 1 | 100.00 | 7.0 | even | ok | M+Na |

## HRMS compound 4ga

# Mass Spectrum SmartFormula Report

**Analysis Info**

| | | | |
|---|---|---|---|
| Analysis Name | \\IMPACT_II-10101\Data\Masa_Exacta\OBG_557.d | Acquisition Date | 3/23/2021 4:21:11 PM |
| Method | MS_MASA_EXACTA.m | Operator | Demo User |
| Sample Name | OBG_557 | Instrument | impact II 1825265.10101 |
| Comment | | | |

**Acquisition Parameter**

| | | | | | |
|---|---|---|---|---|---|
| Source Type | ESI | Ion Polarity | Positive | Set Nebulizer | 2.4 Bar |
| Focus | Active | Set Capillary | 4000 V | Set Dry Heater | 250 °C |
| Scan Begin | 50 m/z | Set End Plate Offset | -500 V | Set Dry Gas | 6.0 l/min |
| Scan End | 1500 m/z | Set Charging Voltage | 2000 V | Set Divert Valve | Source |
| | | Set Corona | 0 nA | Set APCI Heater | 0 °C |

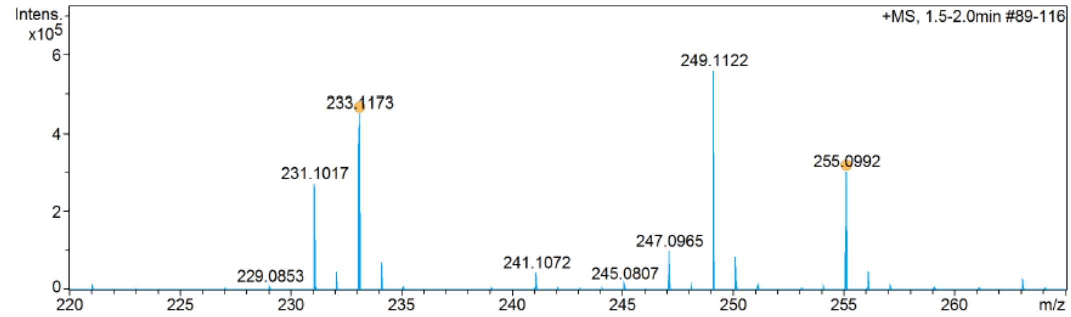


| Meas. m/z | # | Ion Formula | m/z | err [ppm] | mSigma | # mSigma | Score | rdb | e⁻ Conf | N-Rule | Adduct |
|---|---|---|---|---|---|---|---|---|---|---|---|
| 233.1173 | 1 | C14H17O3 | 233.1172 | -0.3 | 1.0 | 2 | 100.00 | 7.0 | even | ok | M+H |
| 255.0992 | 1 | C14H16NaO3 | 255.0992 | -0.1 | 9.4 | 2 | 100.00 | 7.0 | even | ok | M+Na |

## HRMS compound 4gd

# Mass Spectrum SmartFormula Report

**Analysis Info**

| | | | |
|---|---|---|---|
| Analysis Name | \\IMPACT_II-10101\Data\Masa_Exacta\OBG_545.d | Acquisition Date | 3/23/2021 4:26:06 PM |
| Method | MS_MASA_EXACTA.m | Operator | Demo User |
| Sample Name | OBG_545 | Instrument | impact II 1825265.10101 |
| Comment | | | |

**Acquisition Parameter**

| | | | | | |
|---|---|---|---|---|---|
| Source Type | ESI | Ion Polarity | Positive | Set Nebulizer | 2.4 Bar |
| Focus | Active | Set Capillary | 4000 V | Set Dry Heater | 250 °C |
| Scan Begin | 50 m/z | Set End Plate Offset | -500 V | Set Dry Gas | 6.0 l/min |
| Scan End | 1500 m/z | Set Charging Voltage | 2000 V | Set Divert Valve | Source |
| | | Set Corona | 0 nA | Set APCI Heater | 0 °C |

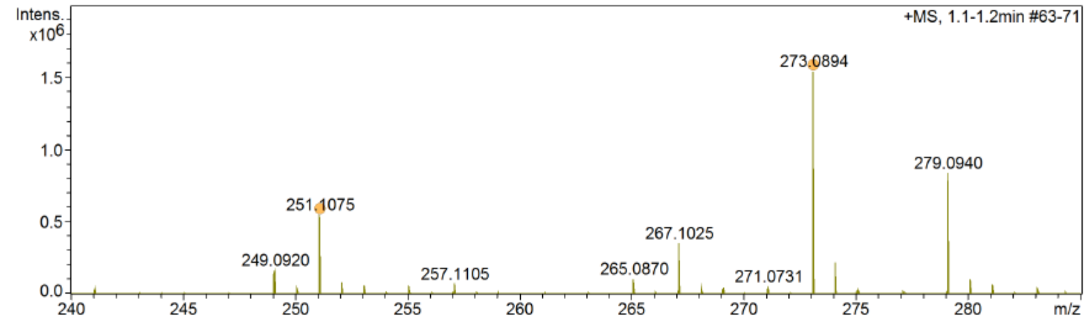


| Meas. m/z | # | Ion Formula | m/z | err [ppm] | mSigma | # mSigma | Score | rdb | e⁻ Conf | N-Rule | Adduct |
|---|---|---|---|---|---|---|---|---|---|---|---|
| 251.1075 | 1 | C14H16FO3 | 251.1078 | 1.3 | 51.8 | 1 | 100.00 | 7.0 | even | ok | M+H |
| 273.0894 | 1 | C14H15FNaO3 | 273.0897 | 1.1 | 4.8 | 1 | 100.00 | 7.0 | even | ok | M+Na |

## HRMS compound 3aa-D

# Mass Spectrum SmartFormula Report

**Analysis Info**

| | | | |
|---|---|---|---|
| Analysis Name | \\IMPACT_II-10101\Data\Masa_Exacta\OBG_525.d | Acquisition Date | 3/23/2021 3:36:33 PM |
| Method | MS_MASA_EXACTA.m | Operator | Demo User |
| Sample Name | OBG_525 | Instrument | impact II 1825265.10101 |
| Comment | | | |

**Acquisition Parameter**

| | | | | | |
|---|---|---|---|---|---|
| Source Type | ESI | Ion Polarity | Positive | Set Nebulizer | 2.4 Bar |
| Focus | Active | Set Capillary | 4000 V | Set Dry Heater | 250 °C |
| Scan Begin | 50 m/z | Set End Plate Offset | -500 V | Set Dry Gas | 6.0 l/min |
| Scan End | 1500 m/z | Set Charging Voltage | 2000 V | Set Divert Valve | Source |
| | | Set Corona | 0 nA | Set APCI Heater | 0 °C |

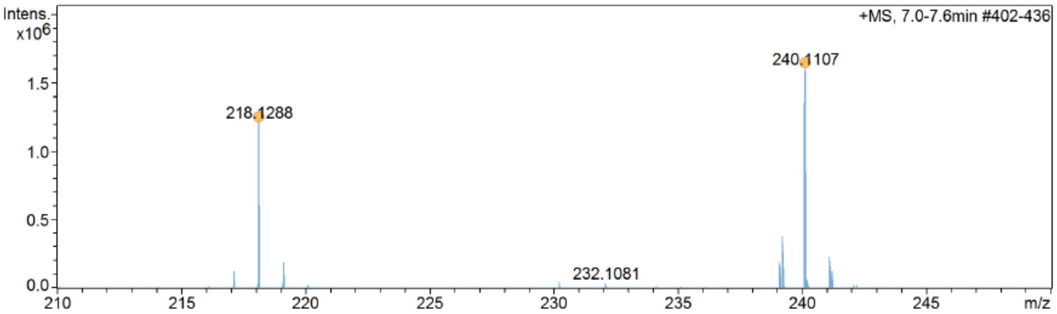


| Meas. m/z | # | Ion Formula | m/z | err [ppm] | mSigma | # mSigma | Score | rdb | e⁻ Conf | N-Rule | Adduct |
|---|---|---|---|---|---|---|---|---|---|---|---|
| 218.1288 | 1 | C14H16DO2 | 218.1286 | -0.8 | 3.5 | 1 | 100.00 | 7.0 | even | ok | M+H |
| 240.1107 | 1 | C14H15DNaO2 | 240.1105 | -0.6 | 3.2 | 1 | 97.69 | 7.0 | even | ok | M+Na |

## HRMS compound 3ca-D

# Mass Spectrum SmartFormula Report

**Analysis Info**

| | | | |
|---|---|---|---|
| Analysis Name | \\IMPACT_II-10101\Data\Masa_Exacta\OBG_526_.d | Acquisition Date | 3/23/2021 3:30:17 PM |
| Method | MS_MASA_EXACTA.m | Operator | Demo User |
| Sample Name | OBG_526_ | Instrument | impact II 1825265.10101 |
| Comment | | | |

**Acquisition Parameter**

| | | | | | |
|---|---|---|---|---|---|
| Source Type | ESI | Ion Polarity | Positive | Set Nebulizer | 2.4 Bar |
| Focus | Active | Set Capillary | 4000 V | Set Dry Heater | 250 °C |
| Scan Begin | 50 m/z | Set End Plate Offset | -500 V | Set Dry Gas | 6.0 l/min |
| Scan End | 1500 m/z | Set Charging Voltage | 2000 V | Set Divert Valve | Source |
| | | Set Corona | 0 nA | Set APCI Heater | 0 °C |

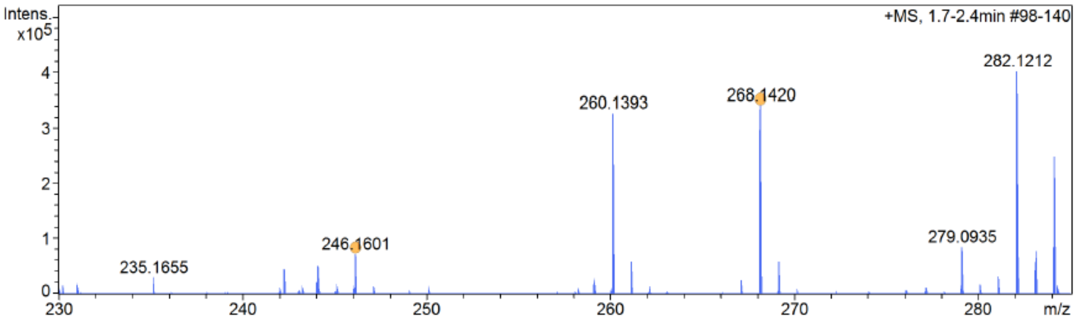


| Meas. m/z | # | Ion Formula | m/z | err [ppm] | mSigma | # mSigma | Score | rdb | e⁻ Conf | N-Rule | Adduct |
|---|---|---|---|---|---|---|---|---|---|---|---|
| 246.1601 | 1 | C16H20DO2 | 246.1599 | -0.8 | 6.6 | 1 | 100.00 | 7.0 | even | ok | M+H |
| 268.1420 | 1 | C16H19DNaO2 | 268.1418 | -0.6 | 0.6 | 1 | 100.00 | 7.0 | even | ok | M+Na |

## HRMS compound 5

# Mass Spectrum SmartFormula Report

**Analysis Info**

| | | | |
|---|---|---|---|
| Analysis Name | \\IMPACT_II-10101\Data\Masa_Exacta\OBG_503.d | Acquisition Date | 3/23/2021 4:29:42 PM |
| Method | MS_MASA_EXACTA.m | Operator | Demo User |
| Sample Name | OBG_503 | Instrument | impact II 1825265.10101 |
| Comment | | | |

**Acquisition Parameter**

| | | | | | |
|---|---|---|---|---|---|
| Source Type | ESI | Ion Polarity | Positive | Set Nebulizer | 2.4 Bar |
| Focus | Active | Set Capillary | 4000 V | Set Dry Heater | 250 °C |
| Scan Begin | 50 m/z | Set End Plate Offset | -500 V | Set Dry Gas | 6.0 l/min |
| Scan End | 1500 m/z | Set Charging Voltage | 2000 V | Set Divert Valve | Source |
| | | Set Corona | 0 nA | Set APCI Heater | 0 °C |

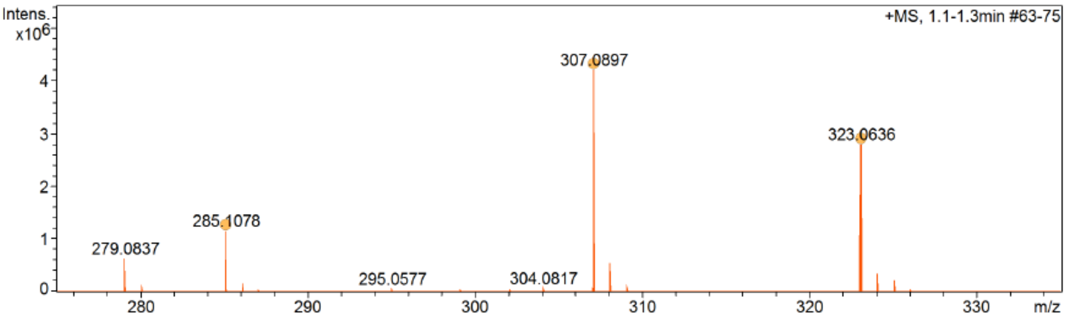


| Meas. m/z | # | Ion Formula | m/z | err [ppm] | mSigma | # mSigma | Score | rdb | e⁻ Conf | N-Rule | Adduct |
|---|---|---|---|---|---|---|---|---|---|---|---|
| 285.1078 | 1 | C12H17N2O6 | 285.1081 | 0.9 | 4.3 | 2 | 100.00 | 6.0 | even | ok | M+H |
| 307.0897 | 1 | C12H16N2NaO6 | 307.0901 | 1.3 | 6.5 | 2 | 100.00 | 6.0 | even | ok | M+Na |
| 323.0636 | 1 | C12H16KN2O6 | 323.0640 | 1.4 | 10.6 | 2 | 100.00 | 6.0 | even | ok | M+K |

## HRMS compound 7aa

# Mass Spectrum SmartFormula Report

**Analysis Info**

| | | | |
|---|---|---|---|
| Analysis Name | D:\Data\Masa_Exacta\OBG_512.d | Acquisition Date | 21/12/2020 14:05:06 |
| Method | MS 50-1000 orgánica.m | Operator | Operator |
| Sample Name | OBG_512 | Instrument | impact II 1825265.10101 |
| Comment | | | |

**Acquisition Parameter**

| | | | | | |
|---|---|---|---|---|---|
| Source Type | ESI | Ion Polarity | Positive | Set Nebulizer | 2.5 Bar |
| Focus | Active | Set Capillary | 3500 V | Set Dry Heater | 250 °C |
| Scan Begin | 50 m/z | Set End Plate Offset | -500 V | Set Dry Gas | 6.0 l/min |
| Scan End | 1500 m/z | Set Charging Voltage | 2000 V | Set Divert Valve | Source |
| | | Set Corona | 0 nA | Set APCI Heater | 0 °C |

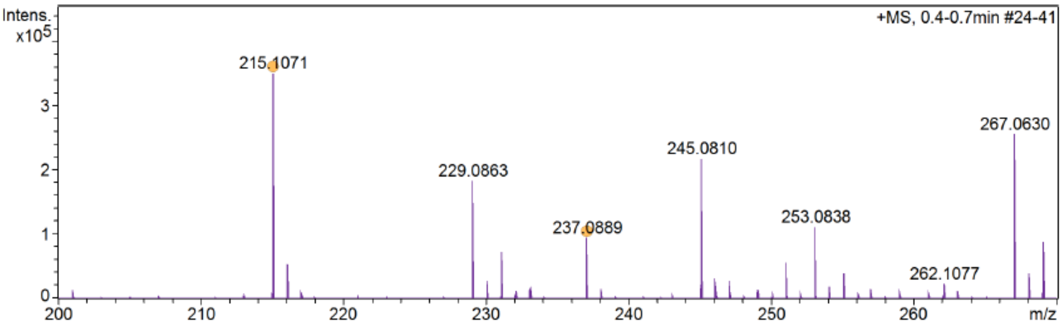


| Meas. m/z | # | Ion Formula | m/z | err [ppm] | mSigma | # mSigma | Score | rdb | e⁻ Conf | N-Rule | Adduct |
|---|---|---|---|---|---|---|---|---|---|---|---|
| 215.1071 | 1 | C14H15O2 | 215.1067 | -1.8 | 0.5 | 1 | 100.00 | 8.0 | even | ok | M+H |
| 237.0889 | 1 | C14H14NaO2 | 237.0886 | -1.3 | 4.8 | 2 | 100.00 | 8.0 | even | ok | M+Na |

## HRMS compound 7ba

# Mass Spectrum SmartFormula Report

**Analysis Info**

Acquisition Date 11/08/2020 12:35:12

Analysis Name D:\Data\KY_447.d
Method MS_MASA_EXACTA.m
Sample Name KY_447
Comment

Operator Operator
Instrument impact II 1825265.10101

**Acquisition Parameter**

| | | | | | |
|---|---|---|---|---|---|
| Source Type | ESI | Ion Polarity | Positive | Set Nebulizer | 2.4 Bar |
| Focus | Active | Set Capillary | 4000 V | Set Dry Heater | 250 °C |
| Scan Begin | 50 m/z | Set End Plate Offset | -500 V | Set Dry Gas | 6.0 l/min |
| Scan End | 1500 m/z | Set Charging Voltage | 2000 V | Set Divert Valve | Source |
| | | Set Corona | 0 nA | Set APCI Heater | 0 °C |

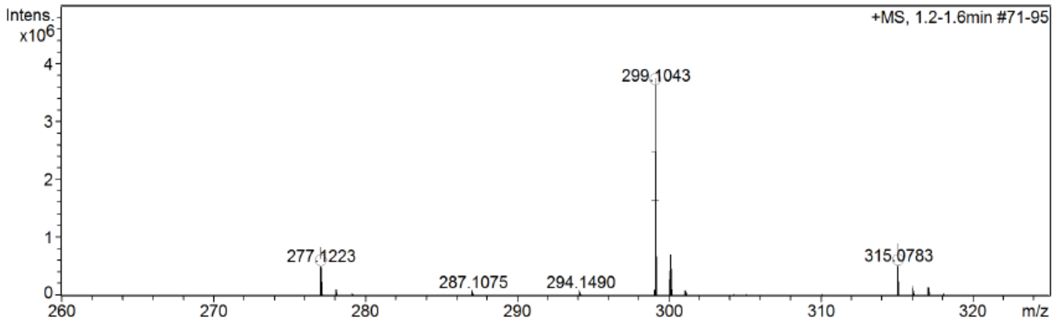


| Meas. m/z | # | Ion Formula | m/z | err [ppm] | mSigma | # mSigma | Score | rdb | e⁻ Conf | N-Rule | Adduct |
|---|---|---|---|---|---|---|---|---|---|---|---|
| 277.1223 | 1 | C19H17O2 | 277.1223 | -0.1 | 5.1 | 2 | 100.00 | 12.0 | even | ok | M+H |
| 299.1043 | 1 | C19H16NaO2 | 299.1043 | -0.1 | 6.2 | 2 | 100.00 | 12.0 | even | ok | M+Na |
| 315.0783 | 1 | C19H16KO2 | 315.0782 | -0.2 | 9.8 | 2 | 100.00 | 12.0 | even | ok | M+K |

## HRMS compound 7ab

# Mass Spectrum SmartFormula Report

**Analysis Info**

Acquisition Date 21/12/2020 13:43:34

Analysis Name D:\Data\Masa_Exacta\OBG_470.d
Method MS 50-1000 orgánica.m
Sample Name OBG_470
Comment

Operator Operator
Instrument impact II 1825265.10101

**Acquisition Parameter**

| | | | | | |
|---|---|---|---|---|---|
| Source Type | ESI | Ion Polarity | Positive | Set Nebulizer | 2.5 Bar |
| Focus | Active | Set Capillary | 3500 V | Set Dry Heater | 250 °C |
| Scan Begin | 50 m/z | Set End Plate Offset | -500 V | Set Dry Gas | 6.0 l/min |
| Scan End | 1500 m/z | Set Charging Voltage | 2000 V | Set Divert Valve | Source |
| | | Set Corona | 0 nA | Set APCI Heater | 0 °C |

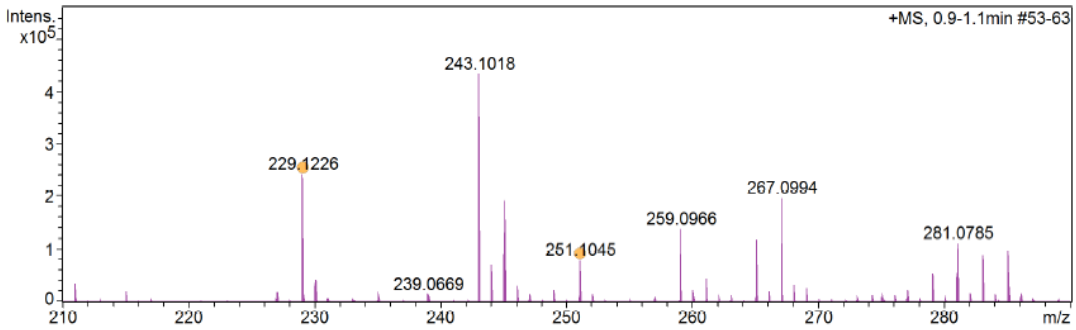


| Meas. m/z | # | Ion Formula | m/z | err [ppm] | mSigma | # mSigma | Score | rdb | e⁻ Conf | N-Rule | Adduct |
|---|---|---|---|---|---|---|---|---|---|---|---|
| 229.1226 | 1 | C15H17O2 | 229.1223 | -1.3 | 8.2 | 1 | 100.00 | 8.0 | even | ok | M+H |
| 251.1045 | 1 | C15H16NaO2 | 251.1043 | -1.1 | 3.7 | 2 | 100.00 | 8.0 | even | ok | M+Na |

## HRMS compound 7bb

# Mass Spectrum SmartFormula Report

**Analysis Info**

| | | | |
|---|---|---|---|
| Analysis Name | \\IMPACT_II-10101\Data\Masa_Exacta\OBG_608_.d | Acquisition Date | 3/23/2021 1:38:53 PM |
| Method | MS_MASA_EXACTA.m | Operator | Demo User |
| Sample Name | OBG_608_ | Instrument | impact II 1825265.10101 |
| Comment | | | |

**Acquisition Parameter**

| | | | | | |
|---|---|---|---|---|---|
| Source Type | ESI | Ion Polarity | Positive | Set Nebulizer | 2.4 Bar |
| Focus | Active | Set Capillary | 4000 V | Set Dry Heater | 250 °C |
| Scan Begin | 50 m/z | Set End Plate Offset | -500 V | Set Dry Gas | 6.0 l/min |
| Scan End | 1500 m/z | Set Charging Voltage | 2000 V | Set Divert Valve | Source |
| | | Set Corona | 0 nA | Set APCI Heater | 0 °C |

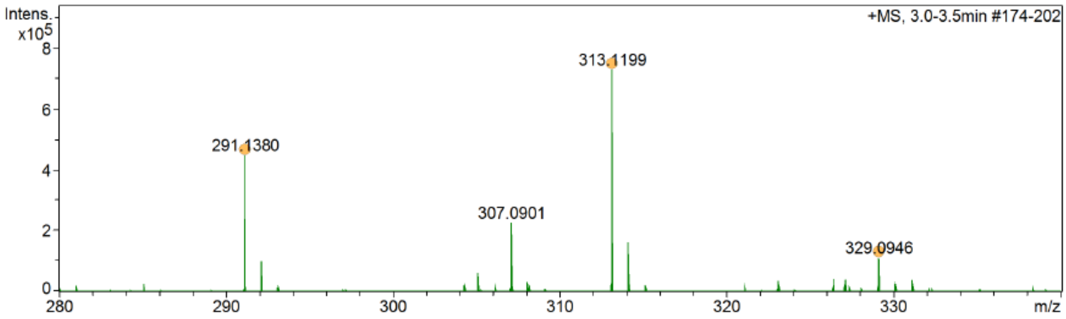


| Meas. m/z | # | Ion Formula | m/z | err [ppm] | mSigma | # mSigma | Score | rdb | e⁻ Conf | N-Rule | Adduct |
|---|---|---|---|---|---|---|---|---|---|---|---|
| 291.1380 | 1 | C20H19O2 | 291.1380 | -0.1 | 3.5 | 1 | 100.00 | 12.0 | even | ok | M+H |
| 313.1199 | 1 | C20H18NaO2 | 313.1199 | -0.1 | 5.1 | 2 | 100.00 | 12.0 | even | ok | M+Na |
| 329.0946 | 1 | C20H18KO2 | 329.0938 | -2.4 | 7.2 | 1 | 100.00 | 12.0 | even | ok | M+K |

## HRMS compound 7ac

# Mass Spectrum SmartFormula Report

**Analysis Info**

| | | | |
|---|---|---|---|
| Analysis Name | D:\Data\Masa_Exacta\OBG_473.d | Acquisition Date | 21/12/2020 13:55:03 |
| Method | MS 50-1000 orgánica.m | Operator | Operator |
| Sample Name | OBG_473 | Instrument | impact II 1825265.10101 |
| Comment | | | |

**Acquisition Parameter**

| | | | | | |
|---|---|---|---|---|---|
| Source Type | ESI | Ion Polarity | Positive | Set Nebulizer | 2.5 Bar |
| Focus | Active | Set Capillary | 3500 V | Set Dry Heater | 250 °C |
| Scan Begin | 50 m/z | Set End Plate Offset | -500 V | Set Dry Gas | 6.0 l/min |
| Scan End | 1500 m/z | Set Charging Voltage | 2000 V | Set Divert Valve | Source |
| | | Set Corona | 0 nA | Set APCI Heater | 0 °C |

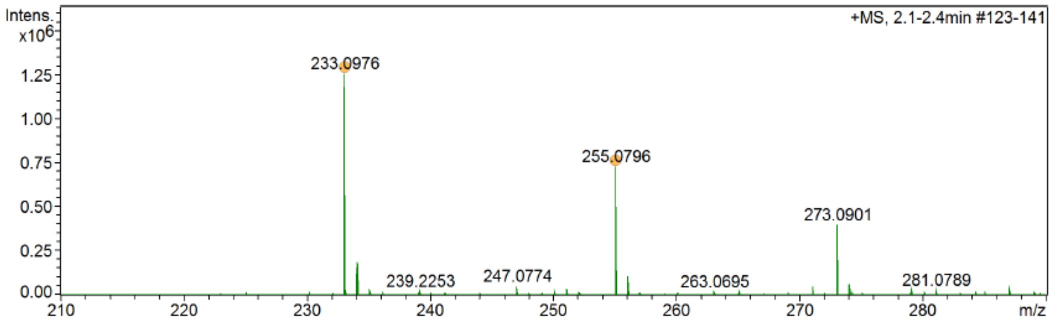


| Meas. m/z | # | Ion Formula | m/z | err [ppm] | mSigma | # mSigma | Score | rdb | e⁻ Conf | N-Rule | Adduct |
|---|---|---|---|---|---|---|---|---|---|---|---|
| 233.0976 | 1 | C14H14FO2 | 233.0972 | -1.7 | 1.7 | 1 | 100.00 | 8.0 | even | ok | M+H |
| 255.0796 | 1 | C14H13FNaO2 | 255.0792 | -1.5 | 2.2 | 1 | 100.00 | 8.0 | even | ok | M+Na |

## HRMS compound 7ad

# Mass Spectrum SmartFormula Report

**Analysis Info**

Acquisition Date 21/12/2020 13:36:21

Analysis Name D:\Data\Masa_Exacta\OBG_469.d
Method MS 50-1000 orgánica.m
Sample Name OBG_469
Comment

Operator Operator
Instrument impact II 1825265.10101

**Acquisition Parameter**

| | | | | | |
|---|---|---|---|---|---|
| Source Type | ESI | Ion Polarity | Positive | Set Nebulizer | 2.5 Bar |
| Focus | Active | Set Capillary | 4000 V | Set Dry Heater | 250 °C |
| Scan Begin | 50 m/z | Set End Plate Offset | -500 V | Set Dry Gas | 6.0 l/min |
| Scan End | 1500 m/z | Set Charging Voltage | 2000 V | Set Divert Valve | Source |
| | | Set Corona | 0 nA | Set APCI Heater | 0 °C |

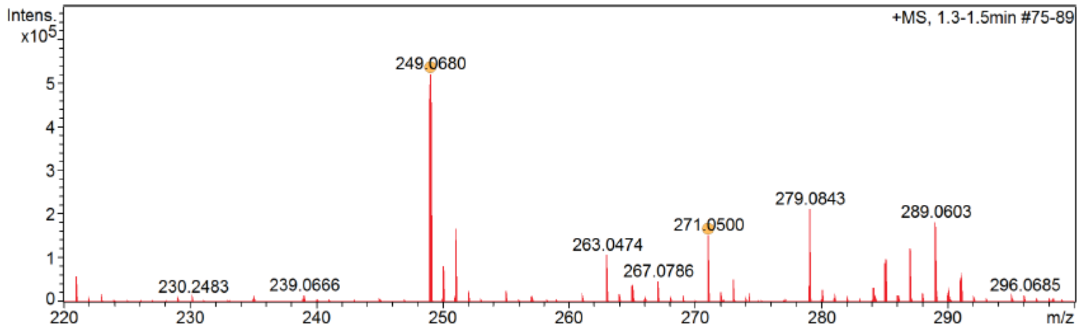


| Meas. m/z | # | Ion Formula | m/z | err [ppm] | mSigma | # mSigma | Score | rdb | e⁻ Conf | N-Rule | Adduct |
|---|---|---|---|---|---|---|---|---|---|---|---|
| 249.0680 | 1 | C14H14ClO2 | 249.0677 | -1.4 | 7.2 | 1 | 100.00 | 8.0 | even | ok | M+H |
| 271.0500 | 1 | C14H13ClNaO2 | 271.0496 | -1.5 | 3.1 | 3 | 92.88 | 8.0 | even | ok | M+Na |

## HRMS compound 7ae

# Mass Spectrum SmartFormula Report

**Analysis Info**

Acquisition Date 21/12/2020 14:00:27

Analysis Name D:\Data\Masa_Exacta\OBG_477_.d
Method MS 50-1000 orgánica.m
Sample Name OBG_477_
Comment

Operator Operator
Instrument impact II 1825265.10101

**Acquisition Parameter**

| | | | | | |
|---|---|---|---|---|---|
| Source Type | ESI | Ion Polarity | Positive | Set Nebulizer | 2.5 Bar |
| Focus | Active | Set Capillary | 3500 V | Set Dry Heater | 250 °C |
| Scan Begin | 50 m/z | Set End Plate Offset | -500 V | Set Dry Gas | 6.0 l/min |
| Scan End | 1500 m/z | Set Charging Voltage | 2000 V | Set Divert Valve | Source |
| | | Set Corona | 0 nA | Set APCI Heater | 0 °C |

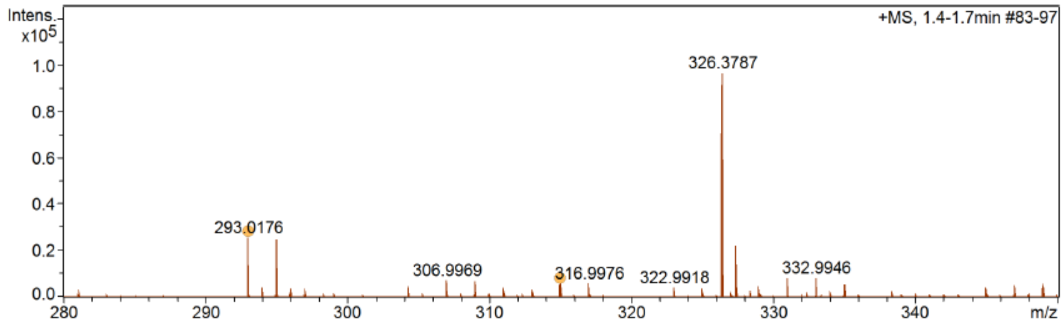


| Meas. m/z | # | Ion Formula | m/z | err [ppm] | mSigma | # mSigma | Score | rdb | e⁻ Conf | N-Rule | Adduct |
|---|---|---|---|---|---|---|---|---|---|---|---|
| 293.0176 | 1 | C14H14BrO2 | 293.0172 | -1.5 | 10.3 | 1 | 100.00 | 8.0 | even | ok | M+H |
| 314.9998 | 1 | C14H13BrNaO2 | 314.9991 | -2.1 | 16.9 | 1 | 100.00 | 8.0 | even | ok | M+Na |

**HRMS compound 7af**

# Mass Spectrum SmartFormula Report

**Analysis Info**

| | | | |
|---|---|---|---|
| Analysis Name | D:\Data\Masa_Exacta\OBG_505.d | Acquisition Date | 21/12/2020 13:19:40 |
| Method | MS 50-1000 orgánica.m | Operator | Operator |
| Sample Name | OBG_505 | Instrument | impact II 1825265.10101 |
| Comment | | | |

**Acquisition Parameter**

| | | | | | |
|---|---|---|---|---|---|
| Source Type | ESI | Ion Polarity | Positive | Set Nebulizer | 2.5 Bar |
| Focus | Active | Set Capillary | 4500 V | Set Dry Heater | 250 °C |
| Scan Begin | 50 m/z | Set End Plate Offset | -500 V | Set Dry Gas | 6.0 l/min |
| Scan End | 1500 m/z | Set Charging Voltage | 2000 V | Set Divert Valve | Source |
| | | Set Corona | 0 nA | Set APCI Heater | 0 °C |

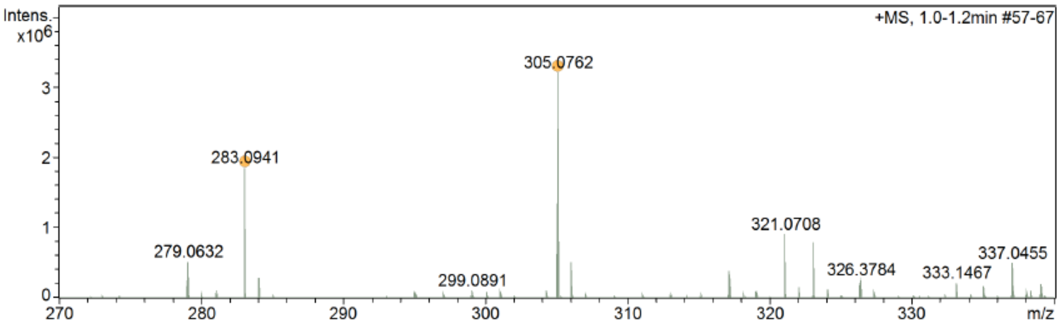


| Meas. m/z | # | Ion Formula | m/z | err [ppm] | mSigma | # mSigma | Score | rdb | e⁻ Conf | N-Rule | Adduct |
|---|---|---|---|---|---|---|---|---|---|---|---|
| 283.0941 | 1 | C15H14F3O2 | 283.0940 | -0.3 | 6.6 | 1 | 100.00 | 8.0 | even | ok | M+H |
| 305.0762 | 1 | C15H13F3NaO2 | 305.0760 | -0.6 | 1.5 | 1 | 100.00 | 8.0 | even | ok | M+Na |

**HRMS compound 7ah**

# Mass Spectrum SmartFormula Report

**Analysis Info**

| | | | |
|---|---|---|---|
| Analysis Name | D:\Data\Masa_Exacta\OBG_482.d | Acquisition Date | 21/12/2020 13:52:02 |
| Method | MS 50-1000 orgánica.m | Operator | Operator |
| Sample Name | OBG_482 | Instrument | impact II 1825265.10101 |
| Comment | | | |

**Acquisition Parameter**

| | | | | | |
|---|---|---|---|---|---|
| Source Type | ESI | Ion Polarity | Positive | Set Nebulizer | 2.5 Bar |
| Focus | Active | Set Capillary | 3500 V | Set Dry Heater | 250 °C |
| Scan Begin | 50 m/z | Set End Plate Offset | -500 V | Set Dry Gas | 6.0 l/min |
| Scan End | 1500 m/z | Set Charging Voltage | 2000 V | Set Divert Valve | Source |
| | | Set Corona | 0 nA | Set APCI Heater | 0 °C |

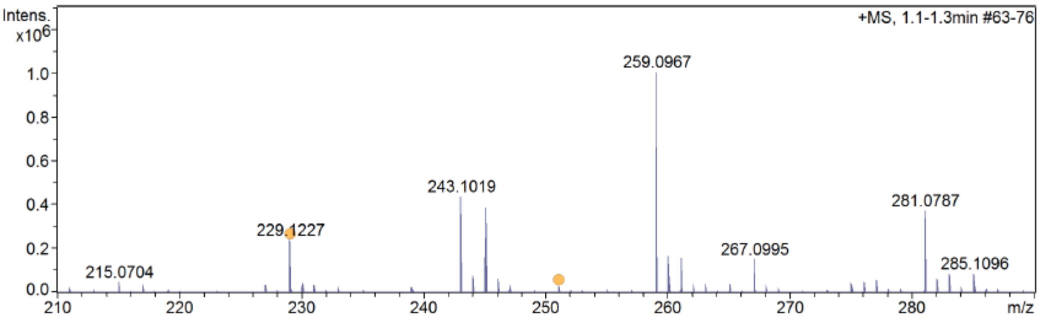


| Meas. m/z | # | Ion Formula | m/z | err [ppm] | mSigma | # mSigma | Score | rdb | e⁻ Conf | N-Rule | Adduct |
|---|---|---|---|---|---|---|---|---|---|---|---|
| 229.1227 | 1 | C15H17O2 | 229.1223 | -1.7 | 3.8 | 1 | 100.00 | 8.0 | even | ok | M+H |
| 251.1046 | 1 | C15H16NaO2 | 251.1043 | -1.5 | 33.9 | 2 | 73.07 | 8.0 | even | ok | M+Na |

## HRMS compound 7bi

# Mass Spectrum SmartFormula Report

**Analysis Info**

Acquisition Date 3/23/2021 1:45:37 PM

Analysis Name \\IMPACT_II-10101\Data\Masa_Exacta\OBG_591.d
Method MS_MASA_EXACTA.m
Sample Name OBG_591
Comment

Operator Demo User
Instrument impact II 1825265.10101

**Acquisition Parameter**

| | | | | | |
|---|---|---|---|---|---|
| Source Type | ESI | Ion Polarity | Positive | Set Nebulizer | 2.4 Bar |
| Focus | Active | Set Capillary | 4000 V | Set Dry Heater | 250 °C |
| Scan Begin | 50 m/z | Set End Plate Offset | -500 V | Set Dry Gas | 6.0 l/min |
| Scan End | 1500 m/z | Set Charging Voltage | 2000 V | Set Divert Valve | Source |
| | | Set Corona | 0 nA | Set APCI Heater | 0 °C |

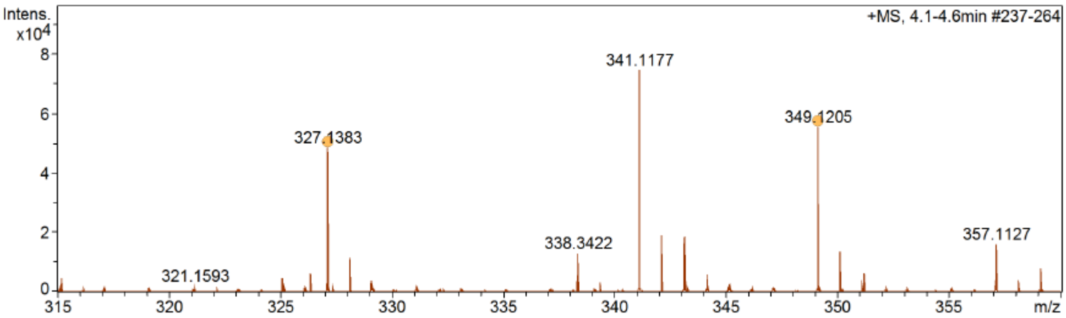


| Meas. m/z | # | Ion Formula | m/z | err [ppm] | mSigma | # mSigma | Score | rdb | e⁻ Conf | N-Rule | Adduct |
|---|---|---|---|---|---|---|---|---|---|---|---|
| 327.1383 | 1 | C23H19O2 | 327.1380 | -0.9 | 9.9 | 1 | 100.00 | 15.0 | even | ok | M+H |
| 349.1205 | 1 | C23H18NaO2 | 349.1199 | -1.6 | 2.8 | 2 | 100.00 | 15.0 | even | ok | M+Na |

## HRMS compound 7aj

# Mass Spectrum SmartFormula Report

**Analysis Info**

Acquisition Date 21/12/2020 12:43:47

Analysis Name D:\Data\OBG_467.d
Method MS_MASA_EXACTA.m
Sample Name OBG_467
Comment

Operator Operator
Instrument impact II 1825265.10101

**Acquisition Parameter**

| | | | | | |
|---|---|---|---|---|---|
| Source Type | ESI | Ion Polarity | Positive | Set Nebulizer | 2.4 Bar |
| Focus | Active | Set Capillary | 4000 V | Set Dry Heater | 250 °C |
| Scan Begin | 50 m/z | Set End Plate Offset | -500 V | Set Dry Gas | 6.0 l/min |
| Scan End | 1500 m/z | Set Charging Voltage | 2000 V | Set Divert Valve | Source |
| | | Set Corona | 0 nA | Set APCI Heater | 0 °C |

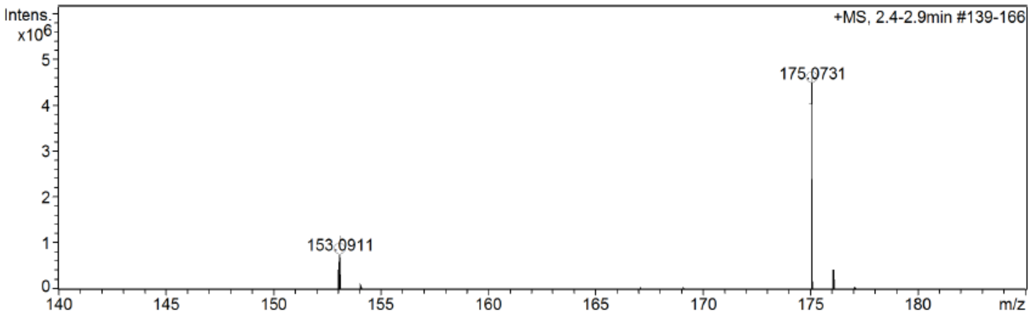


| Meas. m/z | # | Ion Formula | m/z | err [ppm] | mSigma | # mSigma | Score | rdb | e⁻ Conf | N-Rule | Adduct |
|---|---|---|---|---|---|---|---|---|---|---|---|
| 153.0911 | 1 | C9H13O2 | 153.0910 | -0.5 | 4.5 | 1 | 100.00 | 4.0 | even | ok | M+H |
| 175.0731 | 1 | C9H12NaO2 | 175.0730 | -1.0 | 3.9 | 1 | 100.00 | 4.0 | even | ok | M+Na |

## HRMS compound 7ak

# Mass Spectrum SmartFormula Report

**Analysis Info**

| | | | |
|---|---|---|---|
| Analysis Name | D:\Data\OBG_413_BA1_01_5444.d | Acquisition Date | 10/08/2020 13:25:08 |
| Method | FantasmaMASAEXACTA_POS.m | Operator | Demo User |
| Sample Name | OBG_413 | Instrument | impact II 1825265.10101 |
| Comment | | | |

**Acquisition Parameter**

| | | | | | |
|---|---|---|---|---|---|
| Source Type | ESI | Ion Polarity | Positive | Set Nebulizer | 2.4 Bar |
| Focus | Active | Set Capillary | 3500 V | Set Dry Heater | 250 °C |
| Scan Begin | 50 m/z | Set End Plate Offset | -500 V | Set Dry Gas | 6.0 l/min |
| Scan End | 1500 m/z | Set Charging Voltage | 2000 V | Set Divert Valve | Source |
| | | Set Corona | 0 nA | Set APCI Heater | 0 °C |

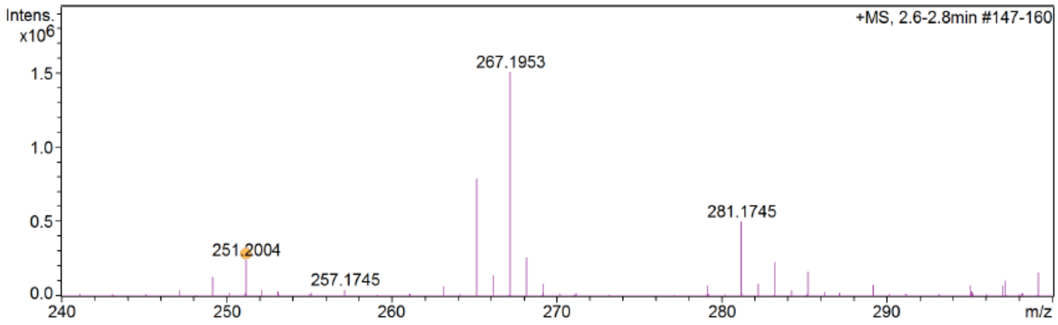


| Meas. m/z | # | Ion Formula | m/z | err [ppm] | mSigma | # mSigma | Score | rdb | e⁻ Conf | N-Rule | Adduct |
|---|---|---|---|---|---|---|---|---|---|---|---|
| 251.2004 | 1 | C16H27O2 | 251.2006 | 0.7 | 2.5 | 1 | 100.00 | 4.0 | even | ok | M+H |

## HRMS compound 7al

# Mass Spectrum SmartFormula Report

**Analysis Info**

| | | | |
|---|---|---|---|
| Analysis Name | D:\Data\Masa_Exacta\OBG_459.d | Acquisition Date | 21/12/2020 14:09:40 |
| Method | MS 50-1000 orgánica.m | Operator | Operator |
| Sample Name | OBG_459 | Instrument | impact II 1825265.10101 |
| Comment | | | |

**Acquisition Parameter**

| | | | | | |
|---|---|---|---|---|---|
| Source Type | ESI | Ion Polarity | Positive | Set Nebulizer | 2.5 Bar |
| Focus | Active | Set Capillary | 3500 V | Set Dry Heater | 250 °C |
| Scan Begin | 50 m/z | Set End Plate Offset | -500 V | Set Dry Gas | 6.0 l/min |
| Scan End | 1500 m/z | Set Charging Voltage | 2000 V | Set Divert Valve | Source |
| | | Set Corona | 0 nA | Set APCI Heater | 0 °C |

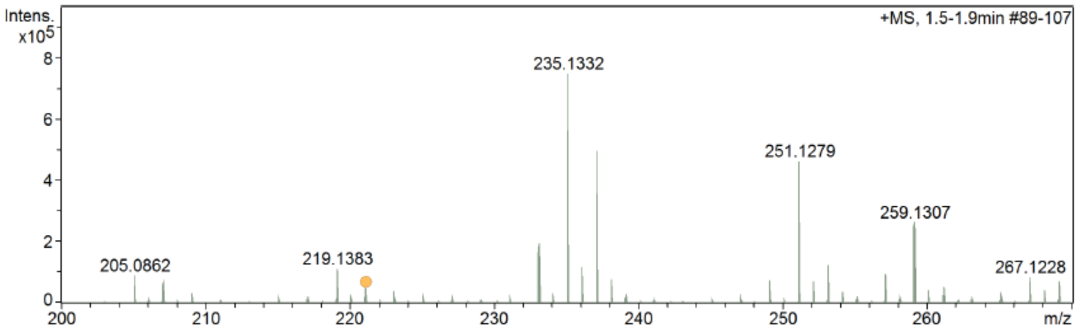


| Meas. m/z | # | Ion Formula | m/z | err [ppm] | mSigma | # mSigma | Score | rdb | e⁻ Conf | N-Rule | Adduct |
|---|---|---|---|---|---|---|---|---|---|---|---|
| 221.1539 | 1 | C14H21O2 | 221.1536 | -1.3 | 8.9 | 1 | 100.00 | 5.0 | even | ok | M+H |
